# The First Molecular Phylogeny of Strepsiptera (Insecta) Reveals an Early Burst of Molecular Evolution Correlated with the Transition to Endoparasitism

**DOI:** 10.1371/journal.pone.0021206

**Published:** 2011-06-28

**Authors:** Dino P. McMahon, Alexander Hayward, Jeyaraney Kathirithamby

**Affiliations:** Department of Zoology, University of Oxford, Oxford, United Kingdom; Laboratoire Arago, France

## Abstract

A comprehensive model of evolution requires an understanding of the relationship between selection at the molecular and phenotypic level. We investigate this in Strepsiptera, an order of endoparasitic insects whose evolutionary biology is poorly studied. We present the first molecular phylogeny of Strepsiptera, and use this as a framework to investigate the association between parasitism and molecular evolution. We find evidence of a significant burst in the rate of molecular evolution in the early history of Strepsiptera. The evolution of morphological traits linked to parasitism is significantly correlated with the pattern in molecular rate. The correlated burst in genotypic-phenotypic evolution precedes the main phase of strepsipteran diversification, which is characterised by the return to a low and even molecular rate, and a period of relative morphological stability. These findings suggest that the transition to endoparasitism led to relaxation of selective constraint in the strepsipteran genome. Our results indicate that a parasitic lifestyle can affect the rate of molecular evolution, although other causal life-history traits correlated with parasitism may also play an important role.

## Introduction

A central focus in evolutionary research is the interaction between molecular evolution and selection at the level of the phenotype, the interface of which unifies aspects of evolutionary research often examined independently [1]. Such an approach offers insight into the factors shaping the rate of molecular evolution, and into the link between genome evolution and species divergence [2]. Strepsiptera is an order of insect parasitoids which display a variety of unusual genetic and phenotypic features [3]–[8]. Targeting groups with complex biologies such as Strepsiptera is useful for testing the validity and generality of ecological and evolutionary theory [9], [10]. However, insufficient molecular data have prevented the study of a number of interesting questions, such as the relationship between genotypic and phenotypic evolution. Strepsiptera display characteristics that are close to the parasite/parasitoid boundary [4], [11]. Female morphology is highly derived (eyes, antennae, mouthparts, legs, wings and reproductive characters are lost) and accompanied by an endoparasitic lifestyle that is host-dependent throughout the lifecycle (except for the family Mengenillidae). In contrast the male is free-flying as an adult and possesses typical insect characteristics. Strepsiptera infect a broad range of hosts, and are recorded from at least 34 families of insects distributed across 7 orders [3], [4], [12]. As with many other parasitic taxa, relatively little research has examined the evolution of host-usage in the group and its effect on speciation. However, recognition of the contribution of parasitic taxa to total animal diversity [13] has emphasised the need to understand the basis of parasite diversification and host usage [14].

The relationship between genotype and phenotype can be examined in a variety of ways. Positive selection in candidate gene phylogenies has been paired with extant phenotypic traits on terminal or internal branches of a phylogeny [15], [16]. Alternatively, a null model of evolution can be compared against models that specify positive selection [17]. These methods specifically target associations in genes responsible for particular phenotypic adaptations (i.e. those under positive selection). Another approach focuses instead on the differing molecular evolutionary rates between species, and in identifying potential life-history traits that influence molecular evolution. Understanding is limited by the availability of suitable methodology, since the field has emerged recently in response to increased DNA sequence data [2]. Investigations have searched for meaningful associations between the rate of molecular evolution and key phenotypic or other extrinsic factors [18], [19], or between phenotypic factors and significant shifts in the pattern of lineage diversification [20]. Results from such studies must be carefully interpreted [21], [22], due to errors associated with phylogeny and rate estimation, or ancestral state reconstruction [1], [2], [23].

Here we investigate the nature and underlying cause of a common feature of higher-level insect phylogenetic analyses: the long-branch separating Strepsiptera from other insect groups [24]–[30]. We explore the link between the strepsipteran phenotype's evolution and: i) variation in the rate of molecular evolution: ii) the pattern of lineage diversification. We reconstruct the first robust molecular phylogeny of Strepsiptera, and use this as a framework to investigate the history of morphological and host-use evolution. Key characteristics include the loss of compound eyes, antennae, legs, wings and reproductive structures in the female, and modifications to the legs and tarsi, and loss of mandibles in some males. The questions we address include the point at which strepsipteran traits evolved, if they emerged more than once and how they are associated with variation in molecular evolution. We explore the hypothesis that the parasitic lifestyle exerts an effect on the rate of molecular evolution [31], an assertion that few studies have so far been able to support [32], [33]. To do so, we examine molecular rate variation in Strepsiptera, and establish a model of evolutionary history that encompasses both molecular and phenotypic evolution.

## Results

### Strepsiptera phylogeny

We used 41 strepsipteran taxa, across 16 genera, and data from four genes: the mitochondrial genes cytochrome c oxidase I (*cox1*), NADH dehydrogenase I (*nad1*), and *small subunit ribosomal RNA* (*16S rRNA*), and the nuclear gene *small subunit ribosomal RNA* (*18S rRNA*) to generate a final alignment of 3930 nucleotides. This consisted of 967 bp 803 bp, and 2160 bp for the *18S rRNA*, *16S rRNA*, and *cox1*+*nad1* partitions respectively.

**Figure 1:**
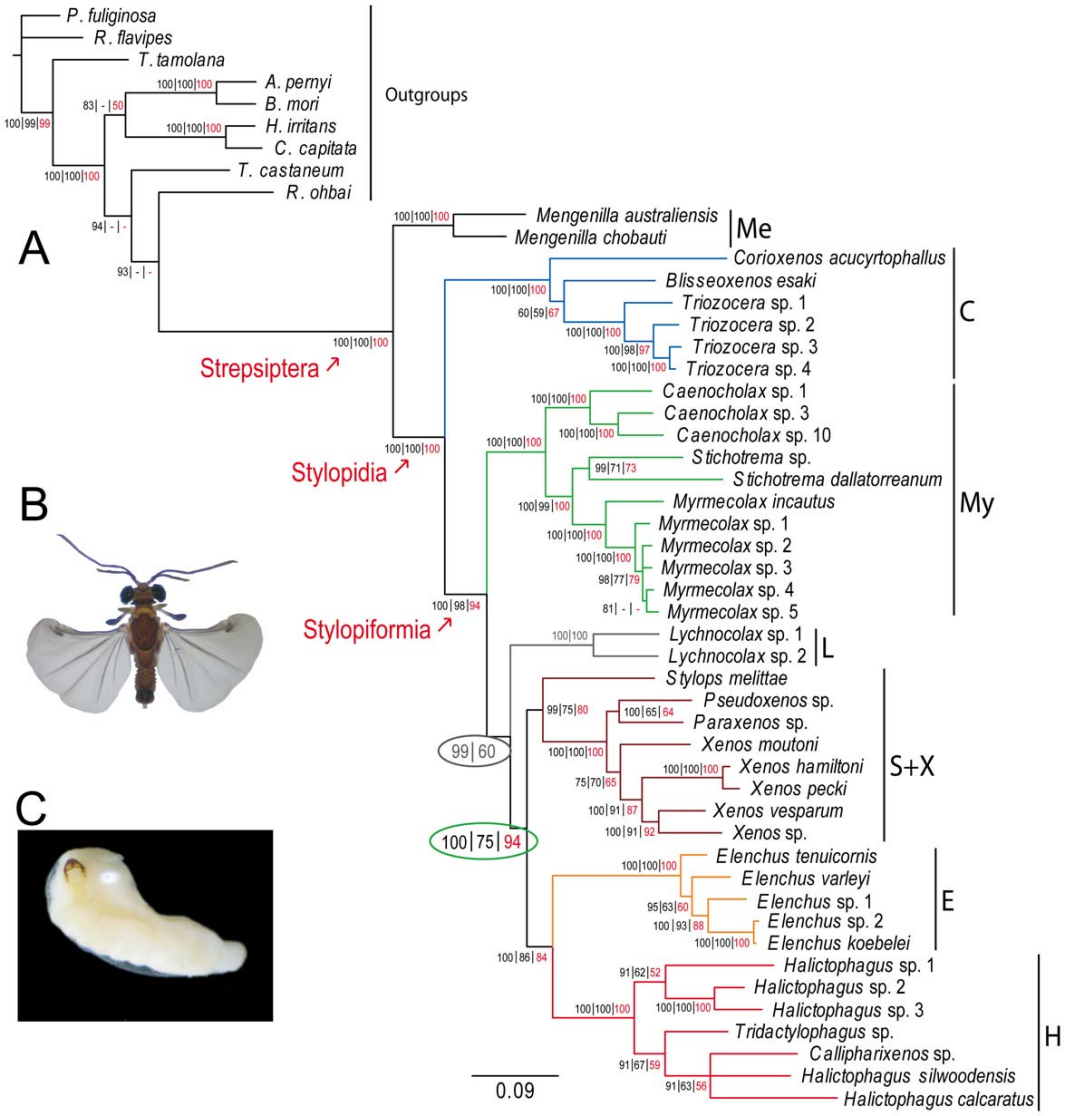
Molecular phylogeny of Strepsiptera. (**A**) Branch lengths from the BI 50% majority rule tree, with support values from BI and ML analyses appearing next to nodes. Support values (%) = BI posterior support | 1000 ML parametric bootstraps with *Lychnocolax* | 500 ML parametric bootstraps without Lychnocolax. Grey oval: support values = BI posterior support | 1000 ML parametric bootstraps including *Lychnocolax*. Green oval: increased ML support following removal of *Lychnocolax*. Me = Mengenillidae; C = Corioxenidae; My = Myrmecolacidae; L = *Lychnocolax*; S+X = Stylopidae+Xenidae; E = Elenchidae; H = Halictophagidae. (**B**) Male *Caenocholax fenyesi* sensu lato (**C**) Female *Caenocholax fenyesi* sensu lato [4].

Across all phylogenetic analyses, we identified monophyletic groupings for extant Strepsiptera, Stylopidia and Stylopiformia, corroborating the findings from previous studies [3], [4], [12], [34] using molecular data for the first time. In the concatenated Bayesian Inference (BI) analyses, all nodes (bar one) at and above the family level receive 100 posterior support (Figure 1A). Maximum Likelihood (ML) bootstrap values are ≥75/100 with *Lychnocolax* (except one: node within grey oval); and ≥80/100 without *Lychnocolax*. We find Myrmecolacidae as the sister-clade to all remaining families within Stylopiformia, and a sister-group relationship between Stylopidae (which parasitize bees) and Xenidae (which parasitize crabronid, sphecid, eumenid and vespid wasps), and between Halictophagidae and Elenchidae that predominantly parasitize Auchenorrhyncha (a “true bug” group, containing amongst others the cicadas, leafhoppers, treehoppers, planthoppers and spittlebugs). The genus *Lychnocolax* has no host records, and was historically placed within Myrmecolacidae [3]. Here, we find evidence that it is an older taxon, as the sister-group to Stylopidae, Xenidae, Elenchidae and Halictophagidae. Its position in the analyses is supported by ribosomal nucleotide composition data (Figure S1), but this is only moderately supported in the concatenated phylogenetic analyses (Figure 1A, grey oval). Removal of *Lychnocolax* led to increased ML bootstrap support in a descendent node (Figure 1A; green oval). Genera are all returned as monophyletic except *Halictophagus*, which occurred as a poorly resolved polyphyletic grouping with *Tridactylophagus* and *Callipharixenos*. The latter species is placed within Halictophagidae, arguing against the separate family-status hypothesized for this lineage.

**Figure 2:**
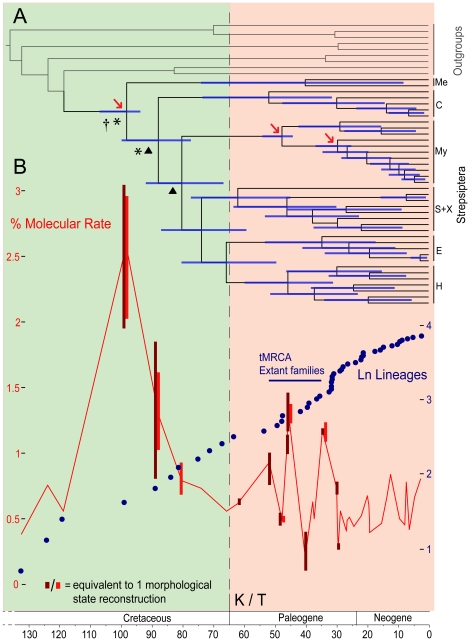
History of divergence and rate of molecular evolution in Strepsiptera. (**A**) BI phylogeny using the MIT1+2 dataset calibrated against time. Node age 95% credibility intervals are indicated over nodes. †Increased relative rate of *18S rRNA*. *Increased relative rate of MIT1+2. ▴Significant Relative Cladogenesis (RCT) statistics. Arrows indicate fossil calibrated nodes. Clade abbreviations follow Figure 1. (**B**) History of molecular rate using MIT1+2 scaled to the tree in panel A with number of ancestral character reconstructions at corresponding nodes (dark red bars = total non-homoplastic state changes in morphology under parsimony [34], red bars = total morphological reconstructions using Bayesian ancestral reconstruction). Blue: Log number of lineages at corresponding distance from root. tMRCA = time to Most Recent Common Ancestor; K/T = Cretaceous/Tertiary boundary.

The dated phylogeny based on the MIT1+2 dataset (see methods) is given in Figure 2, with 95% credibility interval (CI) bars positioned over relevant nodes. The tree reflects the topology produced using the entire (concatenated) dataset presented in Figure 1 (based on the nuclear *18S rRNA*+ mitochondrial *16S rRNA*+ *cox1/nad1* partitions), although there is minor incongruence within Myrmecolacidae, Xenidae, Halictophagidae, Elenchidae. Differences between MIT1+2 and MIT123 (the mitochondrial dataset including 3^rd^ codon positions) on date estimation was minor, with a marginal increase in 95% CIs using MIT1+2 (Table S1). Imprecise CIs concentrate in regions less well informed by available fossil prior information.

### Molecular evolution

Firstly, we investigated the history of molecular rate across the MIT1+2 and MIT123 phylogenies. We found molecular rate estimates ranging from 1–1.5% pairwise sequence divergence per million years (Table S1) for analyses including 3^rd^ codon positions. These are lower than the commonly cited value of 2.3% [35] and more in-keeping with 1.5% [36] for insect mitochondrial DNA and other rates reported for Strepsiptera [10]. However, within this overall pattern, the dated trees and relative rate analyses revealed significant variation in molecular rate, notably at the time to most recent common ancestor (tMRCA) of Strepsiptera and Stylopidia (Figure 2). Both nodes are associated with clades with high relative rates of molecular evolution. The rates at descendent strepsipteran nodes are lower, at around 1% pairwise sequence divergence per million years. The pattern of molecular evolution in the nuclear *18S rRNA* gene is in good overall agreement with the mitochondrial *cox1*+*nad1* gene (MIT1+2, MIT123) datasets (Table S2, Figure S2).

We then compared the evolution of molecular rate with an investigation into the history of diversification rate. The relative cladogenesis test (RCT) indicated a significant shift in Stylopidia and Stylopiformia (Figure 2). But statistics from the topological method in Symmetree were not significant, with upper and lower bound confidence intervals (CI) (at .025 and .975 frequentiles) of 0.079–0.168 and 0.042–0.095 in the *M_R_* and *M_Σ_* tests respectively. Inclusion of 560 missing taxa produced p-value CIs for whole-tree test statistics (*M_R_*; *I_C_*; *M_Π_* *; *M_Π_*; *M_Σ_* *; *M_Σ_*; *B_1_*) between 0.001 - 0.000. This discrepancy could stem from over-representation of Mengenillidae and Corioxenidae, and under-representation of Stylopidae, Halictopaghidae and Myrmecolacidae in the taxon set. In both analyses, individual nodes were not associated with rate shifts, with p-values of 0.121 and 0.209 and 0.107 and 0.185 (with missing taxa) for Stylopidia and Stylopiformia respectively. Furthermore, the branching pattern from the maximum clade credibility (MCC) tree did not depart significantly from a constant-rate/null speciation model: 0.999 (*b* = 0.5, *d* = 0.5, *m* = 560); 0.998 (*b* = 0.5, *d* = 0.0, *m* = 560); 0.836 (*b* = 0.5, *d* = 0.5, *m* = 60); 0.701 (*b* = 0.5, *d* = 0.0, *m* = 60). These results indicate that a burst of molecular rate evolution characterised the early evolution of Strepsiptera, but this did not coincide with a significant shift in lineage diversification.

### Reconstruction of the strepsipteran phenotype

Having established a basic framework for genotypic evolution, we directed attention towards understanding evolution of the strepsipteran phenotype. The morphological character reconstructions used in BI approach are summarized over the MIT1+2 phylogeny in Figure 2, details of the character state reconstructions that were recovered at each node can be found in Table S3. These corroborate the reconstruction of morphological evolution from a previous phylogeny using parsimony [34]. Both closely mirror the pattern of molecular rate depicted in Figure 2.

The long-branch leading to Strepsiptera is linked with phenotypic modifications relating to extreme sexual dimorphism, obligate endoparasitism in the larval stages, and entomophagy (consumption of insects as food). Stylopidia is associated with the evolution of the endoparasitic female (and the continuation of endoparasitism through pupation for males). In males, this node is linked to the reduction or loss of spiracles in the adult and larvae respectively, and the loss of pupal claws. Stylopiformia is associated with modifications to the tarsi, reduction of tarsal number and loss of larval legs in males, and the evolution of the cephalothorax in females.

**Figure 3:**
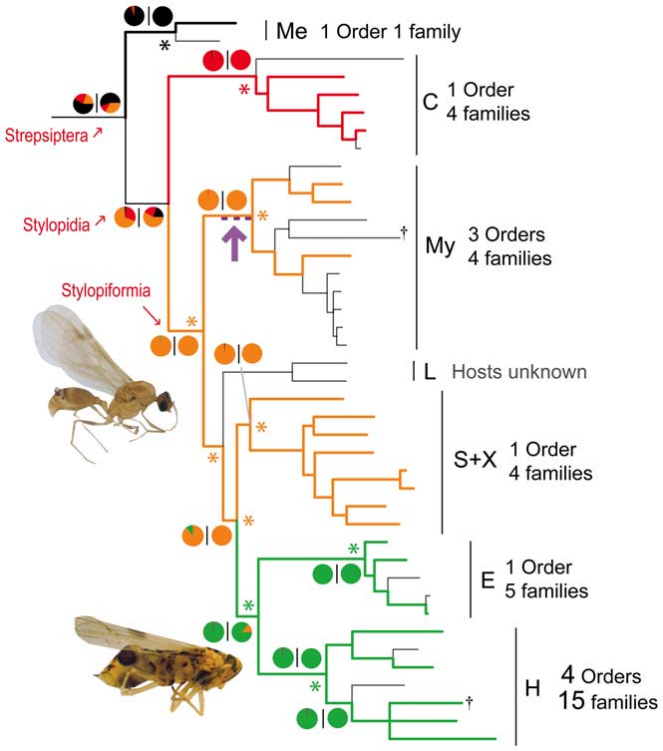
Bayesian reconstruction of male host-usage according to infra-ordinal grouping. Known host records are given next to clades. Unshaded lines = unknown records/equivocal reconstructions. Black = Lepismatidae; Red = Heteroptera; Orange = Hymenoptera; Green = Auchenorrhyncha; Purple = possible origin of heteronomy. *Significant node reconstructions using BFs. Pie charts = posterior probability | ML support. BFs and support charts not shown below family. †Probable parthenogens. Clade abbreviations follow Figure 1B. Images: *Pheidole* sp. (Hymenoptera) with male cephalotheca (top). *Sogatella furcifera* (Homoptera) with *Elenchus japonicas* male puparium. Photographs © J. Kathirithamby.

The history of strepsipteran host-use is summarized in Figure 3. Parasitization of aculeate hymenopterans is predicted to have originated in Stylopiformia, or possibly earlier in the ancestor to Stylopidia, where a secondary switch would be implicated in Corioxenidae to Heteroptera (a “true bug” group, containing amongst others the assassin bugs, bed bugs, seed bugs and shield bugs). In both models, a subsequent switch to Auchenorrhyncha in the ancestor of Elenchidae and Halictophagidae is strongly supported. Outside of Myrmecolacidae, host switching between infraordinal host groups occurs only in Halictophagidae. The ancestral host of Strepsiptera remains unresolved given currently available data.

### Comparison of molecular and phenotypic rates of evolution

**Figure 4:**
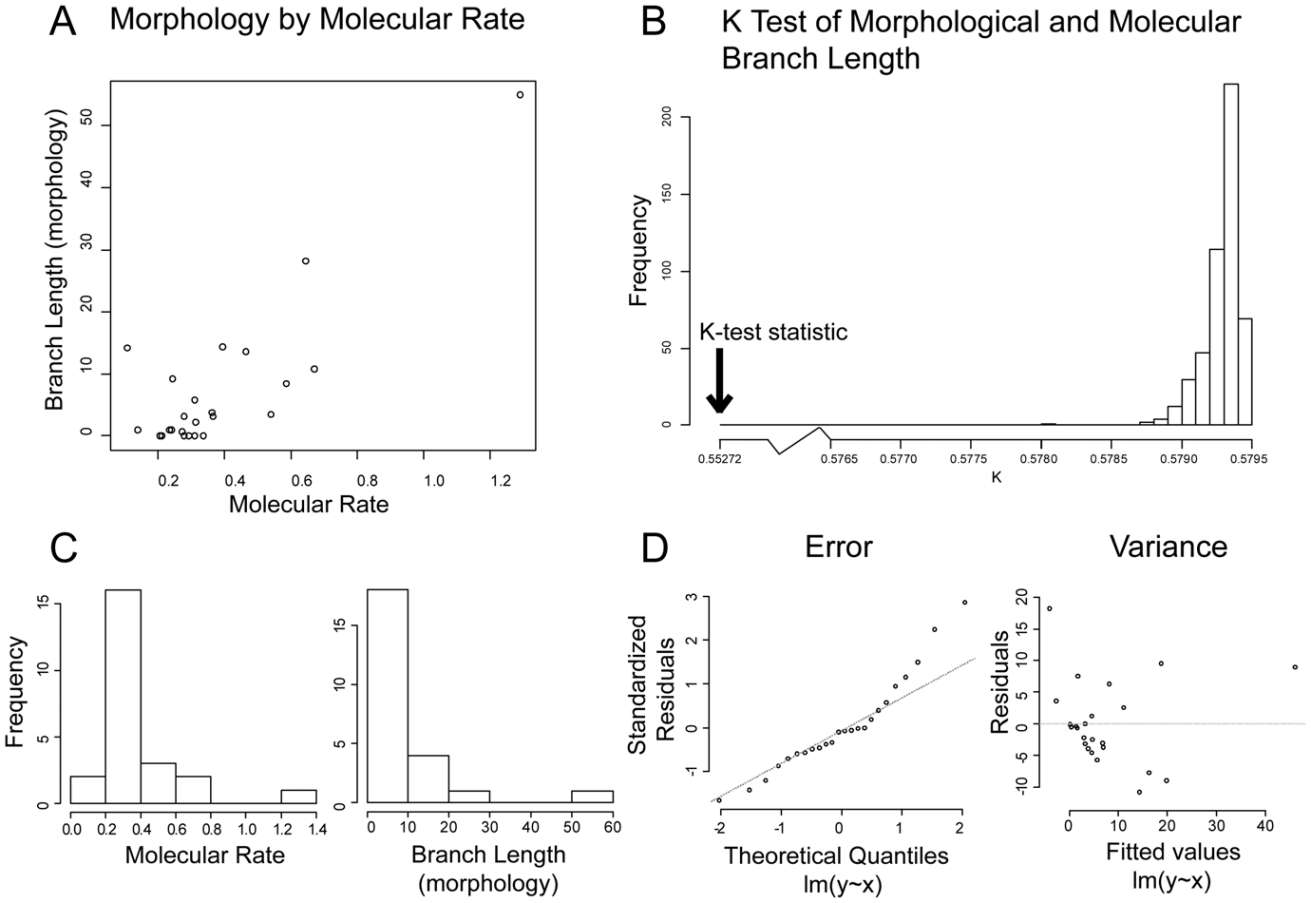
Graphical summary of molecular rate and morphological branch length variation. (**A**) Individual node comparison of molecular rate (pairwise sequence divergence / million years) versus morphological branch length (steps required under parsimony). (**B**) K-tree null distribution, with test-statistic indicated by arrow. (**C**) Distribution of molecular rate (left) and morphological branch length (right) variation. (**D**) Graphical summary of linear model assumptions: non-linearity of error (left) and heterogeneity of variance (right).

We undertook a number of analyses to test the statistical validity of the association between molecular and phenotypic rates of evolution. A linear model indicated that molecular rate is positively associated with morphological branch length variation (T-statistic = 7.360, p-value = 2.29E-07). Molecular rate contributed the majority of variation in branch length (Adjusted R-squared = 0.698). However, non-linearity of error and heterogeneity of variance undermined the assumptions of a parametric statistical approach. The concentration of molecular and morphological rate evolution in the node leading to Strepsiptera represents a significant component of the skew in the distribution (Figure 4). We therefore re-examined the correlation by using a spearman test (*rho* = 0.542, S = 1054.628, p-value = 0.003) (Figure 4A).

We also took an alternative approach by making a whole-tree comparison of morphological versus molecular branch lengths (instead of molecular rate). We generated a null distribution of K-scores (mean = 0.579, S.D = 0.000134, min = 0.5780, max = 0.5794), and compared our K-test statistic of 0.553 against this distribution (see methods). The hypothesis that the observed K-score was due to random processes could be rejected (p-value≪0.001) (Figure 4B). Overall, these results indicate a correlated pattern of molecular and morphological evolution in Strepsiptera.

## Discussion

In this study, we develop a framework for understanding strepsipteran molecular evolutionary history, link it with existing knowledge of strepsipteran morphological evolution, and establish a foundation for further research into the evolution and ecology of this unusual host-parasite system. We retrieve high support for the monophyly of Strepsiptera, Stylopidia and Stylopiformia, and for interrelationships between the extant families. There remain areas of uncertainty, in particular the equivocal position of *Lychnocolax* and the polyphyly of *Halictophagus*, which require taxonomic revision through re-analysis of morphology and inclusion of additional taxa and alternative DNA markers.

### Molecular rate, diversification rate, and phenotypic evolution

We detected a significant shift in molecular rate in the early history of Strepsiptera. Instead of remaining uniformly high, the rate returned to a low and even rate across the phylogeny. Minor peaks in molecular rate are also linked with the diversification of lineages post K/T, in particular the tMRCAs of extant families Xenidae, Halictophagidae, and Elenchidae between 50-30 MYA. Interestingly, the K/T boundary was not closely linked with shifts in either molecular rate or diversification rate. A similar pattern has also been observed in other terrestrial animal groups, including mammals [37], squamates and passerine birds [38]. This trend reflects contemporaneous changes in strepsipteran morphology, which also changed significantly during the group's early history, followed by a period of stability in more recent history (<70 MYA), notwithstanding minor modifications to morphology linked with the tMRCAs of several extant families (Figure 2). Relationships within Stylopiformia are inconsistent with the only other (morphology-based) phylogenetic analysis of Strepsiptera. This important result may be due to the low number of non-homoplasious morphological state changes at intermediate depths of the prior study (Figure 29 in [34]). In the current study, inter-node distances are short compared with surrounding branches in the equivalent region of the tree (Figure 1). This is consistent with the individual node comparison of molecular rate and morphological branch length, which show a positive correlation.

The evolution of strepsipteran structural morphology involved a range of adaptations associated with increasingly specialised parasitism. Extant Mengenillidae represent a transitional condition, in that females reproduce and release progeny whilst outside of the host, with the faculty to leave the free-living pupa to lead a motile lifestyle [39]. We hypothesize that complete female endoparasitism in Stylopidia led to strong sexual selection on free-living adult males, which to copulate successfully must engage with highly modified female structures (the cephalothorax) protruding from living hosts. This may have led to the evolution of hairy adhesive tarsal pads; needed to adhere to diverse host substrates during insemination of the endoparasitic female [40].

The diversification rate in Strepsiptera increased after the initial burst in molecular rate, but a significant individual node shift was only detected in the RCT statistic, at the origin of Stylopidia/Stylopiformia. Lack of evidence for an increase in diversification across other methods suggests this result should be interpreted with caution. We refrain from discussing in depth which phenotypic traits (if any) might be causally linked to the main phase of strepsipteran diversification due to difficulties associated with identifying trait(s) that are responsible for speciation [41], [42]. One might hypothesize that after endoparasitism became an obligate component of all aspects of female life-history (in the ancestor of Stylopidia), the evolution of a more effective method of host immune evasion may have enhanced the ability of Strepsiptera to successfully infect novel hosts, thereby opening opportunities for speciation. During infection, Strepsiptera are contained within a host-derived epithelial membrane, which is thought to conceal the endoparasite from the host's immune system [5]. However, its point of origin remains unknown.

### Did parasitism cause the burst in molecular rate?

We discovered a correlated burst in the rate of molecular and morphological evolution, which coincided with significant increases in the relative rate of molecular evolution, and abrupt shifts in the evolution of rRNA structures (Figure S1). We showed in an overall comparison of branch length that the observed similarity between molecular and morphological trees (K-score) was not due to random processes. The traits that evolved during the early history of Strepsiptera were broadly adaptations relating to the evolution to endoparasitism. These results are consistent with the hypothesis that parasitism may be an important cause of molecular evolutionary rate variation [31]–[33]. A plausible scenario could have involved deleterious mutations in free-living species becoming neutral/nearly-neutral in progressively host-dependent endoparasites. An adaptive interpretation could be that parasitism indirectly led to the selection of increased variation (through recombination or mutation), due to increased red-queen pressures between the host and parasite. But such a hypothesis does not explain why a subsequent decrease in substitution rate is observed in descendent nodes within Strepsiptera. Under the first (non adaptive) model, once most sites had been exposed to novel evolutionary forces, the substitution rate returned to a background level. Our findings are more in-keeping with relaxation of selective constraint as a dominating force in the early evolution of Strepsiptera.

However, the precise relationship between molecular rate and parasitism cannot be conclusively resolved in the current framework. Confounding correlates of endoparasitism may prove causally more relevant [2]. For example, in studies of mammalian molecular evolution, the increase in availability of DNA sequence data has questioned initial hypotheses positing a simple correlation with body size. Later studies used more reliable rate estimates and better methods to demonstrate that rate in the nuclear genome covaried with generation time and fecundity (and body size) but that variation in the rate of the mitochondrial genome was explained by longevity [43] (although in our study, rates between nuclear and mitochondrial genomes are similar). An alternative explanation in Strepsiptera could be that endoparasitism enabled females to increase individual fecundity by being able to concentrate more resources on one aspect of life-history: reproduction. Higher mutation rate could have subsequently stemmed from the associated increase in germline replications per generation. Endoparasitism may have also been correlated with increased generation time and shorter lifespan, where pressures to reproduce prior to host-death or clearance are considered to be critical components of parasite evolution [44]. These factors could be causally important in explaining the evolution of molecular rate in Strepsiptera, but determining which requires a more detailed understanding of life-history, ecology and the fine-scale interaction between Strepsiptera and host. Uncovering the mechanism of immune evasion could represent a particularly important target for future research.

### The Strepsiptera long-branch

This study indicates that elevated molecular evolutionary rate was an important contributing cause of the strepsipteran long-branch. However, missing data in the form of undiscovered extinct (or extant) transitional lineages and imprecision over the nearest extant sister-lineage are also relevant to improving understanding of the causes of molecular rate variation in Strepsiptera. A number of recent studies consolidate the view that Strepsiptera are closely related to Coleoptera [27]–[30] but a precise hypothesis has still not been reached. Increased knowledge of strepsipteran life-history and ecology, in combination with a more detailed understanding of strepsipteran sister-relationships, will lead to better estimations of divergence, allowing for more informative date priors to be incorporated into a relaxed phylogenetic approach [45], [46]. Together, these will help to develop a more accurate picture of the forces responsible for variation in the rate of genome evolution in Strepsiptera. Revisiting hypotheses, like a possible association with rhipiphorine beetles [47] may help to identify potential candidate taxa that interrupt the branch. Alongside approaches that implement more sensitive phylogenetic methodology and larger data sets [48], new data may offer greater understanding of strepsipteran origins. However, this study suggests that the strepsipteran long-branch may never be easy to “break up”.

### Conclusions

In this report, we present the first molecular phylogeny of Strepsiptera. Estimates of morphological branch length, alongside reconstruction of the strepsipteran phenotype reveal a correlation between morphological traits linked to endoparasitism and rate of molecular evolution. The main phase of diversification (Stylopidia, Stylopiformia) is associated with a return to a low and even rate of molecular evolution, and a period of relative morphological stability. This pattern supports the hypothesis that the transition to parasitism from a free-living insect ancestor can affect molecular rate. Greater precision over the nearest extant strepsipteran sister group will lead to better estimations of both divergence and molecular rate. Improved understanding of strepsipteran biology will in future permit the causes of molecular rate variation in Strepsiptera to be examined in greater detail. Together, these results establish an important foundation for further research into the evolution and ecology of a highly unusual host-parasite system.

## Materials and Methods

### Taxon and DNA sampling

Individuals were included from 41 strepsipteran taxa, across 16 genera (50% coverage). Bohartillidae and Bahiaxenidae, which are rare and represented by few specimens, were not included [49]. Three hemi- and six holometabolous outgroup species were selected from nucleotide data in Genbank. Due to the nature of mitochondrial gene evolution in Hymenoptera [50], [51] and the possibility of long-branch attraction between Strepsiptera and Hymenoptera (Hayward et al. in preparation; Figure 4 in [27]), the latter were not included. Specimens were preserved at 4°C in 95% ethanol, and protocols employed for sequence generation follow [8]. The mitochondrial genes cytochrome c oxidase I (*cox1*), NADH dehydrogenase I (*nad1*), and *small subunit ribosomal RNA* (*16S rRNA*), and nuclear gene *small subunit ribosomal RNA* (*18S rRNA*) were chosen to represent independent and variable evolutionary rates (Genbank accession JN082786–JN082922). Chromatograms were inspected manually using FinchTV (www.geospiza.com), and *cox1* and *nad1* fragments were aligned by eye in BioEdit [52], using translated nucleotides to guide the management of indels. *16S* and *18S rRNA* fragments were aligned manually using the comparative structural method [6], [53], [54] and mfold [55], but these do not correspond strictly to the category definitions *sensu* Gillespie [54].

A final alignment consisting of 3930 nucleotides was used in subsequent analyses, consisting of 967 bp 803 bp, and 2160 bp for the *18S rRNA*, *16S rRNA*, and *cox1*+*nad1* partitions respectively, each with 339, 433, and 433 parsimony-informative positions. This approach was compared against an automated alignment strategy using the default settings in MUSCLE [56] and Gblocks [57], but retaining columns with a gap at greater than 50% of taxa. The resultant alignment contained 21% fewer characters (3048 nucleotides) of 642 bp, 774 bp and 1632 bp in the *18S rRNA*, *16S rRNA* and *cox1*+*nad1* partitions, each with 229, 546, and 341 parsimony informative positions respectively. All analyses in this study are based on the structurally-informed “manual” alignment as trees based on the automated approach produced trees with limited support and equivocal topologies (data not shown). For the estimation of molecular rates, divergence estimates and date-informed branch lengths, the mitochondrial *cox1* and *nad1* genes were combined into a single data partition and analysed separately with/without the 3^rd^ codon position (datasets MIT1+2 and MIT123 respectively). Specimen information (including accession numbers, primer information and 18S/16S rRNA template alignments) appears in Table S4.

### Evolutionary model selection and phylogenetic analysis

For Bayesian analyses (BI), the most appropriate models of evolution were selected by comparing harmonic means across separate gene partitions in MrBayes v3.1.2 [58], [59], and then calculating Bayes Factor (BF) values. For maximum likelihood (ML), the Akaike Information Criterion (AIC) approach in MrModelTest v2 [60] and ProtTest v2.4 [61]–[63] was employed to select the most suitable models for RAxML v7.0.3 [64]. For all nucleotide partitions, the GTR+Γ+I model was preferred by BF and AIC with the following harmonic means: −7209.42 (*18S rRNA*); −9235.59 (*16S rRNA*); −14796.14 (MIT1+2); −30436.50 (MIT123) and log-likelihoods: −7160.8037; −9189.0898; −14758.0146; −31345.5293. For partition MIT123, the number of transitions and transversions estimated under the F84 model were plotted against genetic distance for each codon position using DAMBE v5.0.8 [65] (Figure S3, Table S5). A test of substitution saturation [66] and quartet likelihood mapping (TREE-PUZZLE v5.2) [67], [68] indicated high percentages of noise versus signal (20.6% and 32.7% in *cox1* and *nad1* respectively) in synonymous 3^rd^ codon position, and little correspondence between 3^rd^ codon position transition frequencies and genetic distance. Consequently, the mixed amino acid model facilitated by MrBayes was selected for use in all concatenated BI analyses. The amino acid substitution model favoured by the posterior density was MtRev [69] +Γ+I (+F). ProtTest found highest support for MtArt [70] and LG [71], but these are unavailable to MrBayes v3.1.2 and RAxML v7.0.3.

After models were selected, concatenated BI analyses consisted of two independent (MC)^3^ algorithms running for 2 million generations, each with four chains (3 hot, 1 cold), sampling one tree in 200, burn-in cutoffs were inspected manually for each parameter file in Tracer v1.4 [72]: the first 40000 steps were discarded. Inspection of the standard deviation of split frequencies confirmed that runs had converged (0.0059). All parameters except topology were unlinked between partitions. Data were summarized over a majority rule consensus tree (50% cutoff). 1000 ML nonparametric bootstrap pseudoreplicates were estimated in RAxML v7.0.3 [73]. 500 ML bootstrap pseudoreplicates without *Lychnocolax* were also estimated. Trees were imported into FigTree v1.2.3 for editing [74].

### Divergence time estimation

MIT1+2 was employed in a Bayesian relaxed clock framework in BEAST v1.4.8 [75] using the GTR+Γ+I model. *Lychnocolax* taxa were removed prior to analyses and Holometabola was constrained as monophyletic. Likelihood ratio tests using least and most complex evolutionary models in PAML v4 [76], with/without the 3^rd^ codon position were overdispersed with respect to a molecular clock (2ΔlnL = 1398.37, 1539.43, 836.45, 948.41; *df* = 7, P<0.001). Significant rate-heterogeneity was accommodated by employing the relaxed-clock MCMC with an uncorrelated lognormal model (UCLN) [77], calibrated using three strepsipteran fossils. The implementation of fossil priors is described in Text S1.

MCMC analyses ran for 10 million iterations, sampling every 1000^th^ step. The effect of A+T-rich 3^rd^ codon positions was investigated using the MIT123 dataset, in which the two partitions (1+2)(3) were unlinked. Analyses were repeated using the *18S rRNA* dataset. The effect of model choice was assessed by comparing GTR+Γ+I with the SRD framework [78]. Molecular rate estimates were calculated as % pairwise sequence divergences per million years: equal to twice the per lineage rate. Dates were specified as millions of years before present, the Yule process was employed as the tree prior. Parameter files were inspected manually to ensure chain stability across parameters, and to select an appropriate burn-in. Tree files were summarized on a maximum clade credibility (MCC) tree.

### Molecular rate, diversification rate, and tree shape

Relative rates of molecular evolution were examined via cross comparison of families and outgroups in RRTree v1.1.11 [79]. Whole-tree and single node methods were employed to test for departures in diversification rate [39]. The temporal rate cladogenesis test (RCT) statistic was calculated [80], [81]: nodes showing a “trickle-down” effect [39] were excluded. Whole tree simulations of rate-constant/rate-variable variants of the birth-death model in the Laser R-package [82] were conducted. The simulation ran for 1000 trees, comparing the best *constant* speciation model versus best *variable* speciation model (ΔAICrc) using the MIT1+2 tree. Outgroup taxa were pruned, and the birth rate (*b*), death rate (*d*) and unsampled taxa (*m*) were varied. *m* represents unsampled Strepsiptera species diversity. SymmeTree v1.1 [83] was used as an independent topological method. To investigate the impact of missing taxa, 560 tips were assigned to known groupings as soft polytomies. Whole-tree and single-node statistics were calculated using 100000 Bayesian simulations.

### Evolutionary trait reconstruction

For reconstructions of morphology, data were imported into BayesTraits v1.0 [84]. Ancestral states were enforced using the ‘fossil’ prior. Harmonic means were compared for fossilized states, and accepted or rejected using BF values. 2 million MCMC iterations were conducted using the final consensus branching pattern and repeated if harmonic means did not stabilize. A reverse-jump hyperprior with exponential distribution 0–30 was set. ‘ratedev’ was optimized so that proposals were accepted 20–40% of the time. For host-use, major infraordinal divisions were treated as states. Aculeata (ants) were placed as the primary (ancestral) host for Myrmecolacidae: males of Myrmecolacidae parasitize only ants [85] and evidence for a female myrmecolacid in a fossilized ant host [86] indicated this was appropriate. A maximum likelihood approach, using the symmetrical method (Mesquite v2.71 [87]) was implemented to offer an independent measure of support.

### Comparison of molecular and phenotypic rates of evolution

State changes corresponding to non-homoplasious steps from a morphological phylogeny of Strepsiptera [34] were mapped to shared nodes of the MIT1+2 MCC tree (excluding branches leading to missing taxa, conflicting nodes, and clades represented by one taxon – representing 6, 6 and 9 steps respectively). A smaller list of discrete adult and secondary larval characters was used in a Bayesian reconstruction approach to ensure this pattern was repeatable across methods.

As a formal comparison of the relationship between molecular and phenotypic evolution, morphological branch lengths were estimated in Phylip [88], using the genus-level matrix of adult and secondary larval characters from [34], updated to include the current set of taxa. The topology was constrained to follow the MIT1+2 dated MCC tree. For individual nodes, we tested the correlation between % pairwise sequence divergence and morphological branch length using standard statistics in R v2.9.2. As an independent whole-tree method, the K-score was calculated using Ktreedist v1.0 [89] and compared against a null K-distribution (500 simulated trees; following [18]). In this approach, molecular branch length (instead of pairwise sequence divergence) was assessed against morphological branch length.

## Supporting Information

Figure S1 **rRNA variabe and core domain structural attributes mapped onto the Strepsiptera phylogeny.** (**A**) *18S* variable (bar) and core (filled circle) A+T% content. (**B**) *16S* variable (bar) and core (filled circle) A+T% content. (**C**) Variable domain size (nucleotide length) for the *18S* (red) and *16S* (black) genes. Outgroups grey and highlighted. Clade abbreviations and colour scheme follow Figure 1. Note the shifts in variable domain bp length, in both the *18S* (length increase) and *16S* (length decrease) genes at the node leading to Strepsiptera in (C).
(TIF)

Figure S2 **Divergence time and molecular rate patterns using the nuclear** ***18S rRNA*** **dataset.** Red: % molecular rate mapped for each node at corresponding distances from root. Blue: *Ln* number of cumulative lineages at corresponding distances from root. This corroborates the analysis using the mitochondrial partition (Figure 2), confirming that the observed pattern is consistent across genomic compartments.
(TIF)

Figure S3 **Exploration of data quality across the mitochondrial genes.** Transitions and transversions estimated under the F84 model were plotted against genetic distance for each codon position: Green = 1sts, Blue = 2nds, Orange = 3rds. Signal versus noise was graphically visualized using quartet likelihood mapping.
(TIF)

Table S1 **Summary of Strepsiptera divergence times.** Summary of divergence time estimates for the major nodes in the Strepsiptera phylogeny using the combined mitochondrial coding gene (*cox1*+*nad1*) partition. *Pairwise sequence divergences per million years. Clade abbreviations follow figure 1. †Node ages defined by exponential priors.
(DOC)

Table S2 **RRTest comparative analysis across strepsipteran clades.** Bold = P-value with significant rate comparison (bonferroni corrected). *Marginally non-significant after bonferroni adjustment in the mitochondrial (A) and *18S rRNA* partition (B). Clade abbreviations follow figure 1.
(DOC)

Table S3 **List of characters and corresponding states recovered in the reconstruction of strepsipteran morphological traits.** The position in the phylogeny of significant character reconstructions appears in brackets next to the corresponding state, followed by the BF range supporting that reconstruction. Some characters may be considered dependent, if single genotypic events can be demonstrated to produce pleiotropic effects. Possible examples include male/female larval spiracles, and male/female larval legs. * equivocal BFs (0.2–3.8). This might disguise a potentially apomorphic loss of tarsomeres in the Elenchidae+Halictophagidae ancestor.
(DOC)

Table S4 **Specimen, primer information and rRNA template alignments.** Genbank accession and specimen source information; list of primers used in this study (*Primers designed for short-fragment PCR) and 18 rRNA and 16S rRNA template secondary structural alignments.
(DOC)

Table S5 **Test of saturation by mitochondrial gene and codon position.** *Statistics indicating little saturation. †Statistics with substantial saturation (bold). ‡Statistics indicating useless/very poor sequence for phylogenetics (bold). Ts = symmetrical T-statistic. Tns = non-symmetrical T-statistic.
(DOC)

Text S1 (DOC)
