# Supplementary figures and images for: The First Molecular Phylogeny of Strepsiptera (Insecta) Reveals an Early Burst of Molecular Evolution Correlated with the Transition to Endoparasitism
Source: PLoS One. 2011 Jun 28;6(6):e21206. doi: 10.1371/journal.pone.0021206 (PMC3125182; doi:10.1371/journal.pone.0021206)

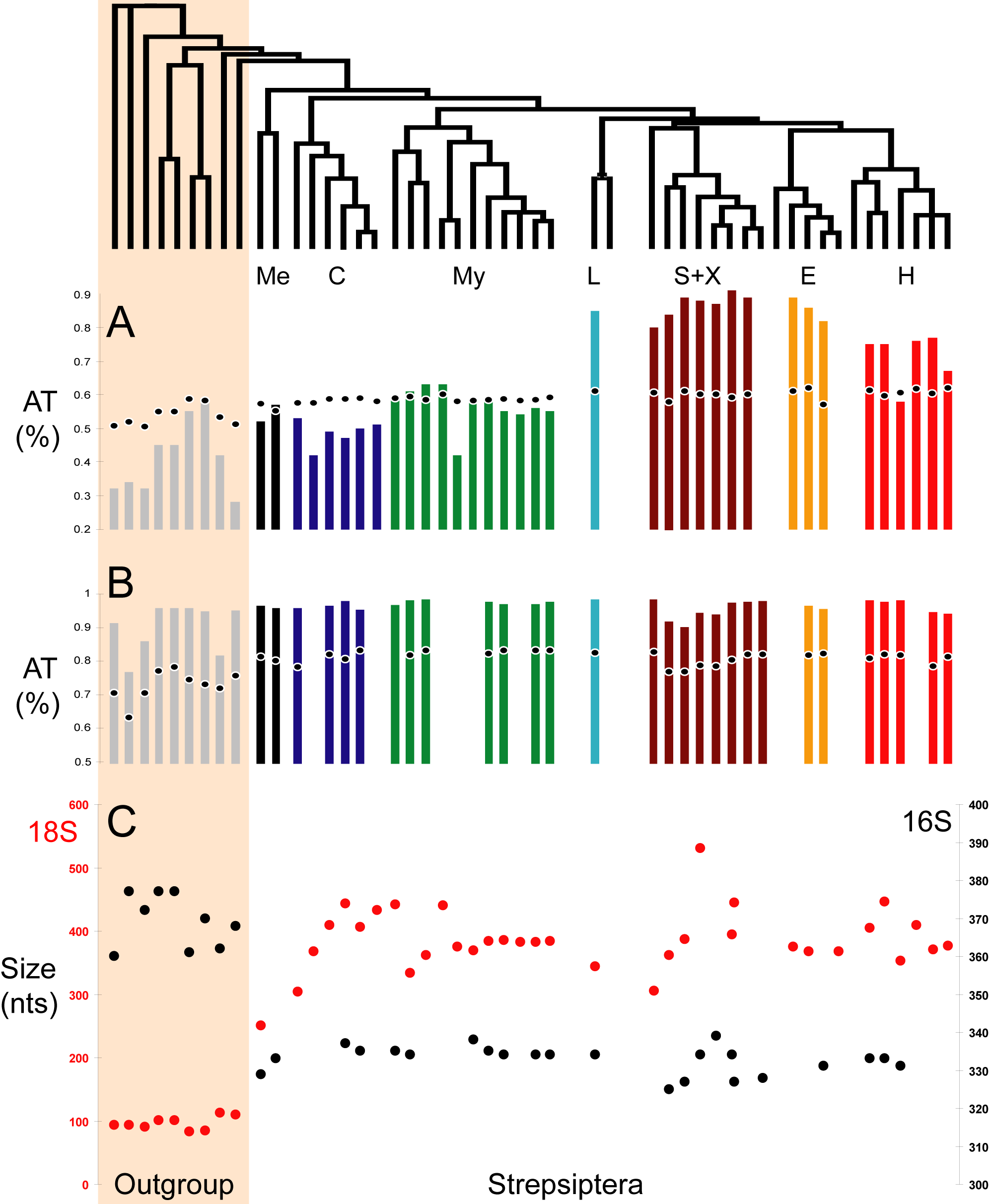

Supplement: Figure S1 — rRNA variabe and core domain structural attributes mapped onto the Strepsiptera phylogeny. (A) 18S variable (bar) and core (filled circle) A+T% content. (B) 16S variable (bar) and core (filled circle) A+T% content. (C) Variable domain size (nucleotide length) for the 18S (red) and 16S (black) genes. Outgroups grey and highlighted. Clade abbreviations and colour scheme follow Figure 1. Note the shifts in variable domain bp length, in both the 18S (length increase) and 16S (length decrease) genes at the node leading to Strepsiptera in (C). (TIF) [file pone.0021206.s001.tif]

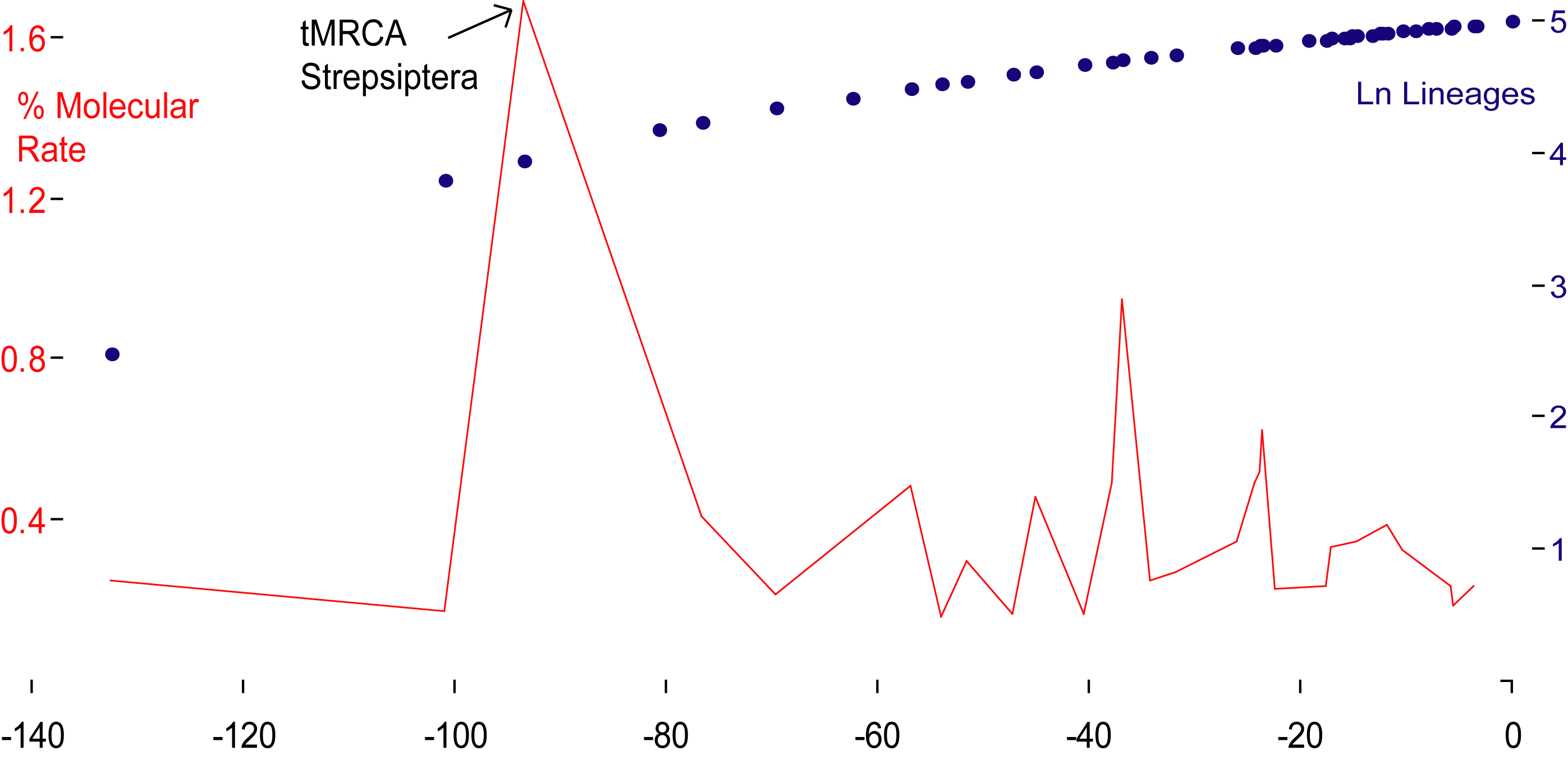

Supplement: Figure S2 — Divergence time and molecular rate patterns using the nuclear 18S rRNA dataset. Red: % molecular rate mapped for each node at corresponding distances from root. Blue: Ln number of cumulative lineages at corresponding distances from root. This corroborates the analysis using the mitochondrial partition (Figure 2), confirming that the observed pattern is consistent across genomic compartments. (TIF) [file pone.0021206.s002.tif]

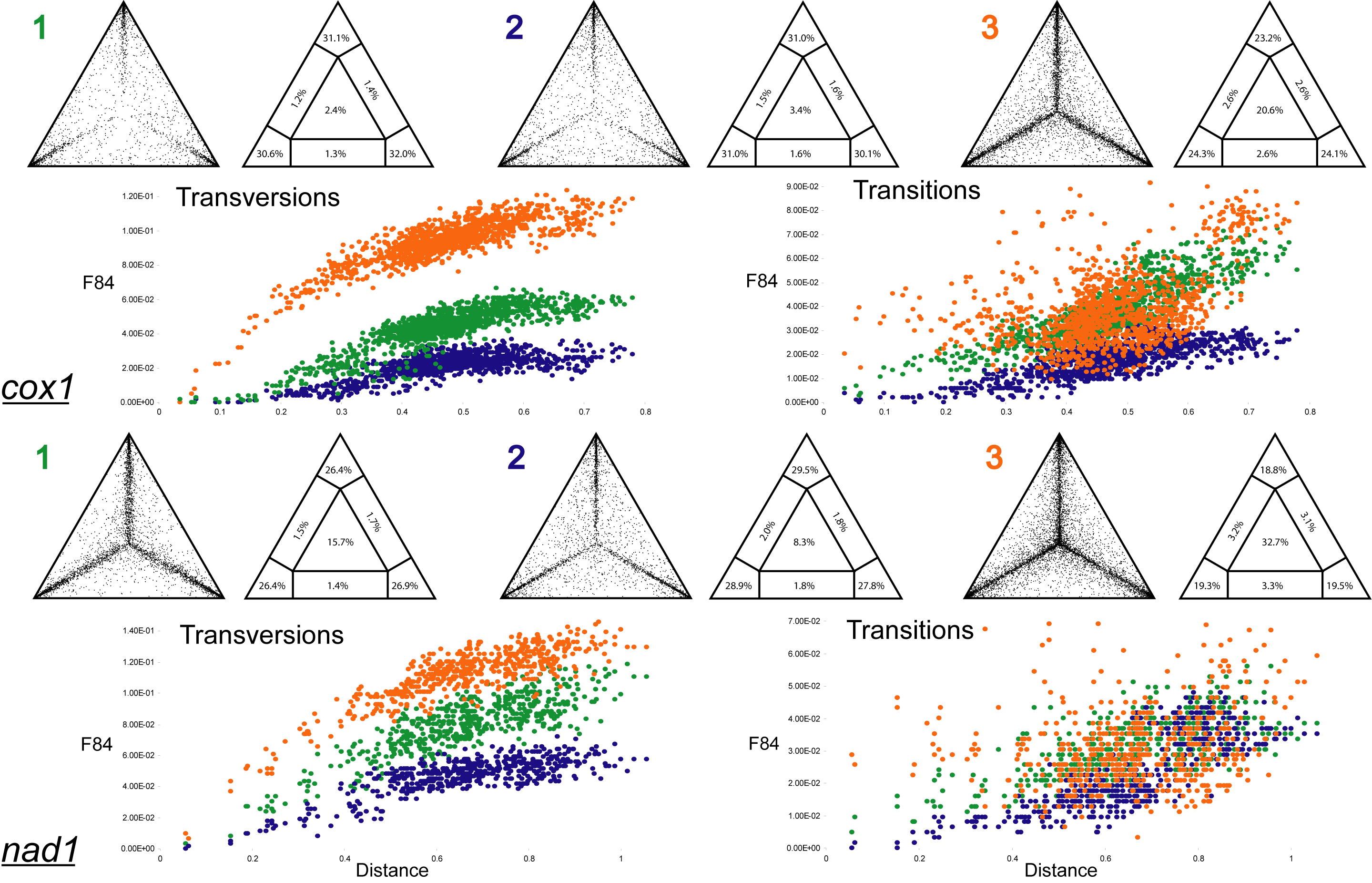

Supplement: Figure S3 — Exploration of data quality across the mitochondrial genes. Transitions and transversions estimated under the F84 model were plotted against genetic distance for each codon position: Green = 1sts, Blue = 2nds, Orange = 3rds. Signal versus noise was graphically visualized using quartet likelihood mapping. (TIF) [file pone.0021206.s003.tif]
