# Supplementary material for: The First Molecular Phylogeny of Strepsiptera (Insecta) Reveals an Early Burst of Molecular Evolution Correlated with the Transition to Endoparasitism
Source: PLoS One. 2011 Jun 28;6(6):e21206. doi: 10.1371/journal.pone.0021206 (PMC3125182; doi:10.1371/journal.pone.0021206)
Supplement: Table S1 — Summary of Strepsiptera divergence times. Summary of divergence time estimates for the major nodes in the Strepsiptera phylogeny using the combined mitochondrial coding gene (cox1+nad1) partition. *Pairwise sequence divergences per million years. Clade abbreviations follow figure 1. †Node ages defined by exponential priors. (DOC) [file pone.0021206.s004.doc]

|  | **MIT(1+2)** |  |  |  | **MIT(1+2)(3)** | |  |  |
| --- | --- | --- | --- | --- | --- | --- | --- | --- |
|  | 95% Upper | 95% Lower | Height | Rate* | 95% Upper | 95% Lower | Height | Rate***** |
|  | **GTR+G+I** |  |  |  |  |  |  |  |
| Strepsiptera† | 106.74 | 93.50 | **97.94** | ***2.58*** | 104.43 | 93.50 | **97.20** | ***4.00*** |
| Stylopidia | 99.59 | 77.08 | **87.68** | *1.29* | 97.12 | 76.68 | **86.66** | *1.57* |
| Stylopiformia | 91.74 | 66.51 | **80.04** | 0.79 | 91.25 | 69.32 | **80.46** | 1.05 |
| S+X+E+H | 86.77 | 59.07 | **73.64** | 0.73 | 86.61 | 62.84 | **74.82** | 1.12 |
| E+H | 80.14 | 49.35 | **65.61** | 0.56 | 79.28 | 52.20 | **66.52** | 1.04 |
| C | 73.37 | 31.30 | **51.80** | 0.93 | 77.66 | 38.55 | **57.35** | 1.42 |
| My† | 53.88 | 44.00 | **47.52** | 0.49 | 53.83 | 44.00 | **47.58** | 0.59 |
| S+X | 77.11 | 44.83 | **61.86** | 0.62 | 78.23 | 49.80 | **63.73** | 1.05 |
| X | 63.23 | 29.85 | **45.92** | 1.08 | 61.54 | 34.38 | **47.95** | 1.58 |
| E | 53.07 | 17.10 | **34.56** | 1.17 | 53.27 | 22.36 | **38.50** | 1.68 |
| H | 59.66 | 30.93 | **45.60** | 1.34 | 61.54 | 34.31 | **48.41** | 1.84 |
|  | **SRD06** |  |  |  |  |  |  |  |
| Strepsiptera† | 106.77 | 93.50 | **98.04** | ***3.06*** | 104.89 | 93.50 | **97.16** | ***4.02*** |
| Stylopidia | 99.15 | 76.33 | **87.30** | *1.45* | 97.74 | 75.20 | **86.53** | *1.78* |
| Stylopiformia | 93.76 | 68.78 | **80.76** | 0.91 | 92.67 | 67.78 | **80.49** | 1.07 |
| S+X+E+H | 89.06 | 61.18 | **75.06** | 0.87 | 86.41 | 60.20 | **74.08** | 1.21 |
| E+H | 80.74 | 51.49 | **66.43** | 0.63 | 79.17 | 50.91 | **65.44** | 1.11 |
| C | 71.14 | 28.94 | **49.02** | 1.04 | 83.54 | 26.12 | **53.37** | 1.60 |
| My† | 53.63 | 44.00 | **47.46** | 0.57 | 55.01 | 44.00 | **47.81** | 0.68 |
| S+X | 79.86 | 45.66 | **63.39** | 0.66 | 79.52 | 42.65 | **63.84** | 1.32 |
| X | 60.55 | 27.38 | **44.28** | 1.03 | 63.30 | 30.60 | **47.21** | 1.68 |
| E | 52.77 | 17.64 | **32.94** | 1.22 | 53.48 | 24.24 | **37.28** | 1.86 |
| H | 60.86 | 31.29 | **45.78** | 1.48 | 34.9 | 61.49 | **49.47** | 2.22 |
