# Supplementary material for: The First Molecular Phylogeny of Strepsiptera (Insecta) Reveals an Early Burst of Molecular Evolution Correlated with the Transition to Endoparasitism
Source: PLoS One. 2011 Jun 28;6(6):e21206. doi: 10.1371/journal.pone.0021206 (PMC3125182; doi:10.1371/journal.pone.0021206)
Supplement: Table S2 — RRTest comparative analysis across strepsipteran clades. Bold = P-value with significant rate comparison (bonferroni corrected). *Marginally non-significant after bonferroni adjustment in the mitochondrial (A) and 18S rRNA partition (B). Clade abbreviations follow figure 1. (DOC) [file pone.0021206.s005.doc]

| **A) MIT1+2** |  |  |  |  |  |  |  |  |
| --- | --- | --- | --- | --- | --- | --- | --- | --- |
| Out | ~ |  |  |  |  |  |  |  |
| Mengenillidae | **1.00E-07** | ~ |  |  |  |  |  |  |
| Corioxenidae | **1.00E-07** | **1.14E-06** | ~ |  |  |  |  |  |
| Myrmecolacidae | **1.00E-07** | **2.75E-05** | 0.18898 | ~ |  |  |  |  |
| *Lychnocolax* | **1.00E-07** | **0.00413*** | 0.01725 | 0.14466 | ~ |  |  |  |
| Stylopidae+Xen’ | **1.00E-07** | **0.00035** | 0.02617 | 0.27327 | 0.65091 | ~ |  |  |
| Elenchidae | **1.00E-07** | **0.00016** | 0.21722 | 0.93458 | 0.23112 | 0.41219 | ~ |  |
| Halictophagidae | **1.00E-07** | **5.59E-05** | 0.30873 | 0.85454 | 0.14423 | 0.26956 | 0.80911 | ~ |
| **B) *18S rRNA*** |  |  |  |  |  |  |  |  |
| Out | ~ |  |  |  |  |  |  |  |
| Mengenillidae | **0.00235*** | ~ |  |  |  |  |  |  |
| Corioxenidae | **0.00048** | 0.38971 | ~ |  |  |  |  |  |
| Myrmecolacidae | **0.00019** | 0.05148 | 0.91377 | ~ |  |  |  |  |
| *Lychnocolax* | **0.00031** | 0.10149 | 0.43378 | 0.46128 | ~ |  |  |  |
| Stylopidae+Xen’ | **0.00198*** | 0.94155 | 0.29863 | 0.21961 | 0.05508 | ~ |  |  |
| Elenchidae | **8.60E-05** | 0.04120 | 0.21737 | 0.22339 | 0.66994 | 0.02638 | ~ |  |
| Halictophagidae | **0.00323*** | 0.86717 | 0.51304 | 0.38655 | 0.12582 | 0.77262 | 0.07935 | ~ |
|  | Out | Me | C | My | L | S+X | E | H |
