# Supplementary material for: The First Molecular Phylogeny of Strepsiptera (Insecta) Reveals an Early Burst of Molecular Evolution Correlated with the Transition to Endoparasitism
Source: PLoS One. 2011 Jun 28;6(6):e21206. doi: 10.1371/journal.pone.0021206 (PMC3125182; doi:10.1371/journal.pone.0021206)
Supplement: Table S3 — List of characters and corresponding states recovered in the reconstruction of strepsipteran morphological traits. The position in the phylogeny of significant character reconstructions appears in brackets next to the corresponding state, followed by the BF range supporting that reconstruction. Some characters may be considered dependent, if single genotypic events can be demonstrated to produce pleiotropic effects. Possible examples include male/female larval spiracles, and male/female larval legs. * equivocal BFs (0.2–3.8). This might disguise a potentially apomorphic loss of tarsomeres in the Elenchidae+Halictophagidae ancestor. (DOC) [file pone.0021206.s006.doc]

| **Character** | **State and point of reconstruction on tree** |
| --- | --- |
| **Male** |
| Ommatidia | Contiguous; separated by spaces (microtrichia) **(Strepsiptera 8.3)** |
| Antennomeres | 8 or more; 7 or less **(Strepsiptera 6.1)** |
| Vein CuA1 + CuA2 | Both; CuA1 absent **(Elenchidae 10.4) (Myrmecolacidae 4.6)**. |
| Vein MA1 + MA2 | Both; MA1 absent **(Stylopidia 5.3-7.9)**; both absent **(Elenchidae 12.4-14.2)**. |
| Labrum | Present; absent **(Strepsiptera 8.1)** |
| Mandibles | Present; cone shaped; absent **(Halictophagidae 10.1-13.2)**. |
| Galea (mouthpart) | Present; absent **(Strepsiptera 7.7)** |
| Lacinia (mouthpart) | Present; absent **(Strepsiptera 7.1)** |
| Palpus maxillaris (mouthpart) | Several palpomeres; single palpomere **(Strepsiptera 6.9)** |
| Labial palps (mouthparts) | Present; absent **(Strepsiptera 6.9)** |
| Mesothoracic halteres | Absent; present **(Strepsiptera 8.0)** |
| Trochanter and femur of foreleg | Separated; fused **(Strepsiptera 5.1)** |
| Trochanter and femur of middle leg | Separated; fused **(Strepsiptera 7.9)** |
| Number of tarsomeres* | 5; 4 **(Stylopiformia 8.1-15.6)**; 3 **(Halictophagidae 9.6-11.5)**; 2 **(Elenchidae 13.4-18.2)**. |
| Claws | Paired and normally developed; strongly reduced; absent **(Stylopiformia 5.6-6.7)**. |
| Accessory claws foreleg basitarsus | Absent; present **(Elenchidae 13.5)**. |
| Accessory claws mid- hindleg basitarsus | Absent; present **(Halictophagidae 9.5)**. |
| Setulose pads (all tarsomeres) | Absent; present **(Stylopiformia 4.6)**. |
| Metacoxa | Free; immobile - integrated into metathorax **(Strepsiptera 8.5)** |
| Abdominal spiracles | Segments I–VIII; segments I–VII; segment I **(Stylopidia 4.3-5.2)**. |
| Larval legs | Well developed; reduced but distinct; absent **(Stylopiformia 7.1-8.1)**. |
| Pupal wing buds | Present; absent **(Stylopidia 7.2)**. |
| Pupal claws | Filiform; absent **(Stylopidia 4.7)**. |
| Larval spiracles | Segments I–VII; absent **(Stylopidia 5.1)**. |
| **Female** |  |
| Extensive sexual dimorphism | Absent; present (females wingless) **(Strepsiptera 8.2)** |
| Tentorium | Present; absent **(Strepsiptera 7.7)** |
| Compound eyes | Present; absent **(Stylopidia 4.8)** |
| Antennae | Present with several antennomeres; absent **(Stylopidia 5.0)** |
| Wings | Present; absent **(Strepsiptera 8.7)** |
| Legs | Present; absent **(Stylopidia 4.7)** |
| Ovarioles | Present; absent **(Strepsiptera 7.0)** |
| Ovipositor | Present; absent **(Strepsiptera 6.1)** |
| Digestive tract | Continuous; interrupted **(Strepsiptera 7.7)** |
| Abdominal spiracles | Segments I–VIII; segments I–VII; segment I **(Stylopidia 4.6-4.9)** |
| Pupal head | Separate prothorax; partially fused; integrated prothorax **(Stylopiformia 8.0-8.5)** |
| Pupal labrum (mouthpart) | Present; absent **(Stylopidia 5.7)** |
| Pupal corpus maxillary | Developed; vestigial **(Stylopiformia 6.6)** |
| Pupal palpus maxillary (palpomeres) | Several; single palpomere **(Strepsiptera 3.7-3.9)**; vestigial **(Stylopiformia 3.2-4.7)** |
| Pupal brood canal external | Absent; present **(Stylopidia 4.6)**. |
| Pupal Cuticular thorns | Present; absent **(Stylopidia 5.0)**. |
