# Supplementary material for: The First Molecular Phylogeny of Strepsiptera (Insecta) Reveals an Early Burst of Molecular Evolution Correlated with the Transition to Endoparasitism
Source: PLoS One. 2011 Jun 28;6(6):e21206. doi: 10.1371/journal.pone.0021206 (PMC3125182; doi:10.1371/journal.pone.0021206)
Supplement: Table S4 — Specimen, primer information and rRNA template alignments. Genbank accession and specimen source information; list of primers used in this study (*Primers designed for short-fragment PCR) and 18 rRNA and 16S rRNA template secondary structural alignments. (DOC) [file pone.0021206.s007.doc]

| **TAXON** | **LOCATION** | ***cox1*** | ***nad1*** | ***16S rRNA*** | ***18S rRNA*** |
| --- | --- | --- | --- | --- | --- |
| **Outgroup** |  |  |  |  |  |
| *Periplaneta fuliginosa* | - | AB126004:  ID:2914104 | AB126004:  ID:2914108 | AB126004:  12617-13916 | DQ874171 |
| *Tamolanica tamolana* | - | DQ241797:  ID:3854635 | DQ241797:  ID:3854634 | DQ241797:  12921-14238 | AY491171 |
| *Reticulitermes flavipes* | - | EF206314:  ID:5183224 | EF206314:  ID:5183213 | EF206314:  12681-13993 | EF363230 |
| *Antheraea pernyi* | - | AY242996:  ID:806814 | AY242996:  ID:806825 | AY242996:  12804-14172 | DQ347469 |
| *Bombyx mori* | - | AB070263:  ID:804651 | AB070263:  ID:804653 | AB070263:  7649-9025 | DQ347470 |
| *Haematobia irritans* | - | DQ029097:  ID:3416083 | DQ029097:  ID:3416095 | DQ029097:  12641-13961 | EU179518 |
| *Ceratitis capitata* | - | AJ242872:  ID:808516 | AJ242872:  ID:808526 | AJ242872:  12782-14116 | AF096450 |
| *Tribolium castaneum* | - | AJ312413:  ID:803829 | AJ312413:  ID:803824 | AJ312413:  12518-13797 | HM156711 |
| *Rhagophthalmus ohbai* | - | AB267275  ID:6407553 | AB267275  ID:6407556 | AB267275  12282-13558 | AB298864 |
| **Mengenillidae** |  |  |  |  |  |
| *Mengenilla australiensis* Kifune & Hirashima ♂ | AUSTRALIA, Queensland, Blackdown Tablelands NP, South Mimosa Creek, S23º47.687’, E 149º 04.195’, 16.iii.2006, light trap, (N. Jönsson, T. Malm, & D. Williams | GU188852 | GU188852 | GU188852 | JN082886 |
| *Mengenilla chobauti* Hofeneder ♂ | SPAIN: Jumilla, Murcia, N37º59N, W1º8’, 09.x.2003, light trap (J.A. Delgado & F. Collantes) | JN082786 | JN082825 | JN082858 | JN082887 |
| **Corioxenidae** |  |  |  |  |  |
| *Corioxenos acucyrtophallus* Cook ♂ | MEXICO: Tapachula, El Colegio de la Frontera Sur, N14º53, W92º17', Al.127m, black light, 05.vii.2006 (J. Barrera, H. Gomez) | JN082791 | JN082830 | JN082862 | JN082893 |
| *Triozocera* sp. [1] ♂ | PAPUA NEW GUINEA: Dami Research Station, West New Britain, S5º 32' 07.08", E150º 19'57.71", light trap, x.2000 (S. Makai) | JN082787 | JN082826 |  | JN082888 |
| *Triozocera* sp. [2] ♂ | BRAZIL: EMPARN-Jiqui - city of Parnamirim, state of Rio Grande do Norte, S5º55'36", W35º11'33", Malaise trap, 17-25.vi.2005 (R. Andreazze, H Andrade, M. Pinheiro) | JN082788 | JN082827 | JN082859 | JN082889 |
| *Triozocera* sp. [3] ♂ | USA, South Carolina, Charleston County, Mt Pleasant, N32º49'38.28", W79º49'03.216", uv light, 31.vii.2006 (R. Lareau) | JN082789 | JN082828 | JN082860 | JN082890 |
| *Triozocera* sp. [4] ♂ | MEXICO: Tapachula, El Colegio de la Frontera Sur, N14º53, W92º17', Al.127m, black light, 17.v.2007 (J. Barrera, H. Gomez) | JN082790 | JN082829 | JN082861 | JN082891 |
| *Blisseoxenos esaki* ♀ | JAPAN: Kirarazaka Shuzakuin-Rikyu Otowagawa Shugakuin Sako-ku, Kyoto, 35˚3’8’’N, 135˚48’24’’E (Y. Nakase) |  |  |  | JN082892 |
| **Myrmecolacidae** |  |  |  |  |  |
| *Stichotrema dallatorreanum* Hofeneder ♀ | PAPUA NEW GUINEA: Dami Research Station, West New Britain, S32’ 07.08”, E150 º 19’ 57.71”, hand collected, 12.x.2000 (S. Makai) | JN082794 | JN082833 |  | JN082896 |
| *Myrmecolax incautus* Kogan & Oliveira ♂ | FRENCH GUYANA: Camp Patawa, N4º 32’40.5”, W52º 09’ 08.4”, 14.iii.2007; Forêt Kerrenroch, Petit Saut, N5^o^ 04’09.7”, 18.iii.2007 (R. S. Ferreira, D. Fresneau, P. Devienne) | JN082796 | JN082835 | JN082863 | JN082898 |
| *Myrmecolax* sp. [4] ♂ | BRAZIL: Para Belo Monte, Rio Xingu, Rodovia Transamazóica, ,S03º05 52”, W51º41’31”, light trap, 07.iv.2008 (J. A. Jarafael, F. F. Xeavier F) | JN082800 | JN082836 | JN082866 | JN082902 |
| *Stichotrema* sp. ♂ | THAILAND: Sakon Nakhon Phu PhanNP, dry Dip forest, N17º09.921’, E103º54.485’, Malaise trap, 25-31.x.2006 (S. Tongboonchai) | JN082795 | JN082834 |  | JN082897 |
| *Myrmecolax* sp. [5] ♂ | MEXICO: Estacion de Biologia Tropical ‘Los Tuxtlas’, Instituto de Biologia UNAM, 30km Carretera Catamaco, Montepio Apdo, San Andrés, Tuxtla, Veracruz, black light, N18º35’, W95º5’, 17.v.2007 (J. Kathirithamby, J. Barrera, H. Gomez, D. P. McMahon | JN082801 | JN082837 | JN082867 | JN082903 |
| *Myrmecolax* sp. [3] ♂ | BRAZIL: Paraiso Chapada dos Veadeiros Rio San Minguel, S14º11’34”, light trap, 26.iii.2008 (J. A. Jarafael, Rafael, F. F. Xeavier F.) | JN082799 |  |  | JN082901 |
| *Myrmecolax* sp. [1] ♂ | BRAZIL: Paraiso Chapada dos Veadeiros Rio San Minguel, S14º11’34”, 26.iii.2008 (J. A. Rafael, F. F. Xeavier F.) | JN082797 |  | JN082864 | JN082899 |
| *Myrmecolax* sp. [2] ♂ | BRAZIL: EMPARN-Jiqui – city of Parnamirim, state of Rio Grande do Norte, S5º55’36”, W35º11’33”, Malaise trap, 17-25.vi.2004 (R. Andreazze, H Andrade, M. Pinheiro) | JN082798 |  | JN082865 | JN082900 |
| *Caenocholax* sp. [1] ♂ | PANAMA: Barro Colorado Island, N9°9.046’, W79°50.793’, elevation 143 m. Malaise trap, 04-07.iv.2007 (D. Windsor) | JN082802 | JN082838 | JN082868 | JN082904 |
| *Caenocholax* sp. [3] ♂ | ARGENTINA: Corrientes, Camaronera, S26º31’ , W58 17’, 19. xii.2003 (L. Calcaterra & L. Nuñez) | JN082803 | JN082839 | JN082869 | JN082905 |
| *Caenocholax* sp. [10] ♂ | USA: College Station, Brazon Co., Lick Creek Park, Texas, 30.5649N, 96.2113W, Malaise trap, x.2005 (K. Zhaurova) | JN082804 | JN082840 |  | JN082906 |
| **Lychnocolax** |  |  |  |  |  |
| *Lychnocolax* sp. [2] ♂ | THAILAND: Nakhon Nayok Khao Yai MP San Jao Khaokeaw, 14º22’ 101º23.253’E, 5-12.iii.2007 (P. Sandao) | JN082793 | JN082832 | JN082870 | JN082895 |
| *Lychnocolax* sp. [1] ♂ | THAILAND: oetKraduna sava Mah in pine forest, 16 º10’ 02” 101º 4’ 7”, pan traps, 2-3.i.2007, (Tgano igask Salsa) | JN082792 | JN082831 |  | JN082894 |
| **Stylopidae** |  |  |  |  |  |
| *Stylops melittae* Kirby ♂ | SWEDEN: Upplands Bro. Lindormsnäs grustag, N59° 29′ 43.49″, E17° 34′ 04.94″, Håvad flygande (caught in a net in flight) 29.iii/2007 (B. Viklund) (loan from Naturhistoriska Riksmuseet, S Stockholm Loan no 687/07) | JN082812 | JN082846 | JN082878 | JN082913 |
| **Xenidae** |  |  |  |  |  |
| *Xenos moutoni* De Buysson ♂ | JAPAN: Tsukuba, Ibaraki, 36 00’N 140 07’E, from nest, 15.ix.2006 (S. Makino) | JN082805 | JN082841 | JN082872 | JN082907 |
| *Xenos vesparum* Rossi ♂ | ITALY: Florence, hand collected, 09.2000 (D. Hughes) | JN082806 | JN082842 | JN082873 | JN082908 |
| *Xenos pecki* Kirby ♂ | USA: Vermilion River Observatory, Dannville, Vermilion County ,Illinois, N40° 03’ 36”, W87° 33’52” (NAD83/WGS84),Malaise trap, vi.2007 (A. Toth) | JN082808 | JN082843 | JN082874 | JN082909 |
| *Xenos hamiltoni* Kathirithamby & Hughes ♂ | MEXICO: Estacion de Biologia Tropical ‘Los Tuxtlas’, Instituto de Biologia UNAM, 30km Carretera Catamaco, Montepio Apdo, San Andrés, Tuxtla, N18º35’, W95º5’, Veracruz, from nest, 08.v.2002 (D. Hughes) | JN082807 |  | JN082871 |  |
| *Xenos* sp. ♂ | SOUTH AFRICA: Murrayfield, Pretoria.N25° 44’ 60.00”, E28° 17’ 52.80”, hand collected, 7.iv.2008 (C, Eardley) | JN082809 |  | JN082875 | JN082910 |
| *Paraxenos* sp. ♀ | USA: Sioux city, Iowa, N42°32.5’, W90° 23.48’, hand collected, 30.viii.2008 (G. K. Lechner) | JN082810 | JN082844 | JN082876 | JN082911 |
| *Pseudoxenos* sp. ♂ | PORTUGAL: Almograve, Alentejo, N37° 39’ 7.20”, W8° 48’ 3.60”, hand collected, 25.ix.2008 (A. Hayward, D. McMahon) | JN082811 | JN082845 | JN082877 | JN082912 |
| **Halictophagidae** |  |  |  |  |  |
| *Halictophagus silwoodensis* Waloffi ♀ | BELGIUM: Nationaal Park Hoge Kempen, Mecelse Heide, Maasmechelen, Belgium, N50°58’26.29”, E5°39’23.38”, ♂: yellow pan traps, ♀: glass insect traps attracted males, 8-24.v.2007 (H. Henderickx) | JN082818 | JN082852 | JN082882 | JN082916 |
| *Halictophagus calcaratus* Pasteels ♂ | SOUTH AFRICA: Clanwilliam, Ysterfontein farm, Province Western Cape, S32° 9’ 57.8” E18° 46’41.0”, sweep netting, 25.xi.2008 (J. Hatting) | JN082814 | JN082848 | JN082880 | JN082915 |
| *Tridactylophagus* sp. ♂ | PAPUA NEW GUINEA: Dami Research Station, West New Britain, S32’ 07.08”, E150 º 19’ 57.71”, light trap, ix.2000 (S. Makai) | JN082813 | JN082847 | JN082879 | JN082914 |
| *Callipharixenos* sp. ♀ | PHILLIPINES: Carabao, Pico & Katchamitha, N10º40’, E122º57’, sweep netting, 1994-98 (V. R. Ocampo, G. M. Anober, R. S. Rejesus) | JN082819 | JN082853 |  | JN082919 |
| *Halictophagus* sp. [1] ♀ | PHILLIPINES: Carabao, Pico & Katchamitha, sweep netting, 1994-98 (V. R. Ocampo, G. M. Anober, R. S. Rejesus) | JN082815 | JN082849 |  |  |
| *Halictophagus*  sp. [3] ♂ | MEXICO: Tapachula, El Colegio de la Frontera Sur, N14º53’, W92º17’, Al.127m, Black light,16.xii.2006-23.01.07 (J. Barrera, H. Gomez) | JN082817 | JN082851 | JN082881 | JN082918 |
| *Halictophagus* sp. [2] ♂ | ROMANIA: Fundulea, N44° 28’ 22.80”, E26° 31’ 1.20”, yellow pan trap, 1.vii.2008 (Z. Acs) | JN082816 | JN082850 | JN082883 | JN082917 |
| **Elenchidae** |  |  |  |  |  |
| *Elenchus tenuicornis* Kirby♂ | EUROPE: Struman Kommun, Vasterbottens Lan, (Ly Laxrias RN), N65º45’0”, E15º15’0”, light trap, 11-12.vii.1992 (J. A. Jenzen) | JN082820 |  |  |  |
| *Elenchus varleyi* Kathirithamby) ♂ | AUSTRALIA: Barrow Island, Western Australia, N 20º 49’ 35’’ E 115 º 26’ 39’’, suction, 06.v.2006 (S. Callan, R. Graham) | JN082821 | JN082854 |  | JN082921 |
| *Elenchus* sp. [1] ♂ | MEXICO: Tapachula, El Colegio de la Frontera Sur, N14º53, W92º17’, Al.127m, light trap, 15.xii.2006,15.v-16.vi.2007 (J. Barrera, H. Gomez) | JN082822 | JN082855 |  | JN082920 |
| *Elenchus* sp. [2] ♂ | USA: Charleston County, Mt Pleasant, S. Carolina (tube C), N32º49’38.28”, W79º49’03.216”, UV, 10-15.viii.2006 (R. Lareau) | JN082823 | JN082856 | JN082885 |  |
| *Elenchus koebelei* Pierce ♂ | USA: St Mark’s National Wildlife Refuge, Florida, Goose Creek, N30.10º 621’, W84.26º 238’, sweep net, 30.vi.2005 (J. Kathirithamby, S. Taylor) | JN082824 | JN082857 | JN082884 | JN082922 |

| **LOCUS** | **NAME** | **SEQUENCE** | **REFERENCE** |
| --- | --- | --- | --- |
| *Cox1* | LCO_DEG (F) | TWTCWACHAAYCATAARGATATTGG | [1] |
| *Cox1* | JERRY_DEG (F) | CAACAYTTATTYTGATTYTTYGG | [2] |
| *Cox1* | WALLY_DEG (F)* | GAGYTCAYCAYATATTTAC | This study |
| *Cox1* | PETER_DEG (F)* | GGAWTTACWGGWATTATYTTAGC | This study |
| *Cox1* | ALEX_DEG (R)* | TCAATTTCCAAAYCCYCCYAT | [3] |
| *Cox1* | NANCY_DEG (R) | CCDGGTAAAATTAAAATATAAACTTC | [2] |
| *Cox1* | HANNAH_DEG (R)* | CCWGTRGGAAYWCCAATAAT | This study |
| *Cox1* | MARIA_DEG (R)* | GCWACWACATAATAWGTATCATG | This study |
| *Cox1* | COI_1265 (R) | AARTGTTGRGGRAARAADGTTA | This study |
| *Cox1* | GEORGE_DEG (R) | TCTGARTAYCGTCGYGGTAT | This study |
| *16S rRNA* | 12S-791 (F) | TATGYACAYATYGCCCGTC | [3] |
| *16S rRNA* | 16S-857 (F) | GACYGTRCDAAGGTAGCATAAT | [3] |
| *16S rRNA* | 16S-994 (R) | GACGAKAAGACCCYATARAD | [3] |
| *16S rRNA* | 16S-1214 (R) | GCGACCTCGATGTTGGATTW | [3] |
| *16S rRNA/nad1* | 16S_FOR1 (F) | CATGATCTGAGTTNAAAYCGG | [3] |
| *16S rRNA/nad1* | 16S_FOR2 (F) | GAGTTNAAAYCGGTGTGAGCC | [3] |
| *Nad1* | ND1-143-F | GGTTATATTCADATTCGTAARRG | [3] |
| *Nad1* | ND1-175-R | TTWATWCCATCHCYWAAAGGTTG | [3] |
| *Nad1* | ND1-646-R | CWGAAACTAAYTCWGATTCHCC | [3] |
| *Nad1* | ND1-847-R | TTTATCATAHCGAWAWCGAGG | [3] |
| *18S rRNA* | 18S_23 (F) | GGATCCTGGCAGTAGTTATATG | [4] |
| *18S rRNA* | 18S_560 (F) | GGCGTRTCTTTCAATTGTCTGC | This study |
| *18S rRNA* | 18S_849 (F) | CGGTAATTCCAGCTCCATTAG | [4] |
| *18S rRNA* | 18S_615 (R) | GTAGRCATGTAAYCTACCATCG | [4] |
| *18S rRNA* | 18S_905 (R) | CTACGAGCTTTTTAAMCGCAAC | This study |
| *18S rRNA* | 18S_1547 (R) | GTACGAATGCCCCCATCCG | [4] |

1. Folmer O, Black M, Hoeh W, Lutz R, Vrijenhoek R (1994) DNA primers for amplification of mitochondrial cytochrome c oxidase subunit I from diverse metazoan invertebrates. Mol Mar Biol Biotech 3: 294-299.
2. Simon C, Frati F, Beckenbach A, Crespi B, Liu H, Flook P (1994) Evolution, weighting, and phylogenetic utility of mitochondrial gene sequences and compilation of conserved polymerase chain reaction primer. Ann Entomol Soc Am 87: 651-690.
3. McMahon DP, Hayward A, Kathirithamby J (2009) The mitochondrial genome of the ‘twisted-wing parasite’ *Mengenilla australiensis*: a comparative study. BMC Genomics **10:** 603.
4. Kathirithamby J, Hayward A, McMahon DP, Ferreira RS, Andreazze R, Tadeu de Almeida Andrade H, Fresneau D (2010) Conspecifics of a heterotrophic heteronomous species of Strepsiptera (Insecta) are matched by molecular characterization. Syst Entomol 35: 234-242.

**18S rRNA template alignment**

**Outgroup taxa in blue**

**H61 RAA1 RAA1’ H61' H113 H122 H143 RAA2 RAA2’ H143' H144 H144' ((....((....((((..( ............ )..))))...)).)) ...... ((( ...... (((((((...((....(((((( ....((((.....( ............ ).....)))). (((.((...((((((( ...... )))))))....)))))**

**Periplaneta fulig' ||GUCUCAGUACGAGCC----| [----------------] [GCAUUAA-----] [---------------] |----GGUGAAACCGC|| AAAAGG |CUC| AUUAAA ||UCAGUUAUGGUUCCUUAGAUCG| ||--------------| [-----------------] [UACAAUCCCUUA] [----------------] |-----------|| ||CUU-GGAUAACUGUGG| UA-AUU |CUAGAGCUAAUACAUG||**

**Tamolanica tamolana ||GUCUCAGUGCAAGCC----| [----------------] [GCAUUAA-----] [---------------] |----GGUGAAACCGC|| GAAUGG |CUC| AUUAAA ||UCAGUUAUGGUUCCUUAGAUGG| ||--------------| [-----------------] [UGGACAGUUA--] [----------------] |-----------|| ||CUU-GGAUAACUGUGG| UA-AUU |CUAGAGCUAAUACAUG||**

**Reticulitermes fla’ ||????????GCAAGCC----| [----------------] [GAAUUAA-----] [---------------] |----GGUGAAACCGC|| GAAUGG |CUC| AUUAAA ||UCAGUUAUGGUUCCUUAGAUGA| ||--------------| [-----------------] [UGCACAGUUA--] [----------------] |-----------|| ||CUU-GGAUAACUGUGG| UA-AUU |CUAGAGCUAAUACAUG||**

**Antheraea pernyi ||GUCUCAGUGCAAGCC----| [----------------] [GUAUUAA-----] |---------------] |----GGCGAUACCGC|| GAAUGG |CUC| AAUAUA ||UCAGUUUUGGUUCCUUAGAUCU| ||--------------| [-----------------] [UACUCAGUUA--] [----------------] |-----------|| ||CUU-GGAUAACUGUGG| UA-AUU |CUAGAGCUAAUACAUG||**

**Bombyx mori ||GUCUCAGUGCAAGCC----| [----------------] [GUAUUAA-----] [---------------] |----GGCGAUACCGC|| GAAUGG |CUC| AAUAUA ||UCAGUUUUGGUUCCUUAGAUCU| ||--------------| [-----------------] [UACUCAGUUA--] [----------------] |-----------|| ||CUU-GGAUAACUGUGG| UA-AUU |CUAGAGCUAAUACAUG||**

**Haematobia irritans ||GUCUAAGUACACACG----| [----------------] [AAUUAAC-----] [---------------] |----AGUGAAACCGC|| AAAAGG |CUC| AUUAUA ||UCAGUUAUGGUUCCUUAGAUCG| ||--------------| [-----------------] [UUAACAGUUA--] [----------------] |-----------|| ||CUU-GGAUAACUGUGG| UA-AUU |CUAGAGCUAAUACAUG||**

**Ceratitis capitata ||GUCUAAGUACAAACA----| [----------------] [AAUUAAA-----] [---------------] |----AGUGAAACCGC|| AAAAGG |CUC| AUUAUA ||UCAGUUAUGGUUCCAUAGAUCG| ||--------------| [-----------------] [UUAACAGUUA--] [----------------] |-----------|| ||CUU-GGAUAACUGUGG| UA-AUU |CUAGAGCUAAUACAUG||**

**Tribolium castaneum ||GUCUCAGUACAAGCC----| [----------------] [AAAUUAA-----] [---------------] |----GGUGAAACCGC|| GAAAGG |CUC| AUUAAA ||UCAGUUAUGGUUCCUUAGAUCG| ||--------------| [-----------------] [UACCCACAUUUA] [----------------] |-----------|| ||CUU-GGAUAACUGUGG| UA-AUU |CUAGAGCUAAUACAUG||**

**Rhagophthalmus ohb' ||???????????????????| [????????????????] [????UAA-----] [---------------] |----GGUGAAACCGC|| GAAAGG |CUC| AUUAAA ||UCAGUUAUGGUUCCUUAGAUCG| ||--------------| [-----------------] [UACCCACAUUUA] [----------------] |-----------|| ||CUU-GGAUAACUGUGG| UA-AUU |CUAGAGCUAAUACAUG||**

**Mengenilla australiensis ||GUCUAAGUACUAGCCGACC| [----------------] [AUUUU-------] [---------------] |AAAUGGUGAAACCGC|| GAAAAG |CUC| AUUAAA ||UCGGCUUUGGUUUAUUAGACAG| ||UUUACAAAUUGA-G| [UGAUUUUUCGC------] [UUUU--------] [-----GGCGUUUUAUC] |CGAA-AUUGUU|| ||GAU-GGAUAACUGUGG| UACAUU |CUAGAGCUAAUACAUG||**

**Mengenilla chobauti ||???????????????????| [????????????????] [????????????] [???????????????] |???????????????|| ?????? |???| ?????? ||??????????????????????| ||??????????????| [?????????????????] [????????????] [????????????????] |???????????|| ||????????????????| ?????? |????????????????||**

**Triozocera sp.(2) ||GUGUAAGUACUAGCCAACU| [----------------] [AAUA--------] [---------------] |AAAUGGUGAAACCGC|| GAAAAG |CUC| AUUAAA ||UCGGCUUUGGUUUAUUAGAUAG| ||UAUGUAGAUUGAAG| [UUUUUCGUUGC------] [CUUAUGU-----] [----GCACACGAAAAG] |UGAAUAUCUAA|| ||AAU-GGAUAACUGUGG| UA-AUU |CUAGAGCUAAUACAUG||**

**Triozocera sp.(3) ||GUGUAAGUACUAGCCAACU| [----------------] [AAUA--------] [---------------] |AAAUGGUGAAACCGC|| GAAAAG |CUC| AUUAAA ||UCGGCUUUGGUUUAUUAGAUAG| ||UAUGUAGAUUGAAG| [UUUUUCGUUGC------] [UCAUAU------] [----GCACACGAAAAG] |UGAAUAUCUAA|| ||AAU-GGAUAACUGUGG| UA-AUU |CUAGAGCUAAUACAUG||**

**Triozocera sp.(1) ||GUGUAAGUACUAGCCAACU| [----------------] [AAUA--------] [---------------] |AAAUGGUGAAACCGC|| GAAAAG |CUC| AUUAAA ||UCGGCUUUGGUUUAUUAGAUAG| ||UAUGUAGAUUGAAA| [UUUUUUGCUGCGCA---] [AGAU--------] [---UGCGCACAAAAAG] |UGAAUAUCUAA|| ||AAU-GGAUAACUGUGG| UA-AUU |CUAGAGCUAAUACAUG||**

**Triozocera sp.(4) ||GUGUAAGUACUAGCCAACU| [----------------] [AAUA--------] [---------------] |AAAUGGUGAAACCGC|| GAAAAG |CUC| AUUAAA ||UCGGCUUUGGUUUAUUAGAUAG| ||UAUGUAGAUUGAAG| [UUUUUCGUUGC------] [UUAUAU------] [----GCACACGAAAAG] |UGAAUAUCUAA|| ||AAU-GGAUAACUGUGG| UA-AUU |CUAGAGCUAAUACAUG||**

**Blisseoxenos esaki ||GUGUUAGUACUUGCCAACU| [----------------] [AAUA--------] [---------------] |AAAUGGUGAAACCGC|| GAAAGG |CUC| AUUAAA ||UCGGCUUUGGUUCAUUGGAUAG| ||UGCGCAGAUUUGAA| [UUUUUCGUCGAGAGCAA] [AAU---------] [-UUGCUCACGUGAAAG] |AUGCAAUCUAC|| ||AAU-GGAUAACUGUGG| UA-AUC |CUAGAGCUAAUACAUG||**

**Corioxenos acucyrtophallus ||GUGUAAGUACGAGCCAACU| [----------------] [AAUA--------] [---------------] |AAAUGGUGAAACCGC|| GAAAAG |CUC| AUUAAA ||UCGGCUUUGGUUCCUUUGAUAA| ||UGCGCAUAUG----| [ACAUUCGUUCAGUCCAC] [CUA---------] [-GUGAACUGACGAAGA] |----UCAAUAU|| ||UAU-GGAUAACUGUGG| UA-AUU |CUAGAGCUAAUACAUG||**

**Lychnocolax sp.(2) ||???????????????????| [????????????????] [????????????] [???????????????] |???????????????|| ?????? |???| ?????? ||??????????????????????| ||UAAGCAGAUGGGAA| [AUUUUUUAAAUU-----] [UUCU-----???] [----AAUUUAAAAAAU] |GGAAAGUCUAA|| ||AAU-GGAUACCUGUAG| UA-AUU |CUAGAGCUAAUACAUG||**

**Lychnocolax sp.(1) ||???????????????????| [????????????????] [????????????] [???????????????] |???????????????|| ?????? |???| ?????? ||??????????????????????| ||??????????????| [?????????????????] [????????????] [????????????????] |???????????|| ||????????????????| ?????? |????????????????||**

**Stichotrema dallatoreanum ||GUGUAAGUACUAGCCA-UU| [----------------] [-AUA--------] [---------------] |AAAUGGUGAAACCGC|| GAAAAG |CUC| AUUAAA ||UCGGCUUUGGUUCCUUAGACAG| ||UAUGCAGAUAGGAA| [UUUUUU-C---------] [GCGA--------] [---------GAAAAAA] |UGAAAGUCUGA|| ||UUU-GGAUAACUGUGG| UA-AUU |CUAGAGCUAAUACAUG||**

**Stichotrema sp. ||GUGUAAGUACUAGCCAACU| [----------------] [-AUA--------] [---------------] |AAAUGGUGAAACCGC|| GAAAAG |CUC| AUUAAA ||UCGGCUUUGGUUCCUUAGACUG| ||UUUGCAGAUGAUAG| [UUUCUUCC---------] [GCGA--------] [--------GGGAGAAG] |UGAAAGUCUGA|| ||UUU-GGAUAACUGUGG| UA-AUU |CUAGAGCUAAUACAUG||**

**Myrmecolax sp.(4) ||GUGUAAGUACUAGCCAAAU| [----------------] [-AUA--------] [---------------] |AAAUGGUGAAACCGC|| GAAAAG |CUC| AUUAAA ||UCGGCUUUGGUUCCUUAGACAG| ||UAUGCAGAUGAGAG| [CUCUUUUU---------] [GAAA--------] [--------GAGAAGAG] |CAAAAGUCUGA|| ||UUU-GGAUAACUGUGG| UA-AUU |CUAGAGCUAAUACAUG||**

**Myrmecolax sp.(3) ||GUGUAAGUACUAGCCAAAU| [----------------] [-AUA--------] [---------------] |AAAUGGUGAAACCGC|| GAAAAG |CUC| AUUAAA ||UCGGCUUUGGUUCCUUAGACAG| ||UAUGCAGAUGGGAG| [CUCUUUUU---------] [GAAA--------] [--------GAGAAGAG] |CAAAAGUCUGA|| ||UUU-GGAUAACUGUGG| UA-AUU |CUAGAGCUAAUACAUG||**

**Myrmecolax sp.(1) ||GUGUAAGUACUAGCCAAUU| [----------------] [-AUA--------] [---------------] |AAAUGGUGAAACCGC|| GAAAAG |CUC| AUUAAA ||UCGGCUUUGGUUCCUUAGACAG| ||UAUGCAGAUGGGAG| [CUCUUUUU---------] [GAAA--------] [--------GAGAAGAG] |CAAAAGUCUGA|| ||UUU-GGAUAACUGUGG| UA-AUU |CUAGAGCUAAUACAUG||**

**Myrmecolax sp.(2) ||GUGUAAGUACUAGCCAAAU| [----------------] [-AUA--------] [---------------] |AAAUGGUGAAACCGC|| GAAAAG |CUC| AUUAAA ||UCGGCUUUGGUUCCUUAGACAG| ||UAUGCAGAUGGGAG| [CUCUUUUU---------] [GAAA--------] [--------GAGAAGAG] |CAAAAGUCUGA|| ||UUU-GGAUAACUGUGG| UA-AUU |CUAGAGCUAAUACAUG||**

**Myrmecholax incautus ||GUGUAAGUACUAGCCAACU| [----------------] [-AUA--------] [---------------] |AAAUGGUGAAACCGC|| GAAAAG |CUC| AUUAAA ||UCGGCUUUGGUUCCUUAGACAG| ||UAUGCAGAUGGGAG| [UUUUUUUC---------] [UUAG--------] [--------GAAGAAAA] |CAAAAGUCUGA|| ||UUU-GGAUAACUGUGG| UA-AUU |CUAGAGCUAAUACAUG||**

**Myrmecholax sp.(5) ||GUGUAAGUACAAGCCAAAU| [----------------] [-AUA--------] [---------------] |AAAUGGUGAAACCGC|| GAAAAG |CUC| AUUAAA ||UCGGCUUUGGUUCCUUAGACAG| ||UAUGCAGAUAGGAG| [CUCUUUUU---------] [GAAA--------] [--------GAGAAGAG] |CAAAAGUCUGA|| ||UUU-GGAUAACUGUGG| UA-AUU |CUAGAGCUAAUACAUG||**

**Caenocholax sp.(1) ||GUCUAAGUACUAGCCAACU| [----------------] [-UAAA-------] [---------------] |GAAUGGUGAAACCGC|| GAAAAG |CUC| AUUAAA ||UCGGCUUUGGUUCCUUAGAUAG| ||UUUGCAGAUUGGAG| [UUAUUUUCGGAGCGC--] [UGUU--------] [-GCGUUUUGAAAAGAA] |CAAAAUUCUGA|| ||AAU-GGAUAACUGUGG| UA-AUU |CUAGAGCUAAUACAUG||**

**Caenocholax sp.(3) ||GUCUAAGUACUAGCCAACU| [----------------] [UUUA--------] [---------------] |AAAUGGUGAAACCGC|| GAAAAG |CUC| AUUAAA ||UCGGCUUUGGUUCCUUAGAUAG| ||UUUGCAGACUGGAG| [UUUCUUUCGA-------] [ACUA--------] [------UCGAAAGAAA] |CAAAAUUCUGA|| ||AAU-GGAUAACUGUGG| UA-AUU |CUAGAGCUAAUACAUG||**

**Caenocholax sp.(10) ||GUCUAAGUACUAGCCAACU| [----------------] [UUUA--------] [---------------] |AAAUGGUGAAACCGC|| GAAAAG |CUC| AUUAAA ||UCGGCUUUGGUUCCUUAGAUAG| ||UUUGCAGAUUGGAG| [UUUUUUUCGA-------] [CCUG--------] [------UCGAAAAAAA] |CAAAAUUCUGA|| ||AAU-GGAUAACUGUGG| UA-AUU |CUAGAGCUAAUACAUG||**

**Xenos moutoni ||?????????????CCAACU| [AAAUUUUUUUUU----] [UUUUUUUUU---] [---AAAGAAAG-ACU] |AAACGGUGAAACCGC|| GAAAAG |CUC| AUUAAA ||UCGGCUUUGGUUCCUUAGAUAG| ||UAAGCAGAUCUGAU| [UUUUUUUCAAA------] [AAUU--------] [-----UUUGAAAAAAA] |AGAAAAUCUGA|| ||AAU-GGAUAA-UGUGG| UA-AUU |CUAGAGCUAAUACAUG||**

**Xenos vesparum ||GUCUAAGUACUAGCCAA-U| [AAUUCUUUUUUU----] [UUUUUUUUUUUU] [---AAAAAAAGAAGU] |AAACGGUGAAACCGC|| GAAAAG |CUC| AUUAAA ||UCGGCUUUGGUUCCUUAGAUAG| ||UAAGCAGAUCUGAU| [UUUUUUUUAAAAU----] [GUAAA-------] [--AUUUUGAAAAAAAA] |AGAAAAUCUGA|| ||AAU-GGAUAACUGUGG| UA-AUU |CUAGAGCUAAUACAUG||**

**Xenos pecki ||???????UACUAGCCA-CA| [GAUUCUUUUUUUUUUU] [GAAA--------] [AAAAAGAAAAGAAAU] |AAACGGUGAAACCGC|| GAAAAG |CUC| AUUAAA ||UCGGCUUUGGUUCCUUAGAUAG| ||UAAGCAGAUCUGAU| [UUUUUUUCAA-------] [UAAAUUAAU---] [------UUGAAAAAAA] |AGAAAAUCUGA|| ||GAU-GGAUAACUGUGG| UA-AUU |CUAGAGCUAAUACAUG||**

**Xenos sp. ||???????????????????| [????????????????] [????????????] [???????????????] |???????????????|| ?????? |???| ?????? ||??????????????????????| ||??????????????| [?????????????????] [????????????] [????????????????] |???????????|| ||????????????????| ?????? |????????????????||**

**Paraxenos sp. ||GUCUAAGUACUAGCCAAUA| [AUUUUUCUUUU-----] [UUUU--------] [---AAAAGAAAAAGA] |AAACGGUGAAACCGC|| GAAAAG |CUC| AUUAAA ||UCGGCUUUGGUUCCUUAGAUAG| ||UAAGCAGAUCUGAU| [UUUUUUUCGAAAU----] [UUAA--------] [AUAUUUUGAAAAAAAA] |GAAAAAUCUGA|| ||AAU-GGAUAACUGUGG| UA-AUU |CUAGAGCUAAUACAUG||**

**Pseudoxenos sp. ||GUCUAAGUUCUAGCCAAAA| [CCUUAUUUUUUUUUUU] [UAAA--------] [---AAAAAAAGAAAU] |AAACGGUGAAACCGC|| GAAAAG |CUC| CUUAAA ||UCGGCUUUGGUUCCUUAGAUAG| ||UAAGCAGAUGUGAU| [UUUUUUUUAAAAU----] [GUAA--------] [---AUUUUAAAAAAAA] |AGAAAAUCUGA|| ||AAU-GGAUAACUGUGG| UA-AUU |CUAGAGCUAAUACAUG||**

**Stylops mellitae ||GUCUAAGUACUAGCCAAAU| [----------------] [UUUUAA------] [---------------] |AAAUGGUGAAACCGC|| GAAAGG |CUC| AUUAAA ||UCGGCUUUGGUUCCUUAGAUAG| ||UAUGCAGAUCGAAU| [AUUUUUUCAAAAUU---] [UUUGU-------] [--GAUUUUGGAAAAAA] |UGAAAAUCUGA|| ||UAU-GGAUAACUGUGG| UA-AUU |CUAGAGCUAAUACAUG||**

**Tridactylophagus sp. ||GUAUAAGUACUAGCCGGCU| [CUUUA-----------] [UUAAUU------] [------UAAAGAGCG] |AAAUGGUGAAACCGC|| GAAAAG |CUC| AUUAAA ||UCGGCUUUCGUUACUUAGAUAA| ||UUCGCAGUAAGUAA| [UUUUUUUC---------] [GUAU--------] [-------GAAAAAAAU] |UAAAUCACUGA|| ||UAU-GGAUAACUGUGG| UA-AUU |CUAGAGCUAAUACAUG||**

**Halictophagus calcaratus ||GUCUAAGUACUAGCCGAUU| [UUUAU-----------] [UAAUU-------] [------UAAAAAACU] |AAAUGGUGAAACCGC|| AAAAAG |CUC| AUUAAA ||UCGGCUUUGGUUCCUUAGAUAG| ||UUUGCAGUAGAUAA| [UUUUUUUC---------] [UUUAUU------] [-------GAAAAAAAU] |UAAAAUACUGA|| ||UUU-GGAUAACUGUGG| UA-AUU |CUAGAGCUAAUACAUG||**

**Halictophagus sp.(3) ||GUCUCAGUACUAGCCGAUU| [UUAAU-----------] [UUUAU-------] [-------AUUAAAAA] |AAAUGGUGAAACCGC|| GAAAAG |CUC| AUUAAA ||UCGGCUUUGGUUCCUUAGAUAG| ||UUCGCAGAUUGAAA| [UUUUUUUCCAAU-----] [GCAA--------] [--AUUGGAAAAAAAUU] |UAAAUGUCUGA|| ||UAU-GGAUAACUGUGG| UA-AUU |CUAGAGCUAAUACAUG||**

**Halictophagus silwoodensis ||GUCUAAGUACUAGCCGAUU| [UUUUUC----------] [GAAA--------] [----GAAAAAAAAAC] |AAAUGGUGAAACCGC|| AAAAAG |CUC| AUUAAA ||UCGGCUUUGGUUCCUUAGAUAG| ||UUAGCAGUGAACAA| [UUUUUUUC---------] [UUAAUU------] [-------GAAAAAAAU] |UACUAAACUGA|| ||UAU-GGAUAACUGUGG| UA-AUU |CUAGAGCUAAUACAUG||**

**Halictophagus sp.(2) ||GUCUCAGUACUAGCCGAUU| [UUAAU-----------] [UUUAU-------] [-------AUUAAAAG] |AAAUGGUGAAACCGC|| GAAAAG |CUC| AUUAAA ||UCGGCUUUGGUUCCUUAGAUAG| ||UUUGCAGAAUUGAA| [AUUUUUUUCGA------] [UUUCUU------] [----UCGAAAAAAAUU] |AAAAUGUCUGA|| ||UAU-GGAUAACUGUGG| UA-AUU |CUAGAGCUAAUACAUG||**

**Callipharixenos sp. ||GUCUAAGUACUAGCCGAUU| [UUUA------------] [UUAAUU------] [---UAAAAAAAC---] |AAAUGGUGAAACCGC|| AAAAAG |CUC| AUUAAA ||UCGGCUUUGGUUCCUUAGAUAG| ||UUUGCAGUCGGAAA| [AUUUUUUUC--------] [UUAAAU------] [-------GAAAAAAAU] |UCUAAAACUGA|| ||UAU-GGAUAACUGUGG| UA-AUU |CUAGAGCUAAUACAUG||**

**Elenchus sp.(1) ||GUGUAAGUACUAGCCAAAA| [----------------] [CUA---------] [---------------] |AAAUGGUGAAACCGC|| GAAAAG |CUC| AUUAAA ||UCGGCUUUGGUUCCUUAGAUAG| ||UAAGCAGAAAUGAU| [UUUCCAAAAUGUAAAUA] [UUUAUU------] [UAUUUUGUUUUGGAGA] |AAAAAUUCUGA|| ||UAUUGGAUAACUGUGG| UA-AUU |CUAGAGCUAAUACAUG||**

**Elenchus varleyi ||GUGUAAGUACUAGCCAAAA| [----------------] [CUA---------] [---------------] |AAAUGGUGAAACCGC|| GAAAAG |CUC| AUUAAA ||UCGGCUUUGGUUCCUUAGAUAG| ||UAAGCAGAAAUGAU| [UUUUCAAAAAUUAAAU-] [GUAAAA------] [-AUUUAAUUUUGAAAA] |AAAAAUUCUGA|| ||CAUUGGAUAACUGUGG| UA-AUU |CUAGAGCUAAUACAUG||**

**Elenchus koebelei ||GUGUAAGUACUCGCCAAAA| [----------------] [CUA---------] [---------------] |AAAUGGUGAAACCGC|| GAAAAG |CUC| AUUAAA ||UCGGCUUUGGUUCCUUAGAUAG| ||UAAGCAGAAAUGAU| [UUUUCAAUAAUUAAAUA] [AUUA--------] [UAUUUAAUUUUGAAAA] |AAAAAUUCUGA|| ||UAUUGGAUAACUGUGG| UA-AUU |CUAGAGCUAAUACAUG||**

**RAA3 H184a RAA4 RAA4’ H184a' H184b-1 HV2-V4 RAA5 H184b-2 RAA6**

**(((( ....... )))) .... ((((( ((((((((( ........ (((( .......**

**Periplaneta fulig' [CAACUCG-----------] ||GUCC| [-CGACCG----------------------------] [GAGA---] [-------------------------------CGGAAG-] |GGGC|| GCUU ||UUAUU| AGAUC-- |AAAACCAAU| [--------] ||CGGU| [C----------------------------------------------------------] [CUCGC--]**

**Tamolanica tamolana [CAACUCG-----------] ||GUCC| [-CGACCG----------------------------] [GCGA---] [-------------------------------CGGAAG-] |GGAC|| GCUU ||UUAUU| AGAUC-- |AAAACCAAU| [--------] ||CGGU| [C----------------------------------------------------------] [UUCG---]**

**Reticulitermes fla’ [CAACUGA-----------] ||GUCC| [-UGACCG----------------------------] [GCGA---] [-------------------------------CGGAAG-] |GGAC|| GCUU ||UUAUU| AGAUC-- |AAAACCAAU| [--------] ||CGGU| [CGC--------------------------------------------------------] [UCAC---]**

**Antheraea pernyi [CAAUCAGA----------] ||ACUC| [-UGACCA----------------------------] [GUGA---] [-------------------------------UGGGAU-] |GAGU|| GCUU ||UUAUU| AGAUC-- |AAAACCAAU| [--------] ||CGAC| [GGAGGGC----------------------------------------------------] [CUCGC--]**

**Bombyx mori [CAAUCAGA----------] ||ACUC| [-UGACCA----------------------------] [GUGA---] [-------------------------------UGGGAU-] |GAGU|| GCUU ||UUAUU| AGAUC-- |AAAACCAAU| [--------] ||CGAC| [GGAGGGC----------------------------------------------------] [CUCGC--]**

**Haematobia irritans [CAUUAUAAA---------] ||ACAC| [-GGACC-----------------------------] [UUUG---] [---------------------------------GGAC-] |GUGU|| GCUU ||UUAUU| AGGCU-- |AAAACCAAG| [--------] ||CGAU| [C----------------------------------------------------------] [GCAA---]**

**Ceratitis capitata [CAAAAUAA----------] ||ACAC| [-GGACC-----------------------------] [UUUUG--] [---------------------------------GAAC-] |GUGU|| GCUU ||UUAUU| AGGCU-- |AAAACCAAG| [--------] ||CGAU| [C----------------------------------------------------------] [GCAA---]**

**Tribolium castaneum [CAAACAGA----------] ||GCUC| [-CGACCG----------------------------] [GAGA---] [-------------------------------CGGAAG-] |GAGC|| GCUU ||UUAUU| AGAUC-- |AAAACCAAU| [--------] ||CGGU| [GGCGGU-----------------------------------------------------] [UUCG---]**

**Rhagophthalmus ohb' [CAAACAGA----------] ||GCUC| [-CGACCG----------------------------] [GAGA---] [-------------------------------CGGAAG-] |GAGC|| GCUU ||UUAUU| AGAUC-- |AAAACCAAU| [--------] ||CGGC| [GGCGGUCGUC-------------------------------------------------] [UCAAC--]**

**Mengenilla australiensis [AAAAAUGUGA--------] ||GUGC| [GGAAUGUCGAUUGCGC-------------------] [UUCG---] [--------------------GUGUGAUAAAGAUGUUUU] |GCAC|| GCUU ||UUAUU| AGAGGAA |AAAGCCUUU| [-CGAU---] ||CAGU| [CGU--------------------------------------------------------] [GCAA---]**

**Mengenilla chobauti [??????????????????] ||????| [???????????????????????????????????] [???????] [??????????????????????????????????????] |????|| ???? ||?????| ??????? |?????????| [????????] ||????| [???????????????????????????????????????????????????????????] [???????]**

**Triozocera sp.(2) [AAAUUUAAUG--------] ||GUGC| [-GAACGUUCGACGCGACUCUGUCUGC---------] [UCAAUU-] [----------GCGACUAGUCCGUGUCGAUCAAUGUU--] |GCAC|| GCUU ||UUAUU| AGAGGAA |AAAGCCAAU| [UUUAU---] ||CGAC| [CGUGGGCACGGUGUCAUGGUUCACUUGGG------------------------------] [CGAAAAU]**

**Triozocera sp.(3) [AAAUUUAAUG--------] ||GUGC| [-GAACGUUCGAUACGACUGUC--------------] [UGCAAAU] [---------------GACCGUCUGUCGAUCAAUGUU--] |GCAC|| GCUU ||UUAUU| AGAGGAA |AAAGCCAAU| [UUUAU---] ||CGAC| [CGGGUUUACGGUGUCAUUGGGCACUUGAG------------------------------] [UGAAAAU]**

**Triozocera sp.(1) [AAAUUAAAUG--------] ||GUGC| [-GAACGGCCGUUACGGAGGGUUU------------] [GUUACA-] [--------------AAACGAUCUGUCGGUUACUGUU--] |GCAC|| GCUU ||UUAUU| AGAGGAA |AAAGCCAAU| [UUUAU---] ||CGAC| [CGAGUACGACACCAGUAAGUACAUGGAC-------------------------------] [GUUU---]**

**Triozocera sp.(4) [AAAUUUAAUG--------] ||GUGC| [-GAACGUUCGAUAUGAUUGUC--------------] [UGCAAAU] [---------------GACCGUCUGUCGAUCAAUGUU--] |GCAC|| GCUU ||UUAUU| AGAGGAA |AAAGCCAAU| [UUUAU---] ||CGAC| [CGGGUUGCGGUGUCAUUGGGCACUUGAGU------------------------------] [GAAA---]**

**Blisseoxenos esaki [AAAUCGAUAG--------] ||GUGC| [-GAAGCAUGUGAUCACCAUGC--------------] [CAUGC--] [----------------GUGUGGUGUCGCAUACACUUU-] |GCAC|| GCUU ||UUAUU| AGAGGAA |AAAGCCAAU| |UUUAU---] ||CGAC| [AUUGCGCGCGUUCGC--------------------------------------------] [AUUAC--]**

**Corioxenos acucyrtophallus [GAAGAAUUACA-------] ||GUGC| [UGAACACGCCUCAUUG-------------------] [CUUGU--] [--------------------CAAUUUGGUGUCUGUUU-] |GCAC|| GCUU ||UUACU| AGAGGAA |AAAGCCUUU| [UUUAU---] ||CGGU| [GCCACUGUUGCCCGU--------------------------------------------] [UCUA---]**

**Lychnocolax sp.(2) [AAACUAAAAAAAAA----] ||GUGU| [-GAAAUUUUAU------------------------] [GAAA---] [--------------------------AUAAAAUUUUU-] |GCAC|| GCUU ||UUAUU| AGAGUAA |AAAGCCAAU| [UAUAA---] ||----| [UUUUCAAAAUAAAUAAAAAUUUUUUUUCAAUAAAUUUAAAU------------------] [UUAAUU-]**

**Lychnocolax sp.(1) [??????????????????] ||????| [???????????????????????????????????] [???????] [??????????????????????????????????????] |????|| ???? ||?????| ??????? |?????????| [????????] ||????| [???????????????????????????????????????????????????????????] [???????]**

**Stichotrema dallatoreanum [GAAAAGAAACA-------] ||GUGU| [-GAAUGAGAUGUGAUUGUGCGC-------------] [GUUAA--] [-------------GCAAUCGAUCCGUCGCUAAAAUUU-] |ACAC|| GCUU ||UUAUU| AGAGAAA |AAAGCCAAU| [UUUG----] ||----| [AUUUUUGCAUUAUUAAUGCGCGGAGUGUGCG----------------------------] [UUAUG--]**

**Stichotrema sp. [AAAAAUGAAUAG------] ||GUGU| [-GAAGUUCGGCGUGCAU------------------] [UCGC---] [-------------------GUGCGCGCCAGACACUUU-] |GCAC|| GCUU ||UUAUU| AGAGAAA |AAAGCCAAU| [UGCA----] ||----| [GUUUUUCCGCGUUCCGACUCGUGCGUGUGACGGGACGCGUUC-----------------] [UUCG---]**

**Myrmecolax sp.(4) [AAAAUGAAUCG-------] ||GUGU| [-GAAAUUUGUGUGU---------------------] [UGUC---] [------------------------ACAGGCAAAUUUC-] |GCAC|| GCUU ||UUAUU| AGAGAAA |AAAGCCAAU| [UUAG----] ||----| [UUUUUUUGAACCGAUGCGCUUGUCGGGUUGCGGUCGCCGUGGAGAUUAGUUGGC-----] [UUAAUU-]**

**Myrmecolax sp.(3) [AAAAUGAAUCG-------] ||GUGU| [-GAAAUUUGUGUGU---------------------] [UGAC---] [------------------------GCAGGCAAAUUUC-] |GCAC|| GCUU ||UUAUU| AGAGAAA |AAAGCCAAU| [UUAG----] ||----| [UUUUUUUGAACCGAUGCGCAUGUGAAGUGGCGCUUGCCUGGGAAAUGAGUCGGC-----] [UUAAUU-]**

**Myrmecolax sp.(1) [AAAAUGAAUCA-------] ||GUGU| [-GAAAUUCGUGUGU---------------------] [UAAC---] [------------------------GCAUACGGAUUUC-] |GCAC|| GCUU ||UUAUU| AGAGAAA |AAAGCCAAU| [UUAG----] ||----| [UUUUUUUGAAUUGAUGCAUCAGUGGAGCUACGUGUGGCAGAUAGAUAAGUUGGC-----] [UUUAUU-]**

**Myrmecolax sp.(2) [AAAAUGAAUCG-------] ||GUGU| [-GAAAUUUGUGUGU---------------------] [UGAC---] [---------------------GC---AGGCAAAUUUC-] |GCAC|| GCUU ||UUAUU| AGAGAAA |AAAGCCAAU| [UUAG----] ||----| [UUUUUUUGAACCGAUGCGCAUGUGAAGUGGCGCUUGCCUGGGAAAUGAGUCGGC-----] [UUAAUU-]**

**Myrmecholax incautus [AAAAGAAAUCA-------] ||GUGU| [-GAAAUCGUGUACGCA-------------------] [UUAACU-] [--------------------UGCGUAUGUGAA-UUUU-] |GCAC|| GCUU ||UUAUU| AGAGAAA |AAAGCCAAU| [UUAG----] ||----| [UUUUUUGAUCAAAGCCGCGGGAGCCAAUGCGUCGCUGUGCAGUG---------------] [UUUUA--]**

**Myrmecholax sp.(5) [AAAAUGAAUCG-------] ||GUGU| [-GUAAUUUGUGUGU---------------------] [UAUU---] [---------------------GC---AGGCAAAUUUC-] |GCAC|| GCUU ||UUAUU| AGAGAAA |AAAGCCAAU| [UUAG----] ||----| [UUUUUUUGAAACGAUGCGCUAGUUGAGUUGCGGUUGUCGGGGAGAUGAGUUGGC-----] [UUAAUU-]**

**Caenocholax sp.(1) [AAAAA-UUUCG-------] ||GUGU| [-GAAAUUUCGCGACAAAUUAGCA------------] [CUUU---] [-------------UGCUGUUUUGUUGCGUAAAAUUUU-] |GCAC|| GCUU ||UUAUU| AGAGAAA |AAAGCCAAU| [U-------] ||----| [UUAUAAACGUUUUGCAUGUGUGCGCACGUACGCACGCACGCAGACUUGUACGCGCGCAC] [AUUC---]**

**Caenocholax sp.(3) [AAAAAAUUUCG-------] ||GUGC| [-GAAAACGCGUUUGC--------------------] [UUCGA--] [--------------------G-CGAACGUU-GAAUUU-] |GCAC|| GCUU ||UUAUU| AGAGAAA |AAAGCCAAU| [U-------] ||----| [UUAUUAUUGUUUGCGUGUGCACUCUUGCGAAACGGUUGC--------------------] [GUCU---]**

**Caenocholax sp.(10) [AAAAAAAUUCG-------] ||GUGC| [-GAAACUGCGUUUGUU-------------------] [UUU----] [--------------------GACAAACGUUAGGCUUU-] |GCAC|| GCUU ||UUAUU| AGAGAAA |AAAGCCAAU| [U-------] ||----| [UUACUAUUGCUCACGUGUGUCUGUUUUAUUGCGAUGAUGGCAUAACAGUUGUUU-----] [UUUU---]**

**Xenos moutoni [AAAAUUAUUUCC------] ||AUGU| [-GAAAAUAUUUAC----------------------] [GAAA---] [-------------------------GUGAU-AUUUUU-] |GCAU|| GCUU ||UUAUU| AGAGGAA |AAAGCCAAU| [UUUUAA--] ||----| [UAAAAAAUUUAAAAGAAUU----------------------------------------] [CUUGU--]**

**Xenos vesparum [AAAAUUAUUUCA------] ||GUGU| [-GAAAAUAUUUU-----------------------] [AAAAAAA] [--------------------------GGAAUAUUUUU-] |GCAC|| GCUU ||UUAUU| AGAGGAA |AAAGCCAAA| [UUUUAA--] ||----| [AAAAUCUUAAAAAAAC-------------------------------------------] [AUUU---]**

**Xenos pecki [AAAAUUAUUUCA------] ||GUGU| [-GAAAAUAUUAUU----------------------] [AAA----] [-------------------------AAUUUUAUUUUU-] |GCAC|| GCUU ||UUAUU| AGAGGAA |AAAGCCAAU| [UUUUAA--] ||----| [AAAUAAUUAAAGAAAUU------------------------------------------] [AAA----]**

**Xenos sp. [??????????????????] ||????| [???????????????????????????????????] [???????] [??????????????????????????????????????] |????|| ???? ||?????| ??????? |?????????| [????????] ||????| [???????????????????????????????????????????????????????????] [???????]**

**Paraxenos sp. [AAAAUUAUUUCA------] ||GUGU| [-GAAUAAUUAAUUUU--------------------] [UGA----] [----------------------AAAAUUAAUAUAUUU-] |GCAC|| GCUU ||UUAUU| AGAGGAA |AAAGCCAAU| [UUUUUAA-] ||----| [AGUUCGAAGAAAUUUUU------------------------------------------] [GAAA---]**

**Pseudoxenos sp. [AAAAUUAUUUCA------] ||GUGU| [-GAAAAUUUUUU-----------------------] [AAA----] [--------------------------AAGAAAUUUUU-] |GCAC|| GCUU ||UUAUU| AGAGGAA |AAAGCCAAU| [UUUUAU--] ||----| [UAAAUAAAACAUUUAAAAAAUAUAUAUCA------------------------------] [UUAAAU-]**

**Stylops mellitae [AAAAAUGAAAUUGA----] ||GUGC| [-GAAACUUU--------------------------] [GUAAAA-] [-----------------------------AAAGUUUU-] |GCAC|| GCUU ||UUAUU| AGAGGAA |AAAGCCAAU| [--------] ||----| [UUUUAAAAAUGAAAAAAAA----------------------------------------] [UUAUU--]**

**Tridactylophagus sp. [AAAAAUUAGAAAUUUA--] ||GUGC| [-GAAAAUCCnGUGCCGCGUUGUUCUUCCGAGCA--] [AAUCUC-] [UGCUCGGACCACAAUGCGUUGUACUGGUACGAUUUUU-] |GCAC|| GCUU ||UUAUU| AGAGUAA |AAAGCCAAC| [UGAAAA--] ||----| [GCACGUUCGUGUUUUGGA-----------------------------------------] [GCGA---]**

**Halictophagus calcaratus [AAAAUAAAAAGAAUUAAA] ||GUGC| [-GAAUAAAUAUUUAGAAAACUUAUCGAAAUUUC--] [GAAU---] [-GAAAUUCAUGAUAAUAACUAAAUAUUAAUCUAUUUU-] |GCAC|| GCUU ||UUAUU| AGAGUAA |AAAGCCAAC| [CU------] ||----| [UUAAAAACAAAUCAAGAUGUGUCGUUUUUUAGUAUU-----------------------] [GCAA---]**

**Halictophagus sp.(3) [AAAAUACUUUUGUUAA--] ||GUGU| [-GAAAAUCCGCAAAAUUCUAAUGUAUUUUUA----] [UUAAUU-] [-----UAAAAAUAAAUUGAAUUUGUGGAACAAUUUUU-] |GCAC|| GCUU ||UUAUU| AGAGGAA |AAAGCCAAU| [UUUAAAAA] ||----| [GAAUUUUUCGUAAAAAAAUAGUUGAGAUAUUUUUUGC----------------------] [AACU---]**

**Halictophagus silwoodensis [AAAACGAUUUUUUCUAU-] ||GUGC| [-GAAAAUCUAUUCAGAUUUUUCUAUUUG-------] [UUCA---] [----CAAAUAGAAAAGUUUUUGUUUAGGUAAAUUUUU-] |GCAC|| GCUU ||UUAUU| AGAGUAA |AAAGCCAAC| [UUGAUU--] ||----| [UACGUUCGUUUUUAUAAAAAUUUUUACUCGUUGU-------------------------] [GUAU---]**

**Halictophagus sp.(2) [AAAAUACUAUCAUUGAA-] ||GUGU| [-GAAAAUCUGUAAAAAUGAACUGAUUUUUAUUUU-] [ACAU---] [-----AGAAUGAAAGUUCUUUUUAUGGAAUAAUUUUU-] |GCAC|| GCUU ||UUAUU| AGAGGAA |AAAGCCAAU| [U-------] ||----| [UUAUAAGAAUUUUUCGAAU----------------------------------------] [UUU----]**

**Callipharixenos sp. [AAAAUAAAUAAUUAUUAG] ||GUGC| [-GAAAAUUCAAUCAAGUCUAUUUGUUUUAAGCGCC] [UUUC---] [GGCGGCUAAAUUUAUGGCUUGUGUUGGUAAAAUUUUU-] |GCAC|| GCUU ||UUAUU| AGAGUAA |AAAGCCUAA| [C-------] ||----| [AUUUAUUGGUCCGUUUUUUAGGUGAAAGUUUUGAAACGUUUUCU---------------] [UUCGG--]**

**Elenchus sp.(1) [AAAAUUAAAUCA------] ||GUGU| [-GAAAUUUUUUU-----------------------] [GCAA---] [--------------------------AAAAAAAUUUU-] |GCAC|| GCUU ||UUAUU| AGAGGAA |AAAGCCAAU| [UUAA----] ||----| [AAAUAAUUUUUUUU---------------------------------------------] [UUUC---]**

**Elenchus varleyi [AAAAUUAAAUCA------] ||GUGU| [-GAAA-UUUUUU-----------------------] [GUAAAA-] [--------------------------GAAAAUAUUUU-] |GCAC|| GCUU ||UUAUU| AGAGGAA |AAAGCCAAU| [UUAA----] ||----| [AAAUAAUUUUUUUU---------------------------------------------] [CUU----]**

**Elenchus koebelei [AAAAUUAAAUCA------] ||GUGU| [-GAAAUUUUUUU-----------------------] [GCAA---] [--------------------------GAAAAAAUUUU-] |GCAC|| GCUU ||UUAUU| AGAGGAA |AAAGCCAAU| [UUAA----] ||----| [AAAUAAUUUUU------------------------------------------------] [GUAAAA-]**

**RAA6’ H184b-2' RAA7 - “H184b-3” H184b-1' H184c**

**)))) ))))) ....... .((((((**

**Periplaneta fulig' [------------------------------------------------------------------G] |GCCG|| [UUUGCCUU-------------------------------------------------------------------------] GGUGAC---UCUG |AAUAA|| AUUGUG- ||-------|**

**Tamolanica tamolana [------------------------------------------------------------------G] |GCCG|| [UUUGUCUU-------------------------------------------------------------------------] GGUGAC---UCUG |AAUAA|| AUUAUG- ||-------|**

**Reticulitermes fla’ [----------------------------------------------------------------GCG] |GCCG|| [UUUGCCUU-------------------------------------------------------------------------] GGUGAC---UCUG |AAUAA|| AUUGAG- ||-------|**

**Antheraea pernyi [------------------------------------------------------------GUCCGAA] |GUCG|| [UUAAUUUU-------------------------------------------------------------------------] GAUGAA---UCUG |GAUAA|| CUUUU-- ||-------|**

**Bombyx mori [------------------------------------------------------------GUCCGAA] |GUCG|| [UUAAUUUU-------------------------------------------------------------------------] GAUGAA---UCUG |GAUAA|| CUUUU-- ||-------|**

**Haematobia irritans [------------------------------------------------------------------G] |AUCG|| [AUACUUUG-------------------------------------------------------------------------] GUUGAA--CUCUA |GAUAA|| CUU---- ||-------|**

**Ceratitis capitata [------------------------------------------------------------------G] |AUCG|| [UUAUAUUG-------------------------------------------------------------------------] GUUGAA--CUCUA |GAUAA|| CUU---- ||-------|**

**Tribolium castaneum [--------------------------------------------------------------CCGUC] |AUCG|| [UACAACUU-------------------------------------------------------------------------] GGUGAA---UCUG |AAUAA|| CUUUA-- ||-------|**

**Rhagophthalmus ohb' [---------------------------------------------------------GGCGUCCGUC] |GUCG|| [UUCGCUCU-------------------------------------------------------------------------] GGCGAA---UCUG |AAUAA|| CUUCCU- ||-------|**

**Mengenilla australiensis [----------------------------------------------------------------GCG] |AUUG|| [UAGAAACGAA-----------------------------------------------------------------------] GGUGAUGAAUCUG |AAUAA|| CUUGAA- ||CGGGU-C|**

**Mengenilla chobauti [???????????????????????????????????????????????????????????????????] |????|| [?????????????????????????????????????????????????????????????????????????????????] ????????????? |?????|| ??????? ||???????|**

**Triozocera sp.(2) [---------------------------CUCAUGUGACCGCCGUGAUCCGUGCCCAAUUCAAAAAUGU] |GUCG|| [AGAGAGCACCCGUUUCUGUGUGCCGUCGUUGCACUUGAUCGUGCACGACGUUCUUAAGCU---------------------] GGUGUUGAAUCUG |AAUAA|| CUUGAA- ||CGAAUUC|**

**Triozocera sp.(3) [---------------------------CUCAUGUG---CCCGUGACCCGUUUACCAAUUCAAA-UGU] |GUCG|| [AGAGAGCACCUGUUUGUGUGCCGUCGUGCAUUUCGUGCACGACGUUCUUAAAGCU--------------------------] GGUGUUGAAUCUG |AAUAA|| CUUGAA- ||CGAAUUC|**

**Triozocera sp.(1) [---------------------------------GUUUAUGCGCUUACGGUUCGUGCUCUUUAAUUGU] |GUCG|| [AGAAAGAUCGGUACUUGUGUUAGCGAGUAUCGUUCGUGCUGUUCGCGCGGGCGUAAAUGCUUGCACGAUCAACUUUACACU] GGUGUUGAAUCUG |AAUAA|| CUUGAA- ||CGAGUCC|**

**Triozocera sp.(4) [-----------------------------AUCUCAUGUGCCCGUGACCCGUUUACCAAUUGAAAUGU] |GUCG|| [AGAGAGCACCUGUUUUGUGUGCGCCGUCGUGCACAUUUCGUGCACGACGUUCUUAAGCU----------------------] GGUGUUGAAUCUG |AAUAA|| CUUGAA- ||CGAAUUC|**

**Blisseoxenos esaki [----------------------------------GUGAACGCGUGCACAUCUGUUACAGAUGGAAAC] |GUCG|| [AGACAAAUUUAAACACUU---------------------------------------------------------------] GGUGUUGAAUCUG |AAUAA|| CUUGAA- ||CGAAUCU|**

**Corioxenos acucyrtophallus [--------------------------------------------------ACGAGCGACUUUGUCAU] |ACCG|| [AAAUAAAAAAAUU--------------------------------------------------------------------] GGUGUUGAAUCUG |AGUAA|| CUUGGA- ||UGAAUCC|**

**Lychnocolax sp.(2) [------------------------------AUUUAAAUUUUUAUGGAAAAAAAUUAAUUUUUGAAAA] |----|| [AUUUUAAUAUUAAAAU-----------------------------------------------------------------] GGUGUUGAAUCUG |AAUAA|| CUUGAA- ||CGGAUUU|**

**Lychnocolax sp.(1) [???????????????????????????????????????????????????????????????????] |????|| [?????????????????????????????????????????????????????????????????????????????????] ????????????? |?????|| ??????? ||???????|**

**Stichotrema dallatoreanum [-------------------------------------CGCAUAUUCCGUUGUAAAUUUUGCUGAAAU] |----|| [AAAACACGUAAAAU-------------------------------------------------------------------] GGUGUUGAAUCUG |AAUAA|| CUUGAA- ||UGAAUUU|**

**Stichotrema sp. [-------------------------GAACGAUGUCUCUGUCGCCGAGAGUGGUGAAUGGCGAAAAAU] |----|| [GAACCAAAAAA----------------------------------------------------------------------] GGUGUUGAAUCUG |AAUAA|| CUUGAA- ||UGGAUUU|**

**Myrmecolax sp.(4) [----------------GCUUUCGAAUUUUCUAGGCACCGUGGCUUGUGCUGUGCGUCGACAAAAAAG] |----|| [AAUCAAUUAAAAAU-------------------------------------------------------------------] GGUGUUGAAUCUG |AAUAA|| CUUAAA- ||CGGAUUU|**

**Myrmecolax sp.(3) [----------------GCUUUCGAAUUUCUUCGGCACUGCCGCUUGUGCUGCGCGUCGACAAAAAAG] |----|| [AAUCAAUCAAAAAU-------------------------------------------------------------------] GGUGUUGAAUCUG |AAUAA|| CUUAAA- ||CGGAUUU|**

**Myrmecolax sp.(1) [----------------GCUUACGAAUUUUUUUGCAGCGUACGCUUAAGCUGUGUAUCGUCAAAAAAG] |----|| [AAUCAAUUUUAGAAAU-----------------------------------------------------------------] GGUGUUGAAUCUG |AAUAA|| CUUAAA- ||CGGAUUU|**

**Myrmecolax sp.(2) [-----------------GCUUUCGAAUUUCUUCGGCACUGCCGCUUGUGCUGCGCGUCGACAAAAAA] |----|| [GAAUCAAUCAAAAA-U-----------------------------------------------------------------] GGUGUUGAAUCUG |AAUAA|| CUUAAA- ||CGGAUUU|**

**Myrmecholax incautus [----------------------CAUUGUGCUCGUGUGCAUUUGCGUUCUCGGGUGAUUGACGAAAAG] |----|| [AAGUUUAACAAAAAAU-----------------------------------------------------------------] GGUGUUGAAUCUG |AAUAA|| CUUGAA- ||CGGGUUC|**

**Myrmecholax sp.(5) [----------------GCCUUUGAAUUUUCUCGGCACUGUUGCUUGUGCUGUGCGUCGACAAAAAAG] |----|| [-AAUCAAUUAAAAA-U-----------------------------------------------------------------] GGUGUUGAAUCUG |AAUAA|| CUUAAA- ||CGGAUUU|**

**Caenocholax sp.(1) [GUGUGCGUUACAAUGUCGCGUCGUCGUCGUCGUUGUCGUCACGUUAUGCGAAUAAAUACAAUCGUAA] |----|| [----AAAUC------------------------------------------------------------------------] GGUGUUGAAUCUG |AAUAA|| CUUGAAA ||CGG-UUU|**

**Caenocholax sp.(3) [------------------------GUGAUCUGUUUCUGCUGGAGCGGCUACgUAAAAUCAAUACUAA] |----|| [UGAAAAAAUUA----------------------------------------------------------------------] GGUGUUGAAUCUG |AAUAA|| CUUGAA- ||UGGAUUC|**

**Caenocholax sp.(10) [--------------GAAUAGCUGUUUUCUAUUAUUGUUUAAACGGCUGCGUGUGAAACCAAUACUAA] |----|| [UAAAAAUUUU-----------------------------------------------------------------------] GGUGUUGAAUCUG |AAUAA|| CUUGAA- ||UGGAUUC|**

**Xenos moutoni [----------------------------------------AAUUUUUUUAAAUUUAUUAAUUUAAAA] |----|| [UACGUUUAAAAAAUUUUAUUAUAUAUUGAUAAUUUUUUUUGAAAU------------------------------------] GGUGUUGAAUCUG |AAUAA|| CUUGAA- ||UGAAUUU|**

**Xenos vesparum [----------------------------------------GUUUUUUUUUGAUUAAUAAAUUUAAUA] |----|| [AUCGUUUAAAAAAUAUCAUUAAUUUGAUAGUUUUUUAAAAU----------------------------------------] GGUGUUGAAUCUG |AAUAA|| CUUGAA- ||UGAAUUU|**

**Xenos pecki [-------------------------------------------------AAUUUUUUUAAUUAUUUU] |----|| [AUUUAAUUCAUUUAAAAAAUUUAUCAUUAAUUUGAUAAUUUUUUAGAAU--------------------------------] GGUGUUGAAUCUG |AAUAA|| CUUGAA- ||UGAAUUU|**

**Xenos sp. [???????????????????????????????????????????????????????????????????] |????|| [?????????????????????????????????????????????????????????????????????????????????] ????????????? |?????|| ??????? ||???????|**

**Paraxenos sp. [-------------------------------------------------AAUAAUUUGUUUCGAACU] |----|| [GAAAUAAUAAAUUAUAAUAAAUCAUUUAAAAAAUAUAUAUCAUUUAAGAUAUAAUUUUUUAAAAU----------------] GGUGUUGAAUCUG |AAUAA|| CUUGAA- ||UGAAUUU|**

**Pseudoxenos sp. [----------------------------------------------------UGAUAAUUUUUUAAA] |----|| [AU-------------------------------------------------------------------------------] GGUGUUGAAUCUG |AAUAA|| CUUGAA- ||UGAAUUU|**

**Stylops mellitae [----------------------------------------------UUUUUUUUCUGAAAUUU-AAA] |----|| [CCUAAUAUAAAAU--------------------------------------------------------------------] GGUGUUGAAUCUG |AAUAA|| CUUGAA- ||UGAAUUU|**

**Tridactylophagus sp. [-------------------------------------------------UCCAGUUCGCGAACGUGU] |----|| [CUAACGAAUAUGAAUCCUUUUUAUUGCAAACUGGAUUUCAGA---------------------------------------] AAUGUUGAAUCUG |AAUAA|| CUUGAA- ||CGAAUCG|**

**Halictophagus calcaratus [-----------------------------AAUAUUAAAAAUUGUACACUCUUUGUAUUUGUUUAUGA] |----|| [UCGUUAGAAAUCUAUUCAUUUUUUGAAUUGAUUUCAAU-------------------------------------------] AAUGUUGAAUCUG |AAUAA|| CUUGAA- ||CGAAUCA|**

**Halictophagus sp.(3) [------------------------------------GCAAAAAUAUCAUAAAUUAUUUGAAAAAUUC] |----|| [GUGAAUUAUUAUAAUUAAUUUUUAAUCAAAAAAUUAAUUCUAUU-------------------------------------] AAUGUUGAAUCUG |AAUAA|| CUUGAA- ||UGAAUCU|**

**Halictophagus silwoodensis [--------------------------------------AUAACGGUAAUUUUUUUAUUACGUUCGUA] |----|| [UAUAAACAUUACAAAUUUUUUUAUAUUAAAAAAAAAUUUGACU--------------------------------------] AAUGUUGAAUCUG |AAUAA|| CUUGAA- ||UGAAUCU|**

**Halictophagus sp.(2) [------------------------------------------------AUUUGAAAAAUUUGUUUGA] |----|| [UCUUGAAAAUUAAUUCGACAAACGAAUUAAUUUGAUU--------------------------------------------] GAUGUUGAAUCUG |AAUAA|| CUUGAA- ||UGAAUCU|**

**Callipharixenos sp. [-----------------------GGAAAUUGUAUUCUACUUUUAUCGUUUAAAUCGGCCAGAUAAAU] |----|| [CAAUAUGAAUUUUUUUUUAAUUGAAAAAAAAAUUUCACC------------------------------------------] AAUGUUGAAUCUA |AAUAA|| CUUGAA- ||CGGGUCU|**

**Elenchus sp.(1) [-----------------------------------------------------AAAAAAAAUUAUUU] |----|| [AAUAUUAAUUAAAUAAAUUUAAAAUUUUAACAUUUUAAAUUUUUUU-----------------------------------] AGUGUUGAAUCUG |AAUAA|| CUUAAA- ||CAAAUUU|**

**Elenchus varleyi [-----------------------------------------------------AAAAAAAAUUAUUU] |----|| [AAUAUUUAUAAAAUAAAUUUGAAAUUGUAAAAAUUUUAAAUUUUUUA----------------------------------] AGUGUUGAAUCUG |AAUAA|| CUUAAA- ||CAAAUUU|**

**Elenchus koebelei [--------------------------------------------------------GAAAAUUAUUU] |----|| [AAUAUUAAUUAAAUAAAUUUAAAAUUUAAAC-AUUUUAAAUUUUUUA----------------------------------] AGUGUUGAAUCUG |AAUAA|| CUUAAA- ||CAAAUUU|**

**RAA8 RAA8’ H184c'**

**........ )))))).**

**Periplaneta fulig' [--------------------------------------------------------------------------------------------------------------------------------------] [--------] [------------------------------------------------------------------------------------------------------------------------------] |-------||**

**Tamolanica tamolana [--------------------------------------------------------------------------------------------------------------------------------------] [--------] [------------------------------------------------------------------------------------------------------------------------------] |-------||**

**Reticulitermes fla’ [--------------------------------------------------------------------------------------------------------------------------------------] [--------] [------------------------------------------------------------------------------------------------------------------------------] |-------||**

**Antheraea pernyi [--------------------------------------------------------------------------------------------------------------------------------------] [--------] [------------------------------------------------------------------------------------------------------------------------------] |-------||**

**Bombyx mori [--------------------------------------------------------------------------------------------------------------------------------------] [--------] [------------------------------------------------------------------------------------------------------------------------------] |-------||**

**Haematobia irritans [--------------------------------------------------------------------------------------------------------------------------------------] [--------] [------------------------------------------------------------------------------------------------------------------------------] |-------||**

**Ceratitis capitata [--------------------------------------------------------------------------------------------------------------------------------------] [--------] [------------------------------------------------------------------------------------------------------------------------------] |-------||**

**Tribolium castaneum [--------------------------------------------------------------------------------------------------------------------------------------] [--------] [------------------------------------------------------------------------------------------------------------------------------] |-------||**

**Rhagophthalmus ohb' [--------------------------------------------------------------------------------------------------------------------------------------] [--------] [------------------------------------------------------------------------------------------------------------------------------] |-------||**

**Mengenilla australiensis [-UUUCGUGAAAGGAAUGGCC------------------------------------------------------------------------------------------------------------------] [GCAA----] [-----------------------------------------------------------------------------------------------------------GGUUGUUUCCGGUAGCGGA] |GGAUUUA||**

**Mengenilla chobauti [??????????????????????????????????????????????????????????????????????????????????????????????????????????????????????????????????????] [????????] [??????????????????????????????????????????????????????????????????????????????????????????????????????????????????????????????] |???????||**

**Triozocera sp.(2) [GUUUCGCUCAGGCGAACAAUCGGCUCGGAUUUGUGCGCG-----------------------------------------------------------------------------------------------] [UUCA----] [-----------------------------------------------------------------------------------------CGUGUGCGUCCGUGCCGUUGUCCGUGUCGGGCGGGAC] |GGAUUUA||**

**Triozocera sp.(3) [GUUCCGCUUAGGCGAACAAUCGGCUCGGACGCGC----------------------------------------------------------------------------------------------------] [UUGC----] [---------------------------------------------------------------------------------------------GCGUGUGUGUGCCGUUGUUCGUGUCGGCGGGAC] |GGAUUUA||**

**Triozocera sp.(1) [GUUUCGCUUAGGCGGAUUAACGUCGCGCGUGUGC----------------------------------------------------------------------------------------------------] [UUCG----] [--------------------------------------------------------------------------------------------GCGUGCACGUGAUUGUUGUCCGUGUCGGGCGAAC] |GGAUUUA||**

**Triozocera sp.(4) [GUUUCGCUUAGGCGAACAAUCGGCUCGGAUGCGC----------------------------------------------------------------------------------------------------] [UCAC----] [---------------------------------------------------------------------------------------------GCGUGUGUGCGCCGUUGUUCGUGUCGGCGGAAC] |GGAUUUA||**

**Blisseoxenos esaki [GUGCGCACGGGUCUGUCGCGUGCCUGUCGCGCCCGCAC------------------------------------------------------------------------------------------------] [UUCG----] [------------------------------------------------------------------------------------GUGCGAGCGCGCGACUCGAAUGCGACGGGCCUCGGUGUGCAC] |GGGUUUA||**

**Corioxenos acucyrtophallus [UUGGAACGCUGUGUGUAGUUGCAGACAC----------------------------------------------------------------------------------------------------------] [UUUUA---] [-------------------------------------------------------------------------------------------------GUGACUGUUCAUUGCGCAUCGGUGUCCGA] |GGGUUCA||**

**Lychnocolax sp.(2) [UAUUUUGAAAGAAUUUUAUUA-----------------------------------------------------------------------------------------------------------------] [AUUAUA--] [--------------------------------------------------------------------------------------------------------UAAUAAAAUUUGAUUAGAAAUA] |GAAUUUA||**

**Lychnocolax sp.(1) [??????????????????????????????????????????????????????????????????????????????????????????????????????????????????????????????????????] [????????] [??????????????????????????????????????????????????????????????????????????????????????????????????????????????????????????????] |???????||**

**Stichotrema dallatoreanum [CCUGCGAAAGAAUUCGAUGUAUUUGAGUUACAUGAUUGUUCAAUUGAGCCAUGCGCAUGCA-------------------------------------------------------------------------] [UUUG----] [-----------------------------------------------------------------UGUGUGUGUGUGUGUUUCAAUGAUGGUCGUUUUGUGCUCACUUUGAAUUCGA-UUAGAAGG] |GAAUUUA||**

**Stichotrema sp. [UCCUUGAAAGGGUUUUGUAGUGUGCCGUGCGCUCC---------------------------------------------------------------------------------------------------] [UUCG----] [-----------------------------------------------------------------------------------------GGCGCGGUCGGUGCGCGUGUGAAAUCCGACUAGAGGA] |AGAUUUA||**

**Myrmecolax sp.(4) [CUUUCGAAAGAAUUUGUGAUAGGAGCUGC---------------------------------------------------------------------------------------------------------] [UUUG----] [-------------------------------------------------------------------------------------------------GCAGUUUCGAUGCAUAUUCGAUUAGAAGG] |AAAUUUA||**

**Myrmecolax sp.(3) [CUUUCGAAAGAAUUUGCAACAGAAGCUGC---------------------------------------------------------------------------------------------------------] [CUCA----] [-------------------------------------------------------------------------------------------------GUGGUUUCGAUGCAUAUUUGAUUAGAAGG] |AAAUUUA||**

**Myrmecolax sp.(1) [CUUUCGAAAGAAUUUGUGACGAAGUGCUC---------------------------------------------------------------------------------------------------------] [UUUG----] [--------------------------------------------------------------------------------------------------GCAUUUCACUGCAGAUUCGAUUAGGGAG] |AGAUUUA||**

**Myrmecolax sp.(2) [CUUUCGAAAGAAUUUGCAACAGGA-GCUGCC-------------------------------------------------------------------------------------------------------] [GUCCU---] [-----------------------------------------------------------------------------------------------GGCAGUUUCGAUGCAUAUUUGA-UUAGAAGG] |AAAUUUA||**

**Myrmecholax incautus [CUUUUGAAAGAAUUUAUCUCGAUGUUACUGCG------------------------------------------------------------------------------------------------------] [UUUAC---] [------------------------------------------------------------------------------------------------------------------UGUUUGUGAAUG] |GAUUUUA||**

**Myrmecholax sp.(5) [CGUUCGAAAGAAUUUGCGAUAGGA-GCUGU--------------------------------------------------------------------------------------------------------] [CUCG----] [----------------------------------------------------------------------------------------------GCAGUUGCGAUGUGCAUAUUUGA-CUAGAAUG] |GAAUUUA||**

**Caenocholax sp.(1) [UCUUUGAAAGAAUUCUUUUUCUA---------------------------------------------------------------------------------------------------------------] [UUUGCU--] [------------------------------------------------------------------------------------------------------UAGAAAGGAAUUUGAACUAGGAGA] |AGAUUUA||**

**Caenocholax sp.(3) [UCUUUGAAAGAGUUUUAUUUCG----------------------------------------------------------------------------------------------------------------] [UUU-----] [------------------------------------------------------------------------------------------------------CGGAAUAGAAUUUGA-UUAGAAGA] |GGGUUUA||**

**Caenocholax sp.(10) [UCUUUGAAAGAAUUUUGUUUCG----------------------------------------------------------------------------------------------------------------] [UUU-----] [------------------------------------------------------------------------------------------------------CGGAAUGAAAUUUGA-UUAGAAGA] |GGGUUUA||**

**Xenos moutoni [CUUUUGAAAAAUUUUUUUAUUGUAUUAAUGUUUUUAUAAUUGUGUAUUUUUUUUUUUUAAAAUUUUUUUUAAAUUAUAUAAAAUCUAAAUAAAAAUUAUUAUACAUAUAAUAUUUAAUAAUAUAUAAAUAUAUU] [UCUU----] [AAUGUGUUUAUAUUUUUUAAUAUUUUAUAUGUAUAUUUUUAUUAUAUAUUUUUUAUAUAUUAAAAAAAGAAAGUAAAAAAGAAAAAAUCCAAUUAUAUUUAUUUCGAUAAAUUAUUGAUUAGAAAG] |AAAUUUA||**

**Xenos vesparum [CUUUUGAAAAAUAUUUAUCGUAUUAAUGUUUUUAUAACUUUUUUUAAUUUUUUUUUAAAAAUUUUAUUUAAAUAAUAUAUAUAUAUAGACA-------------------------------------------] [CUUA----] [-----------------------------------------UGUGUAUGUAUGUAGAAUUAUUUAAUUAUAAAUUAAAAAAAAAAAUAAAAAGUUAUAUUUAUUUCGAUAAAUUUUAAUUAGAAAG] |AAAUUUA||**

**Xenos pecki [CUUUUGAAAAAUAUUUAUUGUAUUAAGGUUUUUAUAACUUUUUUUUUUUUAAAAAAAAUUUAUUUAUUUAU---------------------------------------------------------------] [UUU-----] [-----------------------------------------------------------AUAUUUAAAUAAAAUUAAUUAAAAAAAAAAUAAGUUAUAUUUAUUUCGAUAAAUUUUUAUUAGAAAG] |AAAUUUA||**

**Xenos sp. [??????????????????????????????????????????????????????????????????????????????????????????????????????????????????????????????????????] [????????] [??????????????????????????????????????????????????????????????????????????????????????????????????????????????????????????????] |???????||**

**Paraxenos sp. [CUUUUGAAAAUUAUUUAUCGUUAUUAUAUCUUUUUUAUAAAUUAAUAU--------------------------------------------------------------------------------------] [UUU-----] [-----------------------------------------------------------------------AUAUUAAUUUUAAUGAAAAA GUUUAAUUUAUGUCGAUAAAUUUUGAUUAGAAAG] |AAAUUUA||**

**Pseudoxenos sp. [CUUUUGAAAAAUAUUUAUCGUAUAGAAUAUUUUAUAAUUUUUAUUGAAUUUAAUUUUUUCAAAAUAAAUAUAAACAUUAAAAUUUAUUAUAU------------------------------------------] [UUCG----] [----------------------------------GUGUAAUUAUUUUAUGUUUAAAAUGAUAUUUAUUAGAAAAAAUAUUUUUAAAAAAAAAUAUAAAAUAUUUCGAUAAAUUUUUAAGUAAAAAG] |AAAUUUA||**

**Stylops mellitae [CUUUUGAAAAAAUUUAUUUGUAUAAAUUUUUUUUAU--------------------------------------------------------------------------------------------------] [UCU-----] [-----------------------------------------------------------------------------------------AUAAGGAAAAUUUUUAAGAAUAAAUUUGAUUAGAGAG] |AAAUUUA||**

**Tridactylophagus sp. [UUCUCGAAAUCUAGUAGGCUCGCGAC------------------------------------------------------------------------------------------------------------] [CGUAAAAA] [----------------------------------------------------------------------------------------------------GUUGUGAUCUAUUAGCGAUUAGAGAG] |CGAUUUA||**

**Halictophagus calcaratus [UUUUACGAAAACUAAUCGAUAAGAAU------------------------------------------------------------------------------------------------------------] [UAGUA---] [---------------------------------------------------------------------------------------------------GUUCCCAUUCGAUUAGUAAUUAGUGAA] |UGGUUUA||**

**Halictophagus sp.(3) [UUUCCGAAAGUAUAUUAUUUGGAGAAUUUUUCUCU---------------------------------------------------------------------------------------------------] [UUCG----] [--------------------------------------------------------------------------------------GGAUGAAAAAUUUUUUAAAAAAUUUUAUACGAUUAGGGAG] |AGAUUUA||**

**Halictophagus silwoodensis [UUUUCGAAAAUCGAUUGCAUAAGAAAUUU---------------------------------------------------------------------------------------------------------] [GUAAAA--] [-------------------------------------------------------------------------------------------------AGAUUUUUUAUCUAUUGGUGAUUAGAAAA] |AGAUUUA||**

**Halictophagus sp.(2) [UUUCUGAAAGUAUAUUAUUAGGAAAUUUUUCGCCUCU-------------------------------------------------------------------------------------------------] [UAAC----] [------------------------------------------------------------------------------------GGAGAGUGGAAAAUUUUUGAAUAAUAUUAUACGAUUAGGGAA] |AGAUUUA||**

**Callipharixenos sp. [UUACUGAAAGUUUUCGAAAAAACAAU------------------------------------------------------------------------------------------------------------] [CGUAAAAA] [----------------------------------------------------------------------------------------------------AUUGUUUUUCGUUAACGAUUAGGAAA] |AGGUUUA||**

**Elenchus sp.(1) [CUAUAUAAAAAAUAUGUAAUUUAAUUGAUAAUUUAUUAUUGAACUGGU--------------------------------------------------------------------------------------] [GCAA----] [-------------------------------------------------------------------------ACCAGUUUUAAUGAUGAUUGUUAAUUAUUUAUAUAGUUUUUUAAUUAUUAUAG] |AAGUUUA||**

**Elenchus varleyi [CUAUAAAAAAAAUUAUGUUAUUUUUUAAUGUUUUUUAUAAGUAUCA----------------------------------------------------------------------------------------] [UUUA----] [-----------------------------------------------------------------------------UGAUAUUUUUUGAAAUGUUAAAAUUUAUAUAGUUUUUUUAUUAUUAUAG] |AAAUUUA||**

**Elenchus koebelei [CUAUAAAAAGAAUGUGUAAAUUAUUAAUUUUACAUAAUUAGAAUUGGU--------------------------------------------------------------------------------------] [GCAA----] [--------------------------------------------------------------------------ACUGGUGAUAAUUUUGAAAAAUUAAUGUUUAUGUAUUUUUUUAUUAUUAUAG] |AAGUUUA||**

**H198 RAA9 RAA9’ H198' H122' H240 H240' H289 H289' H113' H316**

**....... ((((( ......... ))))) .. ))))))...)).))))))) (((..(.(((....((((((((( ..... )))))))))))).....)))) ... (((((((( .... ))))...)))) ))) . ((((((( ........**

**Periplaneta fulig' --GCAGA ||UCGCA| [CGGU----------------------------------------------] [CUCCGU---] [----------------------------------------ACC] |GGCGA|| -C |GCAUCUUUCAAAUGUCUGC|| ||CUUAUCAACUGUCGAUGGUAGGU| UCUGC |GCCUACCAUGGUUGUAACGGG|| UAA ||CGGGGAAU| CAGG |GUUCGAUUCCG|| |GAG| A ||GGGAGCC| UGAGAAAC**

**Tamolanica tamolana --GCAGA ||UCGCA| [CGGC----------------------------------------------] [CCCCGA---] [----------------------------------------GCC] |GGCGA|| -C |GCAUCUUUCAAAUGUCUGC|| ||CUUAUCAACUGUCGAUGGUAGGU| UCUGC |GCCUACCAUGGUUGUAACGGG|| UAA ||CGGGGAAU| CAGG |GUUCGAUUCCG|| |GAG| A ||GGGAGCC| UGAGAAAC**

**Reticulitermes fla’ --GCGGA ||UCGCA| [CGGU----------------------------------------------] [CUCCGA---] [----------------------------------------ACC] |GGCGA|| -C |GCAUCUUUCAAAUGUCUGC|| ||CUUAUCAACUGUCGAUGGUAGGC| UCUGC |GCCUACCAUGGUUGUAACGGG|| UAA ||CGGGGAAU| CAGG |GUUCGAUUCCG|| |GAG| A ||GGGAGCC| UGAGAAAC**

**Antheraea pernyi --GCAGA ||UCGCA| [UGGU----------------------------------------------] [CAAGU----] [----------------------------------------ACC] |GGCGA|| -C |GCAUCUUUCAAAUGUCUGC|| ||CUUAUCAACUUUCGAUGGUAGUU| UCUGC |GACUACCAUGGUUGUCACGGG|| UAA ||CGGGGAAU| CAGG |GUUCGAUUCCG|| |GAG| A ||GGGAGCC| UGAGAAAC**

**Bombyx mori --GCAGA ||UCGCA| [UGGU----------------------------------------------] [CAAGU----] [----------------------------------------ACC] |GGCGA|| -C |GCAUCUUUCAAAUGUCUGC|| ||CUUAUCAACUUUCGAUGGUAGUU| UCUGC |GACUACCAUGGUUGUCACGGG|| UAA ||CGGGGAAU| CAGG |GUUCGAUUCCG|| |GAG| A ||GGGAGCC| UGAGAAAC**

**Haematobia irritans --GCAGA ||UCGUA| [UGGU----------------------------------------------] [CUUGU----] [----------------------------------------ACC] |GACGA|| -C |AGAUCUUUCAAAUGUCUGC|| ||CCUAUCAACUUUUGAUGGUAGUA| UCUAG |GACUACCAUGGUUGCAACGGG|| UAA ||CGGGGAAU| CAGG |GUUCGAUUCCG|| |GAG| A ||GGGAGCC| UGAGAAAC**

**Ceratitis capitata --GCAGA ||UCGUA| [UGGU----------------------------------------------] [CCCGU----] [----------------------------------------ACC] |GACGA|| -C |AGAUCUUUCAAACGUCUGC|| ||ACUAUAAACGAUUGGGGGUAGCA| UCUAG |GACCACCAUGG-CGCAACGGG|| AAA ||CGAGGAAU| CAGG |GUUCGAUUCCG|| |GAG| A ||GGGAGCC| UGAGAAAC**

**Tribolium castaneum -CGCUGA ||UCGCA| [CGGU----------------------------------------------] [CUCGC----] [----------------------------------------ACC] |GGCGA|| -C |GCAUCUUUCAAAUGUCUGC|| ||CUUAUCAACUGUCGAUGGUAGGU| UCUGC |GCCUACCAUGGUUGUAACGGG|| UAA ||CGGGGAAU| CAGG |GUUCGAUUCCG|| |GAG| A ||GGGAGCC| UGAGAAAC**

**Rhagophthalmus ohb' --GCUGA ||UCGCA| [CGGU----------------------------------------------] [CUCGC----] [----------------------------------------ACC] |GGCGA|| -C |GCAUCUUUCAAAUGUCUGC|| ||CUUAUCAACUGUCGAUGGUAGGU| UCCGU |GCCUACCAUGGUCGUAACGGG|| UAA ||CGGGGAAU| CAGG |GUUCGAUUCCG|| |GAG| A ||GGGAGCC| UGAGAAAC**

**Mengenilla australiensis AAGCAGA ||CCGCA| [AAUUA-----AUUGUCGCUGCUGGACUC----------------------] [AUUGC----] [-------------------------GAGCCGGCUCGAUGAGGU] |UGCGG|| -C |GUGUCUUUCAAUUGUCUGC|| ||CUUAUCAACUUUCGAUGGUAGGU| UACAU |GCCUACCAUGGUUGUAACGGG|| UAA ||CGGGGAAU| CAGG |GUUCGAUUCCG|| |GAG| A ||GGGAGCC| UGAGAAAC**

**Mengenilla chobauti ??????? ||?????| [??????????????????????????????????????????????????] [?????????] [???????????????????????????????????????????] |???GG|| -C |GUGUCUUUCAAUUGUCUGC|| ||CUUAUCAACUUUCGAUGGUAGGU| UACAU |GCCUACCAUGGUUGUAUCGGG|| UAA ||CGGGGAAU| CAGG |GUUCGAUUCCG|| |GAG| A ||GGGAGCC| UGAGAAAC**

**Triozocera sp.(2) AAGCAGA ||CCGCA| [GUUAAUGGUGAUUGUCGUCGGCGGCAC-----------------------] [UCUAACC--] [--------------------GUGUGCGCGACGACGGGAGCCUU] |UGCGG|| -C |GUAUCUUUCAAUUGUCUGC|| ||CUUAUCAACUUUCGAUGGUAGAU| UACAU |GCCUACCAUGGUUGUAACGGG|| UAA ||CGGGGAAU| CAGG |GUUCGAUUCCG|| |GAG| A ||GGGAGCC| UUAGAAAC**

**Triozocera sp.(3) AAGCAGA ||CCGCA| [GUUAAUGGUGAUUGUCGUCGGCU---------------------------] [AUCUA----] [-----------------------AGUGUGACGACGGGAGCCUU] |UGCGG|| -C |GUAUCUUUCAAUUGUCUGC|| ||CUUAUCAACUUUCGAUGGUAGAU| UACAU |GCCUACCAUGGUUGUAACGGG|| UAA ||CGGGGAAU| CAGG |GUUCGAUUCCG|| |GAG| A ||GGGAGCC| UUAGAAAC**

**Triozocera sp.(1) AAGCAGA ||CCGCA| [GUUAAUGGUGAUUGCCGUCGUCUU--------------------------] [UUCU-----] [----------------------AAGUGCGUCGGCAGAAGCCUU] |UGCGG|| -C |GUAUCUUUCAAUUGUCUGC|| ||CUUAUCAACUUUCGAUGGUAGAU| UACAU |GCCUACCAUGGUUGUAACGGG|| UAA ||CGGGGAAU| CAGG |GUUCGAUUCCG|| |GAG| A ||GGGAGCC| UUAGAAAC**

**Triozocera sp.(4) AAGCAGA ||CCGCA| [GUUAAUGGUGAUUGUCGUCGGCU---------------------------] [AUCU-----] [----------------------AAGUGUGACGACGGGAGCCUU] |UGCGG|| -C |GUAUCUUUCAAUUGUCUGC|| ||CUUAUCAACUUUCGAUGGUAGAU| UACAU |GCCUACCAUGGUUGUAACGGG|| UAA ||CGGGGAAU| CAGG |GUUCGAUUCCG|| |GAG| A ||GGGAGCC| UUAGAAAC**

**Blisseoxenos esaki AAGCAGA ||CCGCA| [AUCAAAGGCGGGCAUCGAUACGCACAA-----------------------] [CUUG-----] [-----------------------UUGUGCCCGGUGCACGCCGA] |UGCGG|| –C |GUAUCUUUCAAUUGUCUGC|| ||CUUAUCAACUGUCGAUGGUAGAU| UACAU |GCCUACCAUGGUUGUAACGGG|| UAA ||CGGGGAAU| CAGG |GUUCGAUUCCG|| |GAG| A ||GGGAGCC| UGAGAAAC**

**Corioxenos acucyrtophallus AAGCUGA ||CCGCU| [UCAUACAGUCGUCUGAAUCCAGC---------------------------] [GAAA-----] [-------------------------GCUAUUUUCAGCGAUUGU] |UGCGG|| -C |GUAUCAUUCAAUUGUCUGC|| ||CUUAUCAACUGUCGAUGGUAGAU| UACAU |GCCUACCAUGGUUGUAACGGG|| UAA ||CGGGGAAU| CAGG |GUUUGAUUCCG|| |GAG| A ||GGGAGCC| UGAGAAAC**

**Lychnocolax sp.(2) AAGCAGA ||CCGCA| [AAUAAAAAACAUUGUUCUUAAUUUAAUGAAUU------------------] [UUU------] [-----------AAUUUAAAAAAUUAAGAAAAAAAAACCAUGAA] |UGCGG|| -C |GUAUCUUUCAAUUGUCUGC|| ||CUUAUCAACUUUCGAUGGUAGAU| UACAU |GUCUACCAUGGUUGUAACGGG|| UAA ||CGGGGAAU| CAGG |GUUCGAUUCCG|| |GAG| A ||GGGAGCC| UGAGAAAC**

**Lychnocolax sp.(1) ??????? ||?????| [??????????????????????????????????????????????????] [?????????] [???????????????????????????????????????????] |?????|| ?? |???????UCAAUUGUCUGC|| ||CUUAUCAACUUUCGAUGGUAGAU| UACAU |GUCUACCAUGGUGAUAACGGG|| UAA ||CGGGGAAU| CAGG |GUUCGAUUCCG|| |GAG| A ||GGGAGCC| UGAGAAAC**

**Stichotrema dallatoreanum AAGCAAA ||CUGCA| [AAAAAUUGCCAUACUAUCGUUAAAUUGUrUUUGGAAUCGUGUGCGCGUGU] [-UUAUU---] [-----ACGCGUAGACACCAUCGCAAUAGAAAGUAUUUGGUAAA] |UGCAG|| -U |G??????UCAAUUGUCUGC|| ||CUUAUCAACUGUCGAUGGUAGGU| UACAU |GCCUACCAUGGUUGUAACGGG|| UAA ||CGGGGAAU| CAGG |GUUCGAUUCCG|| |GAG| A ||GGGAGCC| UGAGAAAC**

**Stichotrema sp. AAGCAAA ||CUGCA| [-UGACAAAGUCGGCCGUGUGCGCG--------------------------] [CUUG-----] [-----------------------CGCGUUGCGGCCCGGCGAAU] |UGCAG|| -U |GUGUCUUUCAAUUGUCCGC|| ||CUUAUCAACUGUCGAUGGUAGGU| UACAU |GCCUACCAUGGUUAUAACGGG|| UAA ||CGGGGAAU| CAGG |GUUCGAUUCCG|| |GAG| A ||GGGAGCC| UGAGAAAC**

**Myrmecolax sp.(4) AAGCAGA ||CUGCA| [-UUUAAGCCCGUGCUCGAUAUUAAGUUGUUGAUCGCU-------------] [UUAUC----] [------------GGCGGUGAGUAGCUGGACGAGUCUCGGAGUU] |UGCAG|| -U |GUGUCUUUCAAUUGUCUGC|| ||CUUAUCAACUUUCGAUGGUAGGU| UACAU |GCCUACCAUGGUUGUAACGGG|| UAA ||CGGGGAAU| CAGG |GUUCGAUUCCG|| |GAG| A ||GGGAGCC| UUAGAAAC**

**Myrmecolax sp.(3) AAGCAGA ||CUGCA| [-UUUAGGCCCGUGCUCGACAUCUAGUUGCUGGUCGCU-------------] [UUGUC----] [------------GGCGGUGAGCAGUUAGACGAGUUUCGGUGUU] |UGCAG|| -U |GUGUCUUUCAAUUGUCCGC|| ||CUUAUCAACUGUCGAUGGUAGGU| UACAU |GCCUACCAUGGUUGUAACGGG|| UAA ||CGGGGAAU| CAGG |GUUCGAUUCCG|| |GAG| A ||GGGAGCC| UUAGAAAC**

**Myrmecolax sp.(1) AAGCAGA ||CUGCA| [-UUUAAAGCCGUGCUCAAUGUUUAGCUGCUUUUCGCU-------------] [UAAUC----] [------------GGUGAUAGGCGGUCAUACGAGUGUCGGUGUU] |UGCAG|| -U |GUGUCUUUCAAUUGUCCGC|| ||CUUAUCAACUGUCGAUGGUAGGU| UACAU |GCCUACCAUGGUUGUAACGGG|| UAA ||CGGGGAAU| CAGG |GUUCGAUUCCG|| |GAG| A ||GGGAGCC| UUAGAAAC**

**Myrmecolax sp.(2) AAGCAGA ||CUGCA| [-UUUAGGCCCGUGCUCGACAUCUAGUUGCUGGUCGCU-------------] [UUGUC----] [------------GGCGAUGAGCAGUUAGACGAGUUUCGGUGUU] |UGCAG|| -U |GUGUCUUUCAAUUGUCUGC|| ||CUUAUCAACUGUCGAUGGUAGGU| UACAU |GCCUACCAUGGUUGUAACGGG|| UAA ||CGGGGAAU| CAGG |GUUCGAUUCCG|| |GAG| A ||GGGAGCC| UUAGAAAC**

**Myrmecholax incautus AAGCAGA ||CUGCA| [-UUAGAAGCCGCAUUCAAAUGAUCGUUGUCUGUGGCGCAC----------] [AUCGC----] [-------GUGCGUUUGGAUAAACGAGAAUGAGUGUCUGGUGAU] |UGCAG|| -U |GUGUCUUUCAAUUGUCUGC|| ||CUUAUCAACUGUCGAUGGUAGGU| UACAU |GCCUACCAUGGUUGUAACGGG|| UAA ||CGGGGAAU| CAGG |GUUCGAUUCCG|| |GAG| A ||GGGAGCC| UUAGAAAC**

**Myrmecholax sp.(5) AAGCAGA ||CUGCA| [-UUUAAGCCCGUGCUCGACAUUUAGUUGCUGAUCGCU-------------] [-UUGUU---] [------------GGCGGUGAGCAGUUAUACGAGUUUCGGUGUU] |UGCAG|| -U |GUGUCUUUCAAUUGUCCGC|| ||CUUAUCAACUUUCGAUGGUAGGU| UACAU |GCCUACCAUGGUUGUAACGGG|| UAA ||CGGGGAAU| CAGG |GUUCGAUUCCG|| |GAG| A ||GGGAGCC| UUAGAAAC**

**Caenocholax sp.(1) AAGCAGA ||CCGCA| [-UUUUUAAGCUACGAACGCGUUAAAUCGCCAUCGGUAUUUUUCCAGUU--] [AAAAAAAUG] [GACGAAAAGAACCGAUCGUUUGCGAUU-AGUGUUCGUAGCGAU] |UGCGG|| -C |GUGUCUUUCAAUUGUCUGC|| ||CUUAUCAACUGUCGAUGGUAGGU| UACAU |GCCUACCAUGGUUGUAACGGG|| UAA ||CGGGGAAU| CAGG |GUUCGAUUCCG|| |GAG| A ||GGGAGCC| UUAGAAAC**

**Caenocholax sp.(3) AAGCAGA ||CCGCA| [UUAAAG-GCUGCAAGCAAAGUAAUG--AGAGCG-----------------] [UUUA-----] [------------------CGUUCGAUUUAAUGCUUGCAGUUUG] |UGUGG|| -C |GUAUCUUUCAAUUGUCUGC|| ||CUUAUCAACUGUCGAUGGUAGGU| UACAU |GCCUACCAUGGUUAUAACGGG|| UAA ||CGGGGAAU| CAGG |GUUCGAUUCCG|| |GAG| A ||GGGAGCC| UUAGAAAC**

**Caenocholax sp.(10) AAGCAGA ||CCGCA| [UUAAAA-GCUGCAAACAAAACGAUG--AGAUCG--CU-------------] [CUCG-----] [----------------GGCGUUCGAUUCGAUGUUUGUAGUUUA] |UGUGG|| -C |GUAUCUUUCAAUUGUCUGC|| ||CUUAUCAACUGUCGAUGGUAGGU| UACAU |GCCUACCAUGGUUAUAACGGG|| UAA ||CGGGGAAU| CAGG |GUUCGAUUCCG|| |GAG| A ||GGGAGCC| UUAGAAAC**

**Xenos moutoni AAGCAGA ||CCGCA| [AGAAAAAAUUUAUUUUAU--------------------------------] [UUUAAU---] [--------------------------AUAAAAUGUARUACAAU] |UGCGG|| -C |GCAUCUUUCAAUUGUCUGC|| ||CUUAUCAACUGUCGAUGGUAGAU| UACAU |GUCUACCAUGGUUGUAACGGG|| UAA ||CGGGGAAU| CAGG |GUUCGAUUCCG|| |GAG| A ||GGGAGCC| UGAGAAAC**

**Xenos vesparum AAGCAGA ||CCGCA| [AAAAAAAAUUCAUUUUAUAU------------------------------] [UUU------] [---------------------------AUAUAAAAUAAAUAAA] |UGCGG|| -C |GUAUCUUUCAAUUGUCUGC|| ||CUUAUCAACUGUCGAUGGUAGAU| UACAU |GUCUACCAUGGUUGUAACGGG|| UAA ||CGGGGAAU| CAGG |GUUCGAUUCCG|| |GAG| A ||GGGAGCC| UGAGAAAC**

**Xenos pecki AAGCAGA ||CCGCA| [AAAAAAAAUUUAUCUUA---------------------------------] [UUUA-----] [------------------------------UAAAWUAAAUAAA] |UGCGG|| -C |GUAUCUUUCAAUYGUCUGC|| ||CUGAUCAACU?CCGUUGGUAGAU| UACAU |GUCUACCAUGGUUGUAACGGG|| UAA ||CGGGGAAU| CAGG |GUUCGAUUCCG|| |GAG| A ||GGGAGCC| UGAGAAAC**

**Xenos sp. AAGCAGA ||CCGCA| [AAAAAACAUGUAUUUUAAAAAM----------------------------] [UUUUUA---] [------------------------AUUUUUAAAAUAAAUAAAU] |UGCGG|| -C |GUAUCUUUCAAUUGUCUGC|| ||CUUAUCAACUGUCGAUGGUAGAU| UACAU |GUCUACCAUGGUUGUAACGGG|| UAA ||CGGGGAAU| CAGG |GUUCGAUUCCG|| |GAG| A ||GGGAGCC| UGAGAAAC**

**Paraxenos sp. AAGCAGA ||CCGCA| [AAAAAAAAUUUAUUUUAU--------------------------------] [UUAU-----] [-----------------------------AUAAAAUAAAUAAA] |UGCGG|| -C |GUAUCUUUCAAUUGUCUGC|| ||CUUAUCAACUUUCGAUGGUAGAU| UACAU |GUCUACCAUGGUUGUAACGGG|| UAA ||CGGGGAAU| CAGG |GUUCGAUUCCG|| |GAG| A ||GGGAGCC| UGAGAAAC**

**Pseudoxenos sp. AAGCAGA ||CCGCA| [AAAAAAAUUUU???????????????????????????????????????] [?????????] [???????????????????????????????????????????] |?????|| ?? |???????UCAAUUGUCUGC|| ||CUUAUCAACUGUCGAUGGUAGAU| UACAU |GUCUACCAUGGUUGUAACGGG|| UAA ||CGGGGAAU| CAGG |GUUCGAUUCCG|| |GAG| A ||GGGAGCC| UGAGAAAC**

**Stylops mellitae AAGCAGA ||CCGCA| [AAUAAAAACUUUUUUUUUCGAAU---------------------------] [CUUGA----] [------------------------AUUUGAAGAAAAAUAGUAA] |UGCGG|| GC |GUAUCUUUCAAUUGUCUGC|| ||CUUAUCAACUGUCGAUGGUAGAU| UACAU |GUCUACCAUGGUUGUAACGGG|| UAA ||CGGGGAAU| CAGG |GUUCGAUUCCG|| |GAG| A ||GGGAGCC| UGAGAAAC**

**Tridactylophagus sp. AAGCAGA ||CCGCA| [AAAUUUAAUGGUCCGGGCU-------------------------------] [UUUUC----] [----------------------------AGCUUGUGAUCGAAU] |UGCGG|| -C |GUGUCUUUCAAUUGUCUGC|| ||CUUAUCAACUGUCGAUGGUAGAU| UAUAU |GUCUACCAUGGUUGUAACGGG|| UAA ||CGGGGAAU| CAGG |GUUCGAUUCCG|| |GAG| A ||GGGAGCC| UGAGAAAC**

**Halictophagus calcaratus AAGCAGA ||CCGCA| [AAAUGUGAAUAUUUUU----------------------------------] [UUUUAU---] [---------------------------- GAAAAAAUUCAUU] |UGCGG|| -C |GUAUCUUUCAAUUGUCUGC|| ||UUUAUCAACUGUCGAUGGUAGAU| UAUAU |GUCUACCAUGGUUGUAACGGG|| UAA ||CGGGGAAU| CAGG |GUUCGAUUCCG|| |GAG| A ||GGGAGCC| UGAGAAAC**

**Halictophagus sp.(3) AAGCAGA ||CCGCA| [AAUAUUAAGGUUAUUUUAUUUGUACGUGUGACGAAUAU------------] [GUAAAA---] [------------GUAUCUCAGAACCGUUCGUAAAAUGACCGAU] |UGCGG|| -C |GUAUCUUUCAAUUGUCUGC|| ||CUUAUCAACUUUCGAUGGUAGAU| UAUAU |GUCUACCAUGGUUGUAACGGG|| UAA ||CGGGGAAU| CAGG |GUUCGAUUCCG|| |GAG| w ||GGGAGCC| UGAGAAAC**

**Halictophagus silwoodensis AAGCAGA ||CCGCA| [AGUGAAUGAUUAAUUUU---------------------------------] [UUU------] [-----------------------------AAAAUUUAUCAAAU] |UGCGG|| -C |GUAUCUUUCAAUUGUCUGC|| ||CUUAUCAACUUUCGAUGGUAGAU| UAUAU |GUCUACCAUGGUUGUAACGGG|| UAA ||CGGGGAAU| CAGG |GUUCGAUUCCG|| |GAG| A ||GGGAGCC| UGAGAAAC**

**Halictophagus sp.(2) AAGCAGA ||CCGCA| [AAUAUUAAUUUAGGUCAUUUUAUUUGUACGUCAUUUGGUGU---------] [GUAAAA---] [---------------GCAUCAAAUCGUUCGUAAAAUGAUCGAU] |UGCGG|| -C |GUAUCUUUCAAUUGUCUGC|| ||CUUAUCAACUUUCGAUGGUAGAU| UAUAU |GUCUACCAUGGUUGUAACGGG|| UAA ||CGGGGAAU| CAGG |GUUCGAUUCCG|| |GAG| A ||GGGAGCC| UGAGAAAC**

**Callipharixenos sp. AAGCAGA ||CCGCA| [AAAAUUAAAUAGUUGUUUUUUUUC--------------------------] [UAUU-----] [------------------------GAAAAAAAAGCAAUUAAAU] |UGCGG|| -C |GUAUCUUUCAAUUGUCUGC|| ||CUUAUCAACUGUCGAUGGUAGAU| UAUAU |GUCUACCAUGGUUGUAACGGG|| UAA ||CGGGGAAU| CAGG |GUUCGAUUCCG|| |GAG| A ||GGGAGCC| UGAGAAAC**

**Elenchus sp.(1) AAGCUGA ||CCGCA| [ACAAUAUGCUUUUUAAAGAAAUUUUU------------------------] [GUAAAA---] [----------------------GAAAAUUUUUUCAAUUAGUAU] |UGCGG|| -C |GUAUCUUUCAAUUGUCUGC|| ||CUUAUCAACUUUCGAUgGUAGAU| UACAU |GCCUACCAUGGUUAUAACGGG|| UAA ||CGGAGAAU| CAGG |GUUCGAUUCCG|| |GAG| A ||GGGAGCC| UGAGAAAC**

**Elenchus varleyi AAGCUGA ||CCGCA| [ACAAUAUGCUUUUUAAAAAAAUUUUU------------------------] [AUAAU----] [----------------------GAAAAUUUUUUCAAAUAGUAU] |UGCGG|| -C |GUAUCUUUCAAUUGUCUGC|| ||CUUAUCAACUUUCGAUGGUAGGU| UACAU |GCCUACCAUGGUUAUAACGGG|| UAA ||CGGAGAAU| CAGG |GUUCGAUUCCG|| |GAG| A ||GGGAGCC| UGAGAAAC**

**Elenchus koebelei AAGCUGA ||CCGCA| [ACAAUAUGCUUUUUAAAGAAAUUUUU------------------------] [GUAAAA---] [----------------------GAAAAUUUUUUCAAUUAGUAU] |UGCGG|| -C |GUAUCUUUCAAUUGUCUGC|| ||CUUAUCAACUUUCGAUGGUAGAU| UACAU |GCCUACCAUGGUUAUAACGGG|| UAA ||CGGAGAAU| CAGG |GUUCGAUUCCG|| |GAG| A ||GGGAGCC| UGAGAAAC**

**H316' H339 H339’ H47' H367 H367' H39' H406 RAA10 H406' H441 H441' H500 H505 H511a H511b H505' H511b' H511a' H500'**

**))))))) . (((( .... )))) ... )))))).) ..... (.(((...((((( ... ))))).)))) . )).)))))) ... ((((((((((.((( ...... )))..)))))).)))) ((((((( ................. )))))).) ... (((((( ((( .... (((( ..... (( . ))) )) ....... )))) ))))))**

**Periplaneta fulig' |GGCUACC|| A ||CAUC| CAAG |GAAG|| GCA |GCAGGCGC| GCAAA ||UUACCCACUCCCG| GCA |CGGGGAGGUA|| G |UGACGAAAA| AUA ||ACGAUACGGGACUC| [AUCC--] |GAGGCCCCGUAAUCGG|| ||AAUGAGC| ACACUUUAAAUCCUUUA |ACGAGUAU|| CCA |UUGGAG| |GGC| AAGU |CUGG| UGCCA |GC| A |GCC| |GC| GGUAAUU |CCAG| |CUCCAA|**

**Tamolanica tamolana |GGCUACC|| A ||CAUC| CAAG |GAAG|| GCA |GCAGGCGC| GCAAA ||UUACCCACUCCCG| GCA |CGGGGAGGUA|| G |UGACGAAAA| AUA ||ACGAUACGGGACUC| [AUCC--] |GAGGCCCCGUAAUCGG|| ||AAUGAGU| ACACUUUAAAUCCUUUA |ACGAGUAU|| CCA |UUGGAG| |GGC| AAGU |CUGG| UGCCA |GC| A |GCC| |GC| GGUAAUU |CCAG| |CUCCAA|**

**Reticulitermes fla’ |GGCUACC|| A ||CAUC| CAAG |GAAG|| GCA |GCAGGCGC| GCAAA ||UUACCCACUCCCG| GCA |CGGGGAGGUA|| G |UGACGAAAA| AUA ||ACGAUACGGGACUC| [AUCC--] |GAGGCCCCGUAAUCGG|| ||AAUGAGU| ACACUCUAAAGACUUUA |ACGAGUAU|| CCA |UUGGAG| |GGC| AAGU |CUGG| UGCCA |GC| A |GCC| |GC| GGUAAUU |CCAG| |CUCCAA|**

**Antheraea pernyi |GGCUACC|| A ||CAUC| CAAG |GAAG|| GCA |GCAGGCGC| GCAAA ||UUACCCACUCCCG| GCA |CGGGGAGGUA|| G |UGACGAAAA| AUA ||ACGAUACGGGACUC| [UUAC--] |GAGGCCUCGUAAUCGG|| ||AAUGAGU| ACACUUUAAAUAUUUUA |ACGAGGAA|| CAA |UUGGAG| |GGC| AAGU |CUGG| UGCCA |GC| A |GCC| |GC| GGUAAUU |CCAG| |CUCCAA|**

**Bombyx mori |GGCUACC|| A ||CAUC| CAAG |GAAG|| GCA |GCAGGCGC| GCAAA ||UUACCCACUCCCG| GCA |CGGGGAGGUA|| G |UGACGAAAA| AUA ||ACGAUACGGGACUC| [UUAC--] |GAGGCCUCGUAAUCGG|| ||AAUGAGU| ACACUUUAAAUAUUUUA |ACGAGGAA|| CAA |UUGGAG| |GGC| AAGU |CUGG| UGC-A |GC| A |GCC| |GC| GGUAAUU |CCAG| |CUCCAA|**

**Haematobia irritans |GGCUACC|| A ||CAUC| UAAG |GAAG|| GCA |GCAGGCGC| GUAAA ||UUACCCACUCCCA| GCU |CGGGGAGGUA|| G |UGACGAAAA| AUA ||ACAAUACAGGACUC| [AUAAUA] |GAGGCCCUGUAAUUGG|| ||AAUGAGU| ACACUUUAAAUCCCUUA |ACAAGGAC|| CUA |UUGGAG| |GGC| AAGU |CUGG| UGCCA |GC| A |GCC| |GC| GGUAACU |CCAG| |CUCCAA|**

**Ceratitis capitata |GGCUACC|| A ||CAUC| UAAG |GAAG|| GCA |GCAGGCGC| GUAAA ||UUACCCACUCCCA| GUU |CGGGGAGGUA|| G |UGACGAAAA| AUA ||ACAAUACAGGACUC| [AUAUCC] |GAGGCCCUGUAAUUGG|| ||AAUGAGU| ACACUUUAAAUCCUUUA |ACAAGGAC|| CUA |UUGGAG| |GGC| AAGU |CUGG| UGCCA |GC| A |GCC| |GC| GGUAAUU |CCAG| |CUCCAA|**

**Tribolium castaneum |GGCUACC|| A ||CAUC| CAAG |GAAG|| GCA |GCAGGCGC| GCAAA ||UUACCCACUCCCG| GCA |CGGGGAGGUA|| G |UGACGAAAA| AUA ||ACGAUACGGGACUC| [AUCC--] |GAGGCCCCGUAAUCGG|| ||AAUGAGU| ACACUCUAAACCCUUUA |ACGAGGAU|| CAA |UUGGAG| |GGC| AAGU |CUGG| UGCCA |GC| A |GCC| |GC| GGUAAUU |CCAG| |CUCCAA|**

**Rhagophthalmus ohb' |GGCUACC|| A ||CAUC| CAAG |GAAG|| GCA |GCAGGCGC| GCAAA ||UUACCCACUCCCG| GCA |CGGGGAGGUA|| G |UGACGAAAA| AUA ||ACGAUACGGGACUC| [AUCC--] |GAGGCCCCGUAAUCGG|| ||AAUGAGC| ACACUCUAAACCCUUUA |ACGAGGAU|| CAA |UUGGAG| |GGC| AAGU |CUGG| UGCCA |GC| A |GCC| |GC| GGUAAUU |CCAG| |CUCCAA|**

**Mengenilla australiensis |GGCUACC|| A ||CAUC| UAAG |GAAG|| GCA |GCAGGCAC| GCAAA ||UUACCCACUCCCG| GCA |CGGGGAGGUA|| G |UGACGAUAA| AUA ||ACGAUCCGGAGCUC| [UGAU--] |GAGUUUCCGAAAUCGG|| ||AAUGAGU| ACAAUUUAAAUCCUUUA |ACGAGGAG|| CUA |AUGGAG| |GGC| AAGU |CUGG| UGCCA |GC| A |GCC| |GC| GGUAAUU |CCAG| |CUCCAU|**

**Mengenilla chobauti |GGCUACC|| A ||CAUC| UAAG |GAAG|| GCA |GCAGGCAC| GYAAA ||UUACCCACUCCCG| ??? |??????????|| ? |?????????| ??? ||??????????????| [??????] |GAGUUUCCGAAAUCGG|| ||AAUGAGU| ACAAUUUAAAUCCUUUA |ACGAGGAG|| CUA |AUGGAG| |GGC| AAGU |CUGG| UGCCA |GC| A |GCC| |GC| GGUAAUU |CCAG| |CUCCAU|**

**Triozocera sp.(2) |AGCUACC|| A ||CAUC| UAAG |GAAG|| GCA |GCAGGCAC| GCAAA ||UUACCCACUCCCA| GCA |CGGGGAGGUA|| G |UGACGAUAA| AUA ||ACGAUCCGGGACUC| [UUUA--] |GAGUUUCCGUAAUCGG|| ||AAUGAGU| ACAGUUUAGGUCCUUUA |ACGAGGAA|| CAA |GUGGAG| |GGC| AAGU |CUGG| UGCCA |GC| A |GCC| |GC| GGUAAUU |CCAG| |CUCCAU|**

**Triozocera sp.(3) |AGCUACC|| A ||CAUC| UAAG |GAAG|| GCA |GCAGGCAC| GCAAA ||UUACCCACUCCCA| GCA |CGGGGAGGUA|| G |UGACGAUAA| AUA ||ACGAUCCGGGACUC| [UUUA--] |GAGUUUCCGUAAUCGG|| ||AAUGAGU| ACAGUUUAGGUCCUUUA |ACGAGGAA|| CAA |GUGGAG| |GGC| AAGU |CUGG| UGCCA |GC| A |GCC| |GC| GGUAAUU |CCAG| |CUCCAU|**

**Triozocera sp.(1) |AGCUACC|| A ||CAUC| UAAG |GAAG|| GCA |GCAGGCAC| GCAAA ||UUACCCACUCCCA| GCA |CGGGGAGGUA|| G |UGACGAUAA| AUA ||ACGAUCCGGGACUC| [UUUA--] |GAGUUUCCGUAAUCGG|| ||AAUGAGU| ACAGUUUAAGUCCUUUA |ACGAGGAA|| CAA |GUGGAG| |GGC| AAGU |CUGG| UGCCA |GC| A |GCC| |GC| GGUAAUU |CCAG| |CUCCAU|**

**Triozocera sp.(4) |AGCUACC|| A ||CAUC| UAAG |GAAG|| GCA |GCAGGCAC| GCAAA ||UUACCCACUCCCA| GCA |CGGGGAGGUA|| G |UGACGAUAA| AUA ||ACGAUCCGGGACUC| [UUUA--] |GAGUUUCCGUAAUCGG|| ||AAUGAGU| ACAGUUUAGGUCCUUUA |ACGAGGAA|| CAA |GUGGAG| |GGC| AAGU |CUGG| UGCCA |GC| A |GCC| |GC| GGUAAUU |CCAG| |CUCCAU|**

**Blisseoxenos esaki |GGCUACC|| A ||CAUC| UAAG |GAAG|| GCA |GCAGGCAC| GCAAA ||UUACCCACUCCCG| GCA |CGGGGAGGUA|| G |UGACGAUAA| AUA ||ACGAUCCGGGACUC| [UAAU--] |GAGUUUCCGUAAUCGG|| ||AAUGAGU| ACAGUUUAAAUCCUUUA |ACGAGGAA|| CAA |GUGGAG| |GGC| AAGU |CUGG| UGCCA |GC| A |GCC| |GC| GGUAAUU |CCAG| |CUCCAC|**

**Corioxenos acucyrtophallus |GGCUACC|| A ||CAUC| UAAG |GAAG|| GCA |GCAGGCAC| GCAAA ||UUACCCACUCCCA| GCA |CGGGGAGGUA|| G |UGACGAUAA| AUA ||ACGAUCCGGGACUC| [UAAU--] |GAGUUUCCGUAAUCGG|| ||AAUGAGU| ACAGUUUAAAUCCUUUA |ACGAGGAA|| CAA |GUGGAG| |GGC| AAGU |CUGG| UGCCA |GC| A |GCC| |GC| GGUAACU |CCAG| |CUCCAU|**

**Lychnocolax sp.(2) |GGCUACC|| A ||CAUC| UAAG |GAAG|| GCA |GCAGGCAC| GUAAA ||UUACCCACUCCCA| GUA |CGGGGAGGUA|| G |UGACGAUAA| AUA ||ACGAUCCGGAACUC| [UAAU--] |GAGUUUCCGUAAUCGG|| ||AAUGAGU| ACAAUUUAAAUCCUUUA |ACAAUGAA|| CUA |AUAGAG| |GGC| AAGU |CUGG| UGCCA |GC| A |GCC| |GC| GGUAAUU |CCAG| |CUCUAU|**

**Lychnocolax sp.(1) |GGCUACC|| A ||CAUC| UAAG |GAAG|| GCA |GCAGGCAC| GUAAA ||UUACCCACUCCCA| GUA |CGGGGAGGUA|| G |UGACGAUAA| AUA ||ACGAUCCGGAACUC| [UAAU--] |GAGUUUCCGUAAUCGG|| ||AAUGAGU| ACAAUUUAAAUCCUUUA |ACGAUGAA|| CUA |AUAGAG| |GGC| AAGU |CUGG| UGCCA |GC| A |GCC| |GC| GGUAAUU |CCAG| |CUCUAU|**

**Stichotrema dallatoreanum |GGCUACC|| A ||CAUC| UAAG |GAAG|| GCA |GCAGGCAC| GCAAA ||UUACCCACUCCCG| GCA |CGGGGAGGUA|| G |UGACGAUAA| AUA ||ACGAUCCGGAACUC| [UAUU--] |GAGUUUCCGAAAUCGG|| ||AAUGAGU| ACAAUUUAAAUCCUUUA |ACGAGGAA|| CAA |AUGGAG| |GGC| AAGU |CUGG| UGCCA |GC| A |GCC| |GC| GGUAAUU |CCAG| |CUCCAU|**

**Stichotrema sp. |AGCUACC|| A ||CAUC| UAAG |GAAG|| GCA |GCAGGCAC| GCAAA ||UUACCCACUCCCG| GCA |CGGGGAGGUA|| G |UGACGAUAA| AUA ||ACGAUCCGAAACUC| [UAUU--] |GGGUUUUCGAAAUUGG|| ||AAUGAGU| ACAAUUUAAAUCCUUUA |ACGAGGAA|| CUA |AUGGAG| |GGC| AAGU |CUGG| UGCCA |GC| A |GCC| |GC| GGUAAUU |CCAG| |CUCCAU|**

**Myrmecolax sp.(4) |GGCUACC|| A ||CAUC| UAAG |GAAG|| GCA |GCAGGCAC| GCAAA ||UUACCCACUCCCG| GCA |CGGGGAGGUA|| G |UGACGAUAA| AUA ||ACGAUCCGGAACUC| [UAAU--] |GAGUUUCCGAAAUCGG|| ||AAUGAGU| ACAAUUUAAAUCCUUUA |ACGAGGAA|| CUA |AUGGAG| |GGC| AAGU |CUGG| UGCCA |GC| A |GCC| |GC| GGUAAUU |CCAG| |CUCCAU|**

**Myrmecolax sp.(3) |GGCUACC|| A ||CAUC| UAAG |GAAG|| GCA |GCAGGCAC| GCAAA ||UUACCCACUCCCG| GCA |CGGGGAGGUA|| G |UGACGAUAA| AUA ||ACGAUCCGGAACUC| [UAAU--] |GAGUUUCCGAAAUCGG|| ||AAUGAGU| ACAAUUUAAAUCCUUUA |ACGAGGAA|| CUA |AUGGAG| |GGC| AAGU |CUGG| UGCCA |GC| A |GCC| |GC| GGUAAUU |CCAG| |CUCCAU|**

**Myrmecolax sp.(1) |GGCUACC|| A ||CAUC| UAAG |GAAG|| GCA |GCAGGCAC| GCAAA ||UUACCCACUCCCG| GCA |CGGGGAGGUA|| G |UGACGAUAA| AUA ||ACGAUCCGGAACUC| [AAAU--] |GAGUUUCCGAAAUCGG|| ||AAUGAGU| ACAAUUUAAAUCCUUUA |ACGAGGAA|| CUA |AUGGAG| |GGC| AAGU |CUGG| UGCCA |GC| A |GCC| |GC| GGUAAUU |CCAG| |CUCCAU|**

**Myrmecolax sp.(2) |GGCUACC|| A ||CAUC| UAAG |GAAG|| GCA |GCAGGCAC| GCAAA ||UUACCCACUCCCG| GCA |CGGGGAGGUA|| G |UGACGAUAA| AUA ||ACGAUCCGGAACUC| [UAAU--] |GAGUUUCCGAAAUCGG|| ||AAUGAGU| ACAAUUUAAAUCCUUUA |ACGAGGAA|| CUA |AUGGAG| |GGC| AAGU |CUGG| UGCCA |GC| A |GCC| |GC| GGUAAUU |CCAG| |CUCCAU|**

**Myrmecholax incautus |GGCUACC|| A ||CAUC| UAAG |GAAG|| GCA |GCAGGCAC| GCAAA ||UUACCCACUCCCG| GCA |CGGGGAGGUA|| G |UGACGAUAA| AUA ||ACGAUCCGGAACUC| [UAAU--] |GAGUUUCCGAAAUCGG|| ||AAUGAGU| ACAAUUUAAAUCCUUUA |ACGAGGAA|| CUA |AUGGAG| |GGC| AAGU |CUGG| UGCCA |GC| A |GCC| |GC| GGUAAUU |CCAG| |CUCCAU|**

**Myrmecholax sp.(5) |GGCUACC|| A ||CAUC| UAAG |GAAG|| GCA |GCAGGCAC| ACAAA ||UUACCCACUCCCG| GCA |CGGGGAGGUA|| G |UGACGAUAA| AUA ||ACGAUCCGGAACUC| [UAAU--] |GAGUUUCCGAAAUCGG|| ||AAUGAGU| ACAAUUUAAAUCCUUUA |ACGAGGAA|| CUA |AUGGAG| |GGC| AAGU |CUGG| UGCCA |GC| A |GCC| |GC| GGUAAUU |CCAG| |CUCCAU|**

**Caenocholax sp.(1) |GGCUACC|| A ||CAUC| UAAG |GAAG|| GCA |GCAGGCAC| GCAAA ||UUACCCAAUUCCG| GCA |CGGAGAGGUA|| G |UGACGAUAA| AUA ||ACGAUCCGGAACUC| [GAAU--] |GAGUUUCCGAAAUCGG|| ||AAUGAGU| ACAGCCUAAAUCCUUUA |ACGAGGAA|| CUA |AUGGAG| |GGC| AAGU |CUGG| UGCCA |GC| A |GCC| |GC| GGUAAUU |CCAG| |CUCCAU|**

**Caenocholax sp.(3) |GGCUACC|| A ||CAUC| UAAG |GAAG|| GCA |GCAGGCAC| GCAAA ||UUACCCACUCCCG| GUA |CGGGGAGGUA|| G |UGACGAAAA| AUA ||ACGAUCCGGAACUC| [GAAU--] |GAGUUUCCGAAAUCGG|| ||AAUGAGU| ACAAUUUAAAUACUUUA |ACGAGGAR|| CAA |AUGGAG| |GGC| AAGU |CUGG| UGCCA |GC| A |GCC| |GC| GGUAAUU |CCAG| |CUCCAU|**

**Caenocholax sp.(10) |AGCUACC|| A ||CAUC| UAAG |GAAG|| GCA |GCAGGCAC| GUAAA ||UUACCCAAUCCCA| GCU |CGGGGAGGUA|| G |UGACGAAAA| AUA ||ACAAUCCGGAACUC| [AAAC--] |GAGUCUCCGAAAUUGG|| ||AAUGAGU| ACAGUUUAAAAACUUUA |ACGAGGAG|| CAA |AUGGAG| |GGC| AAGU |CUGG| UGCCA |GC| A |GCC| |GC| GGUAAUU |CCAG| |CUCCAU|**

**Xenos moutoni |GGCUACC|| A ||CAUC| UAAG |GAAG|| GCA |GCAGGCGC| GCAAA ||UUACCCACUCCCG| GCA |CGGGGAGGUA|| G |UGACGAUAA| AUA ||ACGAUCCGGAACUC| [UAAU--] |GAGUUUCCGUAAUCGG|| ||AAUGAGU| ACAAUUUAAAUCCGUUA |ACGAGGAG|| CAA |AUGGAG| |GGC| AAGU |CUGG| UGCCA |GC| A |GCC| |GC| GGUAAUU |CCAG| |CUCCAU|**

**Xenos vesparum |GGCUACC|| A ||CAUC| UAAG |GAAG|| GCA |GCAGGCGC| GCAAA ||UUACCCACUCCCG| GCA |CGGGGAGGUA|| G |UGACGAUAA| AUA ||ACGAUCCGGAACUC| [UAAU--] |GAGUUUCCGUAAUCGG|| ||AAUGAGU| ACAAUUUAAAUCCGUUA |ACGAGGAG|| CAA |AUGGAG| |GGC| AAGU |CUGG| UGCCA |GC| A |GCC| |GC| GGUAAUU |CCAG| |CUCCAU|**

**Xenos pecki |GGCUACC|| A ||CAUC| UAAG |GAAG|| GCA |GCAGGCGC| GCAAA ||UUACCCACUCCCG| GCA |CGGGGAGGUA|| G |UGACGAUAA| AUA ||ACGAUCCGGAACUC| [AAAU--] |GAGUUUCCGUAAUCGG|| ||AAUGAGU| ACAAUUUAAAAGCGUUA |GCAAGUAU|| CAA |AUGGAG| |GGC| AAGU |CUGG| UGCCA |GC| A |GCC| |GC| GGUAAUU |CCAG| |CUCCAU|**

**Xenos sp. |GGCUACC|| A ||CAUC| UAAG |GAAG|| GCA |GCAGGCGC| GCAAA ||UUACCCACUCCCG| GCA |CGGGGAGGUA|| G |UGACGAUAA| AUA ||ACGAUCCGGAACUC| [UAAU--] |GAGUUUCCGUAAUCGG|| ||AAUGAGU| ACAAUUUAAAUCCGUUA |ACGAGGAG|| CUA |AUGGAG| |GGC| AAGU |CUGG| UGCCA |GC| A |GCC| |GC| GGUAAUU |CCAG| |CUCCAU|**

**Paraxenos sp. |GGCUACC|| A ||CAUC| UAAG |GAAG|| GCA |GCAGGCGC| GCAAA ||UUACCCACUCCCG| GCA |CGGGGAGGUA|| G |UGACGAUAA| AUA ||ACGAUCCGGAACUC| [UAAU--] |GAGUUUCCGUAAUCGG|| ||AAUGAGU| ACAAUUUAAAUCCGUUA |ACGAGGAG|| CAA |AUGGAG| |GGC| AAGU |CUGG| UGCCA |GC| A |GCC| |GC| GGUAAUU |CCAG| |CUCCAU|**

**Pseudoxenos sp. |GGCUACC|| A ||CAUC| UAAG |GAAG|| GCA |GCAGGCGC| GCAAA ||UUACCCACUCCCG| GCA |CGGGGAGGUA|| G |UGACGAUAA| AUA ||ACGAUCCGGAACUC| [UAAU--] |GAGUUUCCGUAAUCGG|| ||AAUGAGU| ACAAUUUAAAUCCUUUA |ACAAGGAG|| CAA |AUGGAG| |GGC| AAGU |CUGG| UGCCA |GC| A |GCC| |GC| GGUAAUU |CCAG| |CUCCAU|**

**Stylops mellitae |GGCUACC|| A ||CAUC| UAAG |GAAG|| GCA |GCAGGCAC| GCAAA ||UUACCCACUCCCG| GCA |CGGGGAGGUA|| G |UGACAAUAA| AUA ||ACGAUCCGGAACUC| [UAAU--] |GAGUUUCCGUAAUCGG|| ||AAUGAGU| ACAAUUUAAAUCCUUUA |ACGAGGAA|| CAA |AUGGAG| |GGC| AAGU |CUGG| UGCCA |GC| A |GCC| |GC| GGUAAUU |CCAG| |CUCCAU|**

**Tridactylophagus sp. |GGCUACC|| A ||CAUC| UAAG |GAAG|| GCA |GCAGGCAC| GCAAA ||UUACCCAUUCCCG| GCA |CGGGGAGGUA|| G |UGACGAAAA| AUA ||ACGAUCCGGAACUC| [GAAU--] |GAGUUUCCGUAAUCGG|| ||AAUGAGU| ACAAUUUAAAUCCUUUA |ACGAGGAA|| CUA |AUGGAG| |GGC| AAGU |CUGG| UGCCA |GC| A |GCC| |gC| GgUAAUU |CCAG| |CUCCAU|**

**Halictophagus calcaratus |GGCUACC|| A ||CAUC| UAAG |GAAG|| GCA |GCAGGCAC| GCAAA ||UUACCCAUUCCCG| GCA |CGGGGAGGUA|| G |UGACGAAAA| AUA ||ACGAUCCGGAACUC| [GAAU--] |GAGUUUCCGUAAUCGG|| ||AAUGAGU| ACAAUUUAAAUCCUUUA |ACGAGGAA|| CUA |AUGGAG| |GGC| AAGU |CUGG| UGCCA |GC| A |GCC| |GC| GGUAAUU |CCAG| |CUCCAU|**

**Halictophagus sp.(3) |GGCUACC|| A ||CAUC| UAAG |GAAG|| GCA |GCAGGCAC| GCAAA ||UUACCCAAUCCCG| GCA |CGGGGAGGUA|| G |UGACGAUAA| AUA ||ACGAUCCGGAACUC| [UAAU--] |GAGUUUCCGUAAUCGG|| ||AAUGAGU| ACAAUUUAAAUACUUUA |ACGAGGAG|| CCA |AUGGAG| |GGC| AAGU |CUGG| UGCCA |GC| A |GCC| |GC| GGUAAUU |CCAG| |CUCCAU|**

**Halictophagus silwoodensis |GGCUACC|| A ||CAUC| UAAG |GAAG|| GCA |GCAGGCAC| GCAAA ||UUACCCAUUCCCG| GCA |CGGGGAGGUA|| G |UGACGAAAA| AUA ||ACGAUCCGGAACUC| [GAAU--] |GAGUUUCCGUAAUCGG|| ||AAUGAGU| ACAAUUUAAAUCCUUUA |ACGAGGAA|| CUA |AUGGAG| |GGC| AAGU |CUGG| UGCCA |GC| A |GCC| |GC| GGUAAUU |CCAG| |CUCCAU|**

**Halictophagus sp.(2) |GGCUACC|| A ||CAUC| UAAG |GAAG|| GCA |GCAGGCAC| GCAAA ||UUACCCAAUCCCG| GCA |CGGGGAGGUA|| G |UGACGAUAA| AUA ||ACGAUCCGGAACUC| [UAAU--] |GAGUUUCCGUAAUCGG|| ||AAUGAGU| ACAAUUUAAAUACUUUA |ACGAGGAA|| CCA |AUGGAG| |GGC| AAGU |CUGG| UGCCA |GC| A |GCC| |GC| GGUAAUU |CCAG| |CUCCAU|**

**Callipharixenos sp. |GGCUACC|| A ||CAUC| UAAG |GAAG|| GCA |GCAGGCAC| GCAAA ||UUACCCAUUCCCG| GCA |CGGGGAGGUA|| G |UGACGAAAA| AUA ||ACGAUCCGGAACUC| [GAAU--] |GAGUUUCCGUAAUCGG|| ||AAUGAGU| ACAAUUUAAAUCCUUUA |ACGAGGAA|| CUA |AUGGAG| |GGC| AAGU |CUGG| UGCCA |GC| A |GCC| |GC| GGUAAUU |CCAG| |CUCCAU|**

**Elenchus sp.(1) |GGCUACC|| A ||CAUC| UAAG |GAAG|| GCA |GCAGGCAC| GCAAA ||UUACCCACUCCCA| GCU |CGGGGAGGUA|| G |UGACGAUAA| AUA ||ACGAUACGGAACUC| [UAAU--] |GAGUUUCCGUGAUCGG|| ||AAUGAGU| GUAAUUUAAAUCCUUAU |ACGAGGAG|| CAA |AUGGAG| |GGC| AAGU |CUGG| UGCCA |GC| A |GCC| |GC| GGUAAUU |CCAG| |CUCCAU|**

**Elenchus varleyi |GGCUACC|| A ||CAUC| UAAG |GAAG|| GCA |GCAGGCAC| GCAAA ||UUACCCACUCCCG| GCU |CGGGGAGGUA|| G |UGACGAUAA| AUA ||ACGAUACGGAACUC| [UUAU--] |GAGUUUCCGUGAUCGG|| ||AAUGAGU| GUAAUUUAAAUCCUUAU |ACGAGGAG|| CAA |AUGGAG| |GGC| AAGU |CUGG| UGCCA |GC| A |GCC| |GC| GGUAAUU |CCAG| |CUCCAU|**

**Elenchus koebelei |GGCUACC|| A ||CAUC| UAAG |GAAG|| GCA |GCAGGCAC| GCAAA ||UUACCCACUCCCG| GCU |CGGGGAGGUA|| G |UGACGAUAA| AUA ||ACGAUACGGAACUC| [UAAU--] |GAGUUUCCGUGAUCGG|| ||AAUGAGU| GUAAUUUAAAUCCUUAU |ACGAGGAG|| CAA |AUGGAG| |GGC| AAGU |CUGG| UGCCA |GC| A |GCC| |GC| GGUAAUU |CCAG| |CUCCAU|**

**H27' H567 H570 H575 H577 RAA11 E23-1 E23-2a RAA12 RAA12’ E23-2a' RAA11’ E23-1'**

**. )))))))))) .......... ((( (( ... ( . (((.(((((((( ....... ((((((((((( ((((( ....... ))))) )))))))))))**

**Periplaneta fulig' U |AGCGUAUAUU| AAAGUUGUUG |CGG| |UU| AAA |A| A |GCU-CGUAGUCG| [GACUU--] ||GUGUCCCACGC| |UGCCG| [GUUCACCG-------] [-CCCG--] [----UCGGUGUCUAAC] |UGGCA| [UGCAC----------] |GUGCGG-ACGU||**

**Tamolanica tamolana U |AGCGUAUAUU| AAAGUUGUUG |CGG| |UU| AAA |A| A |GCU-CGUAGUCG| [GACUU--] ||GUGUCCCACGC| |UGCCG| [GUUCACCG-------] [-CCCG--] [----UCGGUGUUUAAC] |UGGCA| [UGCAC----------] |GUGCGG-ACGU||**

**Reticulitermes fla’ U |AGCGUAUAUU| AAAGCUGUUG |CGG| |UU| AAA |A| A |GCU-CGUAGUCG| [GACUU--] ||GUGUCCCACGC| |UGCCG| [GUUCACCG-------] [-CCCG--] [----UCGGUGUUUAAC] |UGGCA| [CGCAC----------] |GUGCGG-ACGU||**

**Antheraea pernyi U |AGCGUAUACU| AAAAUUGUUG |CGG| |UU| AAA |A| A |GCU-CGUAGUUG| [CAUUU--] ||GUGCGCCGCGC| |UGUCG| [GUGCACCG-------] [-CAUU--] [-----AGCGGUGAUAC] |UGACA| [CGU------------] |CUGCGGAGCAU||**

**Bombyx mori U |AGCGUAUACU| AAAAUUGUUG |CGG| |UU| AAA |A| A |GCU-CGUAGUUG| [CAUUU--] ||GUGCGCCGCGC| |UGUCG| [GUGCACCG-------] [-CAUC--] [-----CGCGGUGAUAC] |UGACA| [CGU------------] |UUGCGGAGCAU||**

**Haematobia irritans U |AGCGUAUAUU| AAAGUUGUUG |CGG| |UU| AAA |A| C |GUU-CGUAGUUG| [AACUU--] ||GUGCUUCAUAC| |GGGUA| [GUAUAGCUAU-----] [-AAUU--] [---GUAGUUUGUACUA] |UACCU| [UAUG-----------] |UAUGUAAGCGU||**

**Ceratitis capitata U |AGCGUAUAUU| AAAGUUGUUG |CGG| |UU| AAA |A| C |GUU-CGUAGUUG| [AAUUU--] ||GUGCUUCAUAC| |GGGUA| [GUACAACUAU-----] [-AAUU--] [---GUGGUAUGUACAU] |UACCU| [UAUG-----------] |UAUGUAAGCGU||**

**Tribolium castaneum U |AGCGUAUAUU| AAAGUUGUUG |CGG| |UU| AAA |A| A |GCU-CGUAGUCG| [AAUUU--] ||GUGUCCCGCGC| |CGCCG| [GUUCAUCG-------] [-UUCG--] [-----CGGUGUU-AAC] |UGGCG| [U--------------] |UCGCGGGACGU||**

**Rhagophthalmus ohb' U |AGCGUAUAUU| AAAGUUGUUG |CGG| |UU| AAA |A| A |GCU-CGUAGUCG| [AAUCU--] ||GUGUCCCGCGC| |CGCCG| [GUUCACCG-------] [-CUCG--] [-----CGGUGUUUAAC] |UGGCG| [UGU------------] |CCGCGGGACGU||**

**Mengenilla australiensis U |AGCAUAUAUU| AAAGUUGUUG |CGU| |UU| AAA |A| A |GCU-CGUAGUUA| [AAUUU--] ||GUGUCUUAUGC| |UGUCA| [GUCGCUCGCAA----] [UUUAAU-] [-UUGCGCAGUUUGAUU] |UGGCG| [U--------------] |GCAUGAGAUGU||**

**Mengenilla chobauti U |AGCAUAUAUU| AAAGUUGUUG |CGU| |UU| AAA |A| A |GCU-CGUAGUCG| [AACCU--] ||GUGUCUCGUGC| |UUUCG| [AAUUCCCGCGA----] [UUUUAU-] [----UCGUGUGGUAUU] |UGUUA| [UUUUAUU--------] |GUACGAGAUAU||**

**Triozocera sp.(2) U |AGUAUAUAUU| AAAGUUGUUG |CGU| |UU| AAA |A| A |GCUUCGUAGUUG| [AAUCU--] ||GUAUCCCAUAC| |UGUCA| [AUCGUUCGUAA----] [UUUAAU-] [------UUACGUGGUU] |UGACA| [UUUUUU---------] |GUAUGGGAUAU||**

**Triozocera sp.(3) U |AGUAUAUAUU| AAAGUUGUUG |CGU| |UU| AAA |A| A |GCU-CGUAGUUG| [AAUCU--] ||GUAUCCCAUAC| |UGUCA| [AUCGUUCGUAA----] [UUUAAU-] [------UUGCGUGGUU] |UGACA| [UUUUUU---------] |GUAUGGGAUAU||**

**Triozocera sp.(1) U |AGUAUAUAUU| AAAGUUGUUG |CGU| |UU| AAA |A| A |GCU-CGUAGUUG| [AAUCU--] ||GUAUCCCAUAC| |UGUCA| [AUCGUUCGUAA----] [UUUAAU-] [------UUGCGUGGUU] |UGACA| [UUUUUU---------] |GUAUGGGAUAU||**

**Triozocera sp.(4) U |AGUAUAUAUU| AAAGUUGUUG |CGU| |UU| AAA |A| A |GCU-CGUAGUUG| [AAUCU--] ||GUAUCCCAUAC| |UGUCA| [AUCGUUCGUAA----] [UUUAAU-] [------UUGCGUGGUU] |UGACA| [UUUUUU---------] |GUAUGGGAUAU||**

**Blisseoxenos esaki U |AGUAUAUAUU| AAAGUUGUUG |CGU| |UU| AAA |A| A |GCU-CGUAGUUG| [AAUCU--] ||GUAUCUCAUAC| |UGUCA| [AUCGUUCGUGA----] [UUCAAU-] [------UCACGUGGUU] |UGACA| [UCACUU---------] |GUAUGAGAUAU||**

**Corioxenos acucyrtophallus U |AGUAUAUAUU| AAAGUUGUUG |CGU| |UU| AAA |A| A |GCU-CGUAGUCG| [AAUCU--] ||UGGUCGCAUAC| |UGGCG| [GCAAUUCGUAA----] [UUUAAU-] [-----UUACGUGUUGC] |UGUCA| [UUCUAU---------] |GUAUGCGACAU||**

**Lychnocolax sp.(2) U |AGCAUAUAUU| AAAGUUGUUG |CGU| |UU| AAA |A| A |GCU-CGUAGUUG| [AAUCU--] ||GUAUUUCAUAC| |UGUCA| [AUCGCUCGUAA----] [UUUAAU-] [--UUAUUGAGUUUGAU| [UGACA| [UUUUU----------] |GUAUGAAAUAU||**

**Lychnocolax sp.(1) U |AGCAUAUAUU| AAAGUUGUUG |CGU| |UU| AAA |A| A |GCU-CGUAG???| [???????] ||???????????| |?????| [???????????????] [???????] [????????????????] |?????| [???????????????] |???????????||**

**Stichotrema dallatoreanum U |AGCGUAUAUU| AAAGUUGUUG |CGU| |UU| AAA |A| A |GCU-CGUAGUCG| [AAUCU--] ||GUAUCUUGCGC| |UGUCG| [AUCGCUCGCGA----] [UUCUU--] [--UCGCGUAGUUUAGA] |UGACA| [UUUUGUAU-------] |GCGUAAGAUAU||**

**Stichotrema sp. U |AGCAUAUAUU| AAAGUUGUUG |CGU| |UU| AAA |A| A |GCU-CGUAGUCG| [AAUCU--] ||GUGUCGUGCAC| |UGUUG| |CCCGCUCGCGA----] [UUUUAU-] [--UCGUGCAGUCUGGG] |CGACA| [UUUUAAAGG------] |GUGCGCGAUAU||**

**Myrmecolax sp.(4) U |AGCAUAUAUU| AAAGUUGUUG |CGU| |UU| AAA |A| A |GCU-CGUAGUCG| [AAUCU--] ||AUAUCUUACGC| |UGUCG| |AUCGCUUGUGA----] [UUUGAU-] [--UCGCG-AGUUUGAU] |UGACA| [UUUU-----------] |GUGUAAGAUAU||**

**Myrmecolax sp.(3) U |AGCAUAUAUU| AAAGUUGUUG |CGU| |UU| AAA |A| A |GCU-CGUAGUCG| [AAUCU--] ||AUAUCUUACGC| |UGUCG| |AUCGCUUGUGA----] [UUUGAU-] [--UCGCG-AGUUUGAU] |GGACA| [UUUU-----------] |GUGUAAGAUAU||**

**Myrmecolax sp.(1) U |AGCAUAUAUU| AAAGUUGUUG |CGU| |UU| AAA |A| A |GCU-CGUAGUCG| [AAUCU--] ||AUAUCUUACGC| |UGUCG| |AUCGCUUGUGA----] [UUUAAU-] [--UCGUG-AGUUUGAU] |UGACA| [UUU------------] |GUGUAAGAUAU||**

**Myrmecolax sp.(2) U |AGCAUAUAUU| AAAGUUGUUG |CGU| |UU| AAA |A| A |GCU-CGUAGUCG| [AAUCU--] ||AUAUCUUACGC| |UGUCG| [AUCGCUUGUGA----] [UUUGAU-] [--UCGCG-AGUUUGAU] |GGACA| [UUUU-----------] |GUGUAAGAUAU||**

**Myrmecholax incautus U |AGCAUAUAUU| AAAGUUGUUG |CGN| |UU| AAA |A| A |GCU-CGUAG???| [AAUCU--] ||AUAUCUUGCGC| |UGUUA| [GUCGCUUGCGA----] [UUUUAU-] [--UCGCG-AGUCUGAU] |UGACA| [UUUU-----------] |GCGUAAGAUAU||**

**Myrmecholax sp.(5) U |AGCAUAUAUU| AAAGUUGUUG |CGU| |UU| AAA |A| A |GCU-CGUAGUCG| [AAUCU--] ||AUAUCUUACGC| |UGUCG| [AUCGCUUGUGA----] [UUUGAU-] [--UCGCG-AGUUUGAU] |GGACA| [UUUU-----------] |GUGUAAGAUAU||**

**Caenocholax sp.(1) U |AGCAUAUAUU| AAAGUUGUUG |CGU| |UU| AAA |A| A |GCU-CGUAGUCG| [AAUUU--] ||GUAUCUCGUAC| |UGUUA| [GACGCUCGCGA----] [UUUUAU-] [---UCGUGCAGUUGUU] |UGACA| [UUUUUCAUU------] |GUACGAGAUAU||**

**Caenocholax sp.(3) U |AGCAUAUAUU| AAAGUUGUUG |CGU| |UU| AAA |A| A |GCU-CGUAGUCG| [AACCU--] ||GUAUCUCGUAC| |UAUUA| [AAAACCCGCGA----] [UUUUAU-] [----GCGCGUGGUUUU] |UAAUA| [UCAUAGU--------] |GUACGAGAUAU||**

**Caenocholax sp.(10) U |AGCAUAUAUU| AAAGUUGUUG |CGU| |UU| AAA |A| A |GCU-CGUAGUCG| [AUUUU--] ||GUAUCUCGUAC| |UUUCG| [AAUUCCCGCGA----] [UUUUAU-] [----UCGUGUGGUUUU] |UGUUA| [UUUAAUU--------] |GUACGAGAUAU||**

**Xenos moutoni U |AGCAUAUAUU| AAAGUUGUUG |CGU| |UU| AAA |A| A |GCU-CGUAGUUG| [AAUCU--] ||GUAUCUCAUCA| |UGUCA| [AUCGCUCGUAA----] [UUUAAU-] [-UUAUGUAGUUUGAUU] |UGACA| [UUUU-----------] |GUAUGAGAUAU||**

**Xenos vesparum U |AGCAUAUAUU| AAAGUUGUUG |CGU| |UA| AAA |A| A |GCU-CGUAGUUG| [AAUCU--] ||GUAUCUCAUCA| |UGUCA| [GUCGCUCGUAA----] [UUUAAU-] [-UUAUGUAGUUUGAUU] |UGACA| [UUUU-----------] |GUAUGAGAUAU||**

**Xenos pecki U |AGCAUAUAUA| AAAGUUGUUG |CGU| |UU| AAA |A| A |GCU-CGUAGUCG| [AAUCU--] ||GUAUCUCAUCA| |UGUCA| [GUCGCUCGUAA----] [UUUAAU-] [-UUAUGUAGUUUGAUU] |UGACA| [UUUU-----------] |GUAUGAGAUAU||**

**Xenos sp. U |AGCAUAUAUU| AAAGUUGUUG |CGU| |UU| AAA |A| A |GCU-CGUAGUUG| [AAUUU--] ||GUAUCUCAUCA| |UGUCA| [UUCGCUCGUAA----] [UUUAAU-] [-UUAUGUAGUUUGUUU] |UGACA| [UUUU-----------] |GUAUGAGAUAU||**

**Paraxenos sp. U |AGCAUAUAUU| AAAGUUGUUG |CGU| |UU| AAA |A| A |GCU-CGUAGUUG| [-AUCU--] ||GUAUCUCAUCA| |UGUCA| [AUCGCUCGUAA----] [UUUAAU-] [-UUACGUAGUUUGAUU] |UGGCA| [UCUU-----------] |GUAUGAGAUAU||**

**Pseudoxenos sp. U |AGCAUAUAUU| AAAGUUGUUG |CGU| |UU| AAA |A| A |GCU-CGUAGUUG| [AAUCU--] ||GUAUCUCAUCA| |UGGUU| [AAUCGCUCAUAA---] [UUUAAU-] [-UUAUGUAGUUUGAUU] |UAACA| [UUUU-----------] |GUAUGAGAUAU||**

**Stylops mellitae U |AGCAUAUAUU| AAAGUUGUUG |CGU| |UU| AAA |A| A |GCU-CGUAGUUG| [AAUCU--] ||GUAUCUCAUAC| |UGUCA| [AUCGCUCGUAA----] [UUUAAU-] [UUAUCGUAGUUUGAUU] |UGACA| [UUGU-----------] |GUAUGAGAUAU||**

**Tridactylophagus sp. U |AGCAUAUAUU| AAAAUUGUUG |CGU| |UA| AAA |A| A |GCU-CGUAG-UG| [AAUCU--] ||CUAUCUCAUAC| |UGUCA| [AACGCCCAUAAUAU-] [UUACUU-] [--AUGCGGUAUGUUUU] |UGACA| [AAAUUUUCUAAUG--] |GUAUGAGAUAU||**

**Halictophagus calcaratus U |AGCAUAUAUU| AAAAUUGUUG |CGU| |UU| AAA |A| A |GCU-CGUAGUUG| [GAAUC--] ||CUAUCUCAUAC| |UGUCA| [AACGCUCAUAAUAU-] [UAAUUU-] [--AUGUAGUUUGUUUU] |UGACA| [AAAGUAUAAAACU--] |GUAUGAGAUAU||**

**Halictophagus sp.(3) U |AGCAUAUAUU| AAAUUUGUUG |CGN| |UU| AAA |?| ? |?????????UUG| [AAUCU--] ||GUAUCUCAUAC| |UGUCA| [AUCGCUCAUAA----] [UUUCUAU] [-UUAUGCAGUUUGACU] |UGACA| [AAAUUUAUU------] |UUUAUUG-UAU||**

**Halictophagus silwoodensis U |AGCAUAUAUU| AAAAUUGUUG |CGU| |UU| AAA |A| A |GCU-CGUAGUU?| [AAUCU--] ||CAAUCACAUAC| |UGUCA| [AACGCUCAUAAUA--] [UUUACU-] [--UAUGUGUACGUUUU] |UGACA| [AAAUUUUAAAAUU--] |GUAUGAGAUAU||**

**Halictophagus sp.(2) U |AGCAUAUAUU| AAAUUUGUUG |CGU| |UU| AAA |A| A |GCU-CGUAGUUG| [AAUCU--] ||GUAUCUCAUAC| |UGUCA| [AUCGCUCAUAAU---] [UUCUAU-] [-UUAUGCAGUUUGACU] |UGACA| [AAAUUUAUUUUUAUU] |GUAUGAGAUAU||**

**Callipharixenos sp. U |AGCAUAUAUU| AAAAUUGUUG |CGU| |UU| AAA |A| A |GCU-CGUAGUUG| [AAUCU--] ||CUAUCUCAUAC| |UGUCA| [AACGCUCAUAAUAU-] [UUACUU-] [--AUGUAGUAUGUUUU] |UGACA| [AAAUUUAAAAAUU--] |GUAUGAGAUAU||**

**Elenchus sp.(1) U |AGCAUAUAUU| AAAGUUGUUG |CGU| |UU| AAA |A| A |GCU-CGUAGUCG| [AAUUUU-] ||GUAUCUCGGCG| |UGUCA| [GUCGUUUAGUAAU--] [UUAAAU-] [AUUAUUAAAUUUGAUG] |UGACA| [AUUUUC---------] |GCAUGAGAUAU||**

**Elenchus varleyi U |AGCAUAUAUU| AAAGUUGUUG |CGU| |UU| AAA |?| ? |???????????G| [AAUCU--] ||GUAUCUCGGCG| |UGUCA| [GUCGUUUAGUAAU--] [UUAAAU-] [AUUAUUAAAUUUGAUA] |UGACA| [AAUUUC---------] |GCAUGAGAUAU||**

**Elenchus koebelei U |AGCAUAUAUU| AAAGUUGUUG |CGU| |UU| AAA |?| ? |????????????| [???????] ||???????????| |?????| [???????????????] [???????] [????????????????] |?????| [???????????????] |???????????||**

**RAA13 E23-4 E23-5 RAA14 RAA14’ E23-5 E23-6**

**.......... ((((((( (((( ........ )))) . (((((**

**Periplaneta fulig' [CC--------] ||UGCCGGU| ||GGCC| [CAAGCCGGCGCGUUUCGGGGUCG-----------------------------------------------------] [GAAA----] [-----------------------------------------------CGUCUCCGGGCGCGUUUCGGGGAG] |GGCC|| - ||-----|**

**Tamolanica tamolana [CC--------] ||UGCCGGU| ||GGCC| [CGAGCCGGCGCGGGU-------------------------------------------------------------] [CUCGU---] [------------------------------------------------------ACCCGUCGUCCGGGGAA] |GGCC|| - ||-----|**

**Reticulitermes fla’ [CC--------] ||UGCCGGU| ||GGCC| [CGAGCCGGCGCCCGCGGGUGAGGGGAGCG-----------------------------------------------] [GUAA----] [---------------------------------------CGUUCUCACACC CCGG GUCGUCUCGGGGAG] |GGCC|| - ||-----|**

**Antheraea pernyi [AU--------] ||CGUCGGU| ||GAGC| [CGUCGGU---------------------------------------------------------------------] [CUAAAAA-] [-----------------------------------------------------------------ACGACG] |GUUC|| - ||-----|**

**Bombyx mori [AU--------] ||CGUCGGU| ||GAGC| [CGGCGUU---------------------------------------------------------------------] [CUAAAAA-] [----------------------------------------------------------------AACGCCG] |GUUC|| - ||-----|**

**Haematobia irritans [AU--------] ||UACCGGU| ||GGAG| [UUCUUAUAUGCGUUAAAUA---------------------------------------------------------] [CUUG----] [---------------------------------------------------------UAUUUGCGUAUGUU] |CCUC|| - ||-----|**

**Ceratitis capitata [AU--------] ||UACCGGU| ||GGAG| [UUCUUAUAUAUAAUUAAUA---------------------------------------------------------] [CAAUG---] [--------------------------------------------------------UAUUUUUUAUAUAUU] |CCUC|| - ||-----|**

**Tribolium castaneum [CC--------] ||UGCCGGU| ||GGGC| [UUAGCUC---------------------------------------------------------------------] [GAGA----] [------------------------------------------------------------------GGGCG] |GCCC|| A ||-----|**

**Rhagophthalmus ohb' [CC--------] ||UGCCGGU| ||GGGC| [GCGGCCC---------------------------------------------------------------------] [GCGA----] [------------------------------------------------------------------GGGCG] |GCCC|| - ||-----|**

**Mengenilla australiensis [CG--------] ||UGCUGGU| ||GG-C| [AU-CGUCCGGUCU---------------------------------------------------------------] [UUUUG---] [--------------------------------------------------------------GGGCCGACG] |G-CC|| A ||ACAGG|**

**Mengenilla chobauti [AUA-------] ||UGCUGGU| ||GAAU| [GUUUAGAAAUUGUGUGUUUUAGACGCGCRCGCUGAC----------------------------------------] [UUUUG---] [-------------------------------------GUCGCUGCUUGCUGCAAUUUCAUACGACUUCGAU] |GUUC|| G ||AUACG|**

**Triozocera sp.(2) [GCA-------] ||CGCUGGC| ||GGGU| [UCGGCACGUGUUGUCGGCAAGCCGUGCACAAUAAAGCGACACAUCGCGUUACCUUUGCGCAAGUGUACGUGUUGC-] [ACUAAAU-] [GUGUGAACGCGGACGCUUGCGCAUUAUACGUAACGUGUGUGCCGUGUGCACGCACCGUCGACGCGUCAGUG] |UCCC|| G ||AUGCG|**

**Triozocera sp.(3) [GCA-------] ||CGCUGGC| ||GGGU| [UACCGGUACGUGUCGGUAAGCCGUGUACAAUAAAACGGCACACUGCGUUGCCUUUGCGCAAGUGCACGUUGCGCAC] [UUUGC---] [-----GUGUGAACGGAUGCUUGCGCAUUGUACGGCAACGUGUGUGCCGUGUGCGCACACCGAUCGUCAGUG] |UCCC|| G ||AUGCG|**

**Triozocera sp.(1) [GCA-------] ||CGCUGGC| ||GGGU| [UCGGUACGUAUCGGCAAAUGUGCAUAUUUAAAGCGGCGCCCUgUUGUUUUGUGUAUGCAAACGUCUGAUGC-----] [UUUAU---] [----------------GCACUCAACGUAUGCGUACGCGCAAUGCGUGCCGUGUGCACGCCGAUCGUCGAGG] |UCCC|| G ||AUGCG|**

**Triozocera sp.(4) [GCA-------] ||CGCUGGC| ||GGGU| [UACCGGUACGUGUCGGUAAGCCGUGUACAAUAAAACGGCACACUGCGUUGCCUUUGCGCAAGUGCACGUUGCACAC] [UUUGC---] [-----GUGUGAACGGAUGCUUGCGCAUUGUACGGCAACGUGUGUGCCGUGUGCGCACACCGAUCGUCAGUG] |UCCC|| G ||AUGCG|**

**Blisseoxenos esaki [GCA-------] ||UGCUGGC| ||GGGU| [CGCUAUGCGUUUGGUCGUGCCCGGGCCUUGAAUGUGUCGUCGACGCCGUCAACGCUGCCGCUGUGUGCGGC-----] [UUU-----] [-------------GUCGCGCCGCG ACAGCGUGGUGGCGACGGCGCUGCUCUCGGCGCUGCCAUUCGUGUG] |ACCC|| G ||AUGCG|**

**Corioxenos acucyrtophallus [GCU-------] ||GACUGGC| ||GGG-| [----------------------------------------------------------------------------] [UUCU----] [-----------------------------------------------------------------------] |-CCC|| - ||GUUCG|**

**Lychnocolax sp.(2) [AAA-------] ||UACUGAU| ||GAAU| [UUUAUAAAAAUUAUUUUAAAAAAAAAAUUAAUUAAUUUUUUUUUUUU?????????????????????????????] [????????] [???????????????????????????????????????????????????????????????????????] |????|| ? ||?????|**

**Lychnocolax sp.(1) [??????????] ||???????| ||????| [????????????????????????????????????????????????????????????????????????????] [????????] [???????????????????????????????????????????????????????????????????????] |????|| ? ||?????|**

**Stichotrema dallatoreanum [UAU-------] ||GGUCGGU| ||GGAU| [UUUUUCGAAAACGACUGUUUCUGAmCAUAUUGGACGyGUGU-----------------------------------] [gCAAUAAA] [----------------------------------CAUGCGAAAAAUGUGUCGGUUAUUUGUUUCUUCGGAA] |AUUC|| G ||AUGCG|**

**Stichotrema sp. [ACG-------] ||CGUCGGU| ||GGAU| [UGCUCGCACGGGUGCGCGCGGCCGC---------------------------------------------------] [GAGA----] [-----------------------------------------------GCGGCUCGCGCAUUCGCUGCGGAA] |AUUC|| G ||AUACG|**

**Myrmecolax sp.(4) [CUC-------] ||UGACGGC| ||GGAU| [GUUCGUUGUUCGCUGCGCG---------------------------------------------------------] [AUU-----] [--------------------------------------------------CGCGUAGUGAAUAUUUCGAUA] |AUUC|| G ||AUACG|**

**Myrmecolax sp.(3) [CUC-------] ||UGACGGC| ||GGAU| [GUUCGUUGUUCGCUGCGCG---------------------------------------------------------] [AUU-----] [--------------------------------------------------CGCGUAGUGAAUAUUUCGAUA] |AUUC|| G ||AUACG|**

**Myrmecolax sp.(1) [UUC-------] ||UGACGGC| ||GGAU| [AUUCGAUGUUCAUUGCGCA---------------------------------------------------------] [AUU-----] [--------------------------------------------------CGCGUAAAUAACAUGUCGAUA] |AUUC|| G ||AUACG|**

**Myrmecolax sp.(2) [CUC-------] ||UGACGGC| ||GGAU| [GUUCGUUGUUCGCUGCGCG---------------------------------------------------------] [AUU-----] [--------------------------------------------------CGCGUAGUGAAUAUUUCGAUA] |AUUC|| G ||AUACG|**

**Myrmecholax incautus [AUG-------] ||UGACGGC| ||GGAU| [GUUCGUCGCUUGGAAUCGGUU-------------------------------------------------------] [UCUC----] [------------------------------------------------AAUCGCUUUCCGAGCGCACGAUA] |AUUC|| G ||AUACG|**

**Myrmecholax sp.(5) [CUC-------] ||UGACGGC| ||GGAU| [GUUCGUUGUUCGCUGCGCG---------------------------------------------------------] [AUU-----] [--------------------------------------------------CGCGCUGUGAAUAUUUCGAUA] |AUUC|| G ||AUACG|**

**Caenocholax sp.(1) [CAA-------] ||UGCUGGU| ||GGAU| [AUUUAGGAUUUGCGUACGUGCGAUCGC-------------------------------------------------] [UUCGC---] [--------------------------------------------GCGUUCGUACGUUUGUCGAAUUCGAU-] |GUUC|| G ||AUACG|**

**Caenocholax sp.(3) [A---------] ||UGCUGGU| ||GGAU| [GCUCAGAAAUUGUAUGCUAUAGAUGUGC------------------------------------------------] [AUUU----] [---------------------------------------GCUCG-UUUCU---AUACUUAUGAUUUCGAU-] |AUUC|| G ||AUACG|**

**Caenocholax sp.(10) [AUA-------] ||GACUGGU| ||GGAU| [GUUUAGAAAUCGUUUGC-UUAUAGACGACGGAC-------------------------------------------] [AUCU----] [---------------------------------------GUUCGC--GCU---AUUCAU-CGAUUUCGAU-] |AUUC|| A ||AUACG|**

**Xenos moutoni [AAA-------] ||UACUGGU| ||GGAU| [UUUUUCAAAAUUGAAUAUUAUUUUUUUAUAUUUAU-----------------------------------------] [UUCAUU--] [----------------------------------AUAAAUAUAAAUUAAAUUGAUAAUGUUCAAUUUUAAA] |AUUC|| G ||AUAUA|**

**Xenos vesparum [AAA-------] ||UACUAGU| ||GGAU| [UUUUUCAAAAUUGAAUUUUAUUUAUUUAUAUUUUGGUUUUUUUUUU------------------------------] [UUUUUUC-] [--------------------------AAAAAAAAAAUCAAUAUAAAUUAUAUAGUAAUUUUCGAUUUUAAA] |AUUC|| G ||AUAUA|**

**Xenos pecki [AAA-------] ||UACUGGU| ||GGAU| [UUUU-CAAAAUUGAAAUUUAUUUUUUUAUAUUUAU-----------------------------------------] [UUUAUU--] [----------------------------------AUAAAUAUAAAUUAAAU-GAUAAUUUUCAAUUUUAAA] |AUUC|| G ||AUAUA|**

**Xenos sp. [AAA-------] ||UACUGGC| ||GGAU| [UUUU-AAAAAUUGAAAAUUAUUAAAUUUAUUUAAAUAUUA------------------------------------] [UAUUGU--] [----AAUUCUAUUUGUGAAUUUCAAUUAAUAUUUUAAUAUAAAUUGUUAUAAAAUAAUAAUCAAUUUUAAA] |AUUC|| G ||AUGCA|**

**Paraxenos sp. [AAA-------] ||UACUGGU| ||GGAU| [UUUUCAAAAUUGAAUAUUAUUUUUUAUAUUUUUU------------------------------------------] [UUUC----] [-----------------------------------AAAAAAUAUAAAUAAAUGGUAAUUUUCAAUUUUAAA] |AUUC|| G ||AUAUA|**

**Pseudoxenos sp. [AAA-------] ||UACUGGU| ||GGAU| [UUUU-CGAAAUUGAAUUUUUAUAUUUAUAUUUU-------------------------------------------] [UUUUAAU-] [-----------------------------------------AAAAUAUAAAUUGAAUUUUCAAUUUUUAAA] |AUUC|| G ||AUAUA|**

**Stylops mellitae [AAA-------] ||UGCUGGU| ||GGAU| [UUUUUUAAAAUCGAUUUCGUUAAAAAAAUU----------------------------------------------] [UUUUUU--] [-----------------------------------------AAUUUUUUUUUUUUUGUUAUCgAUUCUAAA] |AUCC|| G ||AUACU|**

**Tridactylophagus sp. [GCA-------] ||AACUGGU| ||GGAU| [UUUUAAAAAAUUUGUACCGUCGCGGCGAGUUUG-------------------------------------------] [CUUGC---] [-------------------------------------------CAAGUUCGUUGCACGGCGAAAUUUUUAA] |AUUC|| G ||AUGCG|**

**Halictophagus calcaratus [GUG-------] ||AACUGGU| ||GGAU| [UUGACGAGGUCCUGUAAAAAAGUUAUU-------------------------------------------------] [CAAUA---] [------------------------------------------------GAUAACCUUUUAUAGGCCUCUAA] |AUUC|| G ||AUGUU|**

**Halictophagus sp.(3) [GAGAUAUGAC] ||UACUGGC| ||GGAU| [UUUUAUGAAAUUAUAUACUAGAUAAUUUGUGAUUAAAUUG------------------------------------] [UUAU----] [----------------------------UAAUUUAAUCGUUAAGCAAAUUUAUCGAAUAUAUAAUUUUAAA] |AUUC|| G ||AUAUU|**

**Halictophagus silwoodensis [GUG-------] ||AACUGGU| ||GGAU| [UUCAAAAAAAAUUUUCUAAAUUGUUUCGCAUAUUUAUG--------------------------------------] [GUAACA--] [--------------------------------------CAUAAAUAUCGUGCAAUUUGUAAAUUUUUUUUA] |AUUC|| G ||AUACG|**

**Halictophagus sp.(2) [GAC-------] ||UACUGGC| ||GGAU| [UUUAUAAAAUUAUAUACAAAAANAUUGUGU----------------------------------------------] [CGUCAAAA] [--------------------------------------------GCACAAUUUUGCAUAUAUAAUUUUAAA] |AUUC|| G ||AUAUC|**

**Callipharixenos sp. [GCA-------] ||AACUGGU| ||GGAU| [UUUCAAAAAAUUUUCAUUCUAGUUCGAACGAAUGGACGGCGGCAGCGGUAAAGACAACGGCCGCCGCGUUGGCG--] [AUyAU---] [---------CGUAAmCGUCGUGUGUCGUUGUCGACGUUGUCGUCAUAAUUUCUCGACUUGUGAAAUUUUUA] |AUUC|| G ||AUAUU|**

**Elenchus sp.(1) [AAA-------] ||UAUUGGU| ||GGAU| [UUUU-AAGUAAUCAAAAUAAAAAAU---------------------------------------------------] [UUUC----] [---------------------------------------------------AUUUUUUUUUUGAUUAUAAA] |AUUC|| G ||AUACA|**

**Elenchus varleyi [AAA-------] ||UAUUGGU| ||GGAU| [UUUUUAAGUAAUUAUAUUUUAUAAAAGAAA----------------------------------------------] [UUUUUUU-] [--------------------------------------------UUUUUUUUAUAUUAAUAUAAUUAUUAA] |AUUC|| G ||AUACA|**

**Elenchus koebelei [??????????] ||???????| ||????| [????????????????????????????????????????????????????????????????????????????] [????????] [???????????????????????????????????????????????????????????????????????] |????|| ? ||?????|**

**RAA15**

**............**

**Periplaneta fulig' [--------------------------------------------------------------------------------------------------------------------------------------------------------------------------------------] [------------]**

**Tamolanica tamolana [--------------------------------------------------------------------------------------------------------------------------------------------------------------------------------------] [------------]**

**Reticulitermes fla’ [--------------------------------------------------------------------------------------------------------------------------------------------------------------------------------------] [------------]**

**Antheraea pernyi [--------------------------------------------------------------------------------------------------------------------------------------------------------------------------------------] [------------]**

**Bombyx mori [--------------------------------------------------------------------------------------------------------------------------------------------------------------------------------------] [------------]**

**Haematobia irritans [--------------------------------------------------------------------------------------------------------------------------------------------------------------------------------------] [------------]**

**Ceratitis capitata [--------------------------------------------------------------------------------------------------------------------------------------------------------------------------------------] [------------]**

**Tribolium castaneum [--------------------------------------------------------------------------------------------------------------------------------------------------------------------------------------] [------------]**

**Rhagophthalmus ohb' [--------------------------------------------------------------------------------------------------------------------------------------------------------------------------------------] [------------]**

**Mengenilla australiensis [-AAAGCGUAACUCCUGCGUGG-----------------------------------------------------------------------------------------------------------------------------------------------------------------] [-UUUAC------]**

**Mengenilla chobauti [-AW???????????????????????????????????????????????????????????????????????????????????????????????????????????????????????????????????????????????????????????????????????????????????] [????????????]**

**Triozocera sp.(2) [-ACGGAUUCGA---------------------------------------------------------------------------------------------------------------------------------------------------------------------------] [UUAGCU------]**

**Triozocera sp.(3) [-ACGGAUUCGA---------------------------------------------------------------------------------------------------------------------------------------------------------------------------] [UUAGCU------]**

**Triozocera sp.(1) [-GCGGGUUCGA---------------------------------------------------------------------------------------------------------------------------------------------------------------------------] [UUAAUU------]**

**Triozocera sp.(4) [-ACGGAUUCGA---------------------------------------------------------------------------------------------------------------------------------------------------------------------------] [UUAGCU------]**

**Blisseoxenos esaki [-UCGUGUUCGA---------------------------------------------------------------------------------------------------------------------------------------------------------------------------] [UUCACU------]**

**Corioxenos acucyrtophallus [UACC----------------------------------------------------------------------------------------------------------------------------------------------------------------------------------] [UAUAC-------]**

**Lychnocolax sp.(2) [??????????????????????????????????????????????????????????????????????????????????????????????????????????????????????????????????????????????????????????????????????????????????????] [????????????]**

**Lychnocolax sp.(1) [??????????????????????????????????????????????????????????????????????????????????????????????????????????????????????????????????????????????????????????????????????????????????????] [????????????]**

**Stichotrema dallatoreanum [-GUUAAUUAwGCUUUCGUUUUCGCGUAACGGCAAACGAAAUUUGUGAAAUGGAUUUUACAUUCGCGGCUGUAAAAACGAUUCGCGUU-----------------------------------------------------------------------------------------------] [UUGUC-------]**

**Stichotrema sp. [-AACGGUCUCGUAAACAAAUUGAAGUCCGCACGGCGCCAAAGGGAGUCGAAUUCGUGCAGCAUCGGCGG-----------------------------------------------------------------------------------------------------------------] [CUUG--------]**

**Myrmecolax sp.(4) [-AUUAGCUUUUGCUUAUUUGGUUUUGAAGCAGUUCGAUGCGAUUAGCAAGCGGUCGUUAAGACGGACUAGUGAGCUGACGGACCGUAC----------------------------------------------------------------------------------------------] [AUC---------]**

**Myrmecolax sp.(3) [-AUUAGCUUUUGAUUAUUUGGUUUUAAGGUAGUUCGAUGCGAUCGGCAAACGGUCGUGAAGGCAGGCUAGUGAACUGACGAGCCGUAC----------------------------------------------------------------------------------------------] [AUC---------]**

**Myrmecolax sp.(1) [-AUUAGCUUUUACUUAAUUGGUUUUAAAGUGGUUCGAUGUGAUUAGCAAUCGGUCGUAAAGACGGGCUAGUAAACUGAUGAGCCGCGCGUGCGU----------------------------------------------------------------------------------------] [GUAAA-------]**

**Myrmecolax sp.(2) [-AUUAGCUUUUGAUUAUUUGGUUUUAAGGUAGUUCGACGCGAUCGGCAAGCGGUCGUGAAGGCAGACURGUGAACUGACGAGCCGUAC----------------------------------------------------------------------------------------------] [-AUC--------]**

**Myrmecholax incautus [-GUUGACGUUUGUCGAUAAGUUUCAUUGUGAUUGAAUrCAmUCAGCGmGCAmUCGUGAAGUCGAAGCGUGGAACCGAUGAGUUAGAAGAGGCUCGCGCG-----------------------------------------------------------------------------------] [CGUG--------]**

**Myrmecholax sp.(5) [-AUUAGCUUUUGCUUAUUUGGUUUUGAAGUAGUUCGAUGCGAUUAGCAAGCGGUCGUAAAGACGGACUAGUGAACUGACGGGGGCGCCG---------------------------------------------------------------------------------------------] [UACAU-------]**

**Caenocholax sp.(1) [-AUGGAUUUUUUACAAAUUUCGCAAACGGUCAAUCGGUAAAACAAUCGAUGACGAACACGAACAAGAACA----------------------------------------------------------------------------------------------------------------] [GCAG--------]**

**Caenocholax sp.(3) [-AUGAGUUU---AUUUUCUUUCGC--------------------------------------------------------------------------------------------------------------------------------------------------------------] [UUCUG-------]**

**Caenocholax sp.(10) [-AUGGGUUUAGGAUUUAAUUUCGCAGCAGUGUAAAAUGU-----------------------------------------------------------------------------------------------------------------------------------------------] [UUCAAUUACGUU]**

**Xenos moutoni [-AUAAUUUUUUAAUGAAUAAGUUUUUUAAAAAAAAAAUCAAAUUUUUUUUUUUUUUUUAUUAAAAGAUAAUGUUUUUAA-------------------------------------------------------------------------------------------------------] [UUUUU-------]**

**Xenos vesparum [-AUAAUUUUUAAUCAAAAAAGUUUAUCGAAAAUAAAUGUUAUUUUUUAAUAAAAAAUUUUU-------------------------------------------------------------------------------------------------------------------------] [UUA---------]**

**Xenos pecki [-AUAAUUUUUAAUUAAAAAAGUUUAUUUUUUAAUUAAAAAAAUAUUUUUUUAAUACUUGAUUUUUUU-------------------------------------------------------------------------------------------------------------------] [UUAA--------]**

**Xenos sp. [-AUAAUUUUUUAAUUAAUAAAAUUUUAAAAAAAAAAUUAAUAGAAAAAUUUUUUUUUAAACAAUAAAUUUU---------------------------------------------------------------------------------------------------------------] [GUUUA-------]**

**Paraxenos sp. [-AUGAUUUUUAAUUAAAAAAGUUUAAUAAGAAUAAUUUUUUUAAUAAAAAAUUGGAUUUUUGGUUUUUUCAAUUUUUUUUUU????????????????????????????????????????????????????????????????????????????????????????????????????] [????????????]**

**Pseudoxenos sp. [-AUAAUUUUUAAUAAAAAAAGUUUAUUAAUAAUUGUUUUAAAUAUUGUUUUUUU--------------------------------------------------------------------------------------------------------------------------------] [UUUAAUU-----]**

**Stylops mellitae [-GUUAAUUUGAAUUCUAAGAGGAAAUUAGUUUCUUAAUGAAAAAACAAUCGAAAAAAUUAUGU-----------------------------------------------------------------------------------------------------------------------] [-UUUA-------]**

**Tridactylophagus sp. [-GAUCGGUCGGCUUGUUUCUGGAGUCGUCCGAACGCGAACCGAAAUUUCGGCAACGUUAAGUUCCAUUCGACGGAC----------------------------------------------------------------------------------------------------------] [AUCUC-------]**

**Halictophagus calcaratus [-AAUGUUAAACUGUUUCUAGAAUCGAAAAUG GUGCGAUAAAAAUGAAAAAAAACUAUAAAUUGUUUCGUUACGAAUAGAAA----------------------------------------------------------------------------------------------------] [UUU---------]**

**Halictophagus sp.(3) [-GUGGCAAACGUUUAAAAGUGCGAAUUUUAUAUAAUUACAUAGUUAUUUCCUUAUGUUACAUCUGUGUGAUAAAUGUUGAUUGAGUUUUUUUU-----------------------------------------------------------------------------------------] [-UCAUU------]**

**Halictophagus silwoodensis [-UUUGAUGUAACGUUUCUAGGGUUAUUUUUCUUUUUUAAAAAAAAAUGAAAAAAA???????????????????????????????????????????????????????????????????????????????????????????????????????????????????????????????] [????????????]**

**Halictophagus sp.(2) [-GCGAUUAACGUUAAAAGUGAUUCAAAAGAAUUUAAUUCAGUUUACUUCUUUGAAAACAAGUCUGUGUCCGGG?????????????????????????????????????????????????????????????????????????????????????????????????????????????] [????????????]**

**Callipharixenos sp. [-GUUGUUUAAUCGUUUCmAGAGUCAUAUUGUUCAUGAAUUUAGGGCGGCGCUUAACGUUAAGUUUUACUUAUCGACUUUGGCGAUA------------------------------------------------------------------------------------------------] [UUUAUU------]**

**Elenchus sp.(1) [-AAAUAAUUUUUUUUAUUAAUAUAAAUUAUUUAUAGAAAAAAUGAAUUUUUUUUAAUGGAAAAAUUUAAAUUUAUUUUUAAGUUUAAUUUUUUCCUUCUAAAUUUGUUAAAAAAAUAUUAAUUUUUUUAACAUAAAAAAAUUUAGAUAUUUAUAAAAUUUUAUUUAAUCAAUAGUGGAGAAA] [GUUU--------]**

**Elenchus varleyi [-AAAUAAAUUUUUUUUAUUAAUAUAAAUUAUUUAUAGAAAAAGUGAAUUUUUUUUAAAUCGAAAAUUAUUUAUAAUUUUUGUCUAUUAAAAAAUUUAUUUUAUUUUUAGAUAUUUAAUAAAUUUUUACUCAAAUCAAUAAUAAUAUUAUAAUGGUAAUUAAAA-------------------] [UUAAUU------]**

**Elenchus koebelei [??????????????????????????????????????????????????????????????????????????????????????????????????????????????????????????????????????????????????????????????????????????????????????] [????????????]**

**RAA15' E23-6 RAA16 E23-7 RAA17 RAA17’**

**)).))) ................. . ((((( ........**

**Periplaneta fulig' [----------------------------------------------------------------------------------------------------------------------------------] |------|| [--------UACUCAUAC] - ||CCGUC| [GCGGUGCUCU----------------------------------------] [UAAC----] [-----------------------------------------CGAGUGUCUC]**

**Tamolanica tamolana [----------------------------------------------------------------------------------------------------------------------------------] |------|| [--------UA-UCAUAC] - ||CCGUC| [GCGGUGCUCU----------------------------------------] [UAAC----] [-----------------------------------------CGAGUGUCUC]**

**Reticulitermes fla’ [----------------------------------------------------------------------------------------------------------------------------------] |------|| [--------UACACAAUC] - ||CCGUC| [GCGGUGCUCU----------------------------------------] [UGAC----] [-----------------------------------------CGAGUGUCUC]**

**Antheraea pernyi [----------------------------------------------------------------------------------------------------------------------------------] |------|| [-----AAUAUCAAAAUC] - ||CUAUC| [GCGGUGCUCU----------------------------------------] [UCGG----] [-----------------------------------------UGAGUGUCGA]**

**Bombyx mori [----------------------------------------------------------------------------------------------------------------------------------] |------|| [-----AAUAUCAAAAUC] - ||CUAUC| [GCGGUGCUCU----------------------------------------] [UCGG----] [-----------------------------------------UGAGUGUCGA]**

**Haematobia irritans [----------------------------------------------------------------------------------------------------------------------------------] |------|| [-------CUAUUUAAAC] - ||CUGCU| [UCAGUGCUCU----------------------------------------] [UCAU----] [-----------------------------------------CGAGUGUUGU]**

**Ceratitis capitata [----------------------------------------------------------------------------------------------------------------------------------] |------|| [-------CUAUUUAAAC] - ||CUGCU| [UCAGUGCUCU----------------------------------------] [UCAU----] [-----------------------------------------CGAGUGUUGU]**

**Tribolium castaneum [----------------------------------------------------------------------------------------------------------------------------------] |------|| [---------ACUCAAUC] - ||CCGCC| [GCGGUGCUCU----------------------------------------] [UCAC----] [-----------------------------------------UGAGUGUCGA]**

**Rhagophthalmus ohb' [----------------------------------------------------------------------------------------------------------------------------------] |------|| [------AACUACGAAUC] - ||CCGCC| [GCGGUGCUCU----------------------------------------] [UCAC----] [-----------------------------------------UGAGUGUCGA]**

**Mengenilla australiensis [--------------------------------------------------------------------------------------------------------------CCGCGUAGUUUUUGCGCAUU] |CG-UGU|| [-----------------] U ||CUGAU| [ACCUGGUUCGUGCAGUCGGGUCGUCGACGAUAGCAGGUUC----------] [UAC-----] [--------GAAUUUGCUAUCGCCGCAUUCGAACGCCGAACAUCAAA-CGCU]**

**Mengenilla chobauti [??????????????????????????????????????????????????????????????????????????????????????????????????????????????????????????????????] |??????|| [?????????????????] ? ||?????| [??????????????????????????????????????????????????] [????????] [???????????????????????????????????????????????????]**

**Triozocera sp.(2) [----------------------------------------------------------------------------------------------------------------------UCGAAUUCGUAU] |CG-CAU|| [-----------------] U ||CUGAU| [UGGAAACUCACUCUCGCGCAUUAGUGCAUCCGAUCGUCUCGUGUGCCGCG] [CAUCGU--] [CGCGUGCACACGUGACACGGUGCACCGUGUGCGAUUGUGUGAAAUCUCUCU]**

**Triozocera sp.(3) [----------------------------------------------------------------------------------------------------------------------UCGAACUCGUAU] |CG-CAU|| [-----------------] U ||CUGAU| [-CGAAACUCGUGCCCGAUCGUCUCGUGUGCCGC-----------------] [UUCGUU--] [---------------------GUGUGCACACGUGACCGUGCACAAAACUCU]**

**Triozocera sp.(1) [----------------------------------------------------------------------------------------------------------------------UCGAAUUCGUUU] |CG-UAU|| [-----------------] U ||CUGAU| [-CGAAACCUGUGCACGUCUGUCUAUGUGUGCCGC----------------] [UUCGUU--] [------------------GCGCGCUCACGAACGGACUGUGCGCUAAACUCU]**

**Triozocera sp.(4) [----------------------------------------------------------------------------------------------------------------------UCGAAUUCGUAU] |CG-CAU|| [-----------------] U ||CUGAU| [-CGAAACUCGUGCCCGAUCGUCUCGUGUGCCGC-----------------] [UUCGUU--] [-------------------GUGCGCAnnnnnnnnnnnnUGCACARAASUCA]**

**Blisseoxenos esaki [----------------------------------------------------------------------------------------------------------------------UCGUGCACGUCA] |CG-CAU|| [-----------------] U ||CUGAU| [-CGAGGAUCGUGUGCACGCACGCGCCCGCUGUCUAACGCGCG--------] [UUCAUU--] [----------AAUGCGCAGACAUGCGCGGCGAGCGUGCGCGCGACUCUAAC]**

**Corioxenos acucyrtophallus [-------------------------------------------------------------------------------------------------------------------------------GGU] |CG-AAU|| [----------------U] U ||CUGAC| [AUGAACGGCGAC--------------------------------------] [UCAA----] [-----------------------------------------GUCACCGUCU]**

**Lychnocolax sp.(2) [??????????????????????????????????????????????????????????????????????????????????????????????????????????????????????????????????] |??????|| [?????????????????] ? ||?????| [??????????????????????????????????????????????????] [????????] [???????????????????????????????????????????????????]**

**Lychnocolax sp.(1) [??????????????????????????????????????????????????????????????????????????????????????????????????????????????????????????????????] |??????|| [?????????????????] ? ||?????| [??????????????????????????????????????????????????] [????????] [???????????????????????????????????????????????????]**

**Stichotrema dallatoreanum [--------------------------------------------------AACUGCGUUUCGUUGGUUGUGUUUGUUUUCAAUUUCCAGUUCGUUUUUAGUUUCGUUAAGUGAUUUCGGUGAAUUUUAAA] |CG-UUU|| [-----------------] U ||CUGAA| [UGGCUCAAArUGAAUGU---------------------------------] [GAAAAU--] [------------------------------GUGUUCGCUUUGUUUUGCUCA]**

**Stichotrema sp. [----------------------------------------------------------------------------CCGUUGUGCUGCCUCGGCUCUCUGUCGGGCGCGACCUCAAGCGGGCACCGUUGC] |CG-UAU|| [-----------------] U ||CUGAU| [CGACGACGGCGCGGCAGUGUGUGC--------------------------] [GAAC----] [----------------------------GCGCGCACGCCCGCCUCGCUCGA]**

**Myrmecolax sp.(4) [-------------------------------------------GUGCGCGUCUGUCAUCAAGGUUCCUAGUGUGUUGGCCGCGCGCGUCGCGUUGCAAAAUUGCUUUAGAACAAAGUCGAAAGUUUUUAU] |CG-UAU|| [-----------------] U ||CUGAU| [UGGCGUUCACGCUGGUGAAUGCGCGCGGCUUU------------------] [CUAGCUU-] [-----------------------GAGGCUGUGCGUGCAGCAUGUUCGCCCA]**

**Myrmecolax sp.(3) [-------------------------------------------GUGCGCGUUUGUCAUCAAGGUUCCUAGUGUGUUGGCUGCGCGCUGCGUGUUUCAAAACUGCUUUAGAACAAAGUCGAAAGUUUUUAU] |CG-UAU|| [-----------------] U ||CUGAU| [UGGCGUUCGCGUUGGUGAAUGCGCGCAGUUUU------------------] [CUAGUUU-] [-----------------------GAAGCUGUGCGUGCAGCAUGUUCGCCCA]**

**Myrmecolax sp.(1) [-------------------------------------GCGCAUUGCGUGCUUGUCGUUAAGGUUUUUAGUGUUGUUGGCUGUGCGCGUCGCGUUGCAAAAUUGCUUUAGAACAAAGUCAAAAGUUUUUAU] |CG-UAU|| [-----------------] U ||CUAAU| [UGGUGUUCGUGCUGGCGUAUACGCAUGAUUUU------------------] [CAAGGUU-] [---------------------UGGAAAUUGUGUGUGCAGCGCGUUCGCUUA]**

**Myrmecolax sp.(2) [-------------------------------------------GUGCGCGUUUGUCAUCAAGGUUCCUAGUGUGUUGGCUGCGCGCUGCGCGUUGCAAAACUGCUUUAGAACAAAGUCGAAAGUUUUUAU] |CG-UAU|| [-----------------] U ||CUGAU| [UGGCGUUCGCGCUGGUGAAUGCGCGCAGUUUU------------------] [CUAGUUU-] [-----------------------GAAGCUGUGCGUGCAGCAUGUUCGCCCA]**

**Myrmecholax incautus [----------------------------CGCGUGUUGUUUUGUCUGCUUGUCGCUAAAUGGUUUCUAGUUUUGAUGAUUGUGUGCUGCAUUGUUUUCAUUAAUUGCAUUGGAAUUAGGCGAAGGUUUUGU] |CG-UAU|| [-----------------] U ||CUGAU| [UArCGUUCAUGUUGGUG---------------------------------] [AGAAAA--] [---------------------------------AACUAAUGUGCUCGUUUA]**

**Myrmecholax sp.(5) [------------------------------------------CGUGCGCGUUUGUCAUCAAGGUUCCUAGUGUGUUGACUGCGCGCGUUGCGUUGCAAAAUUGCUUUAGAACAAAGUCGAAAGUUUUUAU] |CG-UAU|| [-----------------] U ||CUGAU| [UGGCGUUCGCGCUGGUGUAUGCGCGCAGUUUU------------------] [CUAGCUU-] [-----------------------GAAGCUGUGCGUGCAGCAUGUUCGCUCA]**

**Caenocholax sp.(1) [-----------------------------------------------------------------------UGUUCUGUUUCGUUUCGAAAAUCGAUUCGAUUUUCCGUUGUGAAUUUUUUCAAUUCAAU] |CG-UAU|| [-----------------] U ||CCGGA| [CGCGUGCGAGUAGUUGCACUUU----------------------------] [UCG-----] [--------------------------AAAGUACUACUGCUGUCUGUUUGCG]**

**Caenocholax sp.(3) [--------------------------------------------------------------------------------------------------------------GUG-AAUUAUUAGAUUCAAU] |CG-UAU|| [-----------------] U ||CCGGC| [UGCGUUGGAAAUGUUGCGGUC-AGAAAUGCAG------------------] [AUU-----] [------------------------CUGUAUUAACGCGCACUUUUUCAGCCU]**

**Caenocholax sp.(10) [----------------------------------------------------------------------------------------------ACUAUUUUCGCUGUUUGUGAAAUGCUUAAAUUCGAU] |UG-UAU|| [-----------------] U ||CUAAA| [CUAUGUUGAAAGCGUUGCGGUC-AGAGAUGCAAUUU--------------] [CGUAAAAA] [--------------------GAA-UUGCGUUGUCGCGCUCGUUUUCGGCUU]**

**Xenos moutoni [----------------------------------------------------------AUUAAUUGCAUUUUUUUUUAAUUAAAAAAAAAAAUGAUUUUUAUUUUUGAAAAACUUUAAUUAAUUUUUAAU] |UA-UAU|| [-----------------] U ||CUAAU| [AGAAUUGU------------------------------------------] [UUUUU---] [------------------------------------------AUAAUUUUU]**

**Xenos vesparum [---------------------------------------------------------------------------AAAAAUUUUUUAUAAAAAAAUUAAUUUUUUUCGAUAACUUUUAUUAAAUAUUAAU] |UA-UAU|| [-----------------] U ||CUAAU| [AAAAUUAU------------------------------------------] [UUAAAC--] [------------------------------------------GUAAUUUUU]**

**Xenos pecki [-----------------------------------------------------------------AAAAAAAUUUUUUUAUUAAAAAGAUUUUUUUUAUUUUAAAAAAUUUACUUAAAUUAAAAUUUAAU] |UA-UAU|| [-----------------] U ||CUAAU| [AAAAUUAU------------------------------------------] [UUUUU---] [------------------------------------------GUAAUUUUU]**

**Xenos sp. [----------------------------------------------------------------AAAAUUAUUAUUAAAAAAAAAUUAGAUUUAAUUUUUUUUGAAAAAUUUUUGAUUAAAAAAUUAAAU] |UG-UAU|| [-----------------] U ||CUAAU| [AAAAUUAUUUU---------------------------------------] [UUUUAUU-] [---------------------------------------AAAAUGAUUAUU]**

**Paraxenos sp. [??????????????????????????????????????????????????????????????????????????????????????????????????????????????????????????????????] |??????|| [?????????????????] ? ||?????| [??????????????????????????????????????????????????] [????????] [???????????????????????????????????????????????????]**

**Pseudoxenos sp. [------------------------------------------------------------------------------AAAAAAUUAAUAUUUAAUUAAAUUUUUGAUAACUUUUUAUUAAAAAUGAAAU] |UA-UAU|| [-----------------] U ||CUAAU| [AAAAUUAU------------------------------------------] [UUUAU---] [------------------------------------------AUAAUUUUU]**

**Stylops mellitae [-----------------------------------------------------------------ACAUUAGUUUUUUGUGUUGUUUUUCAUUUUAAAUGGGAAAUUAUUUUUUUUCUUUUCAAAUUAAU] |AA-UGU|| [AUUUUGAUGAAAUCUUU] U ||CUAAU| [CGAAAAUUU-----------------------------------------] [--------] [------------------------------------------------UUU] Tridactylophagus sp. [--------------------------------------------------------GUUUGUCGAUUGGAAUGUUGUCAACAAUUUCGUUCGUUGUCUCGGCGUCUCGCUGAAUGGAAAUAUGAUCGAUU] |CG-UAU|| [-----------------] U ||CUAAU| [UGUCGAUAUCGAAAUGGAUUUCGGAA------------------------] [UUUU----] [------------------------------UUUCGUUCCAUUGUCGACAUA] Halictophagus calcaratus [----------------------------------------------------UUUCAAUCGUAAUUUAACAAAUGUUUUUUUUCUUUUUUCAUACCUUUUCGUUUCGUUCAAAUGAAACAAUAACAUUUU] |AA-CAU|| [-----------------] U ||CUAAA| [UGUUGAUUAAAUUUUGUAUGAAAU--------------------------] [GAAA----] [------------------------------AUUUCAACAUUAUCAACAAAU]**

**Halictophagus sp.(3) [----------------------------------AGAAAAAUUUCGAUUAACGUUCAAAUUUAUCGCAUUAGAUUUAAGUGAAAUAAUAUGCAUUUAUCGGUUCGCACAUUUAUUUAGUCGUUUUGUCGA] |CGAUAU|| [-----------------] U ||CUGAC| [UGUUUGACGAUUUAACGAAGAAAUUU------------------------] [GUAAAA--] [-----------------------------GAAAUUUUUUCCAGUCAACAUG]**

**Halictophagus silwoodensis [??????????????????????????????????????????????????????????????????????????????????????????????????????????????????????????????????] |??????|| [?????????????????] ? ||?????| [??????????????????????????????????????????????????] [????????] [???????????????????????????????????????????????????]**

**Halictophagus sp.(2) [??????????????????????????????????????????????????????????????????????????????????????????????????????????????????????????????????] |??????|| [?????????????????] ? ||?????| [??????????????????????????????????????????????????] [????????] [???????????????????????????????????????????????????]**

**Callipharixenos sp. [---------------------------------------------UAUUGUUCAUCGCGCGUAUAGGUAAAAUGUUAUUCGCUUCUUGAAUGCGUGCACAUUGAUUCAUUUAUAUGAAACAUGAACAAUA] |AA-UAU|| [-----------------] U ||CUAAU| [UGUUGAUUAGAAAUUUACAACGGCCUCGCCGACAUCGAA-----------] [UCAGU---] [------------------UUCGUUGCCGGCGGAAUCGUUAUUUUCAGCAAA]**

**Elenchus sp.(1) [----------UUUCACCAUUAUUGAAAAAUUUUAUUAAAUAAUUAAAAAAAAGUUAAUUAAUUAUUUCUUUUUAAUUAAUUAAUAAUUUUUUUAUUUUAAAUAAUUUAUUUAAUUAUCGUAAAAAUAUUU] |UG-UAU|| [-----------------] U ||CUAAU| [AAAAAUUAUGUUAUU-----------------------------------] [UUUAU---] [---------------------------------AAUGCAUAAUUUUUUGAU]**

**Elenchus varleyi [UUUUUUUUACCUAAUUAUUAUUGAAAAAAUUUAAUUUAAGUAAUUUAAAAAAAAGUUAAUCAAUUAUUUAUUUAUUUGAUUAAUAAUUUUUUUGUUUUAAAUAAUUUAUUUAAUUAUCGUAAAAAUAUUU] |UG-UAU|| [-----------------] U ||CUAAU| [UAAAAUUUGUAU--------------------------------------] [UAUU----] [-----------------------------------AUAUAAAUUUUAUUAU]**

**Elenchus koebelei [?????????????????????????????????????????????????????????????????????????????????????????????????????????????????????????????????] |???????|| [?????????????????] ? ||?????| [??????????????????????????????????????????????????] [????????] [???????????????????????????????????????????????????]**

**E23-7’ E23-4’ E23-8 E23-9 E23-10 E23-11 E23-10’ E23-12 RAA18 E23-12’ E23-9’ E23-11’ E23-8’ E23-13 E23-14a E23-14b E23-13’ HV2-V4’ E23-14b’**

**))))) ))))))) ... ((( ............ (( .. (( . (((( .. )) ((((((( ...... )))))) .... )) ......... )))) . ))) . (.((( ... (((((( ((((( )))) ))))))))) .. ... ))))) ......**

**Periplaneta fulig' |GAUGG|| |GCCGGCA|| CGU |UUA| CUUUGAACAAAU |UA| GA |GU| G |CUUA| AA |GC| ||AGGCAGC| [AG----] |CCGCCU|| -GAA |UA| CCGAGUGCA |UGGA| A |UAA| U |GGAAU| AGG |ACCUCG| |GUUCU| |AUUU| * |GUUGGUUUU| -- ** --C |GGAAU| ---CCC**

**Tamolanica tamolana |GAUGG|| |GCCGGCA|| CGU |UUA| CUUUGAACAAAU |UA| GA |GU| G |CUUA| AA |GC| ||AGGCAGC| [AU----] |CCGCCU|| -GAA |UA| CCGAGUGCA |UGGA| A |UAA| U |GGAAU| AGG |ACCUCG| |GUUCU| |AUUU| * |GUUGGUUUU| -- ** --C |GGAAU| ---CCC**

**Reticulitermes fla’ |GAUGG|| |GCCGGCA|| CGU |UUA| CUUUGAACAAAU |UA| GA |GU| G |CUUA| AA |GC| ||AGGCAGC| [AG----] |CCGCCU|| -GAA |UA| CCGAGUGCA |UGGA| A |UAA| U |GGAAU| AGG |ACCUCG| |GUUCU| |AUUU| * |GUUGGUUUU| -- ** --C |GGAAU| ---CCC**

**Antheraea pernyi |GGUGG|| |GCCGACA|| AUU |UUA| CUUUGAACAAAU |UA| GA |GU| G |CUCA| AA |GC| ||GGGCUCA| [AAAUG-] |CUGCUU|| -GAA |UA| UUUCGUGCA |UGGA| A |UAA| U |AGAAU| AUG |AUCUCG| |GUUCU| |AUUU| * |GUUGGUUUU| -- ** --C |AGAAC| ----UC**

**Bombyx mori |GGUGG|| |GCCGACA|| AUU |UUA| CUUUGAACAAAU |UA| GA |GU| G |CUCA| AA |GC| ||GGGCUAA| [AAAUG-] |CUGCUU|| -GAA |UA| UUUCGUGCA |UGGA| A |UAA| U |AGAAU| AUG |AUCUCG| |GUUCU| |AUUU| * |GUUGGUUUU| -- ** --C |AGAAC| ----UC**

**Haematobia irritans |UGUGG|| |GCCGGUA|| CAA |UUA| CUUUGAACAAAU |UA| GA |GU| G |CUUA| AA |GC| ||AGGCUCC| [AA----] |AUGCCU|| -GAA |UA| UUUUGUGCA |UGGA| A |UAA| U |GAAAU| AAG |ACCUCU| |GUUCU| |ACUU| * |AUUGGUUGU| -- ** --U |AGAUC| -----A**

**Ceratitis capitata |UGUGG|| |GCCGGUA|| CAA |UUA| CUUUGAACAAAU |UA| GA |GU| G |CUCA| AA |GC| ||AGGCUCC| [AA----] |AUGCCU|| -GAA |UA| UUUUGUGCA |UGGA| A |UAA| U |GAAAU| AAG |ACCUCU| |GUUCU| |ACUU| * |AUUGGUUUU| -- ** --U |AGAUC| -----A**

**Tribolium castaneum |GGUGG|| |GCCGGCA|| C-U |UUA| CUUUGAACAAAU |UA| GA |GU| G |CUCA| AA |GC| ||AGGCUAA| [AA-CU-] |UCGCCU|| UGAA |UA| CUGUGUGCA |UGGA| A |UAA| U |GGAAU| AGG |ACCUCG| |GUUCU| |AUUU| * |GUUGGUUUU| -- ** --C |GGAAC| ----CC**

**Rhagophthalmus ohb' |GGCGG|| |GCCGGCA|| CGU |UUA| CUUUGAACAAAU |UA| GA |GU| G |CUUA| AA |GC| ||AGGCUAA| [AA-CU-] |UCGCCU|| -GAA |UA| UUGUGUGCA |UGGA| A |UAA| U |GGAAU| AGG |ACCUCG| |GUUCU| |AUUU| * |GUUGGUUUU| -- ** --C |GGAAU| ----CC**

**Mengenilla australiensis |AUCGG|| |ACCAGUG|| AGU |UUA| CUUUGAACAAAU |UA| GA |GU| G |CUUA| AA |GC| ||AGGCAAA| [UUUGA-] |AUGCCU|| UGAA |UA| UGA-GUGCA |UGGA| A |UAA| U |AGAAG| AUG |AUUUCG| |AUACC| |GUUU| * |AACGGCUUU| -U ** G-C |GGAAU| ----AU**

**Mengenilla chobauti |?????|| |???????|| ??? |???| ???????????? |??| ?? |??| ? |????| ?? |??| ||???????| [??????] |??????|| ???? |??| ????????? |????| ? |???| ? |?????| ??? |??????| |?????| |????| * |AUUGGCUUA| AA ** ??? |?????| ---???**

**Triozocera sp.(2) |GUCAG|| |GCCAGCG|| AUA |UUA| CUUUGAACAAAU |UA| GA |GU| G |CUCA| AA |GC| ||AGGCAAA| |UUUAU-] |AUGCCU|| UGAA |UA| UCG-CAGCA |UGGA| A |UAA| U |AGAAU| AUG |AUCUCG| |GUACC| |GUUU| * |AUUGGCUUA| AA ** GAC |GGUAU| ----AU**

**Triozocera sp.(3) |GUCAG|| |GCCAGCG|| AUA |UUA| CUUUGAACAAAU |UA| GA |GU| G |CUCA| AA |GC| ||AGGCAAA| [UUUAU-] |AUGCCU|| UGAA |UA| UCG-CAGCA |UGGA| A |UAA| U |AGAAU| AUG |AUCUCG| |GUUCC| |GUUU| * |AUUGGCUUA| AA ** GAC |GGUAU| ----AU**

**Triozocera sp.(1) |GUCAG|| |GCCAGCG|| AUA |UUA| CUUUGAACAAAU |UA| GA |GU| G |CUCA| AA |GC| ||AGGCGUG| [---AAU] |AUGCCU|| UGAA |UA| UCG-CAGCA |UGGA| A |UAA| U |AGAAU| AUG |AUCUCG| |GUACC| |GUUU| * |AUUGGCUUA| AA ** GAC |GGUAU| ----AU**

**Triozocera sp.(4) |GUCAG|| |GCCAGCG|| AUA |UUA| CUUUGAACAAAU |UA| GA |GU| G |CUCA| AA |GC| ||AGGCAAA| [UUUAU-] |AUGCCU|| UGAA |UA| UCG-CAGCA |UGGA| A |UAA| U |AGAAU| AUG |AUCUCG| |GUACC| |GUUU| * |AUUGGCUUA| AA ** GAC |GGUAU| ----AU**

**Blisseoxenos esaki |GUCGG|| |GCCAGUA|| GAA |UUA| CUUUGAACAAAU |UA| AG |GU| G |CUCA| AA |GC| ||AGGCAAA| [UUUAU-] |AUGCCU|| UGAA |UA| UCG-CAGCA |UGGA| A |UAA| U |AGAAU| AUG |AUCUCG| |GUACC| |GUUU| * |AUUGGCUUA| AA ** GAC |GGUAU| ----AC**

**Corioxenos acucyrtophallus |GUCGG|| |GCCAGUU|| GUA |UUA| CUUUGAUAAAAU |UG| GA |GU| G |CUUA| AA |GC| ||AAGCUCA| [UUUAU-] |AUGCCU|| -GCA |CA| UAU-UUUCA |UGGA| A |UAA| U |AGAAU| AUG |AUCUCG| |GUACC| |GUUU| * |AAAGGCUUA| AC ** UAC |GGUGU| ----AC**

**Lychnocolax sp.(2) |?????|| |???????|| ??? |???| ???????????? |??| ?? |??| ? |????| ?? |??| ||???????| [??????] |??????|| ???? |??| ????????? |????| ? |???| ? |?????| ??? |??????| |?????| |????| * |GUUGGCUUA| AA ** ??? |?????| ??????**

**Lychnocolax sp.(1) |?????|| |???????|| ??? |???| ???????????? |??| ?? |??| ? |????| ?? |??| ||???????| [??????] |??????|| ???? |??| ????????? |????| ? |???| ? |?????| ??? |??????| |?????| |????| * |?????????| ?? ** ??? |?????| ??????**

**Stichotrema dallatoreanum |UGCAG|| |ACCGACU|| GGA |UUA| CUUUGAAUAAAU |UA| AA |GU| G |CUUA| AA |GC| ||AGGCAGA| [UUUUAA] |UUGCCU|| UGAA |UA| UUAUAAGCA |UGGA| A |UAA| U |AGAAU| AUG |AUCUCG| |AUACU| |GUUU| * |AUUGGCUUA| AA ** GAC |AGUAU| ??????**

**Stichotrema sp. |AUUAG|| |GCCGACU|| UAA |UUA| CUUUGAAUAAAU |UA| AA |GU| G |CUUA| AA |GC| ||AGGCGAU| [UUGAAA] |AUGCCU|| UGAA |UA| GAACAGCA |UGGA| A |UAA| U |AGAAU| AUG |AUCUCG| |AUAUU| |GUUU| * |AUUGGCUUA| AA ** GAC |AAUUG| -----U**

**Myrmecolax sp.(4) |AUUGG|| |GUCGUCA|| AUA |UUA| CUUUGAAUAAAU |UA| GA |GU| G |CUUA| AA |GC| ||AGGCAUA| [UUGAA-] |CUGCCU|| UGAA |UA| UGAACAGCA |UGGA| A |UAA| U |AGAAU| AUG |AUCUCG| |AUACC| |GUUU| * |AUUGGCUUA| AA ** ??? |?????| ??????**

**Myrmecolax sp.(3) |AUUGG|| |GUCGUCA|| AUA |UUA| CUUUGAAUAAAU |UA| GA |GU| G |CUUA| AA |GC| ||AGGCAUA| [UUGAA-] |CUGCCU|| UGAA |UA| UGAACAGCA |UGGA| A |UAA| U |AGAAU| AUG |AUCUCG| |AUACC| |GUUU| * |AUUGGCUUA| AA ** ??? |?????| ??????**

**Myrmecolax sp.(1) |AUUGG|| |GUCGUCA|| AUA |UUA| CUUUGAAUAAAU |UA| GA |GU| G |CUUA| AA |GC| ||AGGCAUA| [UUGAA-] |CUGCCU|| UGAA |UA| UGAACAGCA |UGGA| A |UAA| U |AGAAU| AUG |AUCUCG| |AUACC| |GUUU| * |AUUGGCUUA| AA ** GAC |GGUAU| ----AU**

**Myrmecolax sp.(2) |AUUGG|| |GUCGUCA|| AUA |UUA| CUUUGAAUAAAU |UA| GA |GU| G |CUUA| AA |GC| ||AGGCAUA| [UUGAA-] |CUGCCU|| UGAA |UA| UGAACAGCA |UGGA| A |UAA| U |AGAAU| AUG |AUCUCG| |AUACC| |GUUU| * |AUUGGCUUA| AA ** GAC |GGUAU| ??????**

**Myrmecholax incautus |GUUGG|| |GUCGUCA|| AUG |UUA| CUUUGAAUAAAU |UA| GA |GU| G |CUUA| AA |GC| ||AgGCGUA| [UUUAAU] |CUGCCU|| UGAA |UA| UAAACAGCA |yGGA| A |UAA| U |AGAAU| AUG |AUCUCG| |AUACC| |GUUU| * |AUUGGCUUA| AA ** GAC |GGUAU| ??????**

**Myrmecholax sp.(5) |AUUGG|| |GUCGUCA|| AUA |UUA| CUUUGAAUAAAU |UA| GA |GU| G |CUUA| AA |GC| ||AGGCAUA| [UUGAA-] |CUGCCU|| UGAA |UA| UGAACAGCA |UGGA| A |UAA| U |AGAAU| AUG |AUCUCG| |AUACC| |GUUU| * |AUUGGCUUA| AA ** GAC |GGUAU| ??????**

**Caenocholax sp.(1) |UUCGG|| |ACCAGCG|| AAA |UUA| CUUUGAACAAAU |UA| GG |GU| G |CUUA| AA |GC| ||AGGCAAA| [UUUUUA] |AUGCCU|| UGAA |UA| UAAACAGCA |UGGA| A |UAA| U |AGAAA| AUG |AUCUCG| |AUACU| |GUUU| * |AUUGGCUUA| AA ** GAC |?????| ??????**

**Caenocholax sp.(3) |GUCGG|| |ACCAGCG|| AAA |UUA| CUUUGAACAAAU |UA| GA |GU| G |CUUA| AA |GC| ||AGGCAAA| [UUUU--] |AUGCCU|| UGAA |UA| UACACAGCA |UGGA| A |UAA| U |AGAAU| AUG |AUCUCG| |AUACU| |GUUU| * |AUUGGCUUA| AA ** GAC |AGUUU| ??????**

**Caenocholax sp.(10) |GUCGG|| |GCCGGCG|| AAA |UUA| CUUUGAACAAAU |UA| GA |GU| G |CUUA| AA |GC| ||AGGCAAA| [UUUU--] |AUGCCU|| UGAA |UA| UAAACAGCA |UGGA| A |UAA| U |AGAAU| AUG |AUCUCG| |AUACU| |GUUU| * |AUUGGCUUA| AA ** GAC |AGUAU| ??????**

**Xenos moutoni |AUUAG|| |ACCAGUA|| AAA |UUA| CUUUGAACAAAU |UA| GA |GU| G |CUUA| AA |GC| ||AGGCAAA| [UUUAU-] |AUGCCU|| UGAA |UA| UAA-CAGCA |UGGA| A |UAA| U |AGAAU| AUG |AUCUCG| |AUACU| |GUUU| * |AUUGGCUUA| AA ** GAC |AGUAU| ---GAU**

**Xenos vesparum |AUUAG|| |ACCAGUA|| AAA |UUA| CUUUGAACAAAU |UA| GA |GU| G |CUUA| AA |GC| ||AGGCAAA| [UUUAA-] |AUGCCU|| UGAA |UA| UAA-CAGCA |UGGA| A |UAA| U |AGAAU| AUG |AUCUCG| |AUACU| |GUUU| * |AUUGGCUUA| AA ** GAC |AGUAU| ---GAU**

**Xenos pecki |AUUAG|| |ACCAGUA|| AAA |UUA| CUUUGAAGAAAU |UA| GA |GU| G |CUUA| AA |GC| ||AGGCAAA| [UUUAU-] |AUGCCU|| UGAA |UA| UAA-CAGCA |NGGA| A |UAA| U |AGAAU| AUG |AUCUCG| |AUACU| |GUUU| * |AUUGGCUUA| AA ** GAC |AGUAU| ---GAU**

**Xenos sp. |AUUAG|| |ACCAGUA|| AAA |UUA| CUUUGAACAAAU |UA| AA |GU| G |CUUA| AA |GC| ||AGGCAAA| [UUUAA-] |AUGCCA|| UGAA |UA| UAA-CAGCA |UGGA| A |UAA| U |AGAAU| AUG |AUCUCG| |AUACU| |GUUU| * |?????????| ?? ** ??? |?????| ??????**

**Paraxenos sp. |?????|| |???????|| ??? |???| ???????????? |??| ?? |??| ? |????| ?? |??| ||???????| [??????] |??????|| ???? |??| ????????? |????| ? |???| ? |?????| ??? |??????| |?????| |????| * |?????????| ?? ** GAC |AGUAU| ??????**

**Pseudoxenos sp. |AUUAG|| |ACCAGUA|| AAA |UUA| CUUUGAACAAAU |UA| GA |GU| G |CUUA| AA |GC| ||AGGCAAA| [UUUAU-] |AUGCCU|| UGAA |UA| UAA-CAGCA |UGGA| A |UAA| U |AGAAU| AUG |AUCUCG| |AUACU| |GUUU| * |AUUGGCUUA| AA ** ??? |?????| ??????**

**Stylops mellitae |AUGAG|| |ACCAGUA|| AAA |UUA| CUUUGAACAAAU |UA| rA |GU| G |CUUA| AU |GC| ||AGGCAAA| [UUUAU-] |AUGCCA|| UGAA |UA| -AAACAGCA |UGGA| A |UAA| U |AGAAU| AUG |AUCUCG| |AUACU| |GUUU| * |GUUGGCUUA| AA ** GAC |AgUAU| ??????**

**Tridactylophagus sp. |UUUAG|| |ACCAGUU|| AAA |UUA| CUUUGAACAAAU |UA| GA |GU| G |CUUA| AA |GC| ||AGGCUAC| [AUUAU-] |GUGCCU|| UGAA |UA| UAAACAGCA |UGGA| A |UAA| U |AGAAU| AUG |AUCUCG| |AUACU| |GUUU| * |GUUGGCUUA| AA ** GAC |Ag???| ??????**

**Halictophagus calcaratus |UUUAG|| |ACCAGUU|| AAA |UUA| CUUUGAACAAAU |UA| GC |GU| G |CUUA| AA |GC| ||AGGCUCG| [AUUAU-] |AUGCCU|| UGAA |UA| UGAAAAUCA |UGGA| A |UAA| U |AGAAU| AUG |AUCUCG| |AUACU| |GUUU| * |GUUGGCUUA| AA ** GAC |AGUAU| ----AU**

**Halictophagus sp.(3) |GUAAG|| |GCCAGUA|| AUA |UUA| CUUUGAACAAAU |UA| GA |GU| G |CUCA| AU |GC| ||AGGCUCA| [AUUAU-] |AUGCCU|| UGAA |UA| UAAACAGCA |UGGA| A |UAA| U |AGAAU| AUG |AUCUCG| |AUACU| |GUUU| * |AUUGGCUUA| AA ** GAC |AGU??| ??????**

**Halictophagus silwoodensis |?????|| |???????|| ??? |???| ???????????? |??| ?? |??| ? |????| ?? |??| ||???????| [??????] |??????|| ???? |??| ????????? |????| ? |???| ? |?????| ??? |??????| |?????| |????| * |?????????| ?? ** ??? |?????| ??????**

**Halictophagus sp.(2) |?????|| |???????|| ??? |???| ???????????? |??| ?? |??| ? |????| ?? |??| ||???????| [??????] |??????|| ???? |??| ????????? |????| ? |???| ? |?????| ??? |??????| |?????| |????| * |?????????| ?? ** GAC |AGU??| ??????**

**Callipharixenos sp. |UUUAG|| |ACCAGUU|| AAA |UUA| CUUUGAACAAAU |UA| GA |GU| G |CUUA| AA |GC| ||AGGCUUC| [AUUAU-] |AUGCCU|| UGAA |UA| UAAACAGCA |UGGA| A |UAA| U |AGAAU| AUG |AUCUCG| |AUACU| |GUUU| * |GUUGGCUUA| AA ** GAC |AGUAU| ----AU**

**Elenchus sp.(1) |UUUAG|| |ACCAAUA|| AAA |UUA| CUUUGAACAAAU |UA| GA |GU| G |CUUA| AA |GC| ||AGGCAAA| [AUUAA-] |AUGCCU|| UGAA |UA| UAAACAGCA |UGGA| A |UAA| U |AGAAU| AUG |AUCUCG| |AUACU| |GUUU| * |AUUGGCUUA| AA ** GAC |A????| ??????**

**Elenchus varleyi |UUUAG|| |ACCCAUA|| AAA |UUA| CUUUGAACAAAU |UA| GA |GU| G |CUUA| AA |GC| ||AGGCAAA| [AUUAA-] |AUGCCU|| UGAA |UA| UAAACAGCC |UGGA| A |UAA| U |AGAAU| AUG |AUCUCG| |AUACU| |GUUU| * |AUUGGCUUA| AA ** GAC |A????| ??????**

**Elenchus koebelei |?????|| |???????|| ??? |???| ???????????? |??| ?? |??| ? |????| ?? |??| ||???????| [??????] |??????|| ???? |??| ????????? |????| ? |???| ? |?????| ??? |??????| |?????| |????| * |?????????| ?? ** ??? |?????| ??????**

**[* STREPSIPTERA CAP 1-2 Expansion segment]**

**Periplaneta fulig' U**

**Tamolanica tamolana U**

**Reticulitermes fla’ U**

**Antheraea pernyi U**

**Bombyx mori U**

**Haematobia irritans U**

**Ceratitis capitata UC**

**Tribolium castaneum U**

**Rhagophthalmus ohb' U**

**Mengenilla australiensis UUAUUUU**

**Mengenilla chobauti sequence read runs out 🡨 AUCGCGUCAGAUUAGUGAAAAAGCAUUUUUCGUUUGAUAGUAUGCGCAAGUGUAUUGAAGGCGAAAUAAA**

**Triozocera sp.(2) UCGUUUGAA**

**Triozocera sp.(3) UCGUUUGAA**

**Triozocera sp.(1) UCGUUUUGAA**

**Triozocera sp.(4) UCGUUUGAA**

**Blisseoxenos esaki UCAUUUGAA**

**Corioxenos acucyrtophallus UCAUUUGAA**

**Lychnocolax sp.(2) sequence read runs out 🡨 AAAAAAAAAAAAAAUUAAUCUAUA**

**Lychnocolax sp.(1) ?**

**Stichotrema dallatoreanum UUGUUUUAAGAUUUAUUUAUUGCGUACGCGAAGAUGAAUUCGUUGUUUAAUGUAACGUGUUAUACAUGAUACUUUUGCACGAAAGUUGUCUUUGUACGUGAUAUAUAAUAUCAGA**

**Stichotrema sp. UCG**

**Myrmecolax sp.(4) CUUUUCAAACGUACACAUUGGCACUGUUCGUGGUGAUCGGUCUACCGUUUGCUGUUCAGUGUGUUUGUGCGUUUCUA**

**Myrmecolax sp.(3) CUUUUCAAACGUACAUUAACGCUGUUUUCGGUGGCGGCAAUUGGUCAAACGAUUGCUGCUGCUGUUCGGUGUUAGUGCGUUUCUA**

**Myrmecolax sp.(1) CGUUUCAAACGUACAUUUAACGAUGUGGUGAUCCGUUAAACGAUUGUCGCAGUUUUGUGCGUUUCUA**

**Myrmecolax sp.(2) CUUUUCAAGCGUACGUUGACGCUGUUUUUGGUGGCGGUGGCAAUUGGUCAAACGAUUGCUGCUGCUGCUGUUCGGUGUUUGUGCGCGUUUCUA**

**Myrmecholax incautus CUUUUCAAGUGUGUGUUUGUGCGUUUUCUUCCGAGAGAGCACACUGGACGCGCGCUUCUA**

**Myrmecholax sp.(5) CUUUUCAAACGCACAUUGACGCUGUUGUUGGUGGUCGGUCAAACGAUUGCUAAAUUCGGUGUUUGUGCGCGUUUCUA**

**Caenocholax sp.(1) UCGUAAUUUGUUGUUUGCUGCUGCGUACGUUACAACGAUUUAUGUCGUAGUUUGUGCGUAGCUCGAGCAGCGAAAUAAA**

**Caenocholax sp.(3) GUUUGAUGCUUUCUUGUUUUUCGGAGCAAAAAAGUAUUAUUCAUCUGAUAGUAUGCAUUGGUUUUAAUUGACUAGUGUUGUAUGGUCGAUGGAAAGAA**

**Caenocholax sp.(10) GUUUAAUGUUUUUCUUAUCUUCGGAUAGAAAGGCAUUUUUCGUUUGAUGAUAUGCGUAAGCGUAUUGUGAGGCGAAAUGAA**

**Xenos moutoni UUAAAAAAAUAUUAUUUUUUUUUUUGAAUUCUUUUUUUUUCUUUUAGAAAAAGAAAGUUUUUAUUAUAAAAAAUAAAUAAAA**

**Xenos vesparum AAAAAAGAAAAUUUUUUUCUGAUUUUUUUUUUUUUUAAAUGAUUAGAAAAAAAUAAAA**

**Xenos pecki AAAAAAAGUUUUUUUUUUUUUAUUUUAUAUAAAAAAAAAAAUAAAA**

**Xenos sp. AGUAAAAAAAAAUUUCUUUUUUGUAAAAGGUUAAUUUAAAUUUAAUUAAAAAAAAUUAUUUUUACCAAAAAAAAAAUUUUAGAAAA 🡪 sequence read runs out**

**Paraxenos sp. ?**

**Pseudoxenos sp. UUUUAAAAUAUGAAAUUUUUUAUUCUUUUGAAUAAAAAUUUCAAAAAAA**

**Stylops mellitae UUUUUUUUUUUUUUUUUCAAAAgrdkgsgkUUUUUUUUUCUUUUgUCgAUAgAkgAAAgggrAmCmAAACsCyyyCmAAAAAAAAAAAAAA**

**Tridactylophagus sp. GUUGAACUGUUUCGCGAUUGGCCCACGUGCUUUUGCCCGUGCGCGCUGUGUGAAACACUAG**

**Halictophagus calcaratus GGUGAAAACUGCUUGAAAAAAAAAUUGUAAAAAAUUUUUUUUUCAACACUAAAA**

**Halictophagus sp.(3) UUUUAUUGUUUAGUUUAGAAAUUGAUUAAGAUAAGAAUUUUUUCAAAAUUUUUUGACUAAUUGAUUUUAUUAAAUUAAACGAAAA**

**Halictophagus silwoodensis ?**

**Halictophagus sp.(2) ?**

**Callipharixenos sp. GUUAAAACUGUUGCAAAUUGCCGUUUUUCGGnnnnnnnnnnnnnAACUUUGGCGAUAUUUAUUUAUUGUUCAUCGCGCGUAUAGGUAAAAUGUUAUUCGCUUCUUGAAUGCGUGCACAUUGAUUCAUUUAUAUGAAACAUGAACAAUAAAUAUUCUAAUUGUUGAUUAGAAAUUUACAACGGCCUCGCCGACAUCGAAUCAGUUUCGUUGCCGGCGGAAUCGUUAUUUUCAGCAAAUUUAGACCAGUUAAAUUACUUUGAACAAAUUAGAGUGCUUAAAGCAGGCUUCAUUAUAU**

**GCCUUGAAUAUAAACAGCAUGGAAUAAUAGAAUAUGAUCUCGAUACUGUUUGUUAAAACUGUUGCAAAUUGCCGUUUUUGGUGCAUGUUCACACAUGUGCCGGACGUGUAUUCGUUUgGUAACGAAAA**

**Elenchus sp.(1) UUAAAAAUAUUAAAAAAUAUUUAAUGUACAAAAAUUUUUCAUUUUUUUUUAAAAAUGACAAAAUUUGUACAUUUUAA**

**Elenchus varleyi UUUUUUAAUUUUUUUUAAAAUAAUUUUAAAAAAAAUAUUUAAUGUUAGAAAAAAUUUUAUUAUUUUUUCGAAUGUAUAAAAUUUUUUUCAUACAUUUUAA**

**Elenchus koebelei ?**

**[** STREPSIPTERA CAP3 expansion segment]**

**Periplaneta fulig' -**

**Tamolanica tamolana -**

**Reticulitermes fla’ -**

**Antheraea pernyi -**

**Bombyx mori -**

**Haematobia irritans -**

**Ceratitis capitata -**

**Tribolium castaneum -**

**Rhagophthalmus ohb' -**

**Mengenilla australiensis GCACCGUCGAAUGCCGGGCCGCUUUGCGCUUGAUGCGCUUCGUGUGUGUCGUCCGUGAAGUGGUCGCCUGUGCACGGCGGGC**

**Mengenilla chobauti GUGAACAUUGCGGGAGAAUCUGAAA 🡪 sequence read runs out**

**Triozocera sp.(2) GGGCUAGAUAAAACCGGAAACGGUGUUCGGAUGCGCGGGCACGAUACCGGCUACGUCGACGCCUAGUGUGUACUCCGCCCGGGCGUAUAACGGGACGCGUGUGCAAUUCGUGUCCACGUGCGCGGCCCAGUGCUGCUGUGUGAUACGAGCGCACGCGUACCGGUCCGGUUGCGCGAGCACCGGUGUCACUAAAUGGUGUACGCCGUUUAGUAUCGCGUGUGCCCGAUGCAACUCGAAUUAUUUAGUCC**

**Triozocera sp.(3) GGGCUAGAUAAAACCGGAAACGGUGUUCGGAUGCUCGGGCACGGUACCGGGCACAUCGACGCCUAGUGUGCACUCCGUCGGACGCAUAACGGAACGCGUGUGCAAUUCGUAUCUACGUACGCCUAGUGCUGCGAAUACGAGCGCUCGCGUGCCGGUCCGAAUGCGAGCACCGGCGUCACUAAAAUGGUGUGCAUCGUUUAGUAUCGUGUGUGCCCGAUGCAACUCGAAUUAUUUAGCUC**

**Triozocera sp.(1) GGGCUAGAUAAAACCGGAAACGGUGUUCAAAUGCUCGGGCACGAUGCCGGCACGCCGAUGCUUUGUGUGUACUUACUACGAACGCUAUACGGUACGUGGGCAAUUCGUGCGCUCGCAUAAAAAUUGCAGUCACGAGCUCACGAACCGGUUUGUGUAAACACCGGUGUCGCUAAAGGCGUUUUGCCGUUUAGUAUCUUAUGUGACCGAUGCAUCUGAAUUAUUAGUCC**

**Triozocera sp.(4) GGGCUAGAUAAAACCGGAAACGGUGUUCGGAUGCUCGGGCACGGUACCGGGCACAUCGACGCCUAGUGUGCACUCCGUCGGACGCAUAACGGAACGCGUGUGCAAUUCGUAUCUACGUACGCCUAGUGCUGCGAGUACGAGCGCUCGCGUGCCGGUCCGAAUGCGAGCACCGGCGUCACUAAAAUGGUGCGCAUCGUUAGUAUCGUGUGUGCCCGAUGCAACUCGAAUUAUUUAGCUC**

**Blisseoxenos esaki GUGGCAGAUAAGCUAAAUUCGGCGCGCCGGGCACGGCGCACGGCUGUCUCGGCACGCUUGCCGCGC GCGCACGCACACUCACUUGUUGUUUAUGCGUGCGCCGCGCUUGCGUGCACGUACGAGCGCCGCACGCUGUCUAUGUGUUCGCGCGUAUGCUCGAAUUAUUUGCCAC**

**Corioxenos acucyrtophallus GAGCUGAAUAAACAUGUUUGCGUUCGAUUCUCGUGGACGAACCACCCGAAAGUUGAAUAUUGGUCGCUCGCUAAGUGUGGCAAUUUGCGCUAGUGCGUAAAUGGGCUUCCA AUGUUGUCGAUCAGGUGUUUGUCCAUCAUGCGAGUUUCGUCCGUAAAUUAUUCAGUUC**

**Lychnocolax sp.(2) GAAAAUAAAUAAAAAAAAAUUUAAAAUUAUACUAUCGUUAAAAUAUAUUUUUUUAUUUUUUAAGAAAAAGAAAUUAAAAUAUGAAAUUAAUUUUUCAUAUUUUUUUUUAUUCUUUUUUUAAAUAAAAAAAUAUUGAUAGAAAAAUUUAAACUAUUAUUU**

**Lychnocolax sp.(1) ?**

**Stichotrema dallatoreanum GAAGUGUAAUUCGUUGAAUUAAAUUUGUGAUUCGAAUUUUGUUCAAAUGAGCGCAAUUCGUUUUGUGUGCGACAAUGAAUUUUCGUUUGUCAAUCGUUAGCAAUUCGUUUAUUGUCGACUUAUUUAGGACAAUAAUGAAUCACUCGAUUCUUUAUAGAGUUACUUC**

**Stichotrema sp. GGGGCUGCGCUGCCGGGCUCGCCGGAUCGUUCCUCUUGGGUUCGAUGGCGGACGCGGCGGCCGGCUCC**

**Myrmecolax sp.(4) GAAGCAAACCUCUUGAGUUUCGAUCCAGAAAUGAAGGAUUGUUCGA 🡪 sequence read runs out**

**Myrmecolax sp.(3) GAAGCAAAAAUGUUGUGUUUCGAUUCAGAAAUGCAUAAUUGUU 🡪 sequence read runs out**

**Myrmecolax sp.(1) GAGGCAUACCACUUGCGUUUCGAUUCAGAAAUGCAAGGGUUUGUUUC**

**Myrmecolax sp.(2) GAAGCAAACAUGUUGCGUUUCGAUUCAGAAAUGCAUAAUUUGUUUC**

**Myrmecholax incautus GAAGCAGAUUUCUUGUUACGGACAUCGUUCGAUUGUUGGUUUUGUGAUCGCGGCCGGUGGUUAGCUUGCUGACUACGCGCGCAUCCUACrCAGACAGUCUAGUACGGUUCGUUUCAUGAAUGGAGUUGUUUC**

**Myrmecholax sp.(5) GAAGCAAACCUCUGGAGUUUCGAUUGAGAAAUGAAGGGUUUGUUUC**

**Caenocholax sp.(1) GUGAAUGAAAUGUAUGCAAAUUCAUAAAAGACGUAACAAUACAACACUAUCGAUAAAAUACAACGUGCGCGCGAACGCUCGCACGCUUGCGUCCGAUUGGAAACAUUUCUAAUCGUUUUGUUUUCGAAUUAUUCUUUCGUACAUAAGUGUUGUACCUCUUGGAAUUUUUUUUUUCGUUUAUUCGGAAAAAAAAUCCACGUAUGUCUUACUCGCAGUUUUCUGUGUGGUAAUGACAUUUCGCCAAAAAAAAAGAGUCUAAAUGGACGUAUGCCCGUUUAGUUUGUACGCUACAAAA**

**CGCUAGUUCGAUCGAGUUUUCGACAAAUCGUGUGUGUUUACAAUACGCGGUUUGUACAACGAAUUUUGUCGCUGUUGUCGUCGUCGUCGUGUUCGUUGUUGCUAUGUUUGUAUUUUGAUUGCGUUGAUUAUGUUUUCGUUUUCUGAAUUUGUGUUUUGUUCAC**

**Caenocholax sp.(3) GUGACAUUAUCAGCUGAUCGGCUAAAGCAGUAGGUGAAAAAUUGAUUGAAAAUGUUUUCUAGCGUUUCCAUAAAAAUCGGUUUUUUAUCCUACUGUGACGGUUUGCAUUUUGUCAC**

**Caenocholax sp.(10) GUGGCAAAGUUGGGAAAUCUUAAAUGAUUUUCUUUUUGUCCAC**

**Xenos moutoni GAAAUAAAUAAUUUACUUAAAAUUUUUAUACAAAAAUAACAAAAAUUCGUAUCGUUGCAAAGAUUUGAUUUUUUGUAUUUAUUUUUUUGUUUUUUACAAAAAAAUUUACAAAAUCUAUUUGUUUUUUUUUUUUUUAAAAUUAAAUAAAAUAGAUAUUAAUUAAUUUUUUUGCAUUAAAAAACGAUUCGAAAAUAUUUUUGUUAGUUUUUUUUUUUAAAUUUUUUUGUUUAAAAAAGUUUUAAGGAUUUAUUUC**

**Xenos vesparum GAAAUAAAUAAUUUUACUUAAAAAAAAGAUUUUUUUUUUUUUUUUGAUUUAUACAAAAAUAGUAAAAUUUCUUAUCGUUGCAAAAAUUAUAUUUAAAUUUUUUUAAUUUUUGUAUUUUUUUUUUUUAAAUAAAAAAAAAUAACAAAAACUAUUUACAAUUUUUUUUUUUUAAAAAAAAUAAUUUUGUAAAUAGAAAAUUUAAAAACUUUUUUGCAUCCAUAACGUUAAGAAAAUUUUUUUUUUGUUAAAAAAAAAUAAUUUCUUUUUUUUAAGAAUUUAUUUC**

**Xenos pecki GAAAUAAAUAAAAAUUAAAAUUUUUCCCAAAAAAAAAUUACCAAAAUAUUUC 🡪 sequence read runs out 🡨 GUAAAAAAAAAAGUUUUUUUUUUGUCUUUUUUUUGUGUUAAAAAAAAAUGGUAAGAAUUUAUUKC**

**Xenos sp. ?**

**Paraxenos sp. sequence read runs out 🡨 UGUUGGUAUCGUUUGCAAAAAAUUUCAAACGAAAAAUUUUCGUUUUGUGUUUAACAAAGAUCUGUUUUACGUUUAAAUAUUUUUUUCGAUUUUUGAGAAAAAUUUCGAAAAAAAUAUGUAAAUCUAGAUUAGAAAAUUUUUUCGUWAAUUUUUGCAAUUAAAAACGUUACUUUAACUACUGUUAUUUUAAUUUGUUUAAAAAAUUUAAGAAUUAUUUC**

**Pseudoxenos sp. GAAAUAAAAUUAUUACUUGAAAUUUAUAUUGCAAAGAAAACAUGGUUUUUUUUUGUUCAUUGCAAAAUUUUUUUGUAUUAAAAUGAUUUAAAAAAAAAACAAUAUUAUUUUGUAAAAUUUCAUGUAUUUAUUUCGA 🡪 sequence read runs out**

**Stylops mellitae GAAAUAAAUAGUUAUGUAUAAAAUUGUUUCAUACUUAAAAUUCGAAAAAGUUUUUAAUCGAUAAAUUUUUUCCGGAAUUUUUCUUUUGUGAACAAAAUUUAUCGAACUUUUUUGAAUUUUGUAACAAUUUUACGUAUUUAUUUC**

**Tridactylophagus sp. GAAUUGAAUAUUAUUUGAAUAUUGUAUUCAAAAUAGAGGAAUCCGGUCUAGGCAAUUUGUUCGAUCGAAUUUUUCGGGGCUUGCGAUGUCCGUCUACGCGUUGCAUACAAACGCGUUCCUGCGGCCAUUGUGCACAGGAUCGUUUGCUCGCGUGCAAAGGGCGGCGCGCGUUUACGGGUCUCGGAAUCUUUAACGAUCGGACGCUACAAAUUGUCUUUUUAGUGUCGGUUGUUUUCUAUnUAnGAUAUUCAAUUUC**

**Halictophagus calcaratus GAAUUGAUCAUUAUUUGAAUAUAUCAUUAUUCAAAGUGAAAUUUUUUAGUUAUAAACAAUUUUUGUCAUGUUGGUAGAAUUUUGUUUUACUUCAAUAUGAUUUUUUUCAUUUUUGAAAUAAAUAAAAUUCGAUUAAAUCAACGCUGACAUUUAAAAAAAAUUGUUUAAAGAAAUACUAAAUUAUUAAUUAAUUGAUCAAUUC**

**Halictophagus sp.(3) GAUUCAGUUAUUCAAAAGAAUUUAAUACUAUUAAAUCUUUGAAAUUUAUAAAUUUUAAAUCUGGUUCAUGUUCCGUUAAAAUUUUUCGUAAAAAAUUUUUUUCUCGAAAAAUGGAACUUUUUUUCUAUUACUUUAGAAAAAAAACCGAUUUAAUUAUUAAUUUUAUAAAUUUCUUUACAAUAACUGUGUC**

**Halictophagus silwoodensis ?**

**Halictophagus sp.(2) sequence read runs out 🡨 AUUUUUUUUUUUAAUUAAAAAAAAACUCGUUUAAAAAUUAAUUUUAUAAAUUUCUUUACAAUAACGAAUC**

**Callipharixenos sp. GAUUUGCGUAUUAUUUGAAAGGAAUUUUCUCAAUUGCAGAAgUUGCUUUCGAAUUGAAAUUUUUUAGUUUAGGCAAUUGUCUCGCUCGACUAGAUUUACACCAGAAACGUUUAUCGUUCAAAGAUAAAUUUGUGCGCUCGCGCGUGCGCACAUGCAGUAUGUAUCGUGCGCACACUCGUUGUCGCCGUUUCGGUGAAAUCGCUUCGAUGUCGACGUGACGCUAAAAAAAUUGCUUAUUUAAAAUGCUAGAAUUUUUUAUAAAAAGAUGCGCAUUUC**

**Elenchus sp.(1) GAAUAAAUUAUUUUUAUAAAAAUUUUAUUUAAAAAAUAAAAAAAUAAAUUUUUCCAUAAAAUUUAUUAAUUUUUAUUUUUAUUACAUGAAUAAAUUAAAAUAUUUAUUC**

**Elenchus varleyi GAAUAAAUAAUUUUUGAAAAAAUUUUAUUUUAGAAAAUAAAUUAAAUUUUUUUAAUUAAAAAUUUAAUUAUUUUCAUGAUAUAAAUAACAUAAAAAANUUAUUUC**

**Elenchus koebelei ?**

**16S rRNA template alignment**

**H183 H183’ RAA1 H255b H255b’ RAA2 RAA3 H533 H533’ H563 H563’ H579 RAA4 H671a H671b**

**(( .... )) ...... (( .... )) ...... ............. ..... ((( ....... ))) ... ((( ........ ))) . (((((( ((.( ... ((((((**

**Periplaneta fulig' ||--| [----] |--|| [----------------] ------ ||--| [----] |--|| ------ [--------------------------] UUACUGUAUUGGA [UAUUUUAUUAAUUUUUAG-----------------] AAGUA ||AAU| [UUG--UU] |AUU|| GUA ||CCU| UGUGUAUC |AGG|| G ||UUUAUU| [AAAUUAUAUUAUAUAUUUUUUGUUU--] ||CCCG| AUU |UUAAAG|**

**Tamolanica tamolana ||--| [----] |--|| [----------------] ------ ||--| [----] |--|| ------ [--------------------------] GUACUGUAAAGGA [AGAAUCAUGUAAGUUUUAUAAUCAAUAA-------] AAGGU ||AAU| [UUUAUUU] |AUU|| GUA ||UCU| UGUGUAUC |AGA|| G ||UGUAUU| [UAAAUGGAUUAUAAAAUUUUUUUCU--] ||CUCG| AAU |UUAAAA|**

**Reticulitermes fla’ ||--| [----] |--|| [----------------] ------ ||--| [----] |--|| ------ [--------------------------] GUACUGUAGGGGA [GUAUUGAUUUAUUUUUAG-----------------] AAGUU ||UAC| [UUUUUUU] |GUU|| GUA ||CCU| UGUGUAUC |AGG|| G ||UUUACU| [GAAUUUUAUUAUUUAUUUUUAUUU---] ||CCCG| AUU |CUAAAA|**

**Antheraea pernyi ||--| [----] |--|| [----------------] ------ ||--| [----] |--|| ------ [--------------------------] GUAAUGUAAAAGA [AUUUUGAAAUAAUUUGAAAAAAAAUUUUUUAAA--] AAGUA ||AAU| [UUAAUUU] |AUU|| GUA ||UCU| UGUGUAUC |AGA|| G ||UUUAUU| [AAAAAUUUUUUAAAUUUUUAAAUUU--] ||CUCG| AAU |UUAAAA|**

**Bombyx mori ||--| [----] |--|| [----------------] ------ ||--| [----] |--|| ------ [--------------------------] GUAUUGUGGGGGA [AUUUGAAAUAAUAUGAAAUUUAAUUAUUUUAA---] AAGUA ||AAU| [UUUAUUU] |AUU|| GUA ||UCU| UGUGUAUC |AGA|| G ||UUUAUU| [AAAAAUAAUUUUAUUUUUAAAGUUU--] ||CUCG| AAU |UUAAAA|**

**Haematobia irritans ||--| [----] |--|| [----------------] ------ ||--| [----] |--|| ------ [--------------------------] GUAUUGUGAAAGA [AAAUUGAAAUAAUUUGAAAAAUUAUUAUUUUAA--] AAGAA ||AAU| [UUAAUUU] |AUU|| GUA ||CCU| UGUGUAUC |AGG|| G ||UUUAUU| [AAAUAAAAAUUAAUUAAUUUAAUUUU-] ||CUCG| AUU |UUAAAA|**

**Ceratitis capitata ||--| [----] |--|| [----------------] ------ ||--| [----] |--|| ------ [--------------------------] GUAUUGUGAAAGA [UAAUUGAAAUAUUUUGAAAAAUUUUUAUUUAAA--] AAGAA ||AAU| [UUUAUUU] |AUU|| GUA ||CCU| UGUGUAUC |AGG|| G ||UUUAUC| [AAAUAAUUAAUAAUUAUUUAUUAAU--] ||CUCG| AUU |UUGUAA|**

**Tribolium castaneum ||--| [----] |--|| [----------------] ------ ||--| [----] |--|| ------ [--------------------------] GUAAAUGGUCUGU [GAAGGAUUUAAUUUUUUAGGGAGA-----------] AAGCA ||UAG| [UUGAUUU] |UUA|| GUA ||CCU| UUAGUAUC |AGG|| G ||UUUAUU| [AAAUUGGGGUCAAAUAAUUGGUUGC--] ||CUCG| AAU |UUAAAA|**

**Rhagophthalmus ohb' ||--| [----] |--|| [----------------] ------ ||--| [----] |--|| ------ [--------------------------] GUAUUAGUAUAGA [UAUAGAAUUAAUUUUUAAAUUUUUAA---------] AAGAA ||AAA| [UUUACUU] |UUU|| GUA ||CCU| UGUGUUUC |AGG|| G ||UUGAUU| [AAUUUUAAAUUAAAUAUUUAAUUAU--] ||CCCG| AUU |AUAAAA|**

**Mengenilla australiensis ||AU| [AAUA] |AA|| [AAUAUUUUUU-UGUUU] UAGUA- ||UU| [UUU-] |UA|| GAAA-- [UAAUA-AAUUUAAUGAUAGAUAAAAA] GUAUUGUAAAAGA [AAAUUAAAAUUUAUUAA-A----------------] AAGAA ||AAU| [UUUAUUU] |AUU|| GUA ||UCU| UGUGUAUC |AGA|| G ||UUUAUU| [AAUAAUAAAUUAGUUAAUUAAUUAU--] ||CUUG| AAA |UUUUAG|**

**Mengenilla chobauti ||UU| [AAUA] |AA|| [AAUAUUUUUUUUAUUU] UAGUA- ||UU| [UAU-] |AA|| GAAA-- [AAAUA-AUUAUUAUGAUAGAGAAAUA] GUAUUGUAAAAGA [AUAUUAUUAAUUAUAAA-A----------------] AAGAA ||UAU| [UUUAUUA] |AUA|| GUA ||UCU| UGUGUAUU |AGA|| G ||UUUAUU| [AAAAAUAAGUUAUAAAUAUAAUUAU--] ||CUUG| AAA |UUUUAG|**

**Triozocera sp.(2) ||UU| [UAUA] |AA|| [--AAUUUUUUUAUGAU] UAGUU- ||UU| [----] |AA|| AAAA-- [UUAUA-AUUAUUUAUAG--UAAAUUA] GUAUUGAGAAAUA [AUUUUAGUUUUAUUUUU-A----------------] AAGAA ||AAU| [UUUAUUA] |AUU|| GUA ||UCU| UGUGUAUC |AGA|| G ||UUUAUU| [AAAUUUAAUUUAUUAAUAUAAAAAU--] ||UUCG| AAA |UUUUUU|**

**Triozocera sp.(4) ||AU| [UAUA] |AA|| [--AAUAUUUUUAUGAU] UAGUU- ||UU| [----] |UA|| AAAA-- [UUAUU-UUUAUUUAUAG--UGGAUUA] GUAUUGAAAAAUA [AUUUUAAUUUUAUUUUU-U----------------] AAGAA ||AUU| [UUUAUUA] |AUU|| GUA ||UCU| UGUGUAUC |AGA|| G ||UUUAUU| [AAAUUAAAUUUAUUUAAAUAUAAAAAU] ||UUCG| AAU |UUUUUU|**

**Triozocera sp.(3) ||AU| [UAUA] |AA|| [--AAUAUUUUUAUGAU] UAGUU- ||UU| [----] |UA|| AAAA-- [UUAUU-UUUAUUUAUAG--UGGAUUA] GUAUUGAAAAAUA [AUUUUAAUUUUAUUUUU-U----------------] AAGAA ||AUU| [UUUAUUA] |AUU|| GUA ||UCU| UGUGUAUC |AGA|| G ||UUUAUU| [AAAUUAAAUUUAUUUAAAUAUAAAAAU] ||UUCG| AAA |UUUUUU|**

**Corioxenos acucyrt’ ||??| [????] |??|| [????????????????] ?????? ||??| [????] |??|| ?????? [??????????????????????????] ????????????? [???????????????????????????????????] ????? ||???| [???????] |???|| ??? ||???| ???????? |???|| ? ||??????| [???????????????????????????] ||????| ??? |??????|**

**Myrmecolax sp.(4) ||??| [????] |??|| [????????????????] ?????? ||??| [????] |??|| ?????? [??????????????????????????] ????????????? [???????????????????????????????????] ????? ||???| [???????] |???|| ??? ||???| ???????? |???|| ? ||??????| [????????????UUUAUUAUUUAAUUU] ||UUCG| AAU |UCUUAA|**

**Myrmecolax sp.(1) ||??| [????] |??|| [????????????????] ?????? ||??| [????] |??|| ?????? [??????????????????????????] ????????????? [???????????????????????????????????] ????? ||???| [???????] |???|| ??? ||???| ???????? |???|| ? ||??????| [??????????AUUAAUUAUUUUAAUUU] ||UUCG| AAU |UUUUAA|**

**Myrmecolax sp.(2) ||??| [????] |??|| [????????????????] ?????? ||??| [????] |??|| ?????? [??????????????????????????] ????????????? [???????????????????????????????????] ????? ||???| [???????] |???|| ??? ||???| ???????? |???|| ? ||??????| [???????????????AUUAUUUAAUUU] ||UUCG| AAU |UCUUAA|**

**Myrmecholax incautus ||UU| [UAUA] |AA|| [--GUUAAUUUUAUUUU] UAGUA- ||UA| [AUU-] |AA|| GAAA-- [UAAUU--------AUUUUAAUUUUUA] GUAUUGUAAAAGA [AAUAAUUUAAUUAUUAA-U----------------] AAGAA ||UAU| [UUUAUUU] |AUA|| GUA ||UCU| UUUGUAUC |AGA|| G ||UUUAUU| [AAAUAUUUUUUUAAAUAUUUAAAAUU-] ||UUCG| AAU |UUUUAA|**

**Myrmecholax sp.(5) ||??| [????] |??|| [????????????????] ?????? ||??| [????] |??|| ?????? [??????????????????????????] ????????????? [???????????????????????????????????] ????? ||???| [???????] |???|| ??? ||???| ???????? |???|| ? ||??????| [?????????????UUAUUAUUUAAUUU] ||UUCG| AAU |UCUUAA|**

**Caenocholax sp.(1) ||??| [????] |??|| [????????????????] ?????? ||??| [????] |??|| ?????? [??????????????????????????] ????????????? [???????????????????????????????????] ????? ||???| [???????] |???|| ??? ||???| ???????? |???|| ? ||??????| [???????????UUUAAUUAUUUAAUAU] ||UUCG| AAU |UUUUAA|**

**Caenocholax sp.(3) ||UU| [UAUA] |AA|| [--UUUAUUUUUGAUUU] UAGUA- ||UU| [----] |AA|| GAAA-- [AAAUU-------AUUUUUAUUUUUAA] GUAUUGUAAAAGA [AGUAAUUUUAAUUAAUUAA----------------] AAGAA ||UAU| [UUAAUUU] |AUA|| GUA ||UCU| UUUGUAUC |AGA|| G ||UUUAUU| [AAAAUAAAUUUAAAUAUUUAAAUUU--] ||UUCG| AAU |UUUUAA|**

**Lychnocolax sp.(2) ||AU| [UAUA] |AA|| [-AAUAAUUUUUAAUUU] UAGUA- ||UA| [UUU-] |UA|| GAAA-- [UAAUU AAUGAAAUUAUAGAGAAUUU] GUAUUGUAAAAGA [UUUUAUUAUAUUUAAAAAA----------------] AAGAA ||UAU| [UUUAAUU] |AUA|| GUA ||UCU| UGUGUAUC |AGA|| G ||UUUAUU| [AAUAAUUAAUUAAAAAUUUAUUAAU--] ||CUCG| AAA |UUUUAG|**

**Xenos hamiltoni ||??| [????] |??|| [????????????????] ?????? ||??| [????] |??|| ?????? [??????????????????????????] ????????????? [???????????????????????????????????] ????? ||???| [???????] |???|| ??? ||???| ???????? |???|| ? ||??????| [????????????????????UAUUUAU] ||CUCG| AAA |UUUUA-|**

**Xenos moutoni ||UU| [AAUU] |GA|| [---AAUUUAUUAUUAU] UAGUAU ||UG| [AGUA] |UA|| GAAA-- [AUAUU-UUUUUUAUGAAAGUUAAUUA] GUAAUGUAAAUGA [AUUAUUUUAAUAUUUGA-A----------------] AAGAA ||UAU| [UUUAUUU] |AUA|| GUA ||UCU| UGUGUAUC |AGG|| G ||UUUAUU| [UAUAAUAAUUUAUUGAUUUAAAAAU--] ||CUCG| AAA |UUUUAA|**

**Xenos vesparum ||UU| [UAUA] |GA|| [---AAUUUUUUAUUUU] UAGUA- ||UG| [AUA-] |UA|| GAAA-- [AAUUUUUUUUUAUGAUAGAUUAAUAU] GUAAUGUAAAUGA [AUAGUUUAUUUGUUUAA------------------] AAGAA ||UAU| [UUUAUUU] |AUU|| GUA ||UCU| UGUGUAUC |AGA|| G ||UUUAUU| [UAAAAUAAUUUAAUUAUUUAAAAAU--] ||CUCG| AAA |UUUUAA|**

**Xenos pecki ||??| [????] |??|| [????????????????] ?????? ||??| [????] |??|| ?????? [??????????????????????????] ????????????? [???????????????????????????????????] ????? ||???| [???????] |???|| ??? ||???| ???????? |???|| ? ||?????U| [AAUAAUAAAUAUUAAUAAUAUUUAU--] ||CUCG| AAA |UUUUAA|**

**Xenos sp. ||UU| [AAUA] |AA|| [---AGUAUUUUAUUUU] UAGUA- ||UU| [AUU-] |AA|| GAAA-- [AAUUU-AUUAGUAUGAUAGUGAAAUU] GUAAUGUAAAUGA [AUAAAUUUAUUUAAUAA-A----------------] AAGAA ||UAU| [UUUAUUA] |AUU|| GUA ||UCU| UGUGUAUC |AGA|| G ||UUUAUU| [AAAAUUUAUUUAAUUAUUUAAAGAU--] ||CUCG| AAA |UUUUAA|**

**Paraxenos sp. ||UU| [UAUU] |GA|| [---AGUAGCUUGUGUU] UAGUA- ||UG| [GGU-] |UA|| GAAAC- [AAAAU- AAUUAAUUAUAGAGAAUGA] GUAUUGUUAAAGA [AUUAAAUAAAUUUAUUAGA----------------] AAGAA ||UAU| [UUUAAUU] |AUA|| GUA ||UCU| UGUGUAUU |AGA|| G ||UUUAUU| [AAAAUUUUUUAAGGAUUUAGAUGU---] ||CUCG| AAA |UUUUA-|**

**Pseudoxenos sp. ||UU| [UAUU] |GA|| [---AUUAUAUUUUAUU] UAGUA- ||UA| [AUU-] |AA|| GAAAAA [AGUAU---UUUUAUAAUAGAGAAUUA] GUAGUGUAAGUGA [AAUAGAUGAAUUAUUUA-A----------------] AAGAA ||UAU| [UUAAUUU] |AUU|| GUA ||UCU| UGUGUAUC |AGA|| G ||UUUAUU| [AAAAAUAUUUUAAAAAUUUUAAAUAU-] ||CUCG| AAA |UCUUAA|**

**Stylops mellitae ||UU| [UAUA] |GA|| [---AAUAAUUUUUUAU] UAGUA- ||UA| [UAUA] |AA|| GAAA-- [AUAAA AAUUUUAUGAUAAAGAAUAA] GUAUUGUAAAAGA [AUUAAUUAAUUUAAUUAAA----------------] AAGAA ||AAU| [UUAAUUU] |AUU|| GUA ||UCU| UGUGUAUC |AGA|| G ||UUUAUU| [UAAAAUAUAUUAAAAAUUUAAUGAU--] ||CUCG| AAU |UAUUAA|**

**Tridactylophagus sp. ||UA| [UAUA] |AA|| [----UAAAUUUAUUUU] UAGUAU ||UA| [AAU-] |UA|| GAAA-- [AAUUU-UUAAAAUUUAUAAAGUAUUA] GUAUUGUAAAAUA [AUUUAUUUAAUUAAAAA------------------] AAGAA ||UAU| [UUUAUUA] |AUA|| GUA ||UCU| UGUGUAUC |AGA|| G ||UUAAUU| [AAAUUUUAUUUAUAAAUAUAAAUAU--] ||UUUG| AAU |UUUUUU|**

**Halictophagus calcaratus ||--| [----] |--|| [-----------?????] ?????? ||??| [????] |??|| ?????? [?????---UUAAAAUAAUUUUAUUUU] UAUUUGUAAAUUA [GUUAAUUUAUUAGUUUGU-----------------] ----- ||UAU| [-UAUUAA] |AUA|| GUA ||CCU| UUUGUAUU |AGG|| G ||U-AAUU| [UAAUUUAUUAAAUUAUUUAUAUU----] ||CCCG| AAU |UGAGAU|**

**Halictophagus sp.(3) ||??| [????] |??|| [????????????????] ?????? ||??| [????] |??|| ?????? [??????????????????????????] ????????????? [???????????????????????????????????] ????? ||???| [???????] |???|| ??? ||???| ??????UC |AGA|| G ||U-AAUU| [AAAAAUAAUUUAAUUAGUUAAUAU---] ||UUUG| AAU |UUUUUU|**

**Halictophagus silwoodensis ||--| [----] |--|| [-----------UUUAU] UAGUA- ||UU| [UUU-] |AA|| GAAC-- [UUAAU--------------UUUUAAA] GGACUGUAAAGGU [UUUAAUUUUAAAUUAUA------------------] ----- ||UAA| [GAAUUAU] |UUA|| GUA ||CCU| UUUGUAUC |AGG|| G ||UUAAUU| [UAAUUAUUUAAUUAUUUUUGUAU----] ||CCCG| AAC |UGAAUU|**

**Halictophagus sp.(2) ||--| [----] |--|| [-----------AUUUU] UAGUA- ||UA| [UAU-] |AA|| GAAA-- |AAUUU-AAUUUCUUAAUAGUAUAAUA] GUAUGGUAAAAGA [AUAA--UUUUAAUUUUAUA----------------] AAGAA ||UAU| [UUUAUUA] |AUA|| GUA ||UCU| UUUGUAUC |AGA|| G ||UUAAUC| [AAUAAUAAUUUAAUUAUUUAAAUAU--] ||UUUG| AAU |UUUUUU|**

**Elenchus koebelei ||UU| [UAUA] |AA|| [--AGUUGUUUUUUGUU] AAGUA- ||AU| [AUU-] |UU|| GAAA-- |AACUU---------AAAUUAAUUAUU] GUAUUGAAAAAGA [AUUUUAUUUAAUUGUAAAU----------------] AAGAA ||UAU| [UUAAUUA] |UUA|| GUA ||UCU| UUUGUAUC |AGA|| G ||UUUAUU| [AAAAUUAAAUUAAAUAUUUAUUAAU--] ||UUCA| AAU |UUUUAA|**

**Elenchus sp.(1)**  **||UU| [UAUA] |AA|| [--AGUUGUUUUUUGUU] AAGUA- ||AU| [AUU-] |UU|| GAAA-- |AACUU---------AAAUUAAUUAUU] GUAUUGAAAAAGA [AUUUUAUUUAAUUGUAAAU----------------] AAGAA ||UAU| [UUAAUUA] |UUA|| GUA ||UCU| UUUGUAUC |AGA|| G ||UUUAUU| [AAAAUUAAAUUAAAUAUUUAUUAAU--] ||UUCA| AAU |UUUUAA|**

**H687 RAA5 H736 H736’ RAA6 H687’ H777 H777’ H671b’ H671a’ H812 H822 H822’ H837 RAA7 H837’ H946 H946’**

**...... ((((( (((( ........ )))) ))))) .. ((( ..... ))) ...... )))))) ....... ).)) .. (((((( .... ((((( ... ))))) .. (((( )))) .... (((((((((( ........ ))))))))))**

**Periplaneta fulig' ---GAU ||UUAAA| [UAAAUAUUUUAGU--] |UAUU| GUUUCAUA |AAUA| [UUAAUAAUAUUUG-] |UUUGG|| U- ||AGU| GAAAU |AUU|| AGUCGA |CUUUAA| -UGGUGU |CUGG|| UU ||UUUCAA| GAAA ||UAAAU| UUA |AUUUA|| -- ||----| [AAUAUUAAUAAUUAUAUUUCUUUCUUUUUAGUUUUGUAUUUUAUUAAUA] |----|| UUAA ||UAUUAUGGGG| --GCUAAG |CUCUUUAAUA||**

**Tamolanica tamolana ---GAU ||UUAAU| [UAAAAAUAUUAGU--] |UAUU| GUUGCAUA |GAUA| [UUAAUAAUUUUUA-] |AUUGG|| U- ||AAU| GAAAC |GUU|| AUUCGU |UUUUAA| -GGAUAU |CUAG|| UU ||UUUUAA| UAAA ||UGAAU| UUA |AUUCA|| -- ||----| [GAAAAUAUAAUUUAUUAAAUAAUUGAUUAUUAAAAUUUAAUUCUUUAUU] |----|| ---- ||UA---GGUGA| UUUAUGAA |CUUUUAUUUA||**

**Reticulitermes fla’ --GGGG ||CUAUU| [AAUUUAUUUUAGU--] |UGUU| GUUUUAUU |AACA| [UUAAUAAUGGGUU-] |AAUAG|| U- ||GAU| GAAAU |GUU|| AUUCGU |CUUUAG| -GGGUUU |CUGG|| UU ||UUUCAA| GAAA ||UGAAU| UUA |AUUCA|| -- ||----| [UGCGUCUUAUUUAGAGUUUUAUUAUUUUAUUAUUUAUUUUUAUUUGGCG] |----|| UUAA ||GAUUGGGAGG| --GAUUAG |CUUCUUAUUU||**

**Antheraea pernyi ---GAG ||UUAAU| [UAAUUAAUAAAGU--] |UAUU| GUGGCAUA |AAUA| [UUUUUAAUAAUUA-] |AUUAG|| A- ||AAU| GAAAU |GUU|| AAUCGU |UUUUAA| -AUAUAU |CUAG|| UU ||UUUUUA| GAAA ||AAAAU| UUA |AUUUU|| U- ||AAUU| [UAAUUUAAAAUUAAAAUAAAUAUUUAUUUUAAUUUUAAAUUA-------] |AAUU|| -AAA ||UAAUUUAAGG| --GAUAAG |CUUUGAAUUA||**

**Bombyx mori ---GAG ||UUAAU| [UAAUUAAUAAUAAGU] |UAUU| GUAGUAUA |AAUA| [UUUUAAAUAAUUA-] |AUUUG|| A- ||AAU| GAAAU |GUU|| AUUCGU |UUUUAA| -AUAUAU |CUAG|| UU ||UUUAUA| GAAA ||AAAAU| UUA |AUUUU|| U- ||UAUU| [AAUUAGAUUUUUUAUUAAUUAAAUUAAUAAAAAUAUAUUUUU-------] |AAUA|| -AAA ||UAAUUUAAGG| --GAUAAU |CUUUAAAUUA||**

**Haematobia irritans ---GAG ||UUAAU| [AUAUUAUAAAAGU--] |UAAU| GUGAUAAA |AUUA| [UUUUUAAUAAUAU-] |AUUAG|| A- ||AAU| GAAAU |GUU|| AUUCGU |UUUUAA| -AGGUAU |CUAG|| CU ||CUUUAA| GAAA ||UAAAU| UUA |AUUUA|| GA ||AAUU| [UUAUAUUAUUUAAUUAGUUAAAUUAAUUAAAUAAAUAUUU---------] |AAUU|| UUAA ||UAUUUUACGG| --GAUAAG |CUGUAAAAUA||**

**Ceratitis capitata ---GAG ||UUAAU| [AAUUUAUUAAAGU--] |UAAU| GUGUUAAA |AUUA| [UUUUAAAUAAAUU-] |AUUAG|| A- ||AAU| GAAAU |GUU|| AUUCGU |UUAUAA| -AGGUAU |CUAG|| UU ||UUUUUA| GAAA ||UAAAU| UUA |AUUUA|| C- ||AAUU| [UUUUAAUUUUUUUGUUAUUUAUUUAAUAAAAAAAUUUAUAA--------] |AAUU|| -GAA ||UAUUUUAAGG| --GAUAAG |CUUUAAAAUA||**

**Tribolium castaneum ---GAG ||CUAAA| [AUAAU-AUAUUUAU-] |UACU| GUGGAAAA |AGUA| [UUUAUAAUAUUAU-] |UAUAG|| U- ||AAU| GAAAC |GUU|| AGUCGU |UUUUAA| -AUAUAU |CUAG|| UU ||UUCUGA| GAAA ||UAAAU| UUA |AUUUA|| U- ||UUAU| [UUUUUAUUAUAAUUAUAAUUUUUUAUUUUAUGAUUAAA-----------] |AUAG|| AAUA ||UUUUUAUAGG| --GAUAAG |CUUAUAAAAG||**

**Rhagophthalmus ohb' ---GAG ||CUAAU| [UUUUU-AUUUAUUU-] |AAUU| GUUGAAAA |AAUU| [UUUUAAAUAAGAA-] |GUUUG|| A- ||AAU| UAAAU |GUU|| AUUCGU |UUUUAU| -AGAUAU |CUGG|| UU ||UUUAAA| GAAA ||AGAAU| UUA |AUUUU|| U- ||UAAU| [UUUUUUAUAAACAUUUAAUUUUUAGUUGUUUAUUUUUA-----------] |AUUU|| GAAA ||UAUCUAAUGG| --GAUGAG |CUUUUAGAUA||**

**Mengenilla australiensis ---GAG ||UUAUA| [AUUAUUU--AAUUU-] |UAAU| GUUUUAAA |AUUA| [AAAUAAAAAUAUU-] |UAUGG|| U- ||AAU| GAAAA |GUU|| AAUCGA |UUAAAA| -UUAUAU |CUAG|| UU ||AUUUUG| GAAA ||UUAAU| UUA |AUUAA|| UA ||UUAU| [AAAUUAAUUUAUUAAAAUUUAAUUUUUAUUAAA-UUAUUU---------] |AUUA|| UAAA ||UAUAAAAAGG| --GAUAAU |UUUUUUUAUA||**

**Mengenilla chobauti ---GAG ||AAAUA| [AUUUUUA--AAUUU-] |UAUU| GUUAUAUA |AAUA| [AAAUAAAAAAAAU-] |UAUAG|| U- ||AAU| GAUAA |GUU|| AAUCGA |UUAAAA| -UUUUAU |CUAG|| UU ||AUUUUA| GAAA ||UUAAU| UUA |AUUAA|| G- ||UUAU| [UAAGAAAAUUUUAAUAAUAAUUAAUUUAUUAAAAUUAUUUA--------] |AUAA|| -AAA ||UAUAUAAAGG| --GAUAAG |CUUUUUUAUA||**

**Triozocera sp.(2) ---GAU ||CUAUA| [AGUAUUU-AUAAAU-] |UAAU| AUUAUAUA |AUUA| [UUAUAAAAAUAUU-] |UGUGG|| A- ||AGU| UAGAA |GUU|| AUUCGA |AAAAAA| -UAAUAU |CUUA|| UU ||AUUUUA| GAAA ||UUAAU| UUA |AUUAA|| U- ||UUAU| [AAAAAUUAUUAAAUUUAAAAAAAUUUUUAAUUUUAAUUUUUU-------] |AUAA|| -AAA ||AAUUUUUAGG| --GACAAG |CUUAAAUAUA||**

**Triozocera sp.(4) ---GAU ||CUAUA| [AAUAUUU-AAAAAU-] |UAAU| AUUUUAUA |AUUA| [UUAGUAAAGUAUU-] |UAUUG|| A- ||AAU| UAGAA |GUU|| AUUCGA |AAAAAA| -UGAUAU |CUAA|| UU ||AUUUUA| AAAA ||UUAAU| UUA |AUUAA|| U- ||UUAU| [AAAAAUUAUUAAAUCUAUAUUAAAUUUUAUAUUUAAUUUUUU-------] |AUAA|| -AAA ||AAUUUUUAAG| --GAUAAG |CUUAAAUAUU||**

**Triozocera sp.(3) ---GAU ||UUAUA| [AAUAUUU-AAAAAU-] |UAAU| GUUUCAUA |AUUA| [UUAAAAAAAUAUU-] |UAUUG|| A- ||AAU| UAAAA |GUU|| AUUCGA |AAAAAA| -UGAUAU |CUAA|| UU ||AUUUUA| AAAA ||UUAAU| UUA |AUUAA|| U- ||UUAU| [AAAAAUUAUUAAAUUUAUAUUAAAGUUUAUAUUUAAUUUUUU-------] |AUAA|| -AAA ||AAUUUUUAAG| --GAUAAG |CUUAAAUAUU||**

**Corioxenos acucyrt’ ---??? ||?????| [???????????????] |????| ???????? |????| [?????????????-] |?????|| ?? ||???| ????? |???|| ?????? |??????| ??????? |????|| ?? ||??????| ???? ||?????| ??? |?????|| ?- ||????| [?????????????????????????????????????????????????] |????|| ???? ||??????????| ???????? |??????????||**

**Myrmecolax sp.(4) ---GAG ||UUAAU| [UUAAUUUUAUAAUA-] |UAAU| GUUAUAUA |AUUA| [UUUUAAAAAUUAA-] |AUUAG|| A- ||AAU| GAAAA |GUU|| AUUCGU |UUAAGA| -AUAUAU |CUAA|| UU ||UAUUUA| GAAA ||UUAAU| UUA |AUUAA|| U- ||UUAU| [UAUUAAUUUAUAUUUUUAUUUAAUUUUUAAUUUUUA-------------] |AUAA|| -AAA ||GAUUGAUCGG| --GAUAAU |UUGAUUAAUU||**

**Myrmecolax sp.(1) ---GAG ||UUAAU| [UUUAUUUUAUAUUA-] |UAAU| GUUUUAUA |AUUA| [UGUUAAAAAUAAA-] |AUUUG|| A- ||AAU| GAAAA |GUU|| AUUCGU |UUAAAA| -AUAUAU |CUAA|| UU ||UAUUUA| GAAA ||UUAAU| UUA |AUUAA|| U- ||UUAU| [UAUUAAUUUAUUUAUUUAUUAUAUUAUUUAAUUAUUA------------] |AUAU|| -UAA ||GAUUAAUCGG| --GAUAAU |UUGAUUAAUU||**

**Myrmecolax sp.(2) ---GAG ||UUAAU| [UUAAUUUAACAAUA-] |UAAU| GUUUUAUA |AUUA| [UUUUAAAAAUUAA-] |AUUAG|| A- ||AAU| GAAAA |GUU|| AUUCGU |UUAAGA| -AUAUAU |CUAA|| UU ||UAUUUA| GAAA ||UUAAU| UUA |AUUAA|| U- ||UUAU| [UAUAAAUUUAUAUUUUUAUUUAAUUUUUAAUUUUUA-------------] |AUAA|| -AAA ||GAUUGAUCGG| --GAUAAU |UUGAUUAAUU||**

**Myrmecholax incautus ---GAG ||UUAAU| [UUAAUUUUAUAUUA-] |UAAU| GUUAUAUA |AUUA| [UAUUAAAAAUUAA-] |AUUAG|| A- ||AAU| UAUAA |GUU|| AAUCGU |UUAAAA| -AUUUAU |CUAA|| UU ||UAUUUA| GAAA ||UUAAU| UUA |AUUAA|| U- ||UUAU| [UAUUAAUUUAAUUUAUUUAAUUAAAUAUAAAUUAGUA------------] |AUAA|| -AAA ||GAUUGAUCGG| --GAUAAU |UUGAUUAAUU||**

**Myrmecholax sp.(5) ---GAG ||UUAAU| [UUAAUUUGAUAAUA-] |UAAU| GUUAUAUA |AUUA| [UUUUUAAAAUUAA-] |AUUAG|| A- ||AAU| GAAAA |GUU|| AUUCGU |UUAAGA| -AUAUAU |CUAA|| UU ||UAUUUA| GAAA ||UUAAU| UUA |AUUAA|| U- ||UUAU| [UAUUAAUUUAUUUUUUUAUUUAAUUUUUAAUUUUUA-------------] |AUAA|| -AAA ||GAUUAAUCGG| --GAUAAU |UUGAUUAAUU||**

**Caenocholax sp.(1) ---GAG ||UUAAU| [UUAAUUUUUAUAAU-] |AAAU| GUUGUAAA |AUUU| [AUUAAAAAUUUAA-] |AUUGG|| A- ||AAU| AAUAA |AUU|| AAUCGU |UUAAAA| -AUAUAU |CUAA|| UU ||UUUUUA| GAAA ||UUAAU| UUA |AUUAU|| U- ||UUAU| [UAUUAAUUUAAAAUUUUUAAUAAGUAAAUUUUUA---------------] |AUAA|| -GUA ||AAUUAAUCGA| --GAUAAU |UUGAUUAAUU||**

**Caenocholax sp.(3) ---GAG ||UUAAU| [UUAAUU-AUUAUUU-] |UAAU| GUUUUAAA |AUUA| [AUAUAAAAAUUAAA] |AUUAG|| A- ||AAU| GAAAA |GUU|| AAUCGU |UUAAAA| -AUAUAU |CUAA|| UU ||UCUUUA| GAAU ||UUAAU| UUA |AUUAU|| U- ||UUAU| [UAUUAAAUUUAUUUAUUAUUUUAAAAAUUUUUA----------------] |AUAA|| -GAA ||AAUUAAUCGG| --GAUAAU |UUGAUUAAUU||**

**Lychnocolax sp.(2) ---GAA ||AUAAA| [UUUAUUU-AUUUAU-] |AAAU| AUUUUAUA |AUUU| [AAUAAUAAAUAAA-] |UUUAG|| A- ||AAU| GAUAA |GUU|| AAUCGU |UUAAUA| -UAAUAU |CUAG|| AA ||AUUUUA| GAAU ||UUAAU| UAA |AUUAA|| U- ||----| [UAAAAUUUAAAUUAUUAAAUAAUUUAAAUUUAAUAAUUUAAAUUUUA--] |----|| -UAA ||AAUUAAUAGG| --GAUAAU |UUUAUUAAUU||**

**Xenos hamiltoni ---GAG ||UUACA| [UUAAUUUA--UUAA-] |AAAU| AUAAUAAA |AUUU| [UAAUUAAAUUUAA-] |UGUAG|| U- ||AAU| UAUAU |GUU|| AAUCGA |UUAAAA| -UAAUAU |CUAG|| UU ||UUUUUU| GAA- ||UAAAU| UAA |AUUUU|| U- ||----| [AUUAAUUUAAAUAAUUAAAUUAAAUUAAU--------------------] |----|| -UUA ||AUUAAUUAAG| --GCUUAG |CUUAAUUAAU||**

**Xenos moutoni ---GGG ||UUCUA| [UUAAAAUA--UUUU-] |AGAU| AUUAUAAA |AUCU| [UAAUUAAAUUUAA-] |UAUAG|| G- ||AAU| GAAAA |GUU|| AAUCGU |UUAAUA| -UUAUAU |CUAG|| UU ||UUUUUA| GAA- ||AAAAU| UUA |AUUUA|| U- ||----| [UUUAAUUUAUAUUAGAAUUUUAUAUUAA---------------------] |----|| -UAG ||AUUUAUUAGG| --GAUAAG |CUUAAUUAAU||**

**Xenos vesparum ---GAG ||UUAUA| [UUAUUAAA--UUAU-] |UAAU| GUUUUAUA |AUUU| [UUAUUAAGUUUAA-] |UAUAG|| A- ||AAU| GAAAA |GUU|| AAUCGU |UUAAAA| -UUAUAU |CUAG|| UU ||UUUUUA| GAG- ||UGAAU| UUA |AUUUG|| U- ||----| [UAUAAUUUAUAAAAUUGAUUUGAAUUAU---------------------] |----|| -UUA ||AUUUUUUAGG| --GAUAAU |CUUGAAAAAU||**

**Xenos pecki ---GAG ||UUACA| [UUAAUUUA--UUUA-] |AAAU| AUUAUAAA |AUUU| [UAAUAAAAUUUAA-] |UGUAG|| U- ||AAU| UAUAU |GUU|| AAUCGA |UUAAAA| -UAAUAU |CUAG|| UU ||UUUUUU| GAA- ||UUAAU| UAA |AUUUU|| A- ||----| [AUUAAUUUAAAUAGUUAUAUUAAAUUAAU--------------------] |----|| -UUA ||AUUAAUUAAG| --GCUUAG |CUUAAUUAAU||**

**Xenos sp. ---GAG ||UUAUA| [UUAAUAAA--UUAU-] |UGAU| GUUUUAAA |AUUU| [UAAUUAAAUUUAU-] |UAUAG|| A- ||AAU| GAUAA |GUU|| AAUCGU |UUAAAA| -UUAUAU |CUAG|| UU ||UUUUUA| GAA- ||UAAAU| UUA |AUUUU|| U- ||----| [AAUAAUUUAAAUUAUGUUUAAAUUUU-----------------------] |----|| -UAA ||AUUUUUUAGG| --GAUAAG |CUUAAAGGAU||**

**Paraxenos sp. ---GGG ||GUAUA| [UUUAAUUA--UUGU-] |UAAU| GUUUUAUA |AUUA| [UAAUUAAGUUAAA-] |UAUAG|| G- ||AAU| GAAAA |GUU|| AAUCGU |UUAAAA| -UUAUAU |CUAG|| UU ||UUUUUA| GAA- ||AAAAU| UUA |AUUUU|| G- ||----| [UUUAAUAUAUAAUUGAUGUAUUUAGUUAAUAAAUUUAUAUUAUG-----] |----|| -AUA ||AUUUUAUAGG| --GAUAAG |CUUAUGAAAU||**

**Pseudoxenos sp. ---GGG ||UUAUA| [UUUAAGUU--UUAU-] |UAAU| GUUUUAUA |AUUA| [UAGAGAAGUUAAA-] |UAUGG|| U- ||AAU| GAAAA |GUU|| AAUCGG |UUAAGA| -UAAUAU |CUAG|| UU ||UUUUUA| GAAU ||UAAAU| UUA |AUUUA|| U- ||----| [UUUAAUUUAUAAUUUAGGUUUAGUUUAAUAAAUUUAAAUUAAU------] |----|| -UUA ||UUCUUUAAGG| --GAAAAG |CUUUAGAGAA||**

**Stylops mellitae ---AAG ||UUAAA| [UUAAAUUU--UCAU-] |UAAU| AUUAUAAA |AUUA| [UGAGAAAAUUUUA-] |UUUAG|| A- ||AAU| UAAAA |GUU|| AAUCGU |UUAAUA| -AUAUAU |CUAG|| UU ||UUUUUA| GAAA ||UUUAU| UUA |AUUAA|| G- ||----| [UUAAUUUUAAUUUUUAUAAAAAAUUUUGUUUAUAAAAAUUAAUAUUAA-] |----|| -UAA ||AAUUUUUAGG| --GAUAAG |CUUAAAAAUU||**

**Tridactylophagus sp. -UUGAU ||CAAUA| [-AAAUUAAAAUAAU-] |UAAA| GUUUUAUA |UUUA| [UUAUAAAUAAUUU-] |UAUAG|| G- ||AAU| AAAAG |GUU|| AAUCGU |UAAAAA| -AUAUAU |CUAA|| UU ||UUUUUA| GAAA ||GUUAU| UUA |AUUAU|| U- ||----| [AAUUAAUUUAUUAUUAUUAUUAAUUAUGAUGAUAAUAAUAAAUUAAUU-] |----|| -AAA ||UAAUUUUAGG| --GAUAAA |CUUAAAAUUA||**

**Halictophagus calcaratus ---GAC ||CUAUU| [-UAGGUAUUAUUUU-] |AUUU| GUUUUAUA |AAAU| [UUAUUAAUACUUA-] |AUUUG|| U- ||AAU| UAUAA |GUU|| AUUCGA |UUCUCA| UUUAUAU |CUGG|| UU ||UCUUUA| GAU- ||UAAAU| UUA |AUUUU|| G- ||----| [GAAUAUUUUUAAUUUUAUUUAUUAUUUUUAUUC----------------] |----|| -UUA ||UUUUAUAAUG| --GAUAAU |CAUUAUAUUU||**

**Halictophagus sp.(3) UUUGAU ||UGAUA| [-AAAAUAAUUUAGU-] |UAAU| AUUUCAUA |AUUA| [UUAAAAAUAUUUU-] |UAUAG|| G- ||AAU| UAAAG |GUU|| AAUCGU |UAAAGA| -AUAUAU |CUAA|| UU ||UUUUAA| GAGA ||UUAAU| UGA |AUUAA|| U- ||----| [AAUUUAUAAAAAUUAAAAUUAUACUUGUUUAAUAAAAUUUUUUUAAAUU] |----|| -AAA ||AAUUUAAAGG| --GAUAAG |CUUUUAAAUU||**

**Halictophagus silwoodensis ---GAU ||CUAAU| [-UAUAAAUGGUUUU-] |AUUU| GUUGCAAA |AAAU| [UUAUUAAUUUGU--] |UUUAG|| U- ||AGU| AAAAA |GUU|| ACUCGU |UUUUCA| -UUAUAU |CUGG|| UU ||AUUGAG| GAU- ||AAAAU| UAA |AUUUU|| A- ||----| [GAUUUUUUUUUAAAAAAAAUUUGUUUAAUUAAAUC--------------] |----|| -UUA ||UAUUAUCGAG| --GAUAAU |CUCAAUAAAU||**

**Halictophagus sp.(2) ---GAU ||CAAUA| [-AAAAUAAUUUAGU-] |UAAU| GUUUCAUA |AUUA| [UUAAAAAUAUUUU-] |UAUAG|| G- ||AAU| UAAAG |GUU|| AAUCGU |UAAAAA| -AUAUAU |CUAA|| UU ||UUUUAA| GAAA ||UUAAU| UUA |AUUAA|| U- ||----| [AAUUUAUUAAAAUAGAAUUAUUUUUUUUUAUUAAAAUUUUAAUAAAUU-] |----|| -UAA ||GAUUUUUAGG| --GAUAAG |CUUAAAAAUU||**

**Elenchus koebelei ---GAU ||UUAUU| [-AGAUUAUUUUUGU-] |UAUU| GUUUUAUA |AAUA| [UAAAAAAUUUUUU-] |AAUUG|| A- ||AAU| UAAAA |GUU|| AAUCGU |UUAAAA| -AUAUAU |UUAA|| UU ||AUUUUA| GAAA ||UUAAU| UUA |GUUAA|| G- ||----| [AAUUAAUUAUUUAAUUAAAAUAAUUGUUAUUUUAGUUUAUUUUUAAUU-] |----|| -UUA ||GAUUAAUGGG| --GAUAAG |CUUGUUAAUU||**

**Elenchus sp.(1)**  **---GAU ||UUAUU| [-AGAUUAUUUUUAU-] |UAUU| GUUUUAUA |AAUA| [UAAAAAAUUUUUU-] |AAUUG|| A- ||AAU| UAAAA |GUU|| AAUCGU |UUAAAA| -AUAUAU |UUAA|| UU ||AUUUUA| GAAA ||UUAAU| UUA |GUUAA|| G- ||----| [AAUUAGUUAUUUAAAUAAAAUAAUUAUUAUUUUAUUUUAUUUCUAAUU-] |----|| -UUA ||GAUUAAUGGG| --GAUAAG |CUUGUUAAUU||**

**RAA8 - H991 H1057 H1057’ RAA9 - H991’ H812’ RAA10 - H1196 H579’ RAA11 – H1648a H1648b**

**(((..((( ............. )))..))) )))))) ..... )))))) ......... (((**

**Periplaneta fulig' [----------------------------------] ||--------| [-------------] |--------|| [-------------------------------------------------------------------] |UUGGAA|| [UUUUUUAUUUAAUUUUUAUUUAAUUAUA-----------] AUUAU |GAUGUA|| AUUA-GUAA [------UUAUUACUUUAUUUUUAAUUAUAUUUUGUUGAUAUUUUAAA] ||AAG|**

**Tamolanica tamolana [----------------------------------] ||--------| [-------------] |--------|| [-------------------------------------------------------------------] |UUAAAA|| [UAUAAUUAUAAUAUUGUGAGUUAAAGGAGGAUUUUAUGA] AUUUA |GAAUUU|| GUUA-GUGA [----UUAAAGAAUGUUAUUUUGUUAAAUUGGUUGAUAUGUUAGUAUG] ||AAG|**

**Reticulitermes fla’ [----------------------------------] ||--------| [-------------] |--------|| [-------------------------------------------------------------------] |UUGAAA|| [UGUGUUCUUUAUCUUUAAUUUUGGUUAGUA---------] AUUAU |UGUAGA|| AUUA-GUAA [------AUUUGUUAUUAGGUUGUUUUUUUUGGUGUUAAUUUCUUUUG] ||AGG|**

**Antheraea pernyi [AAAUUUUAUAAUGAAUAUUAAAAUUAUUUAAAAA] ||UUUUAUGA| [UUUAAAUUA----] |UUAAUAAA|| [UUUUAUUUUAUUAUAAAUAAUUUUAAAUUUAAAUAAAAUUUAAUUUAAUUUAUUUAAAUUUUUA---] |UAAAAA|| [AAUUUAUAUUAAUGGGGGAU-AUAAAGAA----------] AUUAU |GAUAAA|| AUUA-GUAA [------AAAUAAUAAUAAUAAUAAGUAAAAUUUUUAAGUUAUUUUUA] ||AGG|**

**Bombyx mori [AAAUUUUAUAAUAAUUUUAUUAAAAAAGAAAU--] ||UUAUAUAA| [UUUAUAUUG----] |UUAAUAAA|| [UUUUAUUUUAUUAUAAAUAAUUUUACUAAUAGAAAAAUUUAAUUUUAUUUAAUUUUUUAUA------] |UAAAAA|| [---UAAAUUUUAAUUUAAUAAAUUAAAUU----------] AUAAU |GAUAAA|| AUUA-GUAA [AAUUUAUUAAUUAAUAAAAUUAUUAAUUUAUUUAAUUAAUUAUAAAA] ||AGG|**

**Haematobia irritans [AAUUUUUAAAAAUAUUUAAAUAAAAUAAUAA---] ||AUAUAGAU| [UUAGAAAUA----] |GUUAUUAU|| [UAAUAAAAGUGUUAUAAUUUAUUUUAUAAAUUAAAUUAUUUAUUUAAAUUUUAAAUUUUUA------] |UUAAAG|| [-UUUUAAUUUUAAUUAAAAAAAUUAAAUA----------] AUAAU |GAUAAA|| AUUA-GUAU [-------AUAUUAUUGUAUAAAUAAAUAAAUAUGAAAAGUUUAUGUA] ||AAG|**

**Ceratitis capitata [AAUUUUUAAAAAUUAAUAAAAAUUUUAUAAAA--] ||AUGUAUGC| [UUAGAAUUA----] |GUAAUCAU|| [UAAUAAUUUUGUUAUAAAUUAUUUUUAAAAUAAUAUUAUUUAUUAUAUUUUAAUUUUUUA-------] |UAAAAA|| [-UUUUUUUAUAAAUGUUAUAUAAAAAGUA----------] AUAAU |GAUAAA|| AUUA-GUAU [-----AAAUUUAAAUGUUAAAAUAAAUUUAUUUGAUAAGUUUAUAUA] ||AAG|**

**Tribolium castaneum [GAUUUUCAUAAUUAAGUUGUCACUGAUUAAU---] ||UUAUAGGA| [UUAGAAUUU----] |UUCAUUAU|| [UUUUAGUAUGUUAUAGUUAAUUAAUCUAGUAGUGUGAUUUAAAGUUUAUUUGAAUUUUUUA------] |UCAGAA|| [UUUCUUAAAUAAUGAAGAAUUAUGA-GUA----------] AUAAU |GAUAAA|| AUUA-GUAU [------AAUUUUUUGUAAAAUUAAUUUUUAUUUGCUAGGUUAGUUUA] ||AGG|**

**Rhagophthalmus ohb' [UAUUUUUAAAAAUUAUUUUUUAUUUUUAUUAU--] ||UAAUAUGA| [UUAUUAUUG----] |UCGAUUUA|| [UUAAAUAAUUUAAUAGUUAUUAUUUUUUUUUAUAUUAUUAUUUAUAUUUUAUUUUAAUUUUUUUA--] |UUUAAA|| [UUUAUUAUUUAAUUUGUUUUUAUGA-GUA----------] AUAAU |GAUUAA|| AUUA-GUAA [-----AUAAUUUAUUUAUUAUAAUAAUAUUUAUUUUAGGUAAUUAAA] ||AGG|**

**Mengenilla australiensis [UUAUUAAAAAUGUUAAUAAAAUAAAUAAU-----] ||UUGUUAGA| [UUAGUAUUA----] |UUUAUCAA|| [----UAAUAAUUUUUAUUAAAGAAUUAUUUUUUGUAAAAAAUUUAUUAAUAUUUUAAUUUAUUUA--] |UAAAAU|| [UGAAUUAUUUAAUUAAAUAAUAAUU-AAA----------] AAAAU |GAUAAA|| AUUA-GUAA [-------AUAGUUAUUUAAAAAUUUAUAAAAUUAAAAGAUUAAAUUA] ||AAG|**

**Mengenilla chobauti [AAAUUAUAAAUAUGAUUAAUUAUAAAUAAU----] ||UUGUUAGA| [UUAAUAUUG----] |UUUAUCAA|| [----UAAUAGUUUAUUAAAAAUAUUUAUUUAAUAUUAAAGAUUUAUAAAUAUUUUAAUUUAUUUA--] |UAAAAU|| [UAAUAAAUUUAAUUUAAUAAUAAAU-AUA----------] AUAAU |GAUAAA|| AUUA-GUAA [-------AUAAAAAUUUAUAGAUAAUAUAAAAUAAAAUAUUGAGUUA] ||AAG|**

**Triozocera sp.(2) [AAAUUAUAAAUUAAAAUAAAUAUGUAAAA-----] ||UUGUAAAA| [UUAAUAUU-----] |UUUAUCAA|| [----UUAUAGGUUAUUAUAAAUAUUUUACAAAAUAUAAAAAUUUAUUUAAUUUUAAUUUUAAAA---] |UAAAAU|| [UAUUUUUUUUAAUAAUAUAUUAGAAAGAA----------] AUAAU |GAUAAA|| AUUA-GUAU [------AUUAUAUAAUUAAUUAAUUAAUAUUUAUAAAAAAUUAUUUU] ||AAG|**

**Triozocera sp.(4) [GAGUUAUAAAUAUAAAUAAAGAUUUAAAA-----] ||UUGUAGAG| [UUAAUAUU-----] |UUUAUCAU|| [----UAGUAAAUUAUUAUAAAUAUUUUAAUAAUUAUAAAAAUUUAUUUUGUUUUAAUUUUGAGA---] |UAAAAU|| [UAUUUUUAUAAAUAAUAU--UAAAAAGAA----------] AUAAU |GAUAAA|| AUUA-GUAU [--------AAUAUGAUUAAUUGAUUUAAAUUAAUAAAAAAUUAUUAA] ||AAG|**

**Triozocera sp.(3) [GAGUUAUAAAUGUAAAUAAAGAUUUAAAA-----] ||UUGUAAGA| [UUAAUAUU-----] |UUUAUCAU|| [----UAAUAAAUUAUUAUAAAUAUUUUAAUGAUUAUAGAAAUUUAUUUUGUUUUAAUUUUGAAA---] |UAAAAU|| [UAUUUUUAUUAAUAAUAU--UAAAAAGAA----------] AUAAU |GAUAAA|| AUUA-GUAU [------AUAUUAUAGUUAGUAAAUUUAAAUUAAUAAAAAAUUAUUAA] ||AAG|**

**Corioxenos acucyrt’ [?????????????????????????????-----] ||????????| [?????????----] |????????|| [???????????????????????????????????????????????????????????????????] |??????|| [???????????????????????????????????????] ????? |??????|| ????????? [------?????????????????????????????????????????] ||???|**

**Myrmecolax sp.(4) [AAUUUAAAUAAUUAAUAAAUUAAUAAAAAA----] ||UUGUAGGA| [UUAUAAUUU----] |UUCUUCAU|| [------UUAAUUUGUAAUAAAAUAUUUUUUAUUGUUGAAGAUUUAUUAAUUUAUUAAAUAUUUCA--] |UAAAUA|| [UAUAUUAUUAAAUUUUAUAAU-AAUAAAA----------] AUUAU |GAUAAA|| AUAA-GUAA [------AUUUUAAUUUAUAAAUAUUAUAAUUUUUAAAUUUAAUUUAA] ||AUG|**

**Myrmecolax sp.(1) [AAUUUAUAUAAUAGAUAAAUUUUUAUAAAAA---] ||UUGUAGGA| [UUAUAAUUU----] |UUCUUUAU|| [------UUAAUUUGUAAUAAAAUAUUUUUUUAUAGAUAAAGAUUUAUUAAUUUAUUAAAUAUUUUA-] |UAAAUA|| [UAUAUUAUUAAAUAAUUUAAU-AAUAGAA----------] AUAAU |GAUUAA|| AUAA-GUAA [-------AUUUUAAUUUAUAUGUGUGAUUAAUAAAAAUUUAAUUUAA] ||AUG|**

**Myrmecolax sp.(2) [AAUUUAAAUAAUUAAUAAAUUUAUAAAAAA----] ||AUGUAGGA| [UUAUAAUUU----] |UUCUUCAU|| [------UAAAUUUGUAAUAAAAUAUUUUUUAUUUUUAAAGAUUUAUUAAUUUAUUAAAUUUUUCA--] |UAAAUA|| [UAUAUUAUUAAAUUUUAUAAU-AAUAAAA----------] AUAAU |GAUAAA|| AUAA-GUAA [-------AUUUUAAUUUAUAAAUAUAAUAAUUUUAAAUUUAAUUUAA] ||AUG|**

**Myrmecholax incautus [AAUUUAUAUAUUUAAUAAAUAAAAAAAAAA----] ||AUAUAGGA| [UUAAUAUUG----] |UCUUUUAU|| [-----UAAAUUUUGUUAUAAAAUAUUUUAUUUUUAUAAAGAUUUAUUAAUAUAUUAAAUAUUUUA--] |UAAAUA|| [UUUAUUAUUAAAUUAAAUAAU-AAUAGUA----------] AUAAU |GAUAAA|| AUAA-GUAA [----AUUUUUUAAUUUAUAUAUAAUUUAUUAUAAAAAUUUAAUUUAA] ||AUG|**

**Myrmecholax sp.(5) [AAUUUAAAUAGUUAAUAAAUAAUAAUAAAA----] ||AUGUAGGA| [UUAUAAUUU----] |UUCUUCAU|| [------UUAAUUUGUAAUAAAAUAUUUUUUAUUUUUAAAGAUUUAUUAAUUUAUUAAAUAUUUUA--] |UAAAUA|| [UAUAUUAUUAAAUUUUAUAAU-AAUAAAA----------] AUUAU |GAUAAA|| AUAA-GUAA [------AUUUUAAUUUAUAAAUAUUAUAAUUAUUAAAUUUAAUUUAA] ||AUG|**

**Caenocholax sp.(1) [AAAUUAUAUGAUUAAAAAAUUUUUAAAAUU----] ||UUAUAGGA| [UUAAUAUUU----] |UUUAUUAU|| [---AUUUAAAUUUUUAUAAAUAGAUUUUAUUAUAAUAAAAAUUUAUUUUUUCAUUAAUUUUUUUA--] |UAAAAA|| [UAAAUUAUUUAAUUAA-UAAUUAAUUGUA----------] AUAAU |GAUAAA|| AUUA-GUAA [-------AUUAUUUUUUAAUAAAUUAUUAGAUUGAUAAAUUAAAUUA] ||AUG|**

**Caenocholax sp.(3) [AAUUUAUAUAUUAAAAUAAUUUUUAAAAUU----] ||UUAUAGAA| [UUAAGAUUU----] |UUUAUUAG|| [---UUUUAAAUUUUUAUAAAUAAAUUUUAUUUAAUAAAAAUUUAUUAUUAUAUUAAAUUUUUUA---] |UAAAGA|| [CAUAUUAUUUAAUAAAUU-AAUAGUAAUA----------] AUAAU |GAUAAA|| AUUA-GUAA [-------UUUUUUAUUAAUAUUUAAAAUUAAAUUAAAUUUUAAUUUA] ||AUG|**

**Lychnocolax sp.(2) [AAUUUAUAUGUUUAAUAAAUAAGAUUGU------] ||AAGUAGAA| [UUAAAAUUA----] |UUUAUCAA|| [-----AAAAUUUUUUAUAAAUUAAUUUUUAAAAAAUAAAAAUUUAUUAAUAUUAUUAAAUAUUUUA-] |UAAAAU|| [UAAAAUAAUAAAUUUUAA-GUUAUUUAAA----------] AAAAU |GAUAAA|| AUUA-GUAA [------AUUAUUAAUUUAUUAAUAAAUUAAAAAAAAAAAUUAAAUUU] ||AUG|**

**Xenos hamiltoni [UUAUUAAAUUAUAAAAAAAAAUAAAUUAAAAU--] ||AAAUAGGA| [AUAAUAUUU----] |UUCAUUUU|| [--UAUUUAAUUUAUUAUAAAAAUAAUUUUUAUUAAUAUAAAUUUUUUUUAUAUAAUUAAAUUUUUUA] |AAAAAA|| [UUUAUUUUAUAAUAAGUU-AAAAAUAUUU----------] AUAAU |GAUUAA|| AUUA-GUAA [-----AUUUUAAAUUUAUUAAUAAAAUUAAUAAAAAAAAUUAUUUUU] ||AUG|**

**Xenos moutoni [AGGUUAUAUAAAAAAAAAAUUAUUUAAAUU----] ||AAAUAGGA| [UUAAUAUUU----] |UUCAUUUA|| [----UAUUAUUUUUUAUUAAAUAAUUUAUAAUAUAUAAAAAUUUUUUUUUUUAUUAAUUUAUCUA--] |UAAAAA|| [UAUUUUUAAUAAUUAAAU-AUAAAAAGAA----------] AAAUU |GAUAAA|| AUUA-GUAA [------AUUAGAAAUUUAAAAAUAAGGUUAUAAAGAAAAUUAAAUUU] ||AUG|**

**Xenos vesparum [AUAUUAAAUUUAUUAAAAAAUUGAAUUAAU----] ||UUAUAGGA| [AUAGAAUUU----] |UUCAUUAU|| [-----UUAAUUUUUUAUAAAUAAUUUAUUAUAGAAAAAAGAUUUUUUAGUUUUUUAAGUUUUUUA--] |UAAAAA|| [UAUUAUUUUUAAUUAUUU-AGAAUAAGUG----------] AAAGU |GAUAAA|| AUUA-GUAA [-------AUUUUAUUUAUUAAGUAGAUGUGAAUAAAAAAUUAUUUAU] ||AUG|**

**Xenos pecki [UUAUUAUAUUAUAAAAUAAAUGAAUUAAAAU---] ||AAAUAGGA| [AUAAUAUUU----] |UUCAUUUU|| [--AAUUUAAUUUAUUAUAAAAAUAAUUUUUAUUGAUAUAGAUUUUUUUUAUAAUUAAGUUUUUUA--] |AAAAAA|| [UUUAUUUUCUAAUAAAUUAUAAAUAUUA-----------] AUAAU |GAUUAA|| AUUA-GUAA [------AUUUAAAAUUUAUUUAUAAAAUUAAUAAAAAAAUUAUAUUU] ||AUG|**

**Xenos sp. [UAAUUAAAUUUAAUAAAAAAUGUUAAAAAU----] ||AUAUAGGA| [AUAAAAUUU----] |UUCAUUAU|| [----UAUAGUUUUUUAUAAAUUAUUUAUAUAUAAUUAAAAAUUUUUUAAUUUUUUAAAUUAAUUA--] |UAAAAA|| [UAUUAGAUUAAAUAAAAUUUUUUAAGAG-----------] AUAAU |GAUAAA|| AUUA-GUAA [-----AUAUUUUUUAAUUUAAUUAAUAACAUAAAAUAAAUUAUUUAU] ||AUG|**

**Paraxenos sp. [UGGUUAAAUUUUUAAAAAAUUAAUUUAAGU----] ||AAGUAGGA| [UUAAAAUUG----] |GCAUUUUU|| [-----UAUAGUUUAUUAUAAAUAAAUUAAUAUUAUUAAAGAUUUUUUUUUAAUUAGCUUUGCAUA--] |UAAAAA|| [UAUAUUUUAAAAUAAAAUUUAAGUAGAA-----------] AAAAU |GAUAAA|| AUUA-GUAA [------AUUAAGAUAAAAUUAUAUAAAUAAUUUUAAAAAUUAUAUUU] ||AUG|**

**Pseudoxenos sp. [AGAUUAUAUUUUUAAAUAAUAAAAGAAAAU----] ||AAAUAGGA| [UUAAUAUUG----] |CCAUUUUU|| [----AAUAAUUAUUUAUAAAAAAUUUAUAAGUUUAUGAAGAUUUUUUAAGGAUUAAAUUGACUA---] |UAAAAA|| [UAUAUUUUUUAAUUUAAUUAAAAGAGUG-----------] AUAAU |GAUAAA|| AUUA-GUAA [--------AAUUUAUUAAUUUAUAGGAAUAAAAUAGAAUUUAUUUUU] ||AUG|**

**Stylops mellitae [AAAUUAUAUUUAAAUAAUAAUUUUUUUUAAU---] ||AUAUAAGA| [UUAAAAAUUU---] |ACUAAUAU|| [-------UAUUUUUUUAUAAAUAAUUAAUUAUUUAUAUAAAUUUAUUAAUUAAAUUAAUUUUUUUA-] |UAAAAA|| [UAUUUAUAUUAAUAAAUUUUAUAAAGUA-----------] AUAAU |GAUAAA|| AUUA-GUAA [-------AUAUUAUUUAAUUAAUAAAAUAAAUUUAUUAAUUAAAUUA] ||AUG|**

**Tridactylophagus sp. [UUUUUAUAUAAUAAUUAUAAAAUAAAAAAA----] ||AAGUAAGA| [UUUUUAUUUUUUA] |UCUAAUAA|| [-------AAUUUUAUUAAAAAUAAUUUUUUUAUAUAUUAAAUUUAUUAUUAUUAUUGAUUUUUAUA-] |UAAAAA|| [AAUUAUUAUUAAUUUAAUUAAUUAAUUU-----------] AAAAU |GAUUAA|| AUUA-GUAA [------AUAAAAUUUUUAUAAAAUAAAUUUAUUUAAAUUUAAUUAAA] ||AUG|**

**Halictophagus calcaratus [-AAAUAUAAUUUUGAUUUAUAAAUUU--------] ||UAAUAAAC| [UUUUAAAUU----] |UUGUAUUA|| [---------AGUUUUUAAUAAAUUCAAUUUUGUUAUUUUUAUUUAUUUUAAUUUAUUUAAUUA----] |UAAAGU|| [UUAUUUAAUUAAUAAUUUUUAUUUUAA------------] AUAAU |GAUUUA|| AUUA-GUAU [----------------------------AAUAUUUAUUUAAUUAUAU] ||UUG|**

**Halictophagus sp.(3) [UUUUUAUAUAAUUAAUUUAAUUUUAUAAA-----] ||UAAUAGAA| [UUAAUAUUU----] |UUCAUUUA|| [---UUAUUAUAUUUUUAUUAAAAAUUAUUAAAUUUAAUAAAUUUUAAAUUAAUUAUUAAUUAAUUUA] |UUAAAA|| [AAUUAAUUUUAAUUUAUUAAAUUAAGAU-----------] AAAAU |GAUUAA|| AUUA-GUAU [------AAAAAGAUUUAUUUAAUAGAAAUUAAUUAAAUUUAUUUAAA] ||AUG|**

**Halictophagus silwoodensis [UUUUAAAAUUUAAAUUUUCUUUGUCUU-------] ||UUAUAUCU| [UUAAAAUUU----] |UGUAUUUA|| [------------GUUUUUAAUAAAUUAUUUUUUUAUAAUAUUAUUUUAUUAUAUUUAUAAUUUCA--] |CUUAAU|| [UUUAAAAUUUAAUUUAAUUUUUUUUUA------------] AUAAU |GAUUAA|| AUUA-GUAA [---------------------------AAUUUUUAUAUAAUUUUUAA] ||AU-|**

**Halictophagus sp.(2) [AUUUUAAAUAAUUUUUUUUAAAUUUUUAAA----] ||UAAUAGAA| [UUAAUAUUG----] |UUCAUUUA|| [--UUUAUAUUUUUUUAUUAAAAAUUAAUUUUUAUUAAAAAAUUUUAAAAUAAUUAUUAGAUUUUUUA] |UUAAAA|| [AAUUAUUUUAAAUUGAUUAAAUUAAGUU-----------] AAAAU |GAUUAA|| AUUA-GUAU [-------AAAAAAUUUUAUAAAUUAAUUUAUUAAAAAUUUAAUUAAA] ||AUG|**

**Elenchus koebelei [-UGAUAUGAUUAAUAAUAAUUUAUUUUAAU----] ||UAGUAAGG| [UUAUUAUUUCCUA] |CCU-UUUA|| [-------UUUUAUUGUAAAUAAUUAAAAUUUUAUAUAUAAAUUUUUUAAAUUAUCUAUUUUUUCUA-] |UAAAAU|| [UAAUUAUUAUAAUUUAUUAAAUAAUAAA-----------] AUAAU |GAUAAA|| AUUA-GUAA [-------AUUAAUAUUUGUUUUUAAUUUUAUUUUAUUAAUAAAUUAA] ||AUG|**

**Elenchus sp.(1)**  **[-UAAUAUGAUUAAUAAUAAUUUAUUUUAAU----] ||UAGUAAGG| [UUAUUAUUUCCUA] |UCU-UUUA|| [-----UUUUAUUGUAAAUAAUUAAAAUUUUUUAUAUAUAAAUUUUUUAAAUUUUCUAUUUUUUAUA-] |UAAAAU|| [UAAUUAUUAUAAUUUAUUAAGUAAUAAA-----------] AUAAU |GAUAAA|| AUUA-GUAA [-------AUUAAAUUUUAUUUUUAAUUUUAUUUUAACAAUAAAUUAA] ||AUG|**

**RAA12 H1764a H1764b H1775 H1775’ H1792 RAA13 H1792’ H1830 H1835a H1835b H1835c RAA14 H1835c’ H1835b’ H1835a’ H1906 H1906’ H1925 H1925’ H1935 H1935’ H1830’ H1764b' H1764a'**

**.......... ((( (((((( .. ((( ......... ))) .. .((((((( ......... ))))))). (((( . ((( .. (( ((( ))) .. )) ))) (((((( ...... )))))) ( ... ) ............... (((((( ..... )))))) .......... )))) ... )).)))) )))**

**Periplaneta fulig' AAUUAGGCAA [AAUUAAUA----] ||---| |UUCUCG| CA ||UGU| UUAUCAAAA |ACA|| UC ||UCUUCUUG| [UAUAAA---] |UAUAAAGU|| [AU] ||GACC| U ||GCC| CA |CU| |GA-| [UUAAUU-] |-UU| GA |AG| |GGC|| ||CGCGGU| AUUUUG |ACCGUG|| ||C| AAA |G|| GUAGCAUAAUCAUUA ||GUCCUU| UAAUU |GUGGAC|| UGGAAUGAAU |GGUU|| GGA |CGAGGAA| |---||**

**Tamolanica tamolana AAUUAGGCAA [AACUUU------] ||---| |UGCUCA| CC ||UGU| UUAUUAAAA |ACA|| UG ||GUUUCUUG| [UAUUUUAUU] |UAAGAAAU|| [UU] ||GACC| U ||GCC| CG |CU| |GAA| [UAAAAU-] |UUU| GA |AG| |GGC|| ||CGCAGU| AUAUUG |ACUGUG|| ||C| AAA |G|| GUAGCAUAAUCAUUA ||GUCUUU| UAAUU |GAAGGC|| UGGUAUGAAU |GGUU|| GGA |UGAGGUA| |---||**

**Reticulitermes fla’ AACUAGGCAA [AUUUUA------] ||---| |UGUCCG| CC ||UGU| UUAACAAAA |ACA|| UC ||UCUUCUCG| [UUAGUU---] |UUUGAAGU|| [AU] ||GGCC| U ||GCC| CA |CU| |GAC| [CUUGAAU] |GUU| GA |AG| |GGC|| ||CGCGGU| AUUUUG |ACCGUG|| ||C| AAA |G|| GUAGCAUAGUCAUUA ||GUUCUU| UAAUU |GUGAUC|| UGGUAUGAAU |GGCU|| UGA |CGAGGCA| |---||**

**Antheraea pernyi AAUUCGGCAA [AAAUUUA-----] ||---| |UAUUCA| CU ||UGU| UUAUCAAAA |ACA|| UG ||UCUUUUUG| [GAAAUAA--] |UUUAAAGU|| [CU] ||AAUC| U ||GCC| CA |CU| |GAU| [GAAAU--] |AUU| GA |AG| |GGC|| ||UGCAGU| AUUUUG |ACUGUA|| ||C| AAA |G|| GUAGCAUAAUCAUUA ||GUCUCU| UAAUU |AGGGAC|| UUGUAUGAAG |GAUU|| UGA |UGAAAUA| |---||**

**Bombyx mori AAUUCGGCAA [AUAUUUAAUUC-] ||---| |------| CU ||UGU| UUAUCAAAA |ACA|| UG ||UCUUUUUG| [UUAAUAA--] |UUUAAAGU|| [CU] ||AAUC| U ||GCC| CA |CU| |GAU| [AUAUUU-] |AUU| AA |AG| |GGC|| ||UGCAGU| AUUUUG |ACUGUA|| ||C| AAA |G|| GUAGCAUAAUCAUUA ||GUCUUU| UAAUU |GGUGAC|| UUGUAUGAAA |GAUU|| GGA |UGAAAUA| |---||**

**Haematobia irritans AACUCGGCAA [AAUAA ------] ||---| |UGUUCG| CC ||UGU| UUAACAAAA |ACA|| UG ||UCUUUUUG| [AAUUAUA--] |UUUAAAGU|| [CU] ||AACC| U ||GCC| CA |CU| |GAA| [AUU----] |UUU| AA |AU| |GGC|| ||CGCAGU| AUCCUA |ACUGUG|| ||C| AAA |G|| GUAGCAUAAUCAUUA ||GUCUUU| UAAUU |GAAGGC|| UGGUAUGAAU |GGUU|| GGA |CGAGAUA| |---||**

**Ceratitis capitata AACUCGGCAA [AUAUAU------] ||---| |UACUCG| CC ||UGU| UUAACAAAA |ACA|| UG ||UCUUUUUG| [AGUUAUU--] |UUUAAAGU|| [CU] ||GACC| U ||GCC| CA |CU| |GAA| [UAAUU--] |UUU| AA |AU| |GGC|| ||CGCAGU| AUCCUA |ACUGUG|| ||C| AAA |G|| GUAGCAUAAUCAUUA ||GUCUUU| UAAUU |GAAGGC|| UGGUAUGAAC |GGUU|| GGA |CGAAGUA| |---||**

**Tribolium castaneum AACUCGGCAA [-UUGAA------] ||---| |UUUUCA| CC ||UGU| UUAUUAAAA |ACA|| UG ||GCUUUUUG| [AUUAUAA--] |UUUAAGGU|| [CG] ||GACC| U ||GCC| CA |AU| |GAG| [GAU----] |UUU| -A |AU| |GGC|| ||CGCAGU| AUUUUG |ACUGUG|| ||C| AAA |G|| GUAGCAUAAUCAUUA ||GUUUCU| UAAUU |AGAAGC|| UGGAAUGAAU |GGUU|| UAA |UGAGAAA| |---||**

**Rhagophthalmus ohb' AAUUCGGCAA [AUUUUA------] ||---| |UUUCCG| CC ||UGU| UUAUUAAAA |ACA|| UG ||UCUUUUUG| [AUAAUAA--] |UUUAAAGU|| [UU] ||AAUC| U ||GCU| CA |AU| |GA-| [UUAU---] |-UU| AA |AU| |UGC|| ||CGCAGU| AUUUUG |ACUGUG|| ||C| AAA |G|| GUAGCAUAAUAAUUA ||GUUUUU| UUAUU |GAAAAC|| UUGCAUGAAU |GAUU|| GGA |CGAGAAA| |---||**

**Mengenilla australiensis AAUUUAACAA [AUA---------] ||UAU| |UUUUUA| CC ||UGU| UUAUUAAAA |ACA|| UG ||UCUAUUUG| [AAAAGAA--] |UAAAUAGU|| [UU] ||AAUC| U ||GCC| CA |UU| |GA-| [AAAA---] |-UU| AA |AA| |GGC|| ||UGCAGU| AUAUUG |ACUGUA|| ||C| AAA |G|| GUAGCAUAAUCAUUA ||GUUUUU| UAAUU |GAAAAC|| UGGAAUGAAG |GAUU|| GAA |UA-AAAA| |AUA||**

**Mengenilla chobauti AAUUAGACAA [AUA---------] ||AAU| |UUUUUA| CC ||UGU| UUAUUAAAA |ACA|| UG ||UCUAUUUG| [AAAAGAA--] |UAAAUAGU|| [CU] ||AAUC| U ||GCC| CA |UU| |GA-| [UUAA---] |-UU| AA |AA| |GGC|| ||UGCAGU| AUAUUG |ACUGUA|| ||C| AAA |G|| GUAGCAUAAUCAUUA ||GUUUUU| UAAUU |AGGAAC|| UUGAAUGAAU |GAUU|| GAA |UA-AAAA| |AUG||**

**Triozocera sp.(2) AAUUCGACAA [AA----------] ||UUU| |UAUUCA| UC ||UGU| UUAUUAAAA |ACA|| UA ||UUUAAUUG| [AAUUGAA--] |UAAUUAAU|| [GU] ||AUUC| U ||GCC| CA |UU| |GA-| [UAUA---] |-UU| AA |AA| |GGC|| ||UGCAGU| AUUUUG |ACUGUA|| ||C| AAA |G|| GUAGCAUAAUCAUUU ||GUUUUU| UAAUU |GAAAAC|| UUGAAUGAAG |GGAU|| UAA |UG-AAAU| |AAG||**

**Triozocera sp.(4) AACUAGACAA [AA----------] ||UUU| |UAUCCA| UC ||UGU| UUAUUAAAA |ACA|| UA ||UUUAAUUG| [AAUUGAA--] |UAAUUAAU|| [GU] ||AUUC| U ||GCC| CA |UU| |GA-| [UAAAU--] |-UU| AA |AA| |GGC|| ||UGCAGU| AUUUUG |ACUGUA|| ||C| AAA |G|| GUAGCAUAAUCAUUU ||GUUUUU| UUAUU |GGAAAC|| UUGUAUGAAA |GAAU|| UAA |UG-AGAU| |AUU||**

**Triozocera sp.(3) AACUAGACAA [AA----------] ||UUU| |UAUCCA| UC ||UGU| UUAUUAAAA |ACA|| UA ||UUUAAUUG| [AAUUGAA--] |UAAUUAAU|| [GU] ||AUUC| U ||GCC| CA |UU| |GA-| [UAAA---] |-UU| UA |AA| |GGC|| ||UGCAGU| AUUUUG |ACUGUA|| ||C| AAA |G|| GUAGCAUAAUCCUUU ||GUUUUU| UUAUU |GGAAAC|| UUGUAUGAAG |GAAU|| UAA |UG-AGAU| |AUC||**

**Corioxenos acucyrt’ ?????????? [??----------] ||???| |??????| ?? ||???| ????????? |???|| ?? ||????????| [???????--] |????????|| [??] ||????| ? ||???| ?? |??| |???| [???????] |???| ?? |??| |???|| ||??????| ??AUUG |ACUGUA|| ||C| UAA |G|| GUAGCAUAAUAAUUU ||GUUUUC| UAAUA |GGGAAC|| UUGUAUGAAA |GAUU|| UAU |UG-AAAA| |AAA||**

**Myrmecolax sp.(4) AAUUAGGCAA [AA----------] ||UAA| |UUUUUA| CC ||UGU| UUAUUAAAA |ACA|| UG ||UUUAUUUG| [AAUUAAU--] |UAAAUAAU|| [UU] ||UAUC| U ||GCC| CA |UU| |GA-| [UAAU---] |-UU| UA |AA| |GGC|| ||UAUAGU| AUUUUA |ACUAUA|| ||C| AAA |G|| GUAGCAUAAUAAAUA ||GUUUUU| UAAUU |GAAAAC|| UAGAAUGAAU |GAUU|| UGA |UA-AAAA| |UUA||**

**Myrmecolax sp.(1) AAUUAGGCAA [AA----------] ||UAA| |UUUUUA| CC ||UGU| UUAUUAAAA |ACA|| UG ||UUUAUUUG| [AAUUAAA--] |UAAAUAAU|| [UG] ||UAUC| U ||GCC| CA |UU| |GA-| [UUAU---] |-UU| UA |AA| |GGC|| ||UAUAGU| AUUUUA |ACUAUA|| ||C| AAA |G|| GUAGCAUAAUAAAUA ||GUUUUU| UAAUU |GAAAAC|| UAGAAUGAAU |GAUU|| UGA |UA-AAAA| |UUA||**

**Myrmecolax sp.(2) AAUUAGGCAA [AA----------] ||UAA| |UUUUUA| CC ||UGU| UUAUUAAAA |ACA|| UG ||UUUAUUUG| [AAUUAAA--] |UAAAUAAU|| [UU] ||UAUC| U ||GCC| CA |UU| |GA-| [UUAU---] |-UU| AA |AA| |GGC|| ||UAUAGU| AUUUUA |ACUAUA|| ||C| AAA |G|| GUAGCAUAAUAAAUA ||GUUUUU| UAAUU |GAAAAC|| UAGAAUGAAU |GAUU|| UGA |UA-AAAA| |UUA||**

**Myrmecholax incautus AAUUAAGCAA [AA----------] ||UAA| |UUUUUA| CC ||UGU| UUAUUAAAA |ACA|| UG ||UCUAUUUG| [AAAAGAA--] |UAAAUAGU|| [CA] ||UAUC| U ||GCC| CA |UU| |GA-| [UUGUU--] |-UU| AA |AA| |GGC|| ||UAUAGU| AUUUUA |ACUGUA|| ||C| AAA |G|| GUAGCAUAAUAAAUA ||GUUUUU| UAAUU |GAAAGC|| UAGAAUGAAU |GAUU|| UGA |UA-AAAA| |UUA||**

**Myrmecholax sp.(5) AAUUAGGCAA [AA----------] ||UAA| |UUUUUA| CC ||UGU| UUAUUAAAA |ACA|| UG ||UUUAUUUG| [AAUUAAU--] |UAAAUAAU|| [UU] ||UAUC| U ||GCC| CA |UU| |GA-| [UUAU---] |-UU| UA |AA| |GGC|| ||UAUAGU| AUUUUA |ACUAUA|| ||C| AAA |G|| GUAGCAUAAUAAAUA ||GUUUUU| UAAUU |GAAAAC|| UAGAAUGAAU |GAUU|| UGA |UA-AAAA| |UUA||**

**Caenocholax sp.(1) AAUUAGGCAA [AA----------] ||UAA| |UUUUUA| CC ||UGU| UUAUUAAAA |ACA|| UG ||UCUGUUAG| [AAAUAAA--] |UUAAUAGU|| [CG] ||AAUC| U ||GCC| CG |UU| |GA-| [UUAA---] |-UU| AA |AU| |GGC|| ||UAUAGU| AUUUUA |ACUAUA|| ||C| AAA |G|| GUAGCAUAAUAAAUU ||GUUUUU| UAAUU |GAAAAC|| UAGAAUGAAU |GAUU|| UGA |UG-AAAG| |UUA||**

**Caenocholax sp.(3) AAUUAGACAA [UA----------] ||UAA| |UUUUUA| CC ||UGU| UUAUUAAAA |ACA|| UG ||UCUGUUAG| [AAUUAUA--] |UUAAUAGU|| [CU] ||AAUC| U ||GCC| CG |UU| |GA-| [UUAA---] |-UU| AA |AU| |GGC|| ||UAUAGU| AUUUUA |ACUAUA|| ||C| AAA |G|| GUAGCAUAAUAAAUU ||GUUCUU| UAAUU |GAGAAC|| UAGAAUGAAU |GAUU|| UGA |UA-AAAA| |UUA||**

**Lychnocolax sp.(2) AAUUAGACAG [UA----------] ||UUA| |UUUUUA| CC ||UGU| UUAUUAAAA |ACA|| UG ||UUUAUUUG| [UAUUAAA--] |UAAAUAAU|| [UU] ||AAUC| U ||GCC| CA |UU| |GA-| [AUAA---] |-UU| AA |AA| |GGC|| ||UGCAGU| AUAUUA |ACUGUA|| ||C| AAA |G|| GUAGCGUAAUCAUUA ||GUUUUU| UAAUU |GAAAAC|| UUGUAUGAAA |GAUU|| GGA |UA-AAAA| |UAA||**

**Xenos hamiltoni AAUUCGGCAA [AA----------] ||UUA| |UUUUCA| CC ||UGU| UUAAUAAAA |ACA|| UG ||GUUAAUUG| [AAUUGAA--] |UAAUUAAU|| [UA] ||AACC| U ||GCC| CA |UU| |GA-| [GUAAAA-] |-UU| UA |AA| |GGC|| ||UGCAGU| AUAUUG |ACUGUA|| ||C| AAA |G|| GUAGCAUAAUCAAUA ||GUUUUU| UAAUU |GAAAAC|| UUGUAUGAAA |GGUU|| UAA |UGAAAAA| |UAA||**

**Xenos moutoni AAUUAGGCAA [AU----------] ||ACA| |UUUUCA| CC ||UGU| UUAGUAAAA |ACA|| UG ||GUUAUUUG| [CAUAAGA--] |UAAAUAAU|| [UG] ||GACC| U ||GCC| CA |UU| |GAU| [AUAA---] |AUU| AA |AA| |GGC|| ||UGCAGU| AUUUUA |ACUGUA|| ||C| AAA |G|| GUAGCAUAAUCAUUA ||GCUUUU| UAAUU |GAAAGC|| UUGUAUGAAU |GGUU|| UGA |UGAAAAA| |UGU||**

**Xenos vesparum AAUUAGACAA [AA----------] ||UUA| |UUUUUA| CC ||UGU| UUAAUAAAA |ACA|| UG ||GUUGUUAG| [UAUAGAG--] |UUAAUGAU|| [CA] ||GGCC| U ||GCC| CA |UU| |GA-| [AUU----] |-UU| UA |AA| |GGC|| ||UGCAGU| AUAUUG |ACUGUA|| ||C| AAA |G|| GUAGCGUAAUCAUUA ||GUUUUU| UAAUU |GAAAAC|| UUGUAUGAAU |GGCU|| UGA |UAAAAAA| |UAA||**

**Xenos pecki AAUUCGGCAA [AA----------] ||UUA| |UUUUCA| CC ||UGU| UUAAUAAAA |ACA|| UG ||GUUAAUUG| [AAUUGAA--] |UAAUUAAU|| [CA] ||AACC| U ||GCC| CA |UU| |GA-| [GUAAAA-] |-UU| UA |AA| |GGC|| ||UGCAGU| AUAUUA |ACUGUA|| ||C| AAA |G|| GUAGCAUAAUCAAUA ||GUUUUU| UAAUU |GAAAAC|| UUGUAUGAAA |GGUU|| UAA |UGAAAAA| |UAA||**

**Xenos sp. AAUUAGGCAA [AA----------] ||UUA| |UUUUCA| CC ||UGU| UUAAUAAAA |ACA|| UG ||GUUGUUUG| [UAUUUUA--] |UAAAUGAU|| [UA] ||GACC| U ||GCC| CA |UU| |GA-| [UUU----] |-UU| UA |AA| |GGC|| ||UGCAGU| AUUUUUA|ACUGUA|| ||C| AAA |G|| GUAGCAUAAUCAUUA ||GUUUUU| UAAUU |GAAAAC|| UUGUAUGAAU |GGUU|| UGA |UGAAAAA| |UAA||**

**Paraxenos sp. AACUAGGCAA [AU----------] ||UUA| |UUUUCA| CC ||UGU| UUAUUAAAA |ACA|| UG ||GUUGUUUG| [AAGUGAA--] |UAAAUGAU|| [CA] ||GGUC| U ||GCC| CA |UU| |GA-| [GAU----] |-UU| AA |AA| |GGC|| ||UGCAGU| AUAUUG |ACUGUA|| ||C| AAA |G|| GUAGCAUAAUCAUUA ||GUUUUU| UAAUU |GAAAAC|| UUGUAUGAAU |GAUU|| UAA |UGAGAAA| |UAA||**

**Pseudoxenos sp. AAUUAGGCAA [AA----------] ||UUA| |UUUUCA| CC ||UGU| UUAGUAAAA |ACA|| UG ||GUUGUUUG| [AAUUAAA--] |UAAAUAAU|| [UG] ||AAUC| U ||GCC| CA |UU| |GA-| [AGU----] |-UU| AA |AA| |GGC|| ||UGCAGU| AUACUG |ACUGUA|| ||C| AAA |G|| GUAGCAUAAUCAUUA ||GUUUUU| UAAUU |GAAAAC|| UUGUAUGAAG |GAUU|| UGA |UGAAAGG| |UAA||**

**Stylops mellitae AAUUAGGCAA [AA----------] ||UUA| |UUUCUA| CC ||UGU| UUAUUAAAA |ACA|| UG ||UCUGUUUG| [AAUAGAA--] |UAAAUAGU|| [CU] ||AAUC| U ||GCC| CA |UU| |GA-| [UUAA---] |-UU| AA |AA| |GGC|| ||UGCAGU| AUUUUA |ACUGUA|| ||C| AAA |G|| GUAGCAUAAUAAUUA ||GUUUCU| UAAUU |AGAGAC|| UUGAAUGAAA |GAUU|| UGA |UAAGAAA| |UAA||**

**Tridactylophagus sp. AAUUUGGCAA [AA----------] ||UUA| |UUUUUA| CC ||UGU| UUAAUAAAA |ACA|| UG ||UCUAUUUG| [AAAAGAU--] |UAAAUAGU|| [CG] ||AGUC| U ||GCC| CA |UU| |GA-| [UUAU---] |-UU| UA |AA| |GGC|| ||UGCAGU| AUUUUA |ACUGUA|| ||C| AAA |G|| GUAGCGUAAUCAUUA ||GUUUUU| UAAUU |GAAAAC|| UUGAA-GAAA |GAUU|| UAA |UA-AAAA| |UAA||**

**Halictophagus calcaratus AAUUCGACAA [UUA---------] ||UAA| |UUUCUA| AC ||UGU| UUACCAAAA |ACA|| UU ||UCUUUGAG| [UUAAAAU--] |UUCAAAGU|| [AA] ||GUUC| U ||GCC| CU |AU| |GA-| [AUAU---] |-UU| AA |AU| |GGC|| ||UGCAGU| AUUUUA |ACUGUA|| ||C| AAA |G|| GUAGCAUAAUAAUUA ||GUCUUU| UAAUU |AAGGGC|| UAGUAUGAAU |GAAU|| AAA |UAAGAAA| |UUU||**

**Halictophagus sp.(3) AAUUCGGCAA [AA----------] ||UAA| |UUUUUA| CC ||UGU| UUAUCAAAA |ACA|| UG ||UCUGUUUG| [UAAAAGAA-] |UAAAUAGU|| [CA] ||AAUC| U ||GCC| CA |UU| |GA-| [AAU-- -] |-UU| UA |AA| |GGC|| ||UGCAGU| AAAUUG |ACUGUA|| ||C| AAA |G|| GUAGCAUAAUCAUUA ||GUUUUU| UAAUU |GAAAAC|| UUGUAUGAAU |GAUU|| UAA |UAAAAAA| |UUA|**

**Halictophagus silwoodensis AACUUGACAA [AUA---------] ||UUU| |AUUUCA] AC ||UGU| UUAUCAAAA |ACA|| UU ||UCUUUUAG| [AUUUAUU--] |UUAAAAGU|| [AA] ||AUUC| U ||GCC| CU |AU| |GA-| [UGAU---] |-UU| AA |AU| |GGC|| ||UGCAGU| AAUUUG |ACUGUA|| ||C| AAA |G|| GUAGCAUAAUAAUUA ||GUCUUU| UGAUU |GAAGAC|| UAGAAUGAAG |GAGU|| AAA |UG-AAAU| |UAA||**

**Halictophagus sp.(2) AAUUCGGCAA [UU----------] ||UAA| |UUUUUA| CC ||UGU| UUAUCAAAA |ACA|| UG ||UCUGUUUG| [UAAUGAA--] |UAAAUAGU|| [CA] ||AAUC| U ||GCC| CA |UU| |GA-| [AAU----] |-UU| UA |AA| |GGC|| ||UGCAGU| AUAUUA |ACUGUA|| ||C| AAA |G|| GUAGCAUAAUCAUUA ||GUUUUU| UAAUU |GAAAAC|| UUGUAUGAAG |GAUU|| UAA |UAAAAAA| |UUA||**

**Elenchus koebelei AACUAGGCAA [AA----------] ||GUA| |UUUUUA| CC ||UGU| UUAUUAAAA |ACA|| UG ||UCUAUUUG| [AAUAAAA--] |UAAAUAGU|| [CU] ||GAUC| U ||GCC| CA |UU| |GA-| [UUAAU--] |-UU| UA |AA| |GGC|| ||UGCAGA| AUUUUA |ACUGUA|| ||C| AAA |G|| GUAGCAUAAUAAUUU ||GAUUUU| UAAUU |GGAAUC|| UUGUAUGAAU |GAUA|| UGA |UAAAAAA| |UGC||**

**Elenchus sp.(1)**  **AACUAGGCAA [AA----------] ||GUA| |UUUUUA| CC ||UGU| UUAUUAAAA |ACA|| UG ||UCUAUUUG| [AAUAAAA--] |UAAAUAGU|| [CU] ||GAUC| U ||GCC| CA |UU| |GA-| [UUAAU--] |-UU| UA |AA| |GGC|| ||UGCAGA| AUUUUA |ACUGUA|| ||C| AAA |G|| GUAGCAUAAUAAUUU ||GAUUUU| UAAUU |GGAAUC|| UUGUAUGAAU |GAUA|| UGA |UAAAAAA| |UGC||**

**RAA15 H1648b’ RAA16 – H1648a’ H2023 H2023’ H2043 H2064a H2064b H2077 RAA17 H2077’ H2248 H2248’ RAA18 – H2269-H2347**

**.... ))) ............. ((((( ........ ))))) ..... ???????????? ...... ((((((( ((((( .. (((( )))) .. (((( ..... ))))**

**Periplaneta fulig' [UAU--] ACUG |UUU|| [CUUAUUAUUUUAU-------------] UUGAAUUUAAUUU ||UUAAG| UUAAAAAG |CUUAA|| AUUUA ||UUUAUGGGACGA| GAAGAC ||CCUAUAG| |AGUUU| AU ||ACAU| [--------AUUUUUAUAUUUAUUUUGUUUGAUUAUUUUUAUUAAAUAAUUGAU] |AUGU|| UU ||UGUU| GGGGU |GAUA|| [GGAAGAAUUAAUUAACUCUUUUUUGUUUUAUACAUUUAUUUAUGAUA----]**

**Tamolanica tamolana [AAU--] UCUG |UUU|| [UAUGUUAAUUUGUG------------] UUGAAUUUAGAUU ||UUAAG| UAAAAAGA |CUUAG|| AUGAU ||AUUAAGGGACGA| GAAGAC ||CCUAUAG| |AGUUU| A- ||AUAA| [----AUGUAUUAAUUAGUUGAGUUAUGAAUGUAAUUUUUUAUUAUUAUAAAAU] |UUAU|| UU ||AAUU| GGGGU |GAUU|| [GAAAGAUAAAUGUAACUCUUUUUUAUAUUUAAUAUUUUGAUUAGUU-----]**

**Reticulitermes fla’ [UAG--] GCUG |UCU|| [UAAUUUUGAAUUGUUUA---------] UUGAAUUUGGUCU ||UUGAG| UUAAAAUU |CUUAG|| AUGUU ||UUUAUGGGACGA| GAAGAC ||CCUAUAG| |AGUUU| G- ||ACAU| [----UUAUUCACGGUCUCUUUCUGUUUGUGAGGGUUCACUAGGGCUGUUUAAU] |AUGU|| UU ||UGUU| GGGGU |GAUG|| [GGAGGGAUAUUAUUUAACCCCUCCUUUGUGUUGUUAUAUUUAUUUAUAUUU]**

**Antheraea pernyi [UAA--] UCUG |UCU|| [CAAAAAUAAUUAA-------------] UUGAAAUUAAUUU ||UUUAA| UUAAAAAG |UUAAA|| AUAAA ||UUAAAAAGACGA| GAAGAC ||CCUAUAG| |AGUUU| A- ||AUAU| [UAAGUGUAAAUUUAAGUUAUUUAUAAAAUUUAUUUAUUUAUAAAUUUUGUUUA] |AUAU|| UU ||UAUU| GGGGU |GAUA|| [AAAAAAUAAAAUAAACUUUUUUUUAAUAGUAAACAUAAAUAAGUGAUU---]**

**Bombyx mori [UAA--] ACUG |UCU|| [CUUUUAUAAAAAAAAA----------] UUGAAUUUAAUUU ||UUUAA| UUAAAAAG |UUAAA|| AUUAU ||UUAAAAAGACGA| GAAGAC ||CCUAUAG| |AGUUU| U- ||AUAA| [--------UUUAUUUUGUAAAUAUUUUUAUUUUUAUAUUUUUAUAAUUUUAAA] |UUAU|| UU ||UGUU| GGGGU |GAUA|| [AAAAAAUUUAAUUAACUUUUUUUAAUAGUUUACAUUAAUAAGUGAUU----]**

**Haematobia irritans [UUA--] ACUG |UUU|| [CAUAUAAAUUUAUAA-----------] UAGAAUUUUAUUU ||UUUAG| UCAAAAAG |CUAAA|| AUUUA ||UUUAAAAGACGA| GAAGAC ||CCUAUAA| |AUCUU| U- ||AUAU| [------UUAAAAUAUUAUAUUUUUUUAGAAUUAUUUUAAUAUAAUAUUUAAUA] |AUAU|| UU ||UAUU| GGGGU |GAUA|| [UUAAAAUUUAAUAAACUUUUAAUUAUUUAAAUCAUUAAUUUAUGAAU----]**

**Ceratitis capitata [UAA--] GCUG |UUU|| [CAUAUAAAAUUAUUU-----------] UAGAAUUUUAUAU ||UUUAG| UUAAAAAG |CUAAA|| AUUUU ||AUUAAAAGACGA| GAAGAC ||CCUAUAA| |AUCUU| U- ||AUAU| [-----UUAUAAUUAUUCAAGUUUUUUGGAUUAAUUUUAUUUUUAUAAUUGUAA] |AUAU|| UU ||UGUU| GGGGU |GAUG|| [UUAAAAUUUAAUGAACUUUUAAUUAUUUAUUAAAUCAUUAAUUUAUGAAU-]**

**Tribolium castaneum [UUA--] ACUG |UCU|| [CUGAUUAAAUGAAU------------] UAUAAUUUGAAUU ||UUAAG| UGAGAAUG |CUUAA|| AUUUU ||UUAAAAAGACGA| GAAGAC ||CCUAUAG| |AGUUU| U- ||AUUU| [-----UCUAAGUUUUUAUUGGUUUUGAAGUUUUCUUUUUCUUAUUUGCUUGGA] |AAAU|| UU ||UGUU| GGGGU |GAUA|| [GGAAAAUUGAUUUAACUUUUUUUUUGUUUUUACACUGAUUAGUGAAU----]**

**Rhagophthalmus ohb' [AAU--] UCUG |UCU|| [CUUUAUUAUUUUUUA-----------] UUUAAUUUAACUU ||UUAAG| UUAAAAGG |CUUAA|| AUUAU ||UAUUAAUGACGA| GAAGAC ||CCUAUAG| |AGUUU| U- ||AUUA| [------UUAAUUUAUUAUUAUUGUUUUUAGAAUUUAAAUUUAAUAUAUUUUAA] |AAAU|| UU ||AGUU| GGGGU |GACU|| [AAAAGAUUUAAUUAACUCUUUUAAUUUAUAAUCAUAGAUUUAUGAAC----]**

**Mengenilla australiensis [-----] AUUG |UUU|| [CAAUUUAAUUAA--------------] UUGAAUUUUUUUA ||UUAAG| UGAAAAAA |CUUAA|| AUAAA ||AUUAAAAGACAA| GAAGAC ||CCUAUAA| |AUUUU| U- ||AUUA| [-------------------------------UUAAUUAAUUUAUAUUAAAGUU] |UAAU|| UU ||AAUU| GGGGA |AAUU|| [AAAAGAUUAAAAAAAUUCUUUUUUUUAUUAAUAAAGAUAAUUAAUU-----]**

**Mengenilla chobauti [-----] AUUG |UUU|| [UAAUUUGAUUAA--------------] UUGAAUUUUUUUA ||UUAAG| UGAAAAAA |CUUAA|| AUGAG ||AUUAAAAGACGA| GAAGAC ||CCUAUAA| |AUUUU| U- ||AUUA| [----------------------------UUUAAUAAUUUUUAAUUAAUUAAAG] |UAAU|| UU ||AGUU| GGGGA |AAUU|| [UAAAGAUUAAAAUAAUUCUUUUUAUAAUUAAUAAAGAUAAUUAAUU-----]**

**Triozocera sp.(2) [A----] GUUU |UUU|| [UAUAUAAUUUAU--------------] UGGAAUUUUUUGU ||UUAAG| UGAAAAAA |CUUAA|| AUUAU ||UUUAAAAGACGA| AAAGAC ||CCUAUAA| |AACUU| A- ||AUUC| [-----------------------------UUAUAAUUUAUAAAAAAUUUAUAU] |GAAU|| UU ||GAUU| GGGGA |AAUU|| [UAUAAAAUUAUAAAAUUUUAUUGAUUAAUUAAUUAAAAUUAUAAUUAUUAU]**

**Triozocera sp.(4) [-----] AAUU |UUC|| [UUCUGUAAUUUU--------------] UAGAAUUUUUUGU ||UUAAG| ???????? |?????|| ????? ||????????????| ?????? ||???????| |?????| ?? ||????| [?????????????????????????????????????????????????????] |????|| ?? ||????| ????? |????|| [???????????????????????????????????????????????????]**

**Triozocera sp.(3) [-----] AAUU |UUU|| [UUUUUUAAUUUAU-------------] UAGAAUUUUUUGU ||UUAAG| UGAAAAAA |CUUAA|| AUUAU ||AUUAAAAGACGA| AAAGAC ||CCUAUAA| |AACUU| A- ||AUUU| [-----------------------------UUAUAAUUUAAAAAAAAUUUUUAA] |AAAU|| UU ||AAUU| GGGGA |AAUU|| [UAUAAAAUUAUAAAAUUUUAUUAGUUAAUAAAUUAAAAUUAUAAAUAUUAU]**

**Corioxenos acucyrt’ [-----] UAUU |UUU|| [UUAAAUUAAAUGU-------------] AUGAAAUUUUUUU ||UUAAG| UGAAAAAA |CUUAA|| AUAAG ||UAUAAAAGACGA| GAAGAC ||CCUAUAA| |AACUU| U- ||AUUU| [---------------------AUAAUAUAAAAUAUAGAUAAUUUGAUUUUUAU] |AAAU|| UU ||AAUU| GGGGA |AAUU|| [AUUAAAUUUUAUAAAUUUUAAAGUAAAUAUUUUUAUAUUUAAAUUUUA---]**

**Myrmecolax sp.(4) [-----] ACUU |UAU|| [UUAAAUUAAUAG--------------] UUUAAAUUUUUAU ||UUAAG| UGAAAAAA |CUUAA|| AUUAU ||UAAAAAAGACGA| UCAGAC ||CCUAUAA| |AACUU| U- ||AUUA| [---------------------------UUUUUAAUAAUUUAUUUUAUUAAUAA] |UAAA|| UU ||AGUU| GGGGA |AAUU|| [UUUAAAUUAGUUAAUUUUUUUAAAUAUUAAUUUGUAUAGUAAAAUU-----]**

**Myrmecolax sp.(1) [-----] UCUU |UAU|| [UUAAAUUAUUAA--------------] UUGAAAUUUUUAU ||UUAAG| UGAAAAAA |CUUAA|| AUUAA ||AAAAAAAGACGA| UUAGAC ||CCUAUAA| |AACUU| U- ||AUUA| [---------------------------UUUUUAAUAAUUUAUUUUGUUAAUAA] |UAAA|| UU ||GGUU| GGGGA |AAUU|| [UUUAAAUUAGUUAAUUUUUUAUUUUAUUAAUUAUUAUAAUAAAAUU-----]**

**Myrmecolax sp.(2) [-----] ACUU |UAU|| [UUAAAUUAAUUA--------------] UUUAAAUUUUUAU ||UUAAG| UGAAAAAA |CUUAA|| AUUAU ||UAAAAAAGACGA| UCAGAC ||CCUAUAA| |AACUU| U- ||AUUA| [--------------------------UUUUUAAUAAUUUAUUUUUAUUAAUAA] |UAAA|| UU ||AGUU| GGGGA |AAUU|| [UUUAAAUUAGUUAAUUUUUAUAAUUAUUAAUUAAUAUAAUAAAAUU-----]**

**Myrmecholax incautus [-----] ACUU |UAU|| [UUAAAUUAGAAU--------------] UUUAAAUUUUUAU ||UUAAG| UGAAAAAA |CUUAA|| AUAUA ||UAAAAAAGACGA| UCAGAC ||CCUAUAA| |AACUU| A- ||AUUA| [---------------------------UUUAUUAAAAUUUUAUUUUUAAUUAA] |UAAU|| UU ||AAUU| GGGGA |AAUU|| [AAUAAAUUUGAAAAUUUUAUUUUUAAUGAAUUACUAUUAUAAGAAU-----]**

**Myrmecholax sp.(5) [-----] ACUU |UAU|| [UUAAAUUAAUUA--------------] UUUAAAUUUUUAU ||UUAAG| UGAAAAAA |CUUAA|| AUUAU ||UAAAAAAGACGA| UCAGAC ||CCUAUAA| |AACUU| U- ||AUUA| [---------------------------UUUUUAAUUAUUUAUUUUAUUAAAGA] |UAAA|| UU ||AGUU| GGGGA |AAUU|| [UUUAAAUUAGUUAAUUUUUUAUAAUAUUAAUUUAUAUAGUAAAAUU-----]**

**Caenocholax sp.(1) [-----] ACUU |UAU|| [-UAAUUUAAGAUU-------------] UUGAAAUUUUUAU ||UUAAG| UGAAAAAA |CUUAA|| AUUAU ||UUAAAAAGACGA| UCAGAC ||CCUAUAA| |AACUU| U- ||AUUA| [----------------------------UUAAUAAAUAAUUAAAUUUUAUUGA] |UAAA|| UU ||AGUU| GGGGA |AAUU|| [GUAAAAUUAUUAAAUUUUUAUUUUUAUUAAUUAUUUUAAUAAAAUU-----]**

**Caenocholax sp.(3) [-----] AUUU |UAU|| [-UAAAUUAAAUAA-------------] AUGAAAUUUUUAU ||UUAAG| UGAAAAAA |CUUAA|| AUAAU ||UUAAAAAGACGA| UCAGAC ||CCUAUAA| |AACUU| U- ||AUUA| [---------------------------UUAAAUUAAUUAUUUAUUAAUUUAGA] |UAAU|| UU ||AGUU| GGGGA |AAUU|| [UUGAAAUACUUAAAUUUUUAUUAUUAUUAAUUAUUAUAAUAAAGUU-----]**

**Lychnocolax sp.(2) [-----] AUUU |UAU|| [-UAAUUUAAUAAA-------------] UUGAAAUUUUUAU ||UUAAA| UGAAAAAA |UUUAA|| AUAUU ||UGAAAAAGACGA| UAAGAC ||CCUAUAA| |AACUU| A- ||AUUU| [---------------------------UUAAAUUUAUGAUUUAUAAGAUUUAA] |AAAU|| UU ||AAUU| GGGGA |AAUA|| [UUAAAAUAAAAUUAAUUUUUAAUUUAAUAAAUUUUUAUUAAAAAUAU----]**

**Xenos hamiltoni [-----] ACUU |UAU|| [-UAGUUUAAAAUA-------------] UUAAAUUUUUUAU ||UUAAG| UGAAAAAA |CUUAA|| AUUUU ||UAAAAAAGACGA| UAAGAC ||CCUAUAA| |AAUUU| U- ||AUUA| [---------------------------CUUGUUAAAAUUUAAAAUUUUAAUAA] |UAAU|| UU ||AAUU| GGGGU |AAUU|| [UAAAAUUUAUUUUUAUUUGUUUGUUCUUAUAAUUAUAAUAUU---------]**

**Xenos moutoni [-----] UCUU |UAU|| [-UGAUUUAAUUAA-------------] UUGAAUUUUUUCA ||UUAAG| UGAAAAAA |CUUAG|| AUUUU ||UAAGAAAGACGA| UAAGAC ||CCUAUAA| |AAUUU| U- ||AUUA| [---------------------------UUAUAUAAAUAUUAGAAUAUUUAUAA] |UAAA|| UU ||AAUU| GGGGA |AAUU|| [AAGUAAUUUAAUUUAUUAUAUGUUUUUUAUUAUUAUGAUAAGUU-------]**

**Xenos vesparum [-----] AUUU |UAU|| [UUAAAUUAAU-AA-------------] UUGAAUUUUUUAU ||UUAAG| UGAAAAAA |CUUAA|| AUGUU ||UAAGAAAGACGA| UAAGAC ||CCUAUAA| |AAUUU| U- ||AUUA| [---------------------------AUUUUUAUAAUUUUGAAUUUUAUGAU] |UAAU|| UU ||GGUU| GGGGA |AAUU|| [UUUCAUUAAAUUAAUGUUUGUUUUAAAUUAAUAUAAUAAUUU---------]**

**Xenos pecki [-----] GCUU |UAU|| [-UAAUUUAAAAUA-------------] UUGAAAUUUUUAU ||UUAAG| UGAAAAAA |CUUAA|| AUUUU ||UAAAAAAGACGA| UAAGAC ||CCUAUAA| |AAUUU| U- ||AUUA| [----------------------------UUUUUAAAAUUUUAAAUUUUAAAAA] |UAAU|| UU ||AAUU| GGGGU |AAUU|| [UAAAAUUUAUUUUUAUUUGUUUAUUCUUAUAAUAAUAAUAUU---------]**

**Xenos sp. [-----] ACUU |UAU|| [UUUUAUAAUUAU--------------] UUGAAUUUUUUAU ||UUAAA| UGAAAAAU |UUUAA|| AUAUU ||UAAGAAAGACGA| UAAGAC ||CCUAUAA| |AAUUU| U- ||AUUA| [-----------------------------UAUUGAAAUUUUUAAUUAAUAUAU] |UAAU|| UU ||AGUU| GGGGA |AAUU|| [AAAUUAUUGAAUUAAUUUAUGAAUUUAAUUAAUAUUAUAAAU---------]**

**Paraxenos sp. [-----] GCUU |UAU|| [UAAUAUGAGUAA--------------] UUGAAGUUUAUAU ||UUGAG| UGAAAAAG |CUUAA|| AUUUA ||AGAGAAAGACGA| UAAGAC ||CCUAUAA| |AAUUU| U- ||AUUA| [----------------------------AUAAAGUAGUUUAUAAUUAAUAUAU] |UAAU|| UU ||AGUU| GGGGC |AAUU|| [AAAAGAUUUAAUUCUUUAAAAGAUAAAUUAUGAUGGUAAUAA---------]**

**Pseudoxenos sp. [-----] GCUU |UAU|| [UAAUAUAAAUUA--------------] UUGAAGUUUUUAU ||UUAAG| UGAAAAAA |CUUAA|| AUAUU ||AAAGAUAGACGA| UAAGAC ||CCUAUAA| |AAUUU| U- ||AUUA| [-----------------------------UUAAAUUAAGUUAAAUUAGUUUAA] |UAAU|| UU ||AGUU| GGGGC |AAUU|| [GAAAUUAUUAAAUAAUUUAUUUUUAUAACUUAUGAUGUUAGUG--------]**

**Stylops mellitae [-----] GCUU |UAU|| [UAAUUUAAAAAA--------------] UUUAAUUUUUUGU ||UUAAG| ???????? |?????|| ????? ||????????????| ?????? ||???????| |?????| ?? ||????| [?????????????????????????????????????????????????????] |????|| ?? ||????| ????? |????|| [???????????????????????????????????????????????????]**

**Tridactylophagus sp. [-----] GCUU |UAU|| [UUUUAUUAAUUA--------------] UUUAAAUUUUUAU ||UUAAG| UUAAAAAA |CUUAA|| AUAUU ||UAAAAAAGACAA| UAAGAC ||CCUAUAA| |AACUU| U- ||AAUU| [--------------------------------UUAUUAAAUUUAUUAAAAUAA] |AAUU|| UU ||AAUU| GGGGA |AAUU|| [AUUAGAUUGUAUUAAUUCUUUAUUAUAUAAAUUAUUUAAUAAAAA------]**

**Halictophagus calcaratus [-----] GUUA |UAA|| [UUUAAUUAAUU---------------] ????????????? ||?????| ???????? |?????|| ????? ||????????????| ?????? ||???????| |?????| ?? ||????| [?????????????????????????????????????????????????????] |????|| ?? ||????| ????? |????|| [???????????????????????????????????????????????????]**

**Halictophagus sp.(3) [-----] ACUU |UAU|| [UUUGAAUAUUUU--------------] UAAAAUUUUUUAU ||UUAAG| UGAAAAAA |CUUAA|| AUUUA ||UAAAAAAGACAA| UAAGAC ||CCUAUAA| |AACUU| U- ||AUUU| [-------------------------------AUUAAAUUAUUUAUAAUUAAUU] |AAAU|| UU ||AAUU| GGGGA |AAUU|| [UUUAAAUAUAAUUAAUUUUAAAUAAAAUAAUUUUUUUAAAAAAAUA-----]**

**Halictophagus silwoodensis [UUUUU] UCUU |-AU|| [AAUUAUUUUUUAAAUUUAUUUCA---] ????????????? ||?????| ???????? |?????|| ????? ||????????????| ?????? ||???????| |?????| ?? ||????| [?????????????????????????????????????????????????????] |????|| ?? ||????| ????? |????|| [???????????????????????????????????????????????????]**

**Halictophagus sp.(2) [-----] ACUU |UAU|| [UUUAUUUAUUUU--------------] AUGAAAUUUUUAU ||UUAAG| UGAAAAAA |CUUAA|| AUUAA ||UAAAAAAGACAA| UAAGAC ||CCUAUAA| |AACUU| U- ||AUUU| [-------------------------------AUUAAAUUAUUUAUAAUUAUUU] |AAAU|| UU ||AGUU| GGGGA |AAUU|| [AUUAGAUAAGUAUAAUUCUAAAAAAAUUAUUUUAUUAAAAUAAUAA-----]**

**Elenchus koebelei [-----] UCUU |UAU|| [UUAAUUUAAAUAUUUUAAUUUUUU--] ????????????? ||?????| ???????? |?????|| ????? ||????????????| ?????? ||???????| |?????| ?? ||????| [?????????????????????????????????????????????????????] |????|| ?? ||????| ????? |????|| [???????????????????????????????????????????????????]**

**Elenchus sp.(1)**  **[-----] UCUU |UAU|| [UUAAUUUAAAUAUUUUAAUUUUUUAU] ------------- ||UUAAG| UGAAAAAA |CUUAA|| AUUAU ||AUAAAAAGACAA| UAAGAC ||CCUAUAA| |AACUU| U- ||AUAG| [---------------------------UUUAAGUUAAUUAUUUUUAAAUUAAA] |UUAU|| UU ||AAUU| GGGGA |AAUU|| [AUUAGAUUAAUUAAAUUCUUUUAUUUUUAAAUUAUUUAAU-----------]**

**RAA19 - H2390 H2064b’ H2064a’ H2465 H2465’ H2507 RAA20 - H2530 H2547 H2547’ H2507’ H2589**

**........ ))))) .)).)))) ........ (((.((((((((...(( ....... ))...)))))))..)))) .......... ((((((((((( ((((( ..... ))))) ..... )))))..)..)))).) ..... ??????? ...**

**Periplaneta fulig' AAU-UGAU [CCAAUUUUAUUG--AUUAUAAGAUU-] |AAAUU| |ACCUUAGG|| GAUAACAG ||CGUAAUCUUUUCUGAGA| GUUCUUA |UCGAUGAAUGGGUUUGCG|| ACCUCGAUGU ||UGGAUUAAGAU| [UUAAUUUGGGUGUAGAAUUUCAAUUGUUU----] ||AGGUC| UGUUC |GACCU|| UUAAA |AUCUUACAUGAUCUGA|| GUUCA ||GACCGGC| GUG**

**Tamolanica tamolana AUU-UGAU [CCAUAUUUAAUG--AUUAAAAGAUU-] |AAAUU| |ACCUUAGG|| GAUAACAG ||CAUAAUUCUUUUUGAGA| GUUCUUA |UCGAGAAGGGAGAUUGUG|| ACCUCGAUGU ||UGGAUUAAGAU| [UAGUUUUGGGUGUAGAUGUUCAAGAACU-----] ||AGGUC| UGUUC |GACCU|| UAGAA |AUCUUACAUGAUCUGA|| GUUUA ||AACCGGU| GUG**

**Reticulitermes fla’ GCU-UGAU [CCAUUUAUUUUG--AUUGUAAGAUU-] |AAAUU| |ACCUUAGG|| GAUAACAG ||CGUUAUCUCCCUUGAGA| GUUCUUA |UCGGCAGGGGGGUUUGCG|| ACCUCGAUGU ||UGGAUUAAGGU| [UUAUUUUCGGUGUAGGGGCUGGAAAGUUAUAUU] ||GGGUC| UGUUC |GACCU|| UUAAA |AUCUUACAUGAUCUGA|| GUUCA ||AACCGGC| GUG**

**Antheraea pernyi UUA-UGAU [CCAAUUUUAUUG--AUUAAAAGAAA-] |AAAUU| |ACCUUAGG|| GAUAACAG ||CGUAAUUUUUUCUUUUA| GUUCUUA |UAAGAGAAAAAGUUUGCG|| ACCUCGAUGU ||UGGAUUAAGAU| [AAAAUUUAAAUGCAAAAGUUUAAAAUUUU----] ||UGAUC| UGUUC |GAUCA|| UUAAA |AUCUUACAUGAUCUGA|| GUUCA ||AACCGGU| GUA**

**Bombyx mori AAA-UGAU [CCAAUUUUAUUG--AUUAAAAGAAA-] |AAAUU| |ACCUUAGG|| GAUAACAG ||CGUAAUUUUUUUUUUUA| GUUCAAA |UAAAAAGAAAAGUUUGCG|| ACCUCGAUGU ||UGGAUUAAGAU| [AAAAUUUAAAUGUAGAAGUUUAAAAUUU-----] ||UGAUC| UGUUC |GAUCA|| UUAAA |AUCUUACAUGAUCUGA|| GUUCA ||AACCGGU| GUG**

**Haematobia irritans AAU-UGAU [CCGUUAUUAACG--AUUAAAAAUUU-] |AAGUU| |ACUUUAGG|| GAUAACAG ||CGUAAUUUUUUUGGAGA| GUUCAUA |UCGAUAAAAAAGAUUGCG|| ACCUCGAUGU ||UGGAUUAAGAA| [AUAUGUUUAGGUGUAGCAGCUUAAAUAAU----] ||AAGUC| UGUUC |GACUU|| UUAAA |UUCUUACAUGAUCUGA|| GUUCA ||AACCGGC| GUA**

**Ceratitis capitata AAU-UGAU [CCAUUAUUAGUG--AUUAUAAGAUU-] |AAGUU| |ACUUUAGG|| GAUAACAG ||CGUAAUUUUUUUUGAGA| GUUCAUA |UCGACAAAAAAGUUUGCG|| ACCUCGAUGU ||UGGAUUAAGAU| [ACAUAUUUAGGUGUAGCCGCUUAAACAUA----] ||AAGUC| UGUUC |GACUU|| UUAAA |UUCUUACAUGAUCUGA|| GUUCA ||GACCGGC| GUA**

**Tribolium castaneum UGU-UGAU [CCUGAAUUUUGG--AUUAAAAGAUU-] |AAAUU| |ACCUUAGG|| GAUAACAG ||CGUAAUUUUUUUUGAAA| GUUCACA |UUUACAGAAAAGUUUGCG|| ACCUCGAUGU ||UGGAUUAAAAU| [UAAUUUUCGGUGUAGAAGCUGAAAUAUU-----] ||GGGUC| UGUUC |GACCU|| UUGAA |AUUUUACAUGAUCUGA|| GUUUA ||AACCGGC| GUG**

**Rhagophthalmus ohb' AUA-UAAU [CUAAAAUAAUA---AUUUAA--AUU-] |AAAUU| |ACCUUAGG|| GAUAACAG ||CGUAAUUUCUUUUUAUA| GUUCUUA |UUGAUAGAGAAGUUUGCG|| ACCUCGAUGU ||UGGAUUAAAAU| [AUAUUUCGGGUGAAGAAGCUUGAAUAUU-----] ||AAGUC| UGUUC |GACUU|| UUAAA |AUUUUACAUGAUCUGA|| GUUUA ||AACCGGU| GUA**

**Mengenilla australiensis UAA-UGAU [UUUUUAAAAAAG--AUAUAA-GAAU-] |AAGUU| |ACUUUAGG|| GAUAACAG ||CAUAAUUUUUUUGGAGA| GUUCUUA |UUAAUAAAAAAGAUUGUG|| ACCUCGAUGU ||UGGAUUAUAAU| [AUAUUAUUAUAUGAAGAGUUAUAAUAAUU----] ||AAGUC| UGUUC |GACUU|| --AAA |AUUUUACAUGAUCUGA|| GUUUA ||AAUCGGU| GUG**

**Mengenilla chobauti UAA-UGAU [CCUAAAAAGG----AUGAAA-GAAU-] |AAAUU| |GCUUUAGG|| GAUAACAG ||CAUAAUUUUUUUGAAGA| GUUCUUA |UUAAUAAAAAAGUUUGUG|| ACCUCGAUGU ||UGGAUUAAAAU| [AAAUUAUUAUGAGUAGAAUAAUAAUAAUA----] ||AAGUC| UGUUC |GACUU|| --AAA |AUUUUACAUGAUCUGA|| GUUUA ||AAUCGGU| GUG**

**Triozocera sp.(2) UAA-GGAU [CUUAAAUUUUAGG-AUAAAA-GA-U-] |AAGUU| |ACUUUAGG|| GAUAACAG ||CUUAAUUUUUUUUAAAA| GUUCUUA |UAUAAAAAAAAGUUUGCG|| ACCUCGA??? ||???????????| [?????????????????????????????????] ||?????| ????? |?????|| ????? |????????????????|| ????? ||???????| ???**

**Triozocera sp.(4) ???????? [??????????????????????????] |?????| |????????|| ???????? ||?????????????????| ??????? |??????????????????|| ?????????? ||???????????| [?????????????????????????????????] ||?????| ????? |?????|| ????? |????????????????|| ????? ||???????| ???**

**Triozocera sp.(3) UAA-AGAU [CUUAAAUUUUAGG-AUAGAA-GA-U-] |AAGUU| |ACUUUAGG|| GAUAACAG ||CUUAAUUUUUUUAAAAA| GUUCUUA |UAUAAAAAAAAGUUUAUG|| ACCUCGAUGU ||???????????| [?????????????????????????????????] ||?????| ????? |?????|| ????? |????????????????|| ????? ||???????| ???**

**Corioxenos acucyrt’ UAA-UGAU [CUUUAACUUAAGG-AUUUAA-GA-U-] |AAGUU| |GCUUUAGG|| GAUAACAG ||UAUAAUUUUUUUAUUAA| GUUCUUA |UAUAAAAAAAAGUUUAUU|| ACCCCGAUGU ||UGGAUUAAAAU| [AUAUUAUUUAAUGAAGUUUUUAAAUAUUAU---] ||UAGUC| UGUUC |GACUA|| --AAA |UUUUUACAUGAUCUGA|| GUUUA ||AAUCGGU| GUG**

**Myrmecolax sp.(4) UAA-UGAU [CUUUUUUUAAAGU-AUUGAA-GAAA-] |AAGUU| |GCUUUAGG|| GAUAACAG ||CAUAAUUUUUU??????| ??????? |??????????????????|| ?????????? ||???????????| [?????????????????????????????????] ||?????| ????? |?????|| ????? |????????????????|| ????? ||???????| ???**

**Myrmecolax sp.(1) AAU-UGAU [CUUUUAUUAAAGG-AUUUAA-GAAA-] |AAGUU| |GCUUUAGG|| GAUAACAG ||CAUAAUUUUUU??????| ??????? |??????????????????|| ?????????? ||???????????| [?????????????????????????????????] ||?????| ????? |?????|| ????? |????????????????|| ????? ||???????| ???**

**Myrmecolax sp.(2) UAA-UGAU [CUUUUUUUAAAGU-AUUGAA-GAAA-] |AAGUU| |GCUUUAGG|| GAUAACAG ||CAUAAUUUUUU??????| ??????? |??????????????????|| ?????????? ||???????????| [?????????????????????????????????] ||?????| ????? |?????|| ????? |????????????????|| ????? ||???????| ???**

**Myrmecholax incautus UAU-UGAU [CUUUUAUAUAAAGGAUAAAA-GAAA-] |AAGUU| |GCUUUAGG|| GAUAACAG ||CAUAAUUUUUUUUUAUA| GUUCUUA |UAGAAAAAAAAGAUUGCG|| ACCUC????? ||???????????| [?????????????????????????????????] ||?????| ????? |?????|| ????? |????????????????|| ????? ||???????| ???**

**Myrmecholax sp.(5) UAG-UGAU [CUUUUUUUAAAGU-AUUGAA-GAAA-] |AAGUU| |GCUUUAGG|| GAUAACAG ||CAUAAUUUUUU??????| ??????? |??????????????????|| ?????????? ||???????????| [?????????????????????????????????] ||?????| ????? |?????|| ????? |????????????????|| ????? ||???????| ???**

**Caenocholax sp.(1) AAU-UGAU [CUUAUAGUAUAGG-AUUGAA-GAA--] |AAGUU| |GCUUUAGG|| GAUAACAG ||CAUAAUUUUAUUU????| ??????? |??????????????????|| ?????????? ||???????????| [?????????????????????????????????] ||?????| ????? |?????|| ????? |????????????????|| ????? ||???????| ???**

**Caenocholax sp.(3) UAA-UGAU [CUUUUAAUAAAGG-AUUAAA-GAAA-] |AAGUU| |GCUUUAGG|| GAUAACAG ||CAUAAUUUUAUUUUAUA| GUUCUUA |UAGAAAAUAAAGAUUGCG|| ACCUCG???? ||???????????| [?????????????????????????????????] ||?????| ????? |?????|| ????? |????????????????|| ????? ||???????| ???**

**Lychnocolax sp.(2) UAA-AGAU [CUUUUUUUAAAGG-AUAAAA-GAAA-] |AAGUU| |GCUUUAGG|| GAUAACAG ||CAUAAUUUUUUUUUAUA| GUUCUUA |UAAAAAAUAAAGAUUGUG|| ACCUCGAUGU ||UGGAUUUAAAU| [AAAAUAAAAGGUGAAGUAGUUUUUUAUUA----] ||GAAUC| UGUUC |GAUUU|| --UUU |AUUUAACAUGAUCUGA|| GUUAA ||AA?????| ???**

**Xenos hamiltoni UAU-UGAG [CUUAAUUUUAA---AUUAAAAGAAU-] |AAAUU| |GCUUUAGG|| GAUAACAG ||CG???????????????| ??????? |??????????????????|| ?????????? ||???????????| [?????????????????????????????????] ||?????| ????? |?????|| ????? |????????????????|| ????? ||???????| ???**

**Xenos moutoni UAA-UGAU [UCUAAUAUGAGG--AUGUAA-GAUA-] |UAAUU| |GCUUUAGG|| GAUAACAG ||CGUAAUAUUUUUUUAUA| GUUCUUA |UAGAAAAAAAGGUUUGCG|| ACCUC????? ||???????????| [?????????????????????????????????] ||?????| ????? |?????|| ????? |????????????????|| ????? ||???????| ???**

**Xenos vesparum UAA-AGAU [UUUAGGAUUUAA--AUUAGAAGAUUA] |AAAUU| |GCUUUAGG|| GAUAACAG ||CGUAAUAUUUUUUUAUA| GUUCAUA |UAGAAAAAAAGGUUUGCG|| ACCUCGAUGU ||UGGAUUUAAAU| [AUUAGUAUAAAUGAAGAUUUUUAUAUUAU----] ||UAGUC| UGUUC |GACUU|| --AAU |AUUUAACAUGAUCUGA|| GUUAA ||AAUCGGU| GUG**

**Xenos pecki UAU-UGAA [CUUAAUUUUAA---AUUAAAAGAAG-] |AAAUU| |GCUUUAGG|| GAUAACAG ||CGUAAGAUUUUU?????| ??????? |??????????????????|| ?????????? ||???????????| [?????????????????????????????????] ||?????| ????? |?????|| ????? |????????????????|| ????? ||???????| ???**

**Xenos sp. UUAAGGAU [UUUUAAGUUAUAAAAUAGAAAGAAU-] |AAAUU| |GCUUUAGG|| GAUAACAG ||CGUUAUUUUUUUUUAUA| GUUCGUA |UAGAAAAAAAAGUUUGCG|| ACCUCG???? ||???????????| [?????????????????????????????????] ||?????| ????? |?????|| ????? |????????????????|| ????? ||???????| ???**

**Paraxenos sp. UUA-GGAU [CCUUGGUUUGGG--AUAUAA-GAAA-] |AAAUU| |GCUUUAGG|| GAUAACAG ||GGUAAUGAUUUUUUAUA| GUUCAUA |UAGAAAAAAGAGUUUGCG|| ACCUC????? ||???????????| [?????????????????????????????????] ||?????| ????? |?????|| ????? |????????????????|| ????? ||???????| ???**

**Pseudoxenos sp. UUAAGGAU [CUUAUUUUUAGG--AUAUUA-AAAA-] |GAAUU| |GCUUUAGG|| GAUAACAG ||CGUAAUAAAUUUUUAUA| GUUCUUA |UAGAAAAAUAGGUUUGCG|| ACCUC????? ||???????????| [?????????????????????????????????] ||?????| ????? |?????|| ????? |????????????????|| ????? ||???????| ???**

**Stylops mellitae ???????? [??????????????????????????] |?????| |????????|| ???????? ||?????????????????| ??????? |??????????????????|| ?????????? ||???????????| [?????????????????????????????????] ||?????| ????? |?????|| ????? |????????????????|| ????? ||???????| ???**

**Tridactylophagus sp. UUUGAGAU [CUUAUAUUAAGG--UAUUAAAGAAU-] |UAGUU| |GCUUUAGG|| GAUAACAG ||CAUAAUAAAAAUUUAUA| GUUCUUA |UAUAAAUUUUUGAUUGUG|| ACCUCGAUGU ||UGGAUUUAAAU| [AAAUUAUAAAUAGAAGAGUAUUUAUAAUU----] ||UAGUC| UGUUC |GACUU|| --AAA |AUUUAACAUGAUCUGA|| GUUAA ||AAUCGGU| GUG**

**Halictophagus calcaratus ???????? [??????????????????????????] |?????| |????????|| ???????? ||?????????????????| ??????? |??????????????????|| ?????????? ||???????????| [?????????????????????????????????] ||?????| ????? |?????|| ????? |????????????????|| ????? ||???????| ???**

**Halictophagus sp.(3) UAA-UGAU [CUUUUAUUAAAAG-AUUGAU-GAAU-] |UAGUU| |GCUUUAGG|| GAUAACAG ||CGUUAUAGCUUAUUAUA| GUUCUUA |UUUAAUAA??????????|| ?????????? ||???????????| [?????????????????????????????????] ||?????| ????? |?????|| ????? |????????????????|| ????? ||???????| ???**

**Halictophagus silwoodensis ???????? [??????????????????????????] |?????| |????????|| ???????? ||?????????????????| ??????? |??????????????????|| ?????????? ||???????????| [?????????????????????????????????] ||?????| ????? |?????|| ????? |????????????????|| ????? ||???????| ???**

**Halictophagus sp.(2) UUAAAGAU [CUUUUAUUAAAAG-AUUGAU-GAUU-] |AAGUU| |GCUUUAGG|| GAUAACAG ||CGUUAUAAUUUAUUAUA| GUUCUUA |UUUAAUAAAUUGAUUGCG|| ACCUCG???? ||???????????| [?????????????????????????????????] ||?????| ????? |?????|| ????? |????????????????|| ????? ||???????| ???**

**Elenchus koebelei ???????? [??????????????????????????] |?????| |????????|| ???????? ||?????????????????| ??????? |??????????????????|| ?????????? ||???????????| [?????????????????????????????????] ||?????| ????? |?????|| ????? |????????????????|| ????? ||???????| ???**

**Elenchus sp.(1)**  **???????? [??????????????????????????] |AAGUU| |GCUUUAGG|| GAUAACAG ||CAUAAUAAUUUUUUAAA| GUUCUUA |UAAAAAAAAUUGAUUAUG|| ACCUCGAUGU ||UGGAUUUAAAU| [UAUUAAUUAAAUGUAAAUGUUUAAUUAAU----] ||UAGUC| UGUUC |GACUA|| --AAA |UUUUAACAUGAUCUGA|| GUUUA ||AAUCGGU| GUG**
