# Supplementary material for: The First Molecular Phylogeny of Strepsiptera (Insecta) Reveals an Early Burst of Molecular Evolution Correlated with the Transition to Endoparasitism
Source: PLoS One. 2011 Jun 28;6(6):e21206. doi: 10.1371/journal.pone.0021206 (PMC3125182; doi:10.1371/journal.pone.0021206)
Supplement: Table S5 — Test of saturation by mitochondrial gene and codon position. *Statistics indicating little saturation. †Statistics with substantial saturation (bold). ‡Statistics indicating useless/very poor sequence for phylogenetics (bold). Ts = symmetrical T-statistic. Tns = non-symmetrical T-statistic. (DOC) [file pone.0021206.s008.doc]

| **NumOTU** | **ISS** | **ISS.C**  **Sym** | **T** | **DF** | **P** | **ISS.C**  **Asym** | **T** | **DF** | **P** | **ISS** | **ISS.C**  **Sym** | **T** | **DF** | **P** | **ISS.C**  **Asym** | **T** | **DF** | **P** |
| --- | --- | --- | --- | --- | --- | --- | --- | --- | --- | --- | --- | --- | --- | --- | --- | --- | --- | --- |
| ***cox1*** |  |  |  |  |  |  |  |  |  | ***nad1*** |  |  |  |  |  |  |  |  |
| 1^st^ codon: |  |  |  |  |  |  |  |  |  |  |  |  |  |  |  |  |  |  |
| 4 | 0.509 | 0.79 | 8.838 | 290 | 0.0000* | 0.756 | 7.754 | 290 | 0.0000* | 0.59 | 0.778 | 4.795 | 176 | 0.0000* | 0.744 | 3.926 | 176 | 0.0001* |
| 8 | 0.503 | 0.744 | 7.478 | 290 | 0.0000* | 0.631 | 3.976 | 290 | 0.0001* | 0.591 | 0.725 | 3.568 | 176 | 0.0005* | 0.61 | 0.49 | 176 | **0.6246†** |
| 16 | 0.505 | 0.71 | 6.433 | 290 | 0.0000* | 0.498 | 0.197 | 290 | **0.8438‡** | 0.598 | 0.685 | 2.447 | 176 | 0.0154* | 0.467 | 3.687 | 176 | **0.0003‡** |
| 32 | 0.514 | 0.692 | 5.55 | 290 | 0.0000* | 0.363 | 4.728 | 290 | **0.0000‡** | 0.6 | 0.668 | 2.045 | 176 | 0.0423* | 0.326 | 8.137 | 176 | **0.0000‡** |
| 2^nd^ codon: |  |  |  |  |  |  |  |  |  |  |  |  |  |  |  |  |  |  |
| 4 | 0.189 | 0.79 | 28.026 | 410 | 0.0000* | 0.756 | 26.419 | 410 | 0.0000* | 0.378 | 0.778 | 10.807 | 187 | 0.0000* | 0.744 | 9.886 | 187 | 0.0000* |
| 8 | 0.195 | 0.744 | 22.773 | 410 | 0.0000* | 0.631 | 18.094 | 410 | 0.0000* | 0.377 | 0.726 | 9.107 | 187 | 0.0000* | 0.61 | 6.087 | 187 | 0.0000* |
| 16 | 0.199 | 0.71 | 20.046 | 410 | 0.0000* | 0.498 | 11.741 | 410 | 0.0000* | 0.374 | 0.685 | 8.134 | 187 | 0.0000* | 0.467 | 2.44 | 187 | 0.0156* |
| 32 | 0.206 | 0.692 | 18.38 | 410 | 0.0000* | 0.363 | 5.913 | 410 | 0.0000* | 0.376 | 0.669 | 7.773 | 187 | 0.0000* | 0.326 | 1.304 | 187 | **0.1939‡** |
| 3^rd^ codon: |  |  |  |  |  |  |  |  |  |  |  |  |  |  |  |  |  |  |
| 4 | 0.713 | 0.79 | 3.034 | 403 | 0.0026* | 0.756 | 1.686 | 403 | **0.0925†** | 0.788  0.796  0.809  0.811 | 0.778 | 0.298 | 210 | **0.7662‡** | 0.744 | 1.285 | 210 | **0.2003‡** |
| 8 | 0.734 | 0.744 | 0.405 | 403 | **0.6857†** | 0.631 | 4.269 | 403 | **0.0000‡** | 0.796 | 0.725 | 2.210 | 210 | **0.0282‡** | 0.610 | 5.833 | 210 | **0.0000‡** |
| 16 | 0.752 | 0.71 | 1.867 | 403 | **0.0626‡** | 0.498 | 11.154 | 403 | **0.0000‡** | 0.809 | 0.685 | 4.288 | 210 | **0.0000‡** | 0.467 | 11.849 | 210 | **0.0000‡** |
| 32 | 0.761 | 0.692 | 3.058 | 403 | **0.0024‡** | 0.362 | 17.671 | 403 | **0.0000‡** | 0.811 | 0.668 | 5.275 | 210 | **0.0000‡** | 0.326 | 17.949 | 210 | **0.0000‡** |
