# Supplementary material for: The First Molecular Phylogeny of Strepsiptera (Insecta) Reveals an Early Burst of Molecular Evolution Correlated with the Transition to Endoparasitism
Source: PLoS One. 2011 Jun 28;6(6):e21206. doi: 10.1371/journal.pone.0021206 (PMC3125182; doi:10.1371/journal.pone.0021206)
Supplement: Text S1 — (DOC) [file pone.0021206.s009.doc]

­Setting Fossil Priors

The fossil Strepsiptera *Myrmecolax glaesi* [1-3] from Dominican amber (Oligo-Miocene); and *Caenocholax groehni* [4] from Baltic amber (Eocene) were used to inform the prior distributions of the time to Most Ancient Common Ancestors (tMACA) of extant *Myrmecolax*+*Stichotrema* and Myrmecolacidae, respectively. The mid-Cretaceous (approximately Cenomanian) Burmese fossil: *Cretostylops engeli* [5] was used to inform the time to Most Recent Common Ancestor (tMRCA) of Strepsiptera.

Strepsiptera are quite well represented in the fossil record [1-7], compared to some other parasitic groups with much higher species diversity (e.g. parasitic nematodes ≥ 10,500 species [8]; helminths ≥ 40,000 species [9,10]; lice (Phthiraptera) ≥ 3,000 species [11]; fleas (Siphonaptera) ≥ 2500 species [12]). A prior-based approach to date estimation is appropriate in this study because despite the presence of a comparatively rich strepsipteran fossil record, most of the specimens have a very limited morphology and are restricted to 2-3 specific timeframes in geological history, leading to significant uncertainty over their taxonomy and point of calibration. These factors restricted the number of fossils that could be usefully incorporated into the current analysis. For example, fossils like *Protelencholax* *schleei* were of such taxonomic uncertainty [1,13] that they could not be sensibly incorporated, despite their potentially central role in calibrating uninformed regions of the Strepsiptera phylogeny. Of the fossils that were used, *Caenocholax groenhi* is similar to extant *Caenocholax* species, but exhibits intermediate morphological characteristics, and is therefore most appropriately used to inform the minimum bound of an exponential distribution [14] at the directly ancestral (Myrmecolacidae) node (i.e. the *Caenocholax* tMACA, *sensu* [15]. The same principle applies to the incorporation of the fossil *Myrmecolax glaesi*. On the other hand, *Cretostylops engeli* is incorporated into the tMRCA that describes the prior distribution of extant Strepsiptera (*sensu stricto*), because it shares several apomorphies with Strepsiptera *s. str* [13]. Use of *C. engeli* to inform the tMACA of Strepsiptera is unsuitable because considerable uncertainty surrounds the strepsipteran sister-group. The resulting exponential distributions were subsequently specified with zero-offsets (in millions of years) of 25, 44 and 93.5. Means were set at 1/10 of the value of zero-offsets, corresponding to 5-95% quantiles of 25.1-32.5 for *Myrmecolax*+*Stichotrema*, 44.2-57.2 for Myrmecolacidae and 93.98-121.5 for Strepsiptera *s. str*.

Analyses were repeated in the *18S rRNA* dataset to confirm that the observed pattern of molecular evolutionary rate was not due to unique mitochondrial processes like genetic “draft” [16] and hitchhiking [17]. Mitochondrial and nuclear gene rates can be linked by “compensatory co-adaptation” [18], but the indirect involvement of rRNAs in oxidative phosphorylation pathways makes this process unlikely in this study.

Extended Acknowledgments

We are deeply indebted to the following people for providing the rare and highly valued material that contributed to this study: Z Ács, G. M. Anober, R. Andreazze, H. de Almeida Andrade, J. F. Barrera, L. Calcaterra, S. Callan, F. Collantes, J. Cook, J. A. Delgado, P. Devienne, C. Dewhurst, C. D. Eardley, R. S. Ferreira, D. Fresneau, J. Gomez, R. Graham, J. Hatting, H. Henderickx, D. Hughes, J. Jarafael, J. A. Jenzen, N. Jönsson, R. Lareau, G. Lechner, J. Majer, S. Makino, T. Malm, Y. Nakase, V. R. Ocampo, M. Pinheiro, R. S. Rejesus, P. Sandao, Tgano igask Salsa, M. Sharkey, S. Tongboonchai, S. Taylor, A. Toth, B. Viklund, R. Wharton, D. Williams, D. Windsor, J. Woolley and F. F. Xeavier.

References

1. Kinzelbach R (1979) Das erste neotropische Fossil der Fächerflügler (Stuttgarter Bernsteinsammlung: Insecta, Strepsiptera). Stuttgarter Beiträge zur Naturkunde, serie B, Geologie und Paläontologie 52: 1-14.
2. Kinzelbach R, Pohl H (1994) The fossil Strepsiptera (Insecta: Strepsiptera). Ann Entomol Soc Am 87: 59-70.
3. Kathirithamby J, Grimaldi D (1993) Remarkable stasis in lower Tertiary parasitoids: descriptions, new records, and a review of Strepsiptera in the Oligo-Miocene amber of the Dominican Republic. Ent Scand 24: 31-41.
4. Kathirithamby J, Henderickx H. (2008) First record of the Strepsiptera genus Caenocholax in Baltic amber with description of a new species. Phegea 36: 149-152.
5. Grimaldi D, Kathirithamby J, Shawaroch V (2005) Strepsiptera and triangula in Certaceous amber. Insect Syst Evol 36: 1-20.
6. Pohl H, Kinzelbach (2001) First record of a female stylopid (Strepsiptera: ?Myrecolacidae) parasite of prionomyrmecine ant (Hymenoptera: Formicidae) in Baltic amber. Insect Syst Evol 32: 143-146.
7. Pohl H, Beutel RG, Kinzelbach R (2005) Protoxenidae fam. nov. (Insecta: Strepsiptera) from Baltic amber – a ‘missing link’ in strepsipteran phylogeny. Zool Script 34: 57-69.
8. Barnes RSK (1998) The Diversity of Living Organisms. Oxford: Blackwell Science.
9. Brooks DR, McLennan DA (1993) Comparative study of adaptive radiations with an example using parasitic flatworms (Platyhelminthes: Cercomeria). Am Nat 142: 755-778.
10. Rohde K (1996) Robust phylogenies and adaptive radiations: a critical examination of methods used to identify key innovations. Am Nat 148: 481-500.
11. Barker SC (1994) Phylogeny and classification, origins, and evolution of host associations of lice. Int J Parasitol 24: 1285-1291.
12. Roberts LS, Janovy J (1996) Foundations of Parasitology. Dubuque: W.C. Brown Publishers.
13. Pohl H, Beutel RG (2005) The phylogeny of Strepsiptera (Hexapoda). Cladistics 21: 328-374.
14. Ho SYW (2007) Callibrating molecular estimates of substitution rates and divergence times in birds. J Avian Biol 38: 409-414
15. Hayward A, Stone GN. 2006. Comparative phylogeography across two trophic levels: the oak gall wasp *Andricus kollari* and its chalcid parasitoid Megastigmus stigmatizans. Mol Ecol 15:479-489.
16. Gillespie JH (2000) Genetic drift in an infinite population: the pseudohitchhiking model. Genetics 155: 909-919.
17. Bazin E, Glémin S, Galtier N (2006) Population size does not influence mitochondrial genetic diversity in animals. Science 312: 570–572.
18. Gibson JD, Niehuis O, Verrelli BC, Gadau J (2010) Contrasting patterns of selective constraints in nuclear-encoded genes of the oxidative phosphorylation pathway in holometabolous insects and their possible role in hybrid breakdown in Nasonia. Heredity 104: 310-317.
